# Assembly of Tetrahydroquinolines and 2-Benzazepines by Pd-Catalyzed Cycloadditions Involving the Activation of C(sp^3^)–H Bonds

**DOI:** 10.1021/acs.orglett.1c01594

**Published:** 2021-06-24

**Authors:** Xandro Vidal, José Luis Mascareñas, Moisés Gulías

**Affiliations:** Centro Singular de Investigación en Química Biolóxica e Materiais Moleculares (CIQUS) and Departamento de Química Orgánica, Universidade de Santiago de Compostela, 15782, Santiago de Compostela, Spain

## Abstract

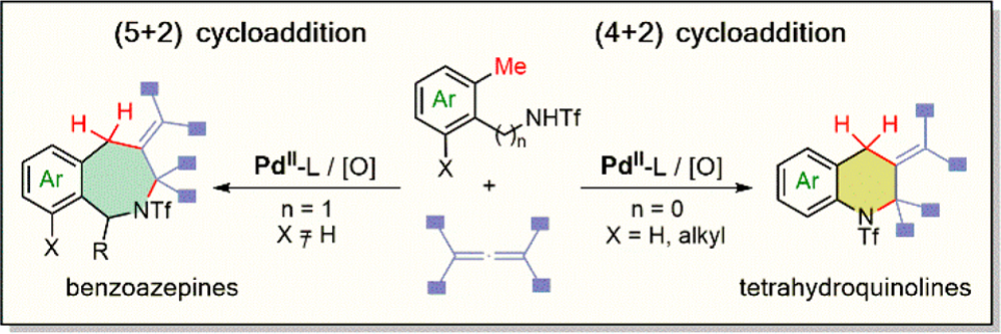

Cycloaddition reactions are among the most practical strategies to assemble cyclic products; however, they usually require the presence of reactive functional groups in the reactants. Here, we report a palladium-catalyzed formal (4 + 2) cycloaddition that involves the activation of C(sp^3^)–H bonds and provides a direct, unconventional entry to tetrahydroquinoline skeletons. The reaction utilizes amidotolyl precursors and allenes as annulation partners, and is catalyzed by Pd(II) precursors in combination with specific *N*-acetylated amino acid ligands. The reactivity can be extended to *ortho*-methyl benzylamides, which provide for the assembly of appealing tetrahydro-2-benzazepines in a formal (5 + 2) annulation process.

Azaheterocycles form the scaffold of many drugs, agrochemicals, dyes, and fragrances and can be found in many natural products. Therefore, the assembly of these skeletons in a sustainable and atom economical fashion remains a primary goal in modern organic synthesis. In this context, one of the more appealing synthetic strategies to build these type of rings consists of the use of metal-catalyzed cycloadditions involving the direct activation of C–H bonds.^1,2^ This is exemplified by the synthesis of indoles from anilides through a formal (3 + 2) oxidative cycloaddition (Scheme 1A).^3^ The reaction involves an initial C(sp^2^)–H activation to form metallacycle **A**, followed by migratory insertion of the unsaturated partner and reductive elimination (Scheme 1A). One could envision a similar annulation to build tetrahydroquinolines (THQs) instead of indoles, which is a central scaffold in many bioactive alkaloids; however this would require the use of 3-carbon cycloaddition partners, which are not obvious to identify (Scheme 1B, *left arrow*).^4^ An alternative, more attractive disconnection for THQ skeletons could be based on a (4 + 2) instead a (3 + 3) disconnection, like that shown in Scheme 1B (*right arrow*), as this would entail the use of common 2-carbon unsaturated partners. Moreover, as 4-atom components, *ortho*-methylanilines are very appealing because of their availability.

**Scheme 1:**
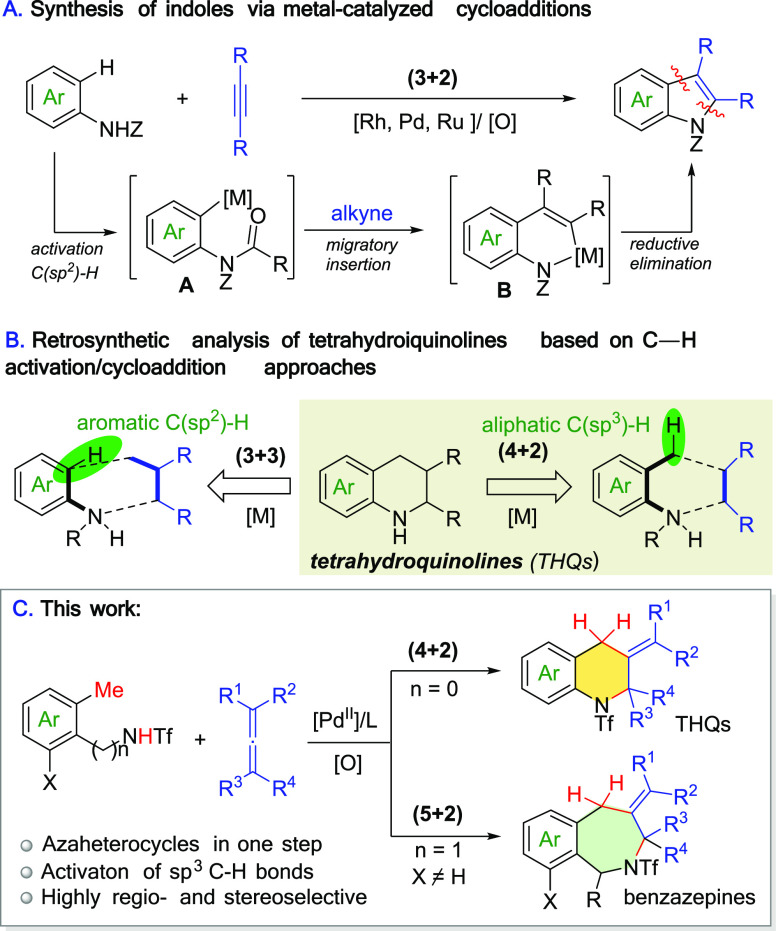
Metal-Catalyzed Annulations To Give Azaheterocycles

However, synthetic reactions that fulfill this retrosynthetic analysis, enabling a formal (4 + 2) cycloaddition between *ortho* methylanilides and unsaturated partners, are unknown. Performing this transformation using transition metal catalysis is challenging, not only because of the well-known difficulties associated with the activation of sp^3^ C–H bonds^5^ but also because the subsequent steps (migratory insertion into the C(sp^3^)–metal bond and reductive elimination) are also more problematic than in the case of substrates with sp^2^ reacting carbons. Indeed, while a vast array of different types of annulations (especially formal cycloadditions) involving the activation of aromatic C(sp^2^)–H bonds have been described, mechanistically related processes based on the activation of sp^3^ C–H bonds are very scarce.^6^

Herein, we report the first examples of transition metal formal (4 + 2) annulations involving *ortho*-methylanilides, using allenes as two-carbon partners (Scheme 1C). Importantly, we also demonstrate that the reaction, which is catalyzed by Pd(II) species, can be extended to benzylamides, providing for the direct assembly of azepines in a formal (5 + 2) cycloaddition approach.

As previously established,^7^ the presence of strong electron-withdrawing groups at the nitrogen is key for successful C–H functionalization reactions in amino aromatic substrates. Therefore, we started our investigation by examining the reactivity of 1,1,1-trifluoro-*N*-(*o*-tolyl)methanesulfonamide (**1a**, Table 1). As partners we paid attention to allenes, owing to their successful performance in previous cycloadditions involving the activation of C(sp^2^)–H bonds.^8^

**Table 1:**
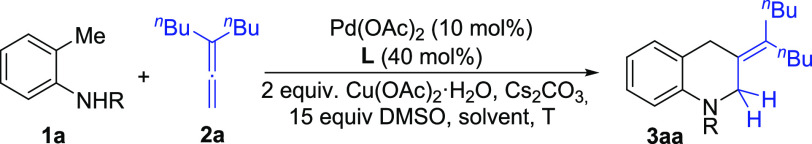
Selected Optimization Results^a^

Entry	R	Solvent	Temp	Ligand **L**	Yield^b^
1	Tf (**1a**)	Toluene	105 °C	–	<5%
2	Tf	Toluene	105 °C	Boc-Val-OH	25%
3	Tf	Toluene	105 °C	Ac-Gly-OH	42%
4	Tf	Toluene	105 °C	Ac-Ala-OH	55%
5	Tf	Toluene	105 °C	Ac-Leu-OH	55%
6	Tf	Toluene	105 °C	Formyl-Val-OH	37%
7	Tf	Toluene	105 °C	Pro-Val-OH	52%
8	Tf	Toluene	105 °C	Ac-Val-OH	60%
9	Ms (**1a′**)	Toluene	105 °C	Ac-Val-OH	39%
10	Ns (**1a″**)	Toluene	105 °C	Ac-Val-OH	33%
11	Tf	*p-*Xylene	105 °C	Ac-Val-OH	49%
12^c^	Tf	THF	105 °C	Ac-Val-OH	53%
13	Tf	2-Me THF	85 °C	Ac-Val-OH	54%
14^d^	Tf	2-Me THF	85 °C	Ac-Val-OH	61%
15^d^^,^^e^	Tf	2-Me THF	85 °C	Ac-Val-OH	56%
**16**^d^^,^^f^	**Tf**	**2-Me THF**	85 °C	**Ac-Val-OH**	**71%**

a Conditions: 0.333 mmol of **1a**, 0.167 mmol of allene **2a**, 2 mL of solvent, under air, 2 equiv of Cu(OAc)_2_·H_2_O, 1.5 equiv of Cs_2_CO_3_, 16 h.

b Yields calculated based on **2a**. Calculated by using an internal standard (entries 1–11). Isolated yields (entries 12–16).

c Reaction performed in sealed tube.

d 1 equiv of Cu(OAc)_2_·H_2_O and 1 equiv of Cs_2_CO_3_.

e 0.167 mmol of **1a**, 0.167 mmol of allene **2a**.

f Slow addition over 1 h of 0.167 mmol of allene **2a** in 1.5 mL of 2-Me THF to the reaction.

Using commercially available allene 5-vinylidenenonane (**2a**), we observed no reaction in the presence of 10 mol % of palladium acetate, and copper acetate as oxidant (in toluene at 105 °C). In line with previous reports on the role of monoprotected amino acids accelerating the rate of Pd-mediated C–H activations,^9^ we found that using 40 mol % of Boc-protected valine as ligand promotes the formation of the desired tetrahydroquinoline product **3aa** in a promising 25% yield (based on allene), as a single regioisomer (entries 1 and 2). We tested other amino acid ligands, bearing different amino-protecting groups, discovering that *N*-acetyl-*L*-valine (Ac-Val-OH) produces the best results (**3aa** formed in 60% yield, entry 8). Other oxidants, including benzoquinone or silver carbonate, are clearly inferior to copper acetate (26% and 20% yield respectively). It is possible to use substrates with other electron-withdrawing groups at the nitrogen than triflyl, such as mesyl and nosyl, albeit the reactions are less efficient (entries 9–10). Interestingly, the reaction also works using the environmentally friendly solvent methyl-THF (54% yield), which even allowed it to proceed at lower temperature (85 °C). Decreasing the amount of Cu(OAc)_2_ and Cs_2_CO_3_ to 1 equiv resulted in the product being obtained in 61% yield (entry 14), while with a lesser amount of palladium salt, conversions were not complete. Finally, we found that a slow addition of allene over a 4 h period led to an increase in yield up to 71% (entry 16).

With the optimized conditions in hand, we investigated the scope of the reaction using different types of allene partners (Scheme 2). Similar to **2a**, the 1,1-disubstituted allene vinylidenecyclohexane (**2b**) worked in good yield (61%). Symmetrical 1,3-disubstituted allenes such nona-4,5-diene (**2c**) also led to the quinoline product **3ac** in 61% yield. Gratifyingly nonsymmetrical 1,3-allenes **2d** and **2e** led to the expected products, with excellent regio- and diastereoselectivities and an up to 76% yield. Furthermore, while ethyl 2,3-butadienoate did not work, probably because of the presence of an electron-withdrawing group, electron-rich monosubstituted allenes like cyclohexylallene (**2f**) or the aryl-substituted derivative **2g** produced the cycloadducts **3af** and **3ag** as mixtures of *E*/*Z* isomers. Remarkably, trisubstituted allenes are also valid cycloaddition partners, and therefore products **3ah**, **3ai**, and **3aj** were obtained (42–73% yields).^10^

**Scheme 2:**
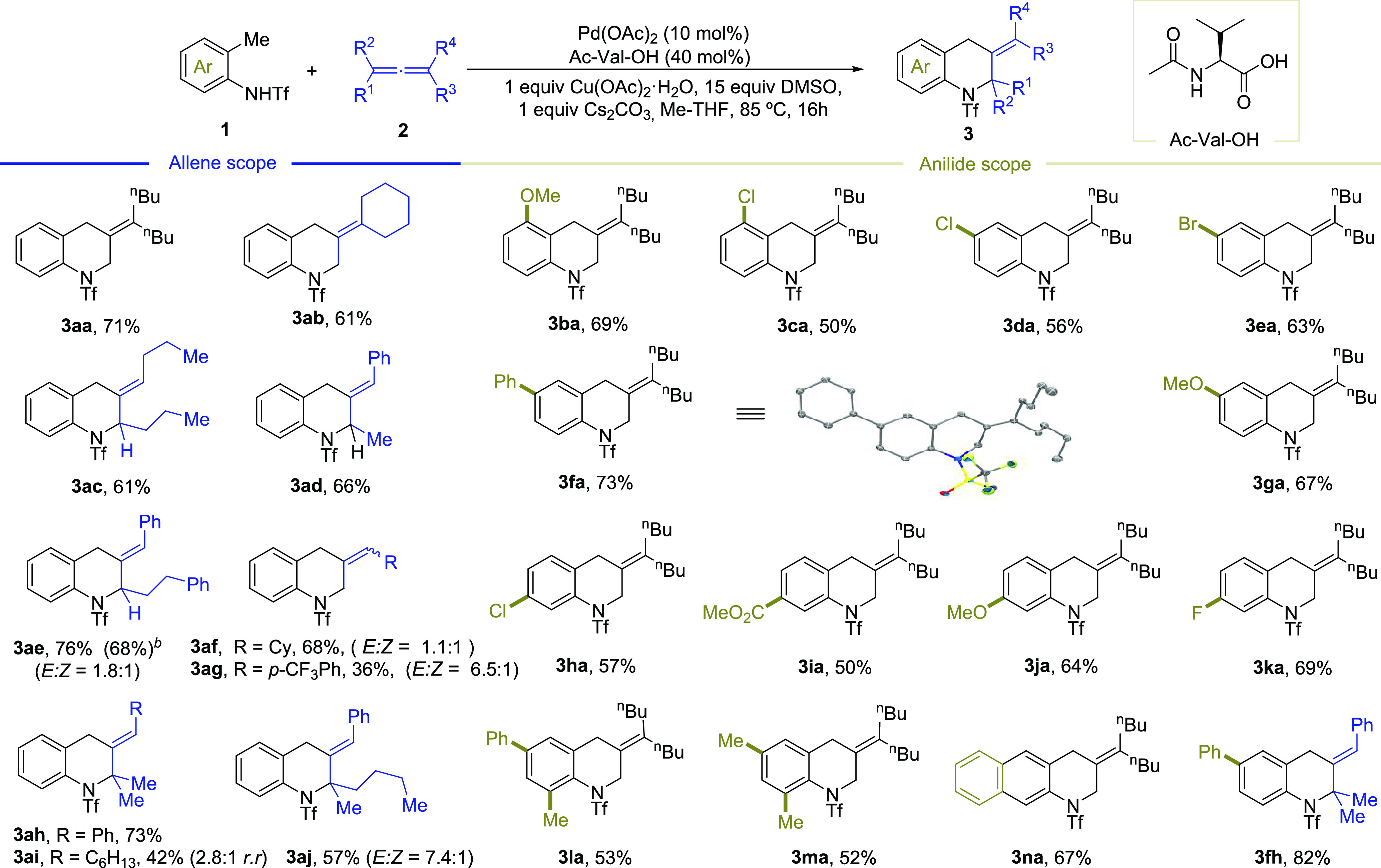
Scope of the Formal (4 + 2) Cycloaddition of *ortho*-Anilides and Allenes Conditions: 0.333 mmol of **1**, 0.167 mmol of allene **2**, 2 mL of Me-THF, under air, 16 h. Regioisomeric ratios >20:1 and *E*/*Z* ratios >20:1, unless otherwise stated. Yield after a gram-scale experiment.

The use of allenes as reaction partners is key for the success of the annulation. Alkynes, like diphenylacetylene, were essentially unreactive, while alkenes, such as ethyl acrylate, failed to give the cycloadducts, providing just traces of products resulting from addition/β-hydride elimination processes (olefination).^11^ This success with allenes is likely associated with several factors: (1) they are not as coordinating as alkynes, and thereby avoid the saturation of the metal coordination sphere to give nonactive complexes; (2) they favor the migratory insertion step owing to the formation of π-allyl intermediates; (3) they also facilitate the reductive elimination step because of the presence of an extra coordinating handle (double bond).^12^

We then explored the scope regarding the *ortho*-methyl anilides, by testing substrates **1b**–**1n**, most of which were prepared by triflation of commercially available substrates. Precursors **1b** and **1c** with substituents *ortho* to the methyl group gave the corresponding products **3ba** and **3ca** in 69% and 50% yield, respectively. Substrates equipped with substituents *meta* to the methyl group such as phenyl or methoxy, or even with halogens (chloro, bromide), also led to moderate yields (**3da**–**3ga**), exhibiting better performance for the electron-rich substrates. The reaction is also compatible with substituents *para* to the methyl group (chloro, methyl ester, methoxy and phenyl), to give the expected products (**3ha**–**3ka**, 50–69% yield). Aryl-disubstituted substrates such **1l** and **1m**, as well as naphthyl anilide **1n**, also led to effective reactions (**3la**–**3na**, 52–67% yield). Finally, as expected, the reaction is general for other allenes, as demonstrated with substrate **1f** and product **3fh**.

Running the reaction of **1a** in the presence of D_2_O or Ac(d_3_)-OD under standard conditions revealed no deuterium incorporation in neither the starting material nor the product, which suggests the C–H activation step is irreversible (eq 1). We also measured the primary kinetic isotopic effect carrying out a competition between **1a** and the deuterated analogue **1a**-*d*_3_. When the competition experiments were carried out in the same vessel, we obtained a *k*_H_/*k*_D_ ≈ 7.3. Using parallel experiments, the resulting value was 2.7. From both experiments we can conclude that the C–H bond cleavage is the turnover limiting step (eq 2).^13^
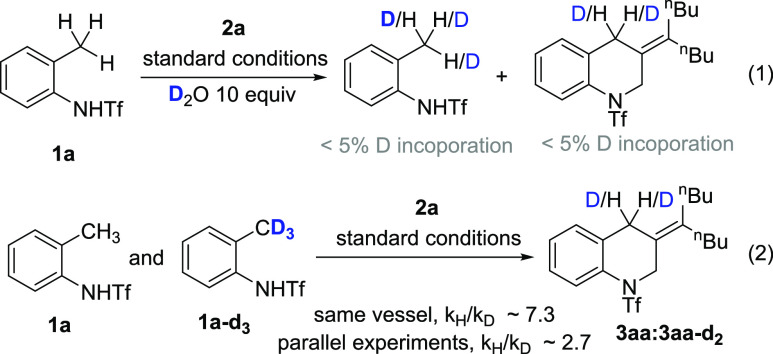
1

At this stage, we wondered whether it would be possible to use *ortho*-methyl benzylamides instead of anilides as annulation precursors. In these substrates the amide directing group is further apart from the methyl substituent, and therefore the required C(sp^3^)–H bond activation was not warranted. The annulation is synthetically relevant, as it could allow the formation of seven-membered tetrahydro-2-benzazepines, through a novel type of formal (5 + 2) annulation.

The route requires use of *ortho* disubstituted benzylamide precursors, to avoid the activation of the C(sp^2^)–H of the aromatic ring (see the Supporting Information). The reaction works well (Scheme 3) and even leads to better yields than that of the homologous anilides. The annulations were better performed using *N*-acetyl-*L*-valine as an amino acid ligand and toluene as solvent, at 105 °C. It is also beneficial to use 2 equiv of allene and of copper acetate. Several interesting azepine products (**5aa**–**5da**) were obtained from readily available starting materials in good to excellent yields (61–90% yield). Substitution in the α-position to the amino group (**5ea**, 87%) are also tolerated. The reaction can also be performed with allenes other than **2a**, illustrated with the formation of **5ch** (61%).^14^

**Scheme 3:**
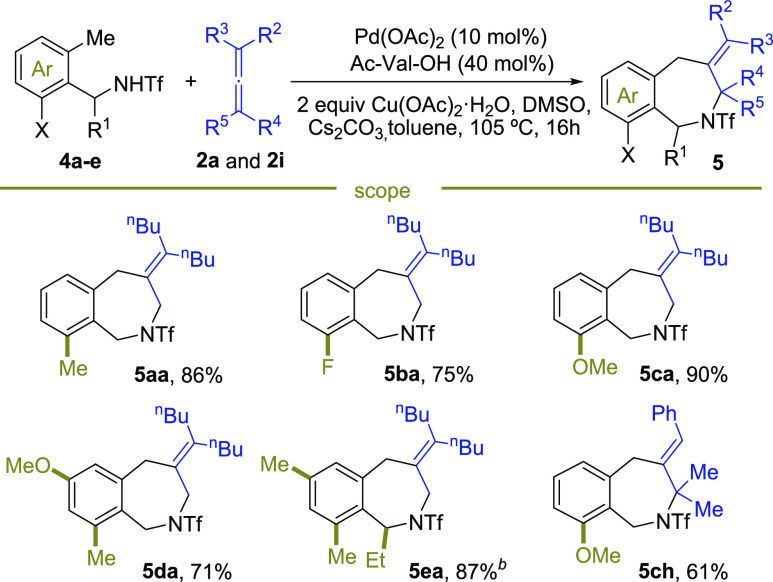
Scope of the (5 + 2) Formal Cycloaddition of *ortho*-Methylbenzylamides and Allenes Conditions: 0.167 mmol of **1a**, 0.333 mmol of allene **2a**, 2 equiv of Cu(OAc)_2_·H_2_O, 2 mL of toluene, 15 equiv of DMSO, 1.5 equiv of Cs_2_CO_3_, under air, 16 h. Racemic Ac-Val-OH was used.

We have also made a preliminary exploration of a kinetic resolution with substrates **4e** and **4f**. After a brief screening of ligands, we found out that with Boc-l-Leu-NHOMe, using standard reaction conditions at 60 °C, the cycloadduct **5fa** was produced with a promising 90:10 enantiomeric ratio (Scheme 4).^15,16^ This result indicates that we can generate optically active tetrahydrobenzazepine skeletons in only three steps from commercially available starting materials and warrants further studies to optimize the process.

**Scheme 4:**
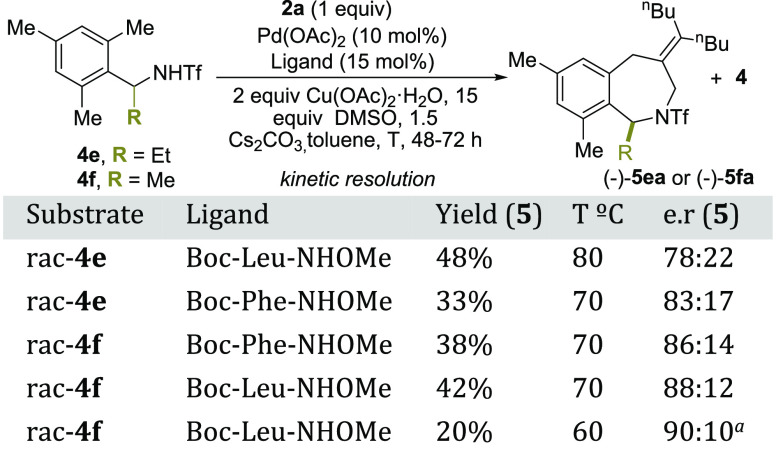
Preliminary Results on a (5 + 2) Enantioselective Annulation 2 equiv of allene.

In conclusion, we have developed a palladium-catalyzed annulation between *ortho*-methyl anilides or benzylamides and allenes involving the activation of benzylic methyl groups. The technology represents a substantial addition to the yet very scarce arsenal of metal cycloaddition tools lying on the activation of C(sp^3^)–H bonds. The approach allows a straightforward assembly of highly substituted tetrahydroquinoline or benzazepine skeletons from inexpensive and readily available starting materials.

## Supporting Information Available

Experimental procedures and spectroscopic data for new compounds; X-ray data for **3fa** and for **6** (PDF)

## Accession Codes

CCDC 2025994 and 2026002 contain the supplementary crystallographic data for this paper. These data can be obtained free of charge via www.ccdc.cam.ac.uk/data_request/cif, or by emailing data_request@ccdc.cam.ac.uk, or by contacting The Cambridge Crystallographic Data Centre, 12 Union Road, Cambridge CB2 1EZ, UK; fax: +44 1223 336033.
