# Supplementary material for: Assembly of Tetrahydroquinolines and 2-Benzazepines by Pd-Catalyzed Cycloadditions Involving the Activation of C(sp3)–H Bonds
Source: Org Lett. 2021 Jun 24;23(14):5323–8. doi: 10.1021/acs.orglett.1c01594 (PMC8488958; doi:10.1021/acs.orglett.1c01594)

Supporting Information

Assembly of tetrahydroquinolines and 2-benzazepines by Pd-catalyzed cycloadditions involving the activation of C(sp^3)-H bonds.

Xandro Vidal, José Luis Mascareñas,* Moisés Gulías*

Centro Singular de Investigación en Química Biolóxica e Materiais Moleculares (CIQUS) and Departamento de Química Orgánica, Universidade de Santiago de Compostela, 15782 Santiago de Compostela, Spain.

e-mail: joseluis.mascarenas@usc.es, moises.gulias@usc.es

Table of contents

1. GENERAL EXPERIMENTAL PROCEDURES **S3**

2. EXPERIMENTAL DATA **S4**

—General procedure for the synthesis of *o*-methylanilines and *o*-methylbenzylamines **S5**

— General procedure for the synthesis of the trifyl *o*-methylanilines and triflyl *o*-methylbenzylamines **S5**

— Optimization of the conditions for the annulation **S10**

— General procedure for the Pd-catalyzed annulation of *o*-methylanilines with allenes **S11**

—Gram-scale synthesis of 3ae and 3ae' **S17**

— General procedure for the Pd-catalyzed annulation of *o*-methylbenzylamines with allenes **S18**

—Reaction with a monomethylated benzylamide **S20**

— Synthesis of the 1,1,1-trifluoro-N-(o-tolyl)methanesulfonamide deuterated in the methyl group **S21**

— Measure of the kinetic isotopic effect (KIE) by a competition test **S22**

— Measure of the kinetic isotopic effect (KIE) by a parallel test **S23**

— Procedure for the kinetic resolution of 4f **S24**

— Procedure for the kinetic resolution of 4e **S27**

— Procedure for the hydrogenation of compound 5cg **S29**

— Procedure for the removal of trifyl group of compound 6 **S29**

—Proposed mechanism for the reaction. Derivatization of 5ch **S30**

—References **S31**

3. CRYSTALOGRAPHIC INFORMATION **S32**

—Compound 3fa **S32**

—Compound 6 **S34**

4. NMR SPECTRA **S36**

GENERAL EXPERIMENTAL PROCEDURES

Reactions were conducted in dry solvents under argon atmosphere unless otherwise stated. Dry solvents were obtained from Aldrich and used without further purification. $Pd(OAc)_2$ (98%) [3375-31-3] was obtained from Strem. All other chemicals, were purchased from Aldrich and used without further purification.

All the amino acids ligands, including Boc-Val-OH, Ac-Gly-OH, Ac-Ala-OH, Ac-Leu-OH, Formyl-Val-OH, Pro-Val-OH and Ac-Val-OH, were commercially available. They were purchased from Aldrich (except Formyl-Val-OH, that was purchased from Fluorochem) and used without further purification.

The abbreviation "rt" refers to reactions carried out at a temperature between 21-25 ºC. Reaction mixtures were stirred using Teflon-coated magnetic stir bars. High reaction temperatures were maintained using Thermowatch-controlled heating blocks. Thin-layer chromatography (TLC) was performed on silica gel plates and components were visualized by observation under UV light, and/or by treating the plates with *p*-anisaldehyde, ninhydrin, phosphomolybdic or potassium permanganate solutions, followed by heating. Flash chromatography was carried out on silica gel. Dryings were performed with anhydrous Na_2SO_4.

Concentration refers to the removal of volatile solvents via distillation using a Büchi rotary evaporator followed by high vacuum.

All palladium-catalyzed reactions were carried out without particular precautions to extrude moisture or oxygen.

^{1}H-NMR spectra were recorded at room temperature on a Varian 300 MHz or 500 MHz spectrometer in $CDCl_3$ [using $CDCl_3$ (for ^{1}H, δ = 7.26) as internal standard]. ^{19}F-NMR (282 MHz) spectra were recorded at room temperature on a Varian 300 MHz or 500 MHz spectrometer in $CDCl_3$. ^{13}C NMR spectra were recorded at room temperature on a Varian spectrometer in $CDCl_3$ [using $CDCl_3$ (for ^{13}C, δ = 77.160) as internal standard]. The following abbreviations were used to explain the multiplicities: s = singlet, d = doublet, t = triplet, q = quartet, m = multiplet, brs = broad singlet. Carbon types and structure assignments were determined from DEPT-NMR and two dimensional experiments (HSQC and HMBC, COSY and NOESY). NMR spectra were analyzed using MestReNova© NMR data processing software (www.mestrelab.com). Mass spectra were acquired using atmospheric pressure chemical ionization (APCI) and were recorded at the CACTUS facility of the University of Santiago de Compostela on Bruker micrOTOF.

Enantiomeric ratios (e.r.) were determined on an Agilent HPLC 1100 Series using commercially available chiral colums. All racemic products were prepared under the same procedure than the chiral products but with the employment of a racemic amino acid.

X-ray crystallographic analysis of **3fa** and **6** was done at the CACTUS facility of the University of Santiago de Compostela. The measurements were performed with a Bruker D8 Venture Photon III-14 using a microfocus sealed tube as diffraction source.

Amounts of isolated products are indicated independently of the scale used.

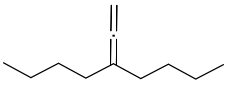
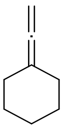
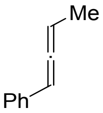
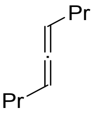
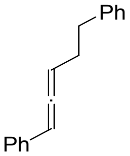

2a **2b** **2c** **2d** **2e**

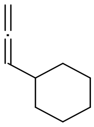
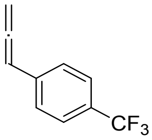
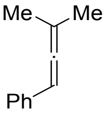
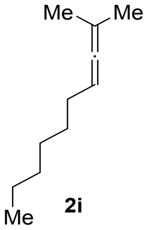
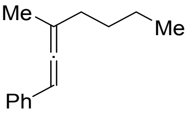

2f **2g** **2h** **2i** **2j**

Allene **2b** (vinylidenecyclohexane) and allene **2g** (propa-1,2-dien-1-ylcyclohexane) were purchased from Aldrich. Allene **2a** (5-vinylidenenonane), allene **2c** (buta-1,2-dien-1-ylbenzene), allene **2d** (nona-4,5-diene), allene **2e** (penta-1,2-diene-1,5-diyldibenzene), allene **2g** (1-(propa-1,2-dien-1-yl)-4-(trifluoromethyl)benzene), allene **2h** ((3-methylbuta-1,2-dien-1-yl)benzene), allene **2i** (2-methyldeca-2,3-diene) and allene **2j** ((3-methylhepta-1,2-dien-1-yl)benzene) were prepared according procedures reported in the literature.[1]

EXPERIMENTAL DATA

General procedure for the synthesis of *o*-methylanilines:

o-toluidine, 3-methoxy-2-methylaniline, 3-chloro-2-methylaniline, 4-chloro-2-methylaniline, 4-bromo-2-methylaniline, 4-methoxy-2-methylaniline, 5-chloro-2-methylaniline, methyl 3-amino-4-methylbenzoate, 5-methoxy-2-methylaniline, 5-fluoro-2-methylaniline and 2,4,6-trimethylaniline were commercially available. 3,5-dimethyl-[1,1'-biphenyl]-4-amine, 3-methyl-[1,1'-biphenyl]-4-amine and 3-methylnaphthalen-2-amine were synthesized with methods previously reported in literature.[2] All the spectral data recorded for these compounds were in agreement with those found in the corresponding literature.[3]

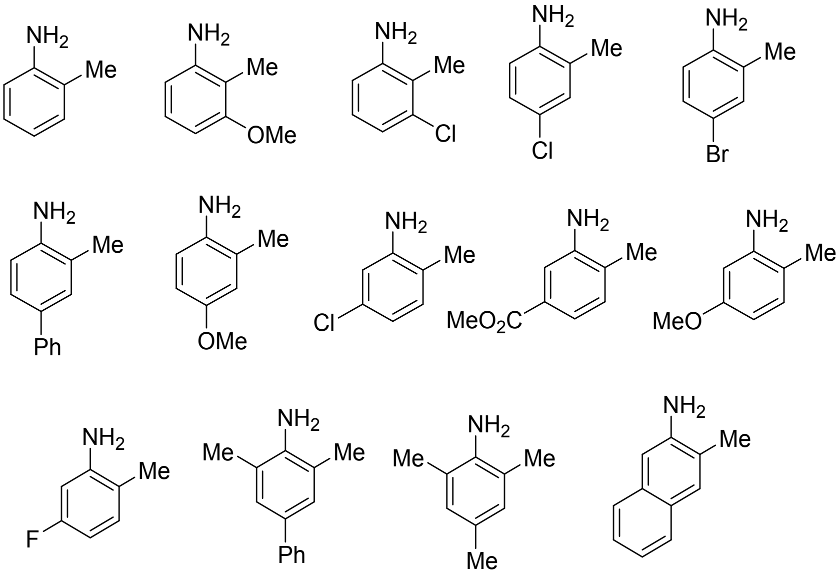

General procedure for the synthesis of *o*-methylbenzylamines:

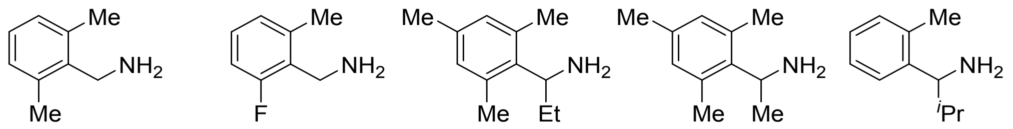

(2,6-dimethylphenyl)methanamine and (2-fluoro-6-methylphenyl)methanamine were commercially available. 1-mesitylpropan-1-amine, 1-mesitylethan-1-amine were synthesized with a method previously reported in literature.[4] 2-methyl-1-(o-tolyl)propan-1-amine was synthesized with a method previously reported in literature.[5]

General procedure for the synthesis of the triflyl *o*-methylanilines (1a-1n) and triflyl *o*-methylbenzylamines (4a, 4b, 4e), from the precursor amines, exemplified for 1a

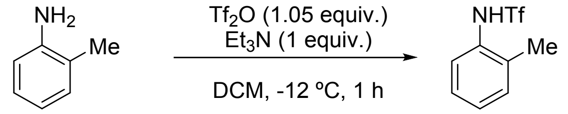

1a

To a solution of *o*-toludine (2.00 g, 18.66 mmol) in dichloromethane (37 mL) under argon atmosphere was added triethylamine (2.6 mL, 1 equiv.) at -12ºC. After the solution was stirred 5 minutes at that temperature, trifluoromethanesulfonic anhydride (3.3 mL, 1.05 equiv.) was added dropwise. The reaction was stirred for 1 h at that temperature before being quenched with water. The organic layer was separated and the aqueous layer extracted with dichloromethane. The combined organic phase was washed with brine and then dried over Na_2SO_4. Evaporation and column chromatography on silica gel (hexanes:diethylether; 80:20) afforded **1,1,1-trifluoro-N-(o-tolyl)methanesulfonamide** (**1a**) as a pale yellow solid (3.61 g, 81% yield). **^{1}H NMR** (300 MHz, $CDCl_3$) δ 7.44 – 7.36 (m, 1H), 7.29 – 7.19 (m, 3H), 6.71 (brs, 1H), 2.38 (s, 3H). **^{19}F NMR** (282 MHz, $CDCl_3$) δ -76.50. **^{13}C NMR** (75 MHz, $CDCl_3$) δ 133.9 (C), 132.1 (C), 131.4 (CH), 128.6 (CH), 127.4 (CH), 126.4 (CH), 119.9 (d, J = 322.3 Hz, C), 17.9 (CH_3). **HRMS** [APCI]: *m/z* calculated for $C_8H_8F_3NO_2S$ $[M]^+$: 239.0222, found 239.0225. Data in agreement with those reported in literature.[6]

1,1,1-Trifluoro-*N*-(3-methoxy-2-methylphenyl)methanesulfonamide (1b)

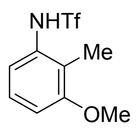

1b

(1.28 g, 81% yield), obtained as a white solid. **^{1}H NMR** (300 MHz, $CDCl_3$) δ 7.19 (t, J = 8.0 Hz, 1H), 7.02 (d, J = 8.1 Hz, 1H), 6.85 (d, J = 8.2 Hz, 1H), 6.57 (brs, 1H), 3.84 (s, 3H), 2.22 (s, 3H). **^{19}F NMR** (282 MHz, $CDCl_3$) δ -76.41. **^{13}C NMR** (75 MHz, $CDCl_3$) δ 158.4 (C), 132.7 (C), 127.0 (CH), 123.3 (C), 119.9 (d, J = 322.4 Hz, C), 118.6 (CH), 110.2 (CH), 55.9 (CH_3), 10.7 (CH_3). **HRMS** [APCI]: *m/z* calculated for $C_9H_{11}F_3NO_3S$ $[M+H]^+$: 270.0406, found 270.0407.

***N*-(3-Chloro-2-methylphenyl)-1,1,1-trifluoromethanesulfonamide (1c)**

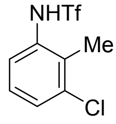

1c

(1.70 g, 88% yield), obtained as a white solid. **^{1}H NMR** (300 MHz, $CDCl_3$) δ 7.40 (d, J = 8.0 Hz, 1H), 7.33 (d, J = 8.0 Hz, 1H), 7.18 (t, J = 8.0 Hz, 1H), 6.77 (brs, 1H), 2.43 (s, 3H). **^{19}F NMR** (282 MHz, $CDCl_3$) δ -76.20. **^{13}C NMR** (75 MHz, $CDCl_3$) δ 135.9 (C), 133.2 (C), 133.1 (C), 129.8 (CH), 127.5 (CH), 125.6 (CH), 119.8 (d, J = 322.2 Hz, C), 15.4 (CH_3). **HRMS** [APCI]: *m/z* calculated for $C_8H_7ClF_3NO_2S$ $[M]^+$: 272.9833, found 272.9832.

N-(4-Chloro-2-methylphenyl)-1,1,1-trifluoromethanesulfonamide (1d)

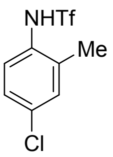

1d

(1.82 g, 94% yield), obtained as a white solid. **^{1}H NMR** (300 MHz, $CDCl_3$) δ 7.39 – 7.22 (m, 3H), 6.70 (brs, 1H), 2.39 (s, 3H). **^{19}F NMR** (282 MHz, $CDCl_3$) δ -76.43. **^{13}C NMR** (75 MHz, $CDCl_3$) δ 135.9 (C), 134.3 (C), 131.3 (CH), 130.6 (C), 127.8 (CH), 127.5 (CH), 119.8 (d, *J* = 322.1 Hz, C), 17.9 (CH_3). **HRMS** [APCI]: *m/z* calculated for $C_8H_7ClF_3NO_2S$ $[M]^+$: 272.9833, found 272.9830.

N-(4-Bromo-2-methylphenyl)-1,1,1-trifluoromethanesulfonamide (1e)

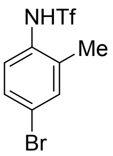

1e

(1.67 g, 81% yield), obtained as a white solid. **^{1}H NMR** (300 MHz, $CDCl_3$) δ 7.43 (d, *J* = 2.3 Hz, 1H), 7.37 (dd, *J* = 8.6, 2.4 Hz, 1H), 7.26 (d, *J* = 8.5 Hz, 1H), 6.68 (s, 1H), 2.35 (s, 3H). **^{19}F NMR** (282 MHz, $CDCl_3$) δ -76.44. **^{13}C NMR** (75 MHz, $CDCl_3$) δ 136.0 (C), 134.2 (CH), 131.2 (C), 130.5 (CH), 127.9 (CH), 122.3 (C), 119.8 (d, *J* = 322.2 Hz, C), 17.8 (CH_3). **HRMS** [APCI]: *m/z* calculated for $C_8H_7BrF_3NO_2S$ $[M]^+$: 316.9327, found 316.9327.

1,1,1-Trifluoro-*N*-(3-methyl-[1,1'-biphenyl]-4-yl)methanesulfonamide (1f)

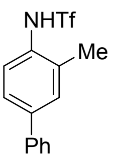

1f

(1.16 g, 84% yield), obtained as a white solid. **^{1}H NMR** (300 MHz, $CDCl_3$) δ 7.57 (d, *J* = 7.7 Hz, 2H), 7.52 – 7.33 (m, 7H), 2.45 (s, 3H). **^{19}F NMR** (282 MHz, $CDCl_3$) δ -76.39. **^{13}C NMR** (75 MHz, $CDCl_3$) δ 141.5 (C), 139.9 (C), 134.3 (C), 131.2 (C), 130.0 (CH), 129.0 (CH), 127.9 (CH), 127.2 (CH), 126.8 (CH), 126.0 (CH), 119.9 (d, *J* = 322.1 Hz, C), 18.1 (CH_3). **HRMS** [APCI]: *m/z* calculated for $C_{14}H_{13}F_3NO_2S$ $[M+H]^+$: 316.0614, found 316.0612.

1,1,1-Trifluoro-*N*-(4-methoxy-2-methylphenyl)methanesulfonamide (1g)

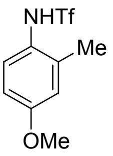

1g

(1.49 g, 71% yield), obtained as a white solid. **^{1}H NMR** (300 MHz, $CDCl_3$) δ 7.18 (d, *J* = 8.5 Hz, 1H), 6.73 – 6.63 (m, 2H), 6.40 (brs, 1H), 3.72 (s, 3H), 2.28 (s, 3H). **^{19}F NMR** (282 MHz, $CDCl_3$) δ -76.41. **^{13}C NMR** (75 MHz, $CDCl_3$) δ 159.7 (C), 137.3 (C), 129.3 (CH), 124.4 (C), 119.9 (d, *J* = 321.9 Hz, C), 116.5 (CH), 112.3 (CH), 55.6 (CH_3), 18.4 (CH_3). **HRMS** [APCI]: *m/z* calculated for $C_9H_{10}F_3NO_3S$ $[M]^+$: 269.0328, found 269.0327.

N-(5-Chloro-2-methylphenyl)-1,1,1-trifluoromethanesulfonamide (1h)

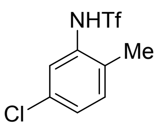

1h

(1.37 g, 71% yield), obtained as a white solid. **^{1}H NMR** (300 MHz, $CDCl_3$) δ 7.48 (s, 1H), 7.32 – 7.22 (m, 2H), 6.75 (brs, 1H), 2.39 (s, 3H). **^{19}F NMR** (282 MHz, $CDCl_3$) δ -76.48. **^{13}C NMR** (75 MHz, $CDCl_3$) δ 133.0 (C), 132.6 (C), 132.2 (CH), 131.8 (C), 128.6 (CH), 126.0 (CH), 119.8 (q, *J* = 322.0 Hz, C), 17.4 (CH_3). **HRMS** [APCI]: *m/z* calculated for $C_8H_7ClF_3NO_2S$ $[M]^+$: 272.9833, found 272.9833.

Methyl 4-methyl-3-((trifluoromethyl)sulfonamido)benzoate (1i)

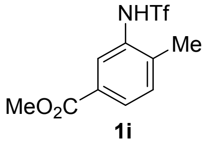

1i

(1.32 g, 73% yield), obtained as a white solid. **^{1}H NMR** (300 MHz, $CDCl_3$) δ 8.01 (s, 1H), 7.90 (d, *J* = 8.0 Hz, 1H), 7.59 (s, 1H), 7.34 (d, *J* = 8.0 Hz, 1H), 3.91 (s, 3H), 2.45 (s, 3H). **^{19}F NMR** (282 MHz, $CDCl_3$) δ -76.60. **^{13}C NMR** (75 MHz, $CDCl_3$) δ 166.7 (C), 140.4 (C), 132.6 (C), 131.6 (CH), 129.6 (CH), 129.3 (C), 128.2 (CH), 119.9 (d, *J* = 322.2 Hz, C), 52.7 (CH_3), 18.3 (CH_3). **HRMS** [APCI]: *m/z* calculated for $C_{10}H_{11}F_3NO_4S$ $[M+H]^+$: 298.0355, found 298.0358.

1,1,1-Trifluoro-*N*-(5-methoxy-2-methylphenyl)methanesulfonamide (1j)

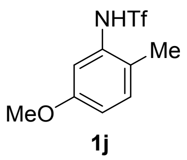

1j

(1.13 g, 72% yield), obtained as a white solid. **^{1}H NMR** (300 MHz, $CDCl_3$) δ 7.15 (d, *J* = 8.4 Hz, 1H), 6.97 (s, 1H), 6.80 (d, *J* = 8.5 Hz, 1H), 6.63 (brs, 1H), 3.79 (s, 3H), 2.28 (s, 3H). **^{19}F NMR** (282 MHz,

$CDCl_3$) δ -76.52. **^{13}C NMR** (75 MHz, $CDCl_3$) δ 158.6 (C), 132.7 (C), 131.8 (CH), 124.9 (C), 119.9 (d, *J* = 322.0 Hz, C), 114.1 (CH), 111.5 (CH), 55.6 (CH_3), 16.9 (CH_3). **HRMS** [APCI]: *m/z* calculated for $C_9H_{10}F_3NO_3S$ $[M]^+$: 269.0328, found 269.0331.

1,1,1-Trifluoro-*N*-(5-fluoro-2-methylphenyl)methanesulfonamide (1k)

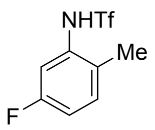

1k

(1.63 g, 79% yield), obtained as a white solid. **^{1}H NMR** (300 MHz, $CDCl_3$) δ 7.33 – 7.20 (m, 2H), 7.02 (td, *J* = 8.3, 2.6 Hz, 1H), 6.92 (brs, 1H), 2.38 (s, 3H). **^{19}F NMR** (282 MHz, $CDCl_3$) δ -76.57, -114.59 (q, *J* = 8.1 Hz). **^{13}C NMR** (75 MHz, $CDCl_3$) δ 161.3 (d, *J* = 246.0 Hz, C), 133.0 (d, *J* = 10.2 Hz, C), 132.2 (d, *J* = 8.7 Hz, CH), 128.3 (d, *J* = 3.7 Hz, C), 119.8 (d, *J* = 322.2 Hz, C), 115.2 (d, *J* = 20.9 Hz, CH), 112.8 (d, *J* = 25.0 Hz, CH), 17.2 (CH_3). **HRMS** [APCI]: *m/z* calculated for $C_8H_7F_4NO_2S$ $[M]^+$: 257.0128, found 257.0126.

***N*-(3,5-Dimethyl-[1,1'-biphenyl]-4-yl)-1,1,1-trifluoromethanesulfonamide (1l)**

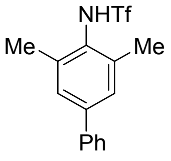

1l

(0.28 g, 74% yield), obtained as a white solid. **^{1}H NMR** (300 MHz, $CDCl_3$) δ 7.62 – 7.52 (m, 2H), 7.51 – 7.31 (m, 5H), 6.38 (s, 1H), 2.47 (s, 6H). **^{19}F NMR** (282 MHz, $CDCl_3$) δ -76.77. **^{13}C NMR** (75 MHz, $CDCl_3$) δ 142.2 (C), 139.9 (C), 138.5 (C), 129.8 (C), 129.0 (CH), 128.0 (CH), 127.9 (CH), 127.3 (CH), 119.7 (d, *J* = 321.9 Hz, C), 19.2 (CH_3). **HRMS** [APCI]: *m/z* calculated for $C_{15}H_{14}F_3NO_2S$ $[M]^+$: 329.0692, found 329.0691.

1,1,1-Trifluoro-*N*-mesitylmethanesulfonamide (1m)

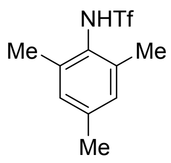

1m

(2.10g, 93% yield), obtained as a white solid. **^{1}H NMR** (300 MHz, $CDCl_3$) δ 6.94 (s, 2H), 6.37 (s, 1H), 2.36 (s, 6H), 2.29 (s, 3H). **^{19}F NMR** (282 MHz, $CDCl_3$) δ -76.89. **^{13}C NMR** (75 MHz, $CDCl_3$) δ 139.4 (C), 137.8 (C), 129.9 (CH), 128.0 (C), 119.7 (d, *J* = 321.8 Hz, C), 21.0 (CH_3), 18.8 (CH_3). **HRMS** [APCI]: *m/z* calculated for $C_{10}H_{12}F_3NO_2S$ $[M]^+$: 267.0535, found 267.0536.

1,1,1-Trifluoro-*N*-(3-methylnaphthalen-2-yl)methanesulfonamide (1n)

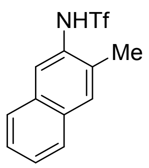

1n

(0.80 g, 87% yield), obtained as a white solid. **^{1}H NMR** (300 MHz, $CDCl_3$) δ 7.91 (s, 1H), 7.87 – 7.74 (m, 2H), 7.73 (s, 1H), 7.54 – 7.44 (m, 2H), 6.58 (brs, 1H), 2.53 (s, 3H). **^{19}F NMR** (282 MHz, $CDCl_3$) δ -76.31. **^{13}C NMR** (75 MHz, $CDCl_3$) δ 132.8 (C), 132.3 (C), 130.9 (C), 130.5 (C), 129.8 (CH), 128.0 (CH), 127.1 (CH), 126.4 (CH), 124.6 (CH), 120.0 (d, *J* = 322.5 Hz, C), 18.4 (CH_3). **HRMS** [APCI]: *m/z* calculated for $C_{12}H_{10}F_3NO_2S$ $[M]^+$: 289.0379, found 289.0376.

***N*-(2,6-Dimethylbenzyl)-1,1,1-trifluoromethanesulfonamide (4a)**

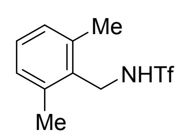

4a

(1.63 g, 82% yield), obtained as a white solid. **^{1}H NMR** (300 MHz, $CDCl_3$) δ 7.20 – 7.02 (m, 3H), 4.67 (brs, 1H), 4.48 (d, *J* = 4.6 Hz, 2H), 2.40 (s, 6H). **^{19}F NMR** (282 MHz, $CDCl_3$) δ -77.26. **^{13}C NMR** (75 MHz, $CDCl_3$) δ 137.8 (C), 130.8 (C), 129.2 (CH), 128.9 (CH), 119.9 (d, *J* = 321.6 Hz, C), 42.7 (CH_2), 19.5 (CH_3). **HRMS** [APCI]: *m/z* calculated for $C_{10}H_{12}F_3NO_2S$ $[M]^+$: 267.0535, found 267.0539.

1,1,1-Trifluoro-*N*-(2-fluoro-6-methylbenzyl)methanesulfonamide (4b)

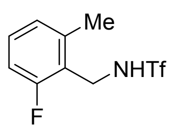

4b

(1.58 g, 86% yield), obtained as a yellow solid. **^{1}H NMR** (300 MHz, $CDCl_3$) δ 7.33 – 7.19 (m, 1H), 7.09 – 6.92 (m, 2H), 5.18 (s, 1H), 4.54 (d, *J* = 5.6 Hz, 2H), 2.44 (s, 3H). **^{19}F NMR** (282 MHz, $CDCl_3$) δ -77.75, 118.51 (m). **^{13}C NMR** (75 MHz, $CDCl_3$) δ 161.9 (d, *J* = 246.0 Hz, C), 139.4 (d, *J* = 3.1 Hz, C), 130.4 (d, *J* = 9.5 Hz, CH), 126.6 (d, *J* = 3.1 Hz, CH), 120.9 (d, *J* = 13.9 Hz, C), 119.7 (d, *J* = 321.2 Hz, C), 113.3 (d, *J* = 22.0 Hz, CH), 39.3 (d, *J* = 5.2 Hz, CH_2), 18.9 (d, *J* = 2.7 Hz, CH_3). **HRMS** [APCI]: *m/z* calculated for $C_9H_9F_4NO_2S$ $[M]^+$: 271.0285, found 271.0284. Data in agreement with those reported in literature.[7]

1,1,1-Trifluoro-*N*-(1-mesitylpropyl)methanesulfonamide (4e)

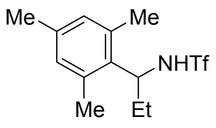

4e

(1.93 g, 55% yield), obtained as a white solid. **^{1}H NMR** (300 MHz, $CDCl_3$) δ 6.85 (s, 2H), 5.54 (d, *J* = 8.3 Hz, 1H), 5.00 (q, *J* = 8.1 Hz, 1H), 2.37 (s, 6H), 2.26 (s, 3H), 2.15 – 1.82 (m, 2H), 0.99 (t, *J* = 7.4 Hz, 3H). **^{19}F NMR** (282 MHz, $CDCl_3$) δ -78.27. **^{13}C NMR** (75 MHz, $CDCl_3$) δ 137.4 (C), 133.2 (C), 119.6 (q, *J* = 321.1 Hz, C), 57.5 (CH), 28.4 (CH_2), 20.9 (CH_3), 20.8 (CH_3), 11.2 (CH_3). **HRMS** [APCI]: *m/z* calculated for $C_{13}H_{18}F_3NO_2S$ $[M]^+$: 309.1005, found 309.1003.

1,1,1-trifluoro-N-(1-mesitylethyl)methanesulfonamide (4f)

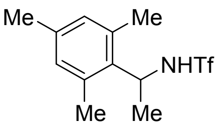

4f

(0.41 g, 33% yield), obtained as a white solid. **^{1}H NMR** (300 MHz, $CDCl_3$) δ 6.85 (s, 2H), 5.44 – 5.32 (m, 1H), 5.32 – 5.19 (m, 1H), 2.39 (s, 6H), 2.26 (s, 3H), 1.63 (d, *J* = 6.5 Hz, 3H). **^{19}F NMR** (282 MHz, $CDCl_3$) δ -77.88. **^{13}C NMR** (75 MHz, $CDCl_3$) δ 137.6 (C), 134.9 (C), 134.2 (C), 130.7 (CH), 119.6 (d, *J* = 321.1 Hz), 51.6 (CH), 21.5 (CH_3), 20.9 (CH_3), 20.7 (CH_3). **HRMS** [APCI]: *m/z* calculated for $C_{12}H_{16}F_3NO_2S$ $[M]^+$: 295.0848, found 295.0850.

1,1,1-trifluoro-N-(2-methyl-1-(o-tolyl)propyl)methanesulfonamide (4g)

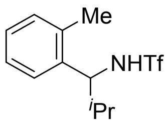

4g

(0.45 g, 91% yield), obtained as a white solid. **^{1}H NMR** (300 MHz, $CDCl_3$) δ 7.33 – 7.06 (m, 4H), 5.73 (brs, 1H), 4.61 (t, *J* = 9.0 Hz, 1H), 2.37 (s, 3H), 2.11 – 1.89 (m, *J* = 6.7 Hz, 1H), 1.09 (d, *J* = 6.7 Hz, 3H), 0.89 (d, *J* = 6.8 Hz, 3H). **^{19}F NMR** (282 MHz, $CDCl_3$) δ -77.69. **^{13}C NMR** (75 MHz, $CDCl_3$) δ 138.8 (C), 135.0 (C), 130.8 (CH), 127.7 (CH), 126.6 (CH), 125.4 (CH), 119.5 (d, *J* = 321.2 Hz, C), 61.5 (CH), 34.7 (CH), 19.5 (CH_3), 19.4 (CH_3), 18.5 (CH_3). **HRMS** [APCI]: *m/z* calculated for $C_{12}H_{16}F_3NO_2S$ $[M]^+$: 295.0848, found 295.0855.

General procedure for the synthesis of the triflyl *o*-methylbenzylamines (4c-4d) from the corresponding alcohols, exemplified for 4c.

(2-methoxy-6-methylphenyl)methanol was commercially available. (4-methoxy-2,6-dimethylphenyl)methanol was synthesized from 4-methoxy-2,6-dimethylbenzaldehyde with a method previously reported in literature. All spectral data recorded were in agreement with those reported in the literature. [8]

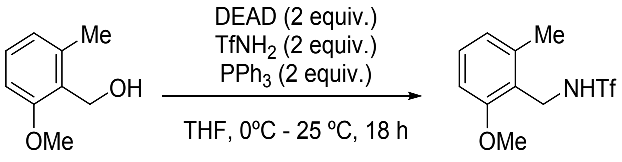

4c

To a solution of (2-methoxy-6-methylphenyl)methanol (600.0 mg, 3.942 mmol), trifluoromethane sulfonamide (1.17 g, 2 equiv.), and triphenylphosphine (2.07 g, 2 equiv.) in THF (20 mL) at 0 °C was added DEAD (3.1 mL, 40% in toluene, 2 equiv.). The reaction mixture was stirred at room temperature overnight, and was quenched with water, then diluted with ethyl acetate. The combined organic phases were washed with brine and then dried over Na_2SO_4. Evaporation and column chromatography on silica gel (hexanes:diethylether; 90:10) afforded **1,1,1-trifluoro-N-(2-methoxy-6-methylbenzyl)methanesulfonamide 4c** as a white solid (589 mg, 53%). **^{1}H NMR** (300 MHz, $CDCl_3$) δ 7.22 (t, *J* = 8.0 Hz, 1H), 6.83 (d, *J* = 7.7 Hz, 1H), 6.78 (d, *J* = 8.4 Hz, 1H), 5.59 (t, *J* = 5.9 Hz, 1H), 4.48 (d, *J* = 5.8 Hz, 2H), 3.87 (s, 3H), 2.40 (s, 3H). **^{19}F NMR** (282 MHz, $CDCl_3$) δ -77.96. **^{13}C NMR** (75 MHz, $CDCl_3$) δ 158.1 (C), 137.9 (C), 129.5 (CH), 123.3

(CH), 122.2 (C), 119.8 (d, J = 321.4 Hz, C), 108.3 (CH), 55.6 (CH_3), 40.7 (CH_2), 19.2 (CH_3). **HRMS** [APCI]: *m/z* calculated for $C_{10}H_{12}F_3NO_3S$ $[M]^+$: 283.0485, found 283.0487.

1,1,1-Trifluoro-N-(4-methoxy-2,6-dimethylbenzyl)methanesulfonamide (4d)

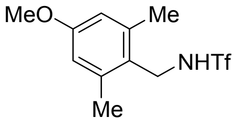

4d

(0.54 g, 50% yield), obtained as a white solid. **^{1}H NMR** (300 MHz, $CDCl_3$) δ 6.56 (s, 2H), 4.87 (brs, 1H), 4.42 (d, J = 4.5 Hz, 2H), 3.76 (s, 3H), 2.35 (s, 6H). **^{19}F NMR** (282 MHz, $CDCl_3$) δ -77.28. **^{13}C NMR** (75 MHz, $CDCl_3$) δ 159.5 (C), 139.3 (C), 123.2 (C), 120.0 (d, J = 321.8 Hz, C), 114.2 (CH), 55.3 (CH_3), 42.4 (CH_2), 19.7 (CH_3). **HRMS** [APCI]: *m/z* calculated for $C_{11}H_{15}F_3NO_3S$ $[M+H]^+$: 298.0719, found 298.0701.

Optimization of the conditions for the annulation of 1a and 2a [a] (Table S1)

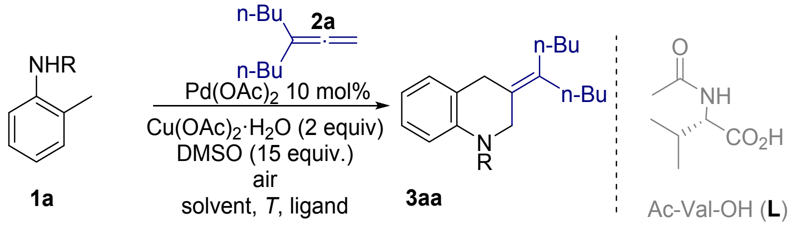

Entry	R	Solvent	Base	Temp	Ligand L[b]	Yield[c]
1	Tf	Toluene	Cs_2CO_3 (1.5 equiv)	105 ºC	-	<5%
2	Tf	Toluene	Cs_2CO_3 (1.5 equiv)	105 ºC	Boc-Val-OH	25%
3	Tf	Toluene	Cs_2CO_3 (1.5 equiv)	105 ºC	Ac-Gly-OH	42%
4	Tf	Toluene	Cs_2CO_3 (1.5 equiv)	105 ºC	Ac-Ala-OH	55%
5	Tf	Toluene	Cs_2CO_3 (1.5 equiv)	105 ºC	Ac-Leu-OH	55%
6	Tf	Toluene	Cs_2CO_3 (1.5 equiv)	105 ºC	Formyl-Val-OH	37%
7	Tf	Toluene	Cs_2CO_3 (1.5 equiv)	105 ºC	Pro-Val-OH	52%
8	Tf	Toluene	Cs_2CO_3 (1.5 equiv)	105 ºC	Ac-Val-OH	60%
9	Ms	Toluene	Cs_2CO_3 (1.5 equiv)	105 ºC	Ac-Val-OH	39%
10	Ns	Toluene	Cs_2CO_3 (1.5 equiv)	105 ºC	Ac-Val-OH	33%
11	Tf	Toluene	K_2CO_3 (1.5 equiv)	105 ºC	Ac-Val-OH	53%
12	Tf	Toluene	Na_2CO_3 (1.5 equiv)	105 ºC	Ac-Val-OH	31%
13	Tf	Toluene	K_3PO_4 (1.5 equiv)	105 ºC	Ac-Val-OH	58%
14	Tf	*p*-Xylene	Cs_2CO_3 (1.5 equiv)	105 ºC	Ac-Val-OH	49%
15[e]	Tf	THF	Cs_2CO_3 (1.5 equiv)	105 ºC	Ac-Val-OH	53%[d]
16[e]	Tf	2-M THF	Cs_2CO_3 (1.5 equiv)	105 ºC	Ac-Val-OH	58%[d]
17	Tf	2-M THF	Cs_2CO_3 (1.5 equiv)	85 ºC	Ac-Val-OH	54%[d]
18[f]	Tf	2-M THF	Cs_2CO_3 (1 equiv)	85 ºC	Ac-Val-OH	61%[d]
19[f,g]	Tf	2-M THF	Cs_2CO_3 (1 equiv)	85 ºC	Ac-Val-OH	56%
20[f,h]	Tf	2-M THF	Cs_2CO_3 (1 equiv)	85 ºC	Ac-Val-OH	55%
21[f,i]	Tf	2-M THF	Cs_2CO_3 (1 equiv)	85 ºC	Ac-Val-OH	56%
22[f,j]	Tf	2-M THF	Cs_2CO_3 (1 equiv)	85 ºC	Ac-Val-OH	71%[d]
23[f,k]	Tf	2-M THF	Cs_2CO_3 (1 equiv)	85 ºC	Ac-Val-OH	No reaction
24[l]	Tf	2-M THF	Cs_2CO_3 (1 equiv)	85 ºC	Ac-Val-OH	7%

[a] Conditions: 0.333 mmol **1a**, 0.167 mmol of allene **2a**, 2 mL of solvent, under air, 16h. [b] 40% of ligand. [c] Yields calculated by using an internal standard. [d] Isolated yield based on **2a**. [e] Reaction performed in sealed tube [f] 1 equiv. of $Cu(OAc)_2 \cdot H_2O$ [g] Without DMSO. [h] 0.167 mmol **1a**, 0.333 mmol of allene **2a**. [i] 0.167 mmol 1a, 0.167 mmol of allene **2a** [j] Slow addition over 1h of 0.167 mmol of allene **2a** in 1.5 mL 2-M THF to the reaction instead of mixing it before heating [k] Without catalyst. [l] 1 Without $Cu(OAc)_2 \cdot H_2O$.

Note: Yields are lower in absence of DMSO (see SI for details). DMSO might help to solubilize the copper salts or/and stabilize metal complex intermediates.

General procedure for the Pd-catalyzed annulation of *o*-methylanilines with allenes, exemplified for 3aa:

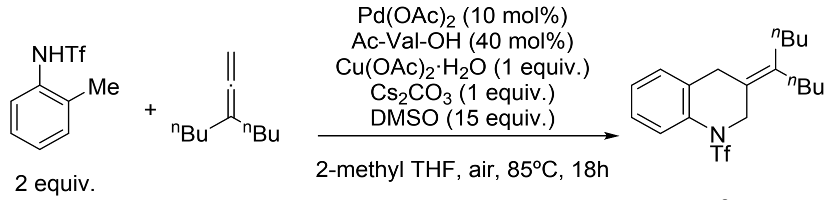

3aa

To a solution of $Pd(OAc)_2$ (3.7 mg, 10 mol%), Ac-Val-OH (10.6 mg, 40 mol%), $Cu(OAc)_2$•H_2O (33.3 mg, 1 equiv.), Cs_2CO_3 (54.4 mg, 1 equiv.) and **1a** (79.9 mg, 0.333 mmol, 2 equiv.) in 2-methyl THF (1 mL) and DMSO (0.178 µL, 15 equiv.), under air atmosphere, heated at 85 ºC, in a Schlenk tube sealed with a rubber septum was slowly added the allene **2a** (25.4 mg, 0.167 mmol) with a syringe pump during 1h . The reaction was stirred at 85 ºC using a Thermowatch-controlled heating block during 18 h and then cooled to room temperature. Evaporation and column chromatography on silica gel (hexanes:diethylether; 99:1) afforded **3-(nonan-5-ylidene)-1-((trifluoromethyl)sulfonyl)-1,2,3,4-tetrahydroquinoline** (**3aa**) as a pale orange oil (46.4 mg, 71% yield). ^{1}H NMR (300 MHz, $CDCl_3$) δ 7.61 – 7.49 (m, 1H), 7.31 – 7.21 (m, 3H), 4.46 (s, 2H), 3.58 (s, 2H), 2.18 – 2.02 (m, 4H), 1.46 – 1.26 (m, 8H), 1.04 – 0.86 (m, 6H). **^{19}F NMR** (282 MHz, $CDCl_3$) δ -75.61. **^{13}C NMR** (75 MHz, $CDCl_3$) δ 137.0 (C), 136.3 (C), 133.1 (C), 129.0 (CH), 127.4 (CH), 127.0 (CH), 124.7 (CH), 123.3 (C), 120.2 (d, J = 324.9 Hz, C), 50.1 (CH_2), 32.03 (CH_2), 31.96 (CH_2), 30.9 (CH_2), 30.8 (CH_2), 30.1 (CH_2), 23.1 (CH_2), 23.0 (CH_2), 14.2 (CH_3), 14.1 (CH_3). **HRMS** [APCI]: *m/z* calculated for $C_{19}H_{27}F_3NO_2S$ $[M+H]^+$: 298.0719, found 298.0701.

3-Cyclohexylidene-1-((trifluoromethyl)sulfonyl)-1,2,3,4-tetrahydroquinoline (3ab)

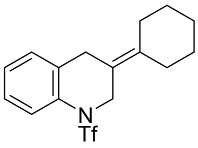

3ab

(37.8 mg, 61% yield), obtained as a transparent oil. **^{1}H NMR** (300 MHz, $CDCl_3$) δ 7.49 – 7.38 (m, 1H), 7.21 – 7.11 (m, 3H), 4.37 (s, 2H), 3.51 (s, 2H), 2.21 – 2.05 (m, 4H), 1.56 – 1.43 (m, 6H). **^{19}F NMR** (282 MHz, $CDCl_3$) δ -75.68. **^{13}C NMR** (75 MHz, $CDCl_3$) δ 136.3 (C), 135.8 (C), 132.9 (C), 129.1 (CH), 127.3 (CH), 126.9 (CH), 124.6 (CH), 120.1 (d, J = 324.6 Hz, C), 119.5 (C), 49.9 (CH_2), 30.7 (CH_2), 30.5 (CH_2), 30.3 (CH_2), 27.7 (CH_2), 27.6 (CH_2), 26.7 (CH_2). **HRMS** [APCI]: *m/z* calculated for $C_{16}H_{19}F_3NO_2S$ $[M+H]^+$: 346.1083, found 346.1076.

(*E*)-3-Butylidene-2-propyl-1-((trifluoromethyl)sulfonyl)-1,2,3,4-tetrahydroquinoline (3ac)

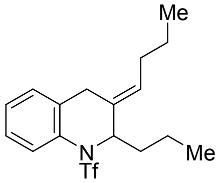

3ac

(36.8 mg, 61% yield), obtained as a transparent oil. **^{1}H NMR** (500 MHz, $CDCl_3$) δ 7.51 – 7.43 (m, 1H), 7.20 – 7.07 (m, 3H), 5.37 (t, J = 7.3 Hz, 1H), 4.64 – 4.55 (m, 1H), 3.50 (dd, J = 64.4, 20.9 Hz, 2H), 2.09 – 1.90 (m, 2H), 1.42 – 1.19 (m, 6H), 0.87 (t, J = 7.4 Hz, 3H), 0.78 (t, J = 7.0 Hz, 3H). **^{19}F NMR** (282 MHz, $CDCl_3$) δ -74.61. **^{13}C NMR** (126 MHz, $CDCl_3$) δ 133.3 (C), 131.9 (C), 131. 1 (C), 129.2 (CH), 127.7 (CH), 127.3 (CH), 127.0 (CH), 126.2 (CH), 120.2 (q, J = 325.6 Hz, C), 64.2 (CH_2), 36.1 (CH_2), 29.5 (CH_2), 28.1 (CH_2), 22.3 (CH_2), 19.2 (CH_2), 13.9 (CH_3), 13.5 (CH_3). **HRMS** [APCI]: *m/z* calculated for $C_{17}H_{22}F_3NO_2S$ $[M]^+$: 361.1323, found 361.1322.

Assignment of structure of **3ac** was based on the observed nOe between the H_a (5.37 ppm, 100%) with H_b (4.64 – 4.55 ppm, 4.7%) as shown in the Figure S1

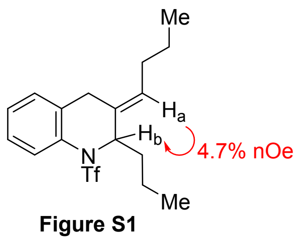

Figure S1

(*E*)-3-Benzylidene-2-methyl-1-((trifluoromethyl)sulfonyl)-1,2,3,4-tetrahydroquinoline (3ad)

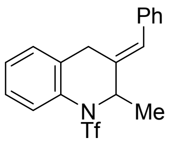

3ad

(40.5 mg, 66% yield), obtained as a white solid. **^{1}H NMR** (500 MHz, $CDCl_3$) δ 7.47 (d, *J* = 7.6 Hz, 1H), 7.32 (t, *J* = 7.7 Hz, 2H), 7.28 – 7.09 (m, 6H), 6.44 (s, 1H), 5.03 (q, *J* = 6.9 Hz, 1H), 3.81 (s, 2H), 1.28 (d, *J* = 6.9 Hz, 3H). **^{19}F NMR** (282 MHz, $CDCl_3$) δ -74.87. **^{13}C NMR** (126 MHz, $CDCl_3$) δ 136.3 (C), 135.8 (C), 133.0 (C), 130.4 (C), 129.1 (CH), 129.0 (CH), 128.6 (CH), 127.43 (CH), 127.42 (CH), 127.37 (CH), 126.3 (CH), 126.1 (CH), 120.2 (q, *J* = 324.9 Hz, C), 60.4 (CH), 29.7 (CH_2), 21.3 (CH_3). **HRMS** [APCI]: *m/z* calculated for $C_{18}H_{17}F_3NO_2S$ $[M+H]^+$: 368.0927, found 368.0928.

Assignment of structure of **3ad** based on the observed nOe between the H_a (5.03 ppm, 100%) with H_b (6.44 ppm, 6.0%) as shown in the Figure S2.

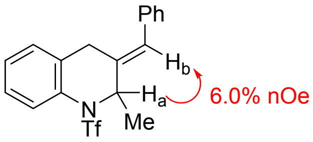

Figure S2

3-Benzylidene-2-phenethyl-1-((trifluoromethyl)sulfonyl)-1,2,3,4-tetrahydroquinoline (3ae and 3ae')

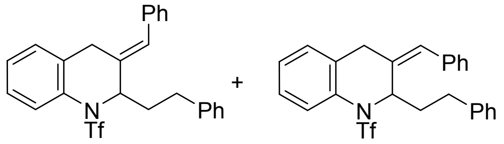

3ae **3ae'**

(1.8:1 E/Z ratio of **3ae** and **3ae'**, inseparable mixture (58.1 mg, 76% yield), obtained as a yellow solid). **^{1}H NMR** (500 MHz, $CDCl_3$) δ 7.59 (d, *J* = 7.3 Hz, 0.54H), 7.49 (d, *J* = 7.8 Hz, 0.31H), 7.43 – 6.98 (m, 13H), 6.51 (s, 0.65H), 5.88 (s, 0.36H), 5.64 (t, *J* = 7.1 Hz, 0.39H), 4.92 (t, *J* = 7.7 Hz, 0.66H), 3.94 (d, *J* = 20.9 Hz, 0.68H), 3.82 (dd, *J* = 21.0, 2.2 Hz, 0.70H), 3.62 (d, *J* = 20.4 Hz, 0.41H), 3.37 (d, *J* = 20.3 Hz, 0.40H), 2.91 – 2.69 (m, 2 H), 2.57 (q, *J* = 7.4 Hz, 0.83H), 1.85 (q, *J* = 7.8 Hz, 1.40H). **^{19}F NMR** (282 MHz, $CDCl_3$) δ -74.32. **^{13}C NMR** (126 MHz, $CDCl_3$) δ 141.4 (C), 140.7 (C), 137.6 (C), 136.2 (C), 134.0 (C), 133.8 (C), 132.9 (C), 131.2 (C), 130.7 (C), 130.4 (C), 129.9 (CH), 129.3 (CH), 129.1 (CH), 129.0 (CH), 128.64 (CH), 128.62 (CH), 128.57 (CH), 128.5 (CH), 127.8 (CH), 127.6 (CH), 127.5 (CH), 127.4 (CH), 127.2 (CH), 127.1 (CH), 126.9 (CH), 126.31 (CH), 126.26 (CH), 126.13 (CH), 126.10 (CH), 120.2 (q, *J* = 325.7 Hz, C), 66.6 (CH), 64.6 (CH), 35.5 (CH_2), 35.2 (CH_2), 32.4 (CH_2), 29.7 (CH_2), 29.6 (CH_2), 28.5 (CH_2). **HRMS** [APCI]: *m/z* calculated for $C_{25}H_{23}F_3NO_2S$ $[M+H]^+$: 458.1396, found 458.1395.

Assignment of structure of **3ae** based on the observed nOe between the H_a (6.51 ppm, 100%) with H_b (4.92 ppm, 8.3%) as shown in the Figure S3.

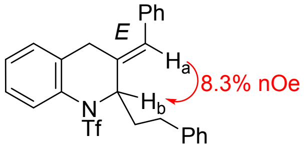

Figure S3

3-(Cyclohexylmethylene)-1-((trifluoromethyl)sulfonyl)-1,2,3,4-tetrahydroquinoline (3af and 3af')

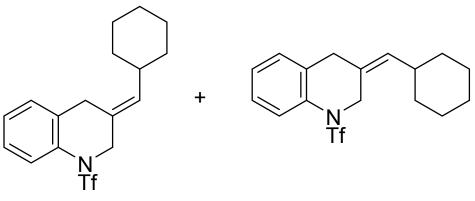

3af **3af'**

(1.1:1 E/Z ratio of **3af** and **3af'**, inseparable mixture (41.2 mg, 68% yield), obtained as a transparent oil). **^{1}H NMR** (500 MHz, $CDCl_3$) δ 7.51 – 7.40 (m, 1H), 7.20 – 7.07 (m, 3H), 5.28 (d, *J* = 9.5 Hz, 0.51), 5.19 (d, *J* = 9.6 Hz, 0.48H), 4.40 (s, 1.01H), 4.22 (s, 1.11H), 3.55 (s, 1.24H), 3.38 (s, 1.05H), 2.25 – 2.12 (m, 0.64H), 2.06 – 1.94 (m, 0.59H), 1.71 – 1.42 (m, 7H), 1.31 – 0.91 (m, 7H). **^{19}F NMR** (282 MHz, $CDCl_3$) δ -74.80, -75.77. **^{13}C NMR** (126 MHz, $CDCl_3$) δ 136.2 (C), 135.7 (C), 133.9 (CH), 133.8 (C), 132.2 (CH), 131.3 (C), 129.4 (CH), 128.5 (C), 128.2 (CH), 127.6 (CH), 127.3 (CH), 127.2 (CH), 127.0 (CH), 126.8 (C), 124.9 (CH), 124.8 (CH), 120.2 (q, *J* = 324.2 Hz, C), 120.1 (q, *J* = 325.2 Hz, C), 54.6 (CH_2), 49.5 (CH_2), 37.2 (CH_2), 36.8 (CH), 35.7 (CH), 32.9 (CH_2), 32.5 (CH_2), 29.6 (CH_2), 26.1 (CH_2), 26.0 (CH_2), 25.94 (CH_2), 25.86 (CH_2). **HRMS** [APCI]: *m/z* calculated for $C_{17}H_{20}F_3NO_2S$ $[M]^+$: 359.1161, found 359.1164.

Assignment of stereochemistry of **3af** and **3af'** was based on the observed nOe between the H_a (5.28 ppm, 100%) with H_b (4.22 ppm,

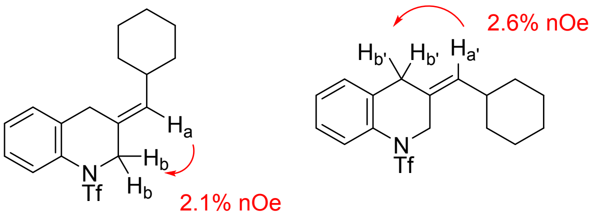

Figure S4

2.1%) and between the $H_{a'}$ (5.19 ppm, 100%) with $H_{b'}$ (3.38 ppm, 2.6%) as shown in the Figure S4.

3-(4-(trifluoromethyl)benzylidene)-1-((trifluoromethyl)sulfonyl)-1,2,3,4-tetrahydroquinoline (3ag and 3ag')

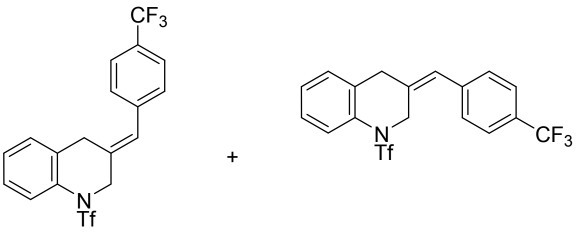

3ag **3ag'**

(6.5:1 E/Z ratio of **3ag** and **3ag'**, inseparable mixture (25.5 mg, 36% yield), obtained as a white solid). **^{1}H NMR** (500 MHz, $CDCl_3$) δ 7.53 (d, J = 7.8 Hz, 2H), 7.49 – 7.41 (m, 1H), 7.26 (d, J = 7.9 Hz, 2H), 7.24 – 7.13 (m, 3H), 7.06 – 7.00 (m, 1H), 6.49 (s, 0.13H), 6.18 (s, 0.85H), 4.62 (s, 0.30H), 4.19 (s, 1.85H), 3.63 (s, 0.31H), 3.54 (s, 1.96H). **^{19}F NMR** (282 MHz, $CDCl_3$) δ -62.25, -62.37, -74.74, -74.98. **^{13}C NMR** (126 MHz, $CDCl_3$) δ 140.7 (C), 139.6 (C), 136.8 (C), 136.1 (C), 135.4 (C), 133.1 (C), 132.2 (C), 129.8 (C), 129.74 (C), 129.67 (CH), 129.5 (C), 128.8 (CH), 128.14 (CH), 128.12 (CH), 128.07 (CH), 127.8 (CH), 127.7 (CH), 126.9 (CH), 125.9 (q, J = 3.8 Hz, CH), 125.7 (q, J = 3.8 Hz, CH), 125.1 (CH), 125.0 (CH), 124.33 (CH), 124.27 (q, J = 271.9 Hz), 123.0 (CH), 120.3 (q, J = 324.9 Hz), 50.4 (CH_2), 49.6 (CH_2), 41.0 (CH_2), 36.7 (CH_2). **HRMS** [APCI]: *m/z* calculated for $C_{18}H_{13}F_6NO_2S$ $[M]^+$: 421.0571, found 421.0574.

Assignment of stereochemistry of **3ag** and **3ag'** was based on the observed nOe between the H_a (6.18 ppm, 100%) with H_b (4.19 ppm, 1.9%) and between the $H_{a'}$ (6.49 ppm, 100%) with $H_{b'}$ (3.63 ppm, 4.2%) as shown in the Figure S5.

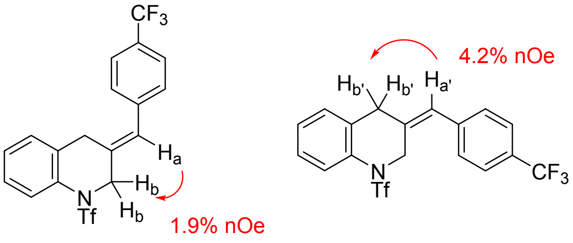

Figure S5

(E)-3-Benzylidene-2,2-dimethyl-1-((trifluoromethyl)sulfonyl)-1,2,3,4-tetrahydroquinoline (3ah)

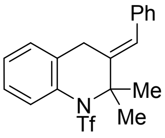

3ah

(46.6 mg, 73% yield), obtained as a white solid. **^{1}H NMR** (300 MHz, $CDCl_3$) δ 7.41 – 7.25 (m, 3H), 7.27 – 7.11 (m, 5H), 7.08 – 7.00 (m, 1H), 6.40 (s, 1H), 3.68 (s, 2H), 1.62 (s, 6H). **^{19}F NMR** (282 MHz, $CDCl_3$) δ -75.27. **^{13}C NMR** (75 MHz, $CDCl_3$) δ 143.1 (C), 137.0 (C), 136.8 (C), 134.4 (C), 129.0 (CH), 128.6 (CH), 128.2 (CH), 128.0 (CH), 127.6 (CH), 127.2 (CH), 127.1 (CH), 123.5 (CH), 120.0 (d, *J* = 324.8 Hz, C), 68.6 (C), 31.4 (CH_2), 29.7 (CH_3). **HRMS** [APCI]: *m/z* calculated for $C_{19}H_{19}F_3NO_2S$ $[M+H]^+$: 382.1083, found 382.1083.

Assignment of stereochemistry of **3ah** was based on the observed nOe between the H_a (6.40 ppm, 100%) with the H_b (1.62 ppm, 6.2%) as shown in the Figure S6.

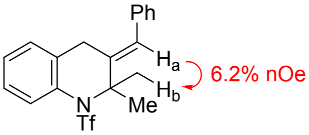

Figure S6

(E)-3-heptylidene-2,2-dimethyl-1-((trifluoromethyl)sulfonyl)-1,2,3,4-tetrahydroquinoline (3ai) and 2-hexyl-3-(propan-2-ylidene)-1-((trifluoromethyl)sulfonyl)-1,2,3,4-tetrahydroquinoline (3ai')

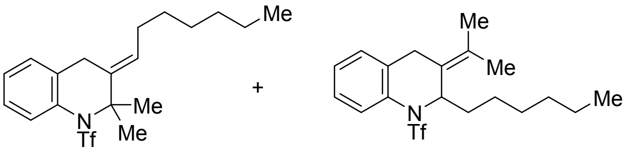

3ai **3ai'**

(2.8:1 ratio of **3ai** and **3ai'**, inseparable mixture (27.5 mg, 42% yield), obtained as a transparent oil). **^{1}H NMR** (500 MHz, $CDCl_3$) δ 7.33 (d, J = 7.1 Hz, 0.58H), 7.23 – 7.08 (m, 3.25H), 5.33 (t, J = 7.1 Hz, 0.74H), 5.03 (t, J = 7.2 Hz, 0.26H), 3.50 – 3.43 (m, 2H), 2.00 (q, J = 7.3 Hz, 1.58H), 1.70 (s, 0.88H), 1.66 (s, 0.88H), 1.48 (s, 4.43H), 1.28 – 1.11 (m, 8.67H), 0.82 (t, J = 6.6 Hz, 2.25H), 0.77 (t, J = 7.0 Hz, 1.02H). **^{19}F NMR** (282 MHz, $CDCl_3$) δ -74.48, -74.57. **^{13}C NMR** (126 MHz, $CDCl_3$) δ 139.2 (C), 137.1 (C), 134.5 (C), 133.0 (C), 132.4 (C), 129.0 (CH), 128.2 (CH), 128.0 (CH), 127.7 (CH), 127.4 (CH), 127.0 (C), 126.9 (CH), 126.8 (CH), 126.3 (CH), 126.1 (C), 123.9 (CH), 120.2

(d, J = 325.6 Hz, C), 120.0 (d, J = 324.9 Hz, C), 67.9 (C), 59.3 (CH), 32.8 (CH_2), 31.8 (CH_2), 31.7 (CH_2), 30.0 (CH_2), 29.8 (CH_2), 29.33 (CH_3), 29.27 (CH_2), 29.1 (CH_2), 28.6 (CH_2), 27.8 (CH_2), 25.7 (CH_2), 22.7 (CH_2), 22.6 (CH_2), 20.3 (CH_3), 20.2 (CH_3), 14.2 (CH_3), 14.1 (CH_3). **HRMS** [APCI]: *m/z* calculated for $C_{19}H_{26}F_3NO_2S$ $[M]^+$: 389.1636, found 389.1633.

Assignment of stereochemistry of **3ai** was based on a NOESY experiment. Qualitative nOe was observed between the H_a (5.33 ppm) with the H_b (1.48 ppm) as shown in the Figure S7.

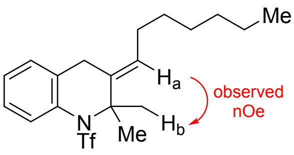

Figure S7

(*E*)-3-Benzylidene-2-butyl-2-methyl-1-((trifluoromethyl)sulfonyl)-1,2,3,4-tetrahydroquinoline (3aj)

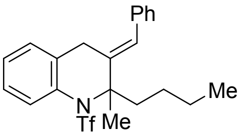

3aj

(40.2 mg, 57% yield), obtained as a white solid. **^{1}H NMR** (300 MHz, $CDCl_3$) δ 7.42 – 6.95 (m, 9H), 6.40 (s, 1H), 3.70 (s, 2H), 1.91 (s, 3H), 1.43 – 0.81 (m, 6H), 0.68 (t, *J* = 7.3 Hz, 3H). **^{19}F NMR** (282 MHz, $CDCl_3$) δ -75.15. **^{13}C NMR** (75 MHz, $CDCl_3$) δ 143.1 (C), 137.0 (C), 136.7 (C), 134.3 (C), 129.0 (CH), 128.5 (CH), 128.2 (CH), 127.9 (CH), 127.7 (CH), 127.2 (CH), 127.0 (CH), 124.3 (CH), 120.0 (d, *J* = 324.6 Hz, C), 71.2 (C), 42.9 (CH_2), 31.5 (CH_2), 25.7 (CH_2), 23.8 (CH_3), 22.6 (CH_2), 13.9 (CH_3). **HRMS** [APCI]: *m/z* calculated for $C_{22}H_{25}F_3NO_2S$ $[M+H]^+$: 424.1553, found 424.1558.

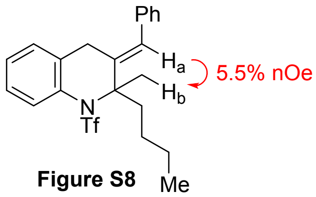

Figure S8

Assignment of stereochemistry of **3aj** was based on the observed nOe between the H_a (6.40 ppm, 100%) with the H_b (1.91 ppm, 5.5%) as shown in the Figure S8.

5-Methoxy-3-(nonan-5-ylidene)-1-((trifluoromethyl)sulfonyl)-1,2,3,4-tetrahydroquinoline (3ba)

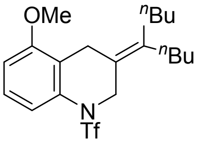

3ba

(48.6 mg, 69% yield), obtained as a yellow oil. **^{1}H NMR** (300 MHz, $CDCl_3$) δ 7.21 – 7.14 (m, 2H), 6.84 – 6.72 (m, 1H), 4.40 (s, 2H), 3.87 (s, 3H), 3.49 (s, 2H), 2.19 – 2.04 (m, 4H), 1.46 – 1.25 (m, 8H), 1.01 – 0.85 (m, 6H). **^{19}F NMR (**282 MHz, $CDCl_3$) δ -75.23. **^{13}C NMR** (75 MHz, $CDCl_3$) δ 157.0 (C), 137.4 (C), 136.9 (C), 126.8 (CH), 122.3 (C), 121.3 (C), 120.1 (d, *J* = 325.4 Hz, C), 116.7 (CH), 108.4 (CH), 55.7 (CH_3), 49.7 (CH_2), 32.0 (CH_2), 31.9 (CH_2), 31.1 (CH_2), 30.0 (CH_2), 24.5 (CH_2), 23.1 (CH_2), 23.0 (CH_2), 14.2 (CH_3), 14.1 (CH_3). **HRMS** [APCI]: *m/z* calculated for $C_{20}H_{29}F_3NO_3S$ $[M+H]^+$: 390.1709, found 390.1704.

5-Chloro-3-(nonan-5-ylidene)-1-((trifluoromethyl)sulfonyl)-1,2,3,4-tetrahydroquinoline (3ca)

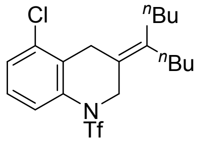

3ca

(35.7 mg, 50% yield), obtained as a white solid. **^{1}H NMR** (500 MHz, $CDCl_3$) δ 7.40 (d, *J* = 8.2 Hz, 1H), 7.25 (d, *J* = 8.0 Hz, 1H), 7.10 (t, *J* = 8.2 Hz, 1H), 4.34 (brs, 2H), 3.55 (s, 2H), 2.04 (dt, *J* = 22.1, 7.5 Hz, 4H), 1.40 – 1.15 (m, 8H), 0.95 – 0.81 (m, 6H). **^{19}F NMR** (282 MHz, $CDCl_3$) δ -75.25. **^{13}C NMR** (126 MHz, $CDCl_3$) δ 138.3 (C), 137.5 (C), 134.0 (C), 130.9 (C), 128.0 (CH), 127.2 (CH), 123.3 (CH), 121.7 (C), 120.1 (q, *J* = 324.9 Hz, C), 49.7 (CH_2), 32.00 (CH_2), 31.98 (CH_2), 31.0 (CH_2), 30.1 (CH_2), 28.8 (CH_2), 23.1 (CH_2), 23.0 (CH_2), 14.12 (CH_3), 14.09 (CH_3). **HRMS** [APCI]: *m/z* calculated for $C_{19}H_{24}ClF_3NO_2S$ $[M-H]^+$: 422.1163, found 422.1163.

6-Chloro-3-(nonan-5-ylidene)-1-((trifluoromethyl)sulfonyl)-1,2,3,4-tetrahydroquinoline (3da)

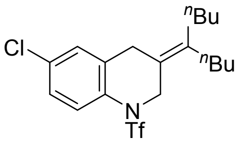

3da

(39.7 mg, 56% yield), obtained as a yellow oil. **^{1}H NMR** (300 MHz, $CDCl_3$) δ 7.39 (d, *J* = 8.4 Hz, 1H), 7.19 – 7.09 (m, 2H), 4.34 (s, 2H), 3.45 (s, 2H), 2.10 – 1.90 (m, 4H), 1.34 – 1.21 (m, 8H), 0.90 – 0.76 (m, 6H). **^{19}F NMR** (282 MHz, $CDCl_3$) δ -75.49. **^{13}C NMR** (75 MHz, $CDCl_3$) δ 137.7 (C), 134.81 (C), 134.78 (C), 133.0 (C), 128.9 (CH), 127.2 (CH), 126.0 (CH), 122.3 (C), 120.1 (d,

J = 325.0 Hz, C), 50.0 (CH_2), 32.1 (CH_2), 32.0 (CH_2), 30.8 (CH_2), 30.1 (CH_2), 23.1 (CH_2), 23.0 (CH_2), 14.14 (CH_3), 14.08 (CH_3). **HRMS** [APCI]: *m/z* calculated for $C_{19}H_{24}ClF_3NO_2S$ $[M-H]^+$: 422.1163, found 422.1161.

6-Bromo-3-(nonan-5-ylidene)-1-((trifluoromethyl)sulfonyl)-1,2,3,4-tetrahydroquinoline (3ea)

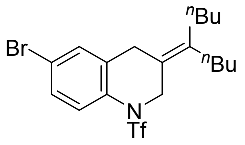

3ea

(49.1 mg, 63% yield), obtained as a yellow oil. **^{1}H NMR** (300 MHz, $CDCl_3$) δ 7.45 – 7.29 (m, 3H), 4.41 (s, 2H), 3.53 (s, 2H), 2.16 – 1.95 (m, 4H), 1.46 – 1.23 (m, 8H), 1.02 – 0.82 (m, 6H). **^{19}F NMR** (282 MHz, $CDCl_3$) δ -75.49. **^{13}C NMR** (75 MHz, $CDCl_3$) δ 137.8 (C), 135.3 (C), 135.1 (C), 131.8 (CH), 130.1 (CH), 126.3 (CH), 122.3 (C), 120.9 (C), 120.1 (d, J = 324.9 Hz, C), 50.0 (CH_2), 32.04 (CH_2), 31.98 (CH_2), 30.8 (CH_2), 30.7 (CH_2), 30.1 (CH_2), 23.1 (CH_2), 23.0 (CH_2), 14.14 (CH_3), 14.08 (CH_3). **HRMS** [APCI]: *m/z* calculated for $C_{19}H_{26}BrF_3NO_2S$ $[M+H]^+$: 468.0814, found 468.0813.

3-(Nonan-5-ylidene)-6-phenyl-1-((trifluoromethyl)sulfonyl)-1,2,3,4-tetrahydroquinoline (3fa)

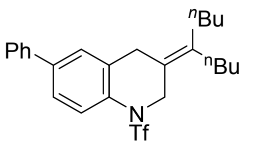

3fa

(56.5 mg, 73% yield), obtained as a white solid. **^{1}H NMR** (300 MHz, $CDCl_3$) δ 7.66 – 7.54 (m, 3H), 7.51 – 7.42 (m, 4H), 7.42 – 7.34 (m, 1H), 4.49 (s, 2H), 3.64 (s, 2H), 2.20 – 2.03 (m, 4H), 1.46 – 1.31 (m, 8H), 1.04 – 0.89 (m, 6H). **^{19}F NMR** (282 MHz, $CDCl_3$) δ -75.46. **^{13}C NMR** (75 MHz, $CDCl_3$) δ 140.4 (C), 140.2 (C), 137.2 (C), 133.3 (C), 129.0 (CH), 127.8 (CH), 127.6 (CH), 127.2 (CH), 125.7 (CH), 125.0 (CH), 123.1 (C), 120.2 (d, J = 325.0 Hz, C), 50.2 (CH_2), 32.1 (CH_2), 32.0 (CH_2), 31.1 (CH_2), 30.8 (CH_2), 30.2 (CH_2), 23.1 (CH_2), 23.0 (CH_2), 14.2 (CH_3), 14.1 (CH_3). **HRMS** [APCI]: *m/z* calculated for $C_{25}H_{29}F_3NO_2S$ $[M-H]^+$: 464.1866, found 464.1859. The structure of this compound was confirmed by X-Ray diffraction analysis (CCDC: 2026002).

6-Methoxy-3-(nonan-5-ylidene)-1-((trifluoromethyl)sulfonyl)-1,2,3,4-tetrahydroquinoline (3ga)

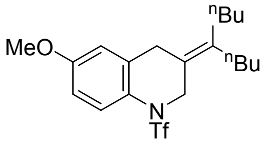

3ga

(47.0 mg, 67% yield), obtained as a white solid. **^{1}H NMR** (300 MHz, $CDCl_3$) δ 7.43 (d, J = 8.6 Hz, 1H), 6.80 – 6.71 (m, 2H), 4.41 (brs, 2H), 3.80 (s, 3H), 3.52 (s, 2H), 2.14 – 1.97 (m, 4H), 1.42 – 1.28 (m, 8H), 1.01 – 0.84 (m, 6H). **^{19}F NMR** (282 MHz, $CDCl_3$) δ -75.46. **^{13}C NMR** (75 MHz, $CDCl_3$) δ 158.7 (C), 136.9 (C), 134.7 (C), 129.0 (C), 126.0 (CH), 123.2 (C), 120.2 (d, J = 325.1 Hz, C), 113.8 (CH), 112.4 (CH), 55.6 (CH_3), 50.3 (CH_2), 32.1 (CH_2), 32.0 (CH_2), 31.2 (CH_2), 30.7 (CH_2), 30.1 (CH_2), 23.1 (CH_2), 23.0 (CH_2), 14.2 (CH_3), 14.10 (CH_3). **HRMS** [APCI]: *m/z* calculated for $C_{20}H_{29}F_3NO_3S$ $[M+H]^+$: 420.1815, found 420.1803.

7-Chloro-3-(nonan-5-ylidene)-1-((trifluoromethyl)sulfonyl)-1,2,3,4-tetrahydroquinoline (3ha)

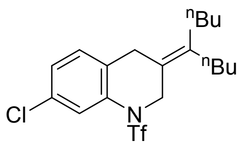

3ha

(40.6 mg, 57% yield), obtained as a yellow solid. **^{1}H NMR** (300 MHz, $CDCl_3$) δ 7.55 (d, J = 2.0 Hz, 1H), 7.26 – 7.10 (m, 2H), 4.41 (brs, 2H), 3.52 (s, 2H), 2.16 – 1.97 (m, 4H), 1.44 – 1.21 (m, 8H), 1.02 – 0.81 (m, 6H). **^{19}F NMR** (282 MHz, $CDCl_3$) δ -75.51. **^{13}C NMR** (75 MHz, $CDCl_3$) δ 137.7 (C), 137.1 (C), 132.3 (C), 131.4 (C), 129.9 (CH), 127.6 (CH), 124.8 (CH), 122.5 (C), 120.1 (d, J = 324.8 Hz, C), 50.0 (CH_2), 32.05 (CH_2), 31.98 (CH_2), 30.8 (CH_2), 30.5 (CH_2), 30.1 (CH_2), 23.1 (CH_2), 23.0 (CH_2), 14.2 (CH_3), 14.1 (CH_3). **HRMS** [APCI]: *m/z* calculated for $C_{19}H_{24}ClF_3NO_2S$ $[M-H]^+$: 422.1163, found 422.1160.

Methyl 3-(nonan-5-ylidene)-1-((trifluoromethyl)sulfonyl)-1,2,3,4-tetrahydroquinoline-7-carboxylate (3ia)

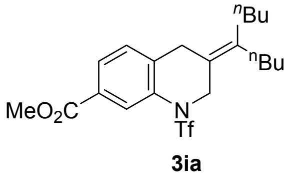

3ia

(37.2 mg, 50% yield), obtained as a white solid. **^{1}H NMR** (500 MHz, $CDCl_3$) δ 8.19 (d, J = 1.6 Hz, 1H), 7.91 (dd, J = 8.0, 1.6 Hz, 1H), 7.29 (d, J = 8.1 Hz, 1H), 4.44 (s, 2H), 3.92 (s, 3H), 3.60 (s, 2H), 2.13 – 2.01 (m, 4H), 1.41 – 1.26 (m, 8H), 0.99 – 0.88 (m, 6H). **^{19}F NMR** (282 MHz, $CDCl_3$) δ -75.52. **^{13}C NMR** (126 MHz, $CDCl_3$) δ 166.2 (C), 138.3 (C), 137.8 (C), 136.4

(C), 129.4 (C), 129.1 (CH), 128.5 (CH), 125.9 (CH), 122.4 (C), 120.1 (q, *J* = 324.6 Hz, C), 52.5 (CH_3), 50.0 (CH_2), 32.1 (CH_2), 32.0 (CH_2), 31.2 (CH_2), 30.8 (CH_2), 30.1 (CH_2), 23.1 (CH_2), 23.0 (CH_2), 14.14 (CH_3), 14.08 (CH_3). **HRMS** [APCI]: *m/z* calculated for $C_{21}H_{29}F_3NO_4S$ $[M+H]^+$: 448.1764, found 448.1766.

7-Methoxy-3-(nonan-5-ylidene)-1-((trifluoromethyl)sulfonyl)-1,2,3,4-tetrahydroquinoline (3ja)

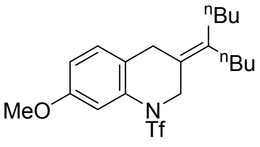

3ja

(44.7 mg, 64% yield), obtained as a white solid. **^{1}H NMR** (500 MHz, $CDCl_3$) δ 7.10 (dd, *J* = 5.6, 3.0 Hz, 2H), 6.80 (dd, *J* = 8.5, 2.6 Hz, 1H), 4.42 (brs, 2H), 3.80 (s, 3H), 3.49 (s, 2H), 2.16 – 1.99 (m, 4H), 1.49 – 1.18 (m, 8H), 1.05 – 0.77 (m, 6H). **^{19}F NMR** (282 MHz, $CDCl_3$) δ -75.52. **^{13}C NMR** (126 MHz, $CDCl_3$) δ 158.4 (C), 136.86 (C), 136.88 (C), 129.5 (CH), 124.8 (C), 123.4 (C), 120.2 (q, *J* = 325.0 Hz, C), 114.0 (CH), 109.9 (CH), 55.6 (CH_3), 50.2 (CH_2), 31.97 (CH_2), 32.01 (CH_2), 30.9 (CH_2), 30.1 (CH_2), 23.1 (CH_2), 23.0 (CH_2), 14.2 (CH_3), 14.1 (CH_3). **HRMS** [APCI]: *m/z* calculated for $C_{20}H_{29}F_3NO_3S$ $[M+H]^+$: 420.1815, found 420.1812.

7-Fluoro-3-(nonan-5-ylidene)-1-((trifluoromethyl)sulfonyl)-1,2,3,4-tetrahydroquinoline (3ka)

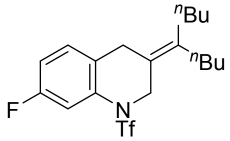

3ka

(47.0 mg, 69% yield), obtained as a yellow oil. **^{1}H NMR** (300 MHz, $CDCl_3$) δ 7.30 (dd, *J* = 9.8, 2.6 Hz, 1H), 7.21 – 7.13 (m, 1H), 6.95 (td, *J* = 8.3, 2.6 Hz, 1H), 4.42 (s, 2H), 3.52 (s, 2H), 2.16 – 1.98 (m, 4H), 1.46 – 1.28 (m, 8H), 1.00 – 0.84 (m, 6H). **^{19}F NMR** (282 MHz, $CDCl_3$) δ -75.57, -114.62 (q, *J* = 8.1 Hz). **^{13}C NMR** (75 MHz, $CDCl_3$) δ 161.0 (d, *J* = 245.6 Hz, C), 137.5 (C), 137.0 (d, *J* = 10.6 Hz, C), 129.9 (d, *J* = 8.9 Hz, CH), 128.5 (d, *J* = 3.2 Hz, C), 122.6 (C), 120.1 (d, *J* = 324.5 Hz, C), 114.6 (d, *J* = 21.4 Hz, CH), 112.1 (d, *J* = 25.5 Hz, CH), 50.0 (CH_2), 32.03 (CH_2), 31.97 (CH_2), 30.9 (CH_2), 30.3 (CH_2), 30.1 (CH_2), 23.1 (CH_2), 23.0 (CH_2), 14.2 (CH_3), 14.1 (CH_3). **HRMS** [APCI]: *m/z* calculated for $C_{19}H_{25}F_4NO_2S$ $[M]^+$: 407.1542, found 407.1544.

8-Methyl-3-(nonan-5-ylidene)-6-phenyl-1-((trifluoromethyl)sulfonyl)-1,2,3,4-tetrahydroquinoline (3la)

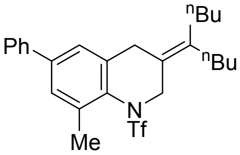

3la

(42.2 mg, 53% yield), obtained as a brown oil. **^{1}H NMR** (500 MHz, $CDCl_3$) δ 7.52 – 7.45 (m, 2H), 7.39 – 7.33 (m, 2H), 7.32 – 7.24 (m, 2H), 7.20 – 7.17 (m, 1H), 4.82 (d, *J* = 16.7 Hz, 1H), 3.87 (d, *J* = 16.7 Hz, 1H), 3.59 (d, *J* = 18.2 Hz, 1H), 3.45 (d, *J* = 18.2 Hz, 1H), 2.41 (s, 3H), 2.09 – 1.95 (m, 2H), 1.97 – 1.84 (m, 2H), 1.36 – 1.18 (m, 8H), 0.91 – 0.79 (m, 6H). **^{19}F NMR** (282 MHz, $CDCl_3$) δ -74.69. **^{13}C NMR** (126 MHz, $CDCl_3$) δ 141.4 (C), 140.3 (C), 136.8 (C), 136.4 (C), 136.1 (C), 134.8 (C), 128.9 (CH), 128.6 (CH), 127.8 (CH), 127.3 (CH), 124.9 (CH), 124.3 (C), 120.2 (q, *J* = 325.2 Hz, C), 51.4 (CH_2), 32.4 (CH_2), 32.0 (CH_2), 31.8 (CH_2), 30.3 (CH_2), 30.2 (CH_2), 23.1 (CH_2), 19.5 (CH_3), 14.2 (CH_3), 14.1 (CH_3). **HRMS** [APCI]: *m/z* calculated for $C_{26}H_{32}F_3NO_2S$ $[M]^+$: 479.2100, found 479.2098.

6,8-Dimethyl-3-(nonan-5-ylidene)-1-((trifluoromethyl)sulfonyl)-1,2,3,4-tetrahydroquinoline (3ma)

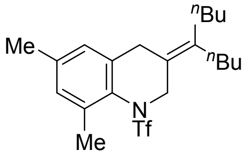

3ma

(36.1 mg, 52% yield), obtained as a yellow oil. **^{1}H NMR** (500 MHz, $CDCl_3$) δ 6.96 (s, 1H), 6.87 (s, 1H), 4.85 (d, *J* = 16.7 Hz, 1H), 3.88 (d, *J* = 16.7 Hz, 1H), 3.54 (d, *J* = 18.1 Hz, 1H), 3.43 (d, *J* = 18.2 Hz, 1H), 2.38 (s, 3H), 2.30 (s, 3H), 2.13 – 2.01 (m, 2H), 2.03 – 1.90 (m, 2H), 1.40 – 1.23 (m, 8H), 1.00 – 0.86 (m, 6H). **^{19}F NMR** (282 MHz, $CDCl_3$) δ -74.42. **^{13}C NMR** (126 MHz, $CDCl_3$) δ 138.3 (C), 136.2 (C), 135.8 (C), 135.7 (C), 133.0 (C), 130.5 (CH), 126.8 (CH), 124.5 (C), 120.2 (q, *J* = 325.3 Hz, C), 51.4 (CH_2), 32.3 (CH_2), 32.0 (CH_2), 31.7 (CH_2), 31.5 (CH_2), 30.3 (CH_2), 30.2 (CH_2), 23.0 (CH_3), 21.1 (CH_3), 19.1 (CH_2), 14.2 (CH_3), 14.1 (CH_3). **HRMS** [APCI]: *m/z* calculated for $C_{21}H_{31}F_3NO_2S$ $[M+H]^+$: 418.2022, found 418.2023.

3-(Nonan-5-ylidene)-1-((trifluoromethyl)sulfonyl)-1,2,3,4-tetrahydrobenzo[g]quinoline (3na)

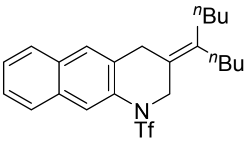

3na

(49.0 mg, 67% yield), obtained as a black oil. **^{1}H NMR** (300 MHz, $CDCl_3$) δ 8.01 (s, 1H), 7.87 – 7.75 (m, 2H), 7.68 (s, 1H), 7.53 – 7.44 (m, 2H), 4.54 (s, 2H), 3.70 (s, 2H), 2.19 – 1.98 (m, 4H), 1.45 – 1.28 (m, 8H), 1.00 – 0.87 (m, 6H). **^{19}F NMR** (282 MHz, $CHCl_3$) δ -75.63. **^{13}C NMR** (75 MHz, $CHCl_3$) δ 136.6 (C), 134.3 (C), 132.5 (C), 132.3 (C), 131.8 (C), 128.3 (CH), 127.2 (CH), 127.0 (CH), 126.8 (CH), 126.3 (CH), 123.4 (C), 123.2 (CH), 120.2 (d, *J* = 324.2 Hz, C), 50.7 (CH_2), 32.1 (CH_2), 32.0 (CH_2), 31.3 (CH_2), 30.6 (CH_2), 30.4 (CH_2), 23.1 (CH_2), 23.0 (CH_2), 14.2 (CH_3), 14.1 (CH_3). **HRMS** [APCI]: *m/z* calculated for $C_{23}H_{28}F_3NO_2S$ $[M]^+$: 439.1793, found 439.1796.

(*E*)-3-Benzylidene-2,2-dimethyl-6-phenyl-1-((trifluoromethyl)sulfonyl)-1,2,3,4-tetrahydroquinoline (3fh)

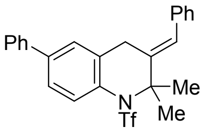

3fh

(62.4 mg, 82% yield), obtained as a yellow solid. **^{1}H NMR** (500 MHz, $CDCl_3$) δ 7.50 – 7.13 (m, 13H), 6.42 (s, 1H), 3.73 (s, 2H), 1.66 (s, 6H). **^{19}F NMR** (282 MHz, $CDCl_3$) δ -75.09. **^{13}C NMR** (126 MHz, $CDCl_3$) δ 143.0 (C), 141.2 (C), 140.0 (C), 136.8 (C), 136.2 (C), 134.6 (C), 128.99 (CH), 128.95 (CH), 128.6 (CH), 128.3 (CH), 127.8 (CH), 127.3 (CH), 127.2 (CH), 126.2 (CH), 125.8 (CH), 123.7 (CH), 120.1 (q, *J* = 324.8 Hz, C), 68.7 (C), 31.5 (CH_2), 29.7 (CH_3). **HRMS** [APCI]: *m/z* calculated for $C_{25}H_{22}F_3NO_2S$ $[M]^+$: 457.1323, found 457.1326.

Gram scale synthesis of 3ae and 3ae'

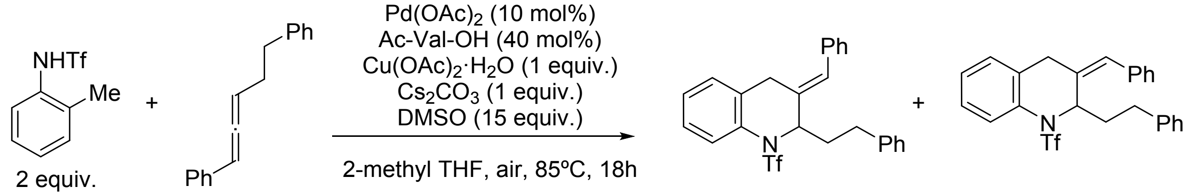

1a **2e** **3ae** **3ae'**

To a solution of $Pd(OAc)_2$ (51.0 mg, 10 mol%), Ac-Val-OH (144.5 mg, 40 mol%), $Cu(OAc)_2$•H_2O (453.1 mg, 1 equiv.), Cs_2CO_3 (739.4 mg, 1 equiv.) and **1a** (1.09 g, 4.54 mmol, 2 equiv.) in 2-methyl THF (7 mL) and DMSO (2.4 mL, 15 equiv.), under air atmosphere, heated at 85 ºC, in a Schlenk tube sealed with a rubber septum was slowly added the allene **2e** (500.0 mg, 2.269 mmol) with a syringe pump during 1h. The reaction was stirred at 85 ºC during 18 h using a Thermowatch-controlled heating block and then cooled to room temperature. Evaporation and column chromatography on silica gel (hexanes:diethylether; 99:1) afforded **3-Benzylidene-2-phenethyl-1-((trifluoromethyl)sulfonyl)-1,2,3,4-tetra hydroquinoline** (1.8:1 E/Z ratio of **3ae** and **3ae'**, inseparable mixture (706.2 mg, 68% yield), obtained as a yellow solid).

General procedure for the Pd-catalyzed annulation of *o*-methylbenzylamines with allenes, exemplified for 5aa:

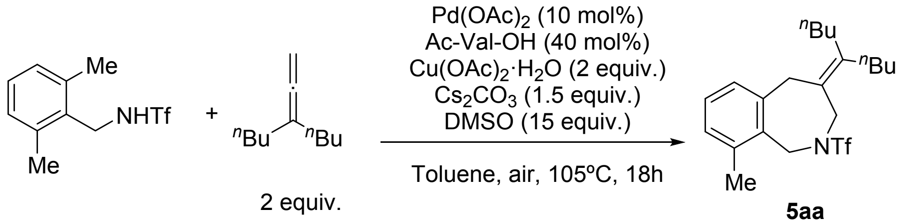

5aa

To a solution of $Pd(OAc)_2$ (3.7 mg, 10 mol%), Ac-Val-OH (10.6 mg, 40 mol%), $Cu(OAc)_2 \cdot H_2O$ (66.7 mg, 2 equiv.), Cs_2CO_3 (81.6 mg, 1.5 equiv.) and **4a** (44.6 mg, 0.167 mmol) in toluene (2 mL) and DMSO (0.178 µL, 15 equiv.), under air atmosphere, in a Schlenk tube was added the allene **2a** (50.9 mg, 0.333 mmol, 2 equiv.). The tube was sealed with a rubber septum and an air atmosphere was injected in the flask with a balloon and a needle. The reaction was heated at 105 °C using a Thermowatch-controlled heating block, stirred during 18 h and then cooled to room temperature. Evaporation and column chromatography on silica gel (hexanes:diethylether; 99:1) afforded **9-methyl-4-(nonan-5-ylidene)-2-((trifluoromethyl)sulfonyl)-2,3,4,5-tetrahydro-1H-benzo[c]azepine (5aa)** as a white solid (60.2 mg, 86% yield). **^{1}H NMR** (300 MHz, $CDCl_3$) δ 7.17 – 7.06 (m, 1H), 7.03 (d, *J* = 7.5 Hz, 2H), 4.64 (brs, 2H), 4.24 (brs, 2H), 3.62 (s, 2H), 2.34 (s, 3H), 2.14 – 2.02 (m, 4H), 1.36 – 1.21 (m, 8H), 0.98 – 0.85 (m, 6H). **^{19}F NMR** (282 MHz, $CDCl_3$) δ -76.17. **^{13}C NMR** (75 MHz, $CDCl_3$) δ 141.7 (C), 139.4 (C), 135.7 (C), 132.6 (C), 128.9 (CH), 127.9 (CH), 127.8 (CH), 124.4 (C), 120.3 (d, *J* = 323.7 Hz, C), 52.5 (CH_2), 48.3 (CH_2), 37.2 (CH_2), 32.7 (CH_2), 32.0 (CH_2), 31.5 (CH_2), 30.8 (CH_2), 23.2 (CH_2), 23.0 (CH_2), 19.8 (CH_3), 14.10 (CH_3), 14.05 (CH_3). **HRMS** [APCI]: *m/z* calculated for $C_{21}H_{31}F_3NO_2S$ $[M+H]^+$: 418.2022, found 418.2018.

9-Fluoro-4-(nonan-5-ylidene)-2-((trifluoromethyl)sulfonyl)-2,3,4,5-tetrahydro-1*H*-benzo[*c*]azepine (5ba)

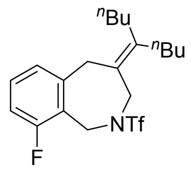

5ba

(52.7 mg, 75% yield), obtained as a yellow oil. **^{1}H NMR** (300 MHz, $CDCl_3$) δ 7.18 (q, *J* = 7.3 Hz, 1H), 7.00 – 6.85 (m, 2H), 4.68 (brs, 2H), 4.26 (brs, 2H), 3.64 (s, 2H), 2.15 – 2.01 (m, 4H), 1.38 – 1.16 (m, 8H), 0.99 – 0.80 (m, 6H). **^{19}F NMR** (282 MHz, $CDCl_3$) δ -76.35, -118.85 (dd, *J* = 9.9, 5.7 Hz). **^{13}C NMR** (75 MHz, $CDCl_3$) δ 160.0 (d, *J* = 246.4 Hz, C), 142.9 (C), 141.5 (C), 129.1 (d, *J* = 9.1 Hz, CH), 125.2 (d, *J* = 3.1 Hz, CH), 123.9 (C), 121.9 (d, *J* = 13.9 Hz, C), 120.2 (d, *J* = 323.5 Hz, C), 113.5 (d, *J* = 22.6 Hz, CH), 52.6 (CH_2), 44.3 (d, *J* = 8.4 Hz, CH_2), 36.4 (CH_2), 32.8 (CH_2), 31.9 (CH_2), 31.4 (CH_2), 30.7 (CH_2), 23.1 (CH_2), 23.0 (CH_2), 14.1 (CH_3). **HRMS** [APCI]: *m/z* calculated for $C_{20}H_{28}F_4NO_2S$ $[M+H]^+$: 422.1771, found 422.1771.

9-Methoxy-4-(nonan-5-ylidene)-2-((trifluoromethyl)sulfonyl)-2,3,4,5-tetrahydro-1*H*-benzo[*c*]azepine (5ca)

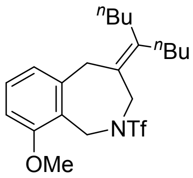

5ca

(65.4 mg, 90% yield), obtained as a white solid. **^{1}H NMR** (500 MHz, $CDCl_3$) δ 7.16 (t, *J* = 7.9 Hz, 1H), 6.75 (dd, *J* = 15.8, 7.9 Hz, 2H), 4.71 (brs, 2H), 4.23 (brs, 2H), 3.81 (s, 3H), 3.60 (s, 2H), 2.10 – 2.00 (m, 4H), 1.35 – 1.19 (m, 8H), 0.95 – 0.82 (m, 6H). **^{19}F NMR** (282 MHz, $CDCl_3$) δ -76.15. **^{13}C NMR** (126 MHz, $CDCl_3$) δ 156.6 (C), 141.4 (C), 140.4 (C), 128.5 (CH), 124.8 (C), 122.7 (C), 122.2 (CH), 120.3 (q, *J* = 323.8 Hz, C), 108.8 (CH), 55.8 (CH_3), 52.4 (CH_2), 45.4 (CH_2), 36.3 (CH_2), 32.8 (CH_2), 31.9 (CH_2), 31.4 (CH_2), 30.8 (CH_2), 23.1 (CH_2), 23.0 (CH_2), 14.09 (CH_3), 14.07 (CH_3). **HRMS** [APCI]: *m/z* calculated for $C_{21}H_{31}F_3NO_3S$ $[M+H]^+$: 434.1971, found 434.1971.

7-Methoxy-9-methyl-4-(nonan-5-ylidene)-2-((trifluoromethyl)sulfonyl)-2,3,4,5-tetrahydro-1*H*-benzo[*c*]azepine (5da)

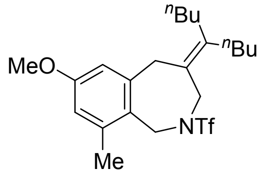

5da

(52.7 mg, 71% yield), obtained as a yellow solid. **^{1}H NMR** (300 MHz, $CDCl_3$) δ 6.58 (s, 2H), 4.59 (brs, 2H), 4.20 (brs, 2H), 3.78 (s, 3H), 3.57 (s, 2H), 2.33 (s, 3H), 2.09 (q, *J* = 7.9 Hz, 4H), 1.37 – 1.24 (m, 8H), 0.96 – 0.85 (m, 6H). **^{19}F NMR** (282 MHz, $CDCl_3$) δ -76.21. **^{13}C NMR** (75 MHz, $CDCl_3$) δ 158.8 (C), 141.9 (C), 141.1 (C), 137.4 (C), 125.0 (C), 124.1 (C), 120.2 (d, *J* = 324.2 Hz, C), 113.7 (CH), 113.4 (CH), 55.3 (CH_3), 52.3 (CH_2), 47.7 (CH_2), 37.7 (CH_2), 32.7

(CH_2), 32.0 (CH_2), 31.5 (CH_2), 30.8 (CH_2), 23.2 (CH_2), 23.0 (CH_2), 20.1 (CH_3), 14.12 (CH_3), 14.06 (CH_3). **HRMS** [APCI]: *m/z* calculated for $C_{22}H_{33}F_3NO_3S$ $[M+H]^+$: 448.2128, found 448.2126.

1-Ethyl-7,9-dimethyl-4-(nonan-5-ylidene)-2-((trifluoromethyl)sulfonyl)-2,3,4,5-tetrahydro-1*H*-benzo[*c*]azepine (5ea)

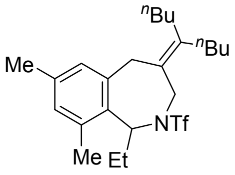

5ea

(66.6 mg, 87% yield), obtained as a yellow oil. NMR characterization performed at 50 ºC. **^{1}H NMR** (500 MHz, $CDCl_3$) δ 6.84 (s, 2H), 5.24 – 5.14 (m, 1H), 4.40 (d, *J* = 14.9 Hz, 1H), 4.19 (d, *J* = 14.9 Hz, 1H), 3.60 (q, *J* = 15.6 Hz, 2H), 2.28 (s, 3H), 2.26 (s, 3H), 2.20 – 2.03 (m, 4H), 1.95 – 1.83 (m, 1H), 1.73 – 1.60 (m, 1H), 1.45 – 1.23 (m, 8H), 1.09 (t, J = 7.4 Hz, 3H), 1.00 – 0.91 (m, 3H), 0.88 (t, *J* = 7.1 Hz, 3H). **^{19}F NMR** (282 MHz, $CDCl_3$) δ -76.45. **^{13}C NMR** (126 MHz, $CDCl_3$) δ 139.7 (C), 137.8 (C), 136.6 (C), 134.9 (C), 134.8 (C), 130.1 (CH), 129.6 (CH), 126.9 (C), 120.2 (q, *J* = 323.4 Hz, C), 62.0 (CH), 48.1 (CH_2), 37.4 (CH_2), 33.1 (CH_2), 32.4 (CH_2), 31.2 (CH_2), 30.6 (CH_2), 26.7 (CH_2), 23.13 (CH_2), 23.11 (CH_2), 20.8 (CH_3), 19.6 (CH_3), 14.0 (CH_3), 13.9 (CH_3), 11.7 (CH_3). **HRMS** [APCI]: *m/z* calculated for $C_{24}H_{37}F_3NO_2S$ $[M+H]^+$: 460.2492, found 460.2496.

(*E*)-4-Benzylidene-9-methoxy-3,3-dimethyl-2-((trifluoromethyl)sulfonyl)-2,3,4,5-tetrahydro-1*H*-benzo[*c*]azepine (5ch)

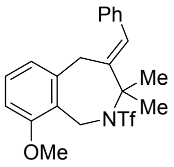

5ch

(43.5 mg, 61% yield), obtained as a white solid. **^{1}H NMR** (300 MHz, $CDCl_3$) δ 7.34 – 7.18 (m, 4H), 7.15 – 7.02 (m, 2H), 6.81 (t, *J* = 8.1 Hz, 2H), 6.66 (s, 1H), 5.42 – 4.49 (m, 2H), 3.90 – 3.79 (m, 5H), 1.87 (s, 6H). **^{19}F NMR** (282 MHz, $CDCl_3$) δ -77.21. **^{13}C NMR** (75 MHz, $CDCl_3$) δ 156.5 (C), 144.3 (C), 140.1 (C), 136.5 (C), 128.9 (CH), 128.7 (CH), 128.3 (CH), 127.3 (CH), 127.1 (CH), 124.5 (C), 121.5 (CH), 119.6 (d, *J* = 323.4 Hz, C), 108.7 (CH), 70.5 (C), 56.0 (CH_3), 44.3 (C), 35.0 (CH_2), 26.7 (CH_3). **HRMS** [APCI]: *m/z* calculated for $C_{21}H_{22}F_3NO_3S$ $[M]^+$: 425.1272, found 425.1274.

Assignment of stereochemistry of **5ch** was based on the observed nOe between the H_a (6.66 ppm, 100%) with H_b (1.87 ppm, 9.9%) as shown in the Figure S9.

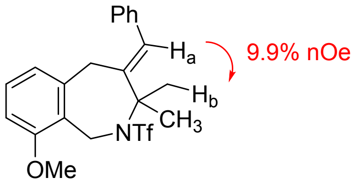

Figure S9

Reaction with a monomethylated benzylamide

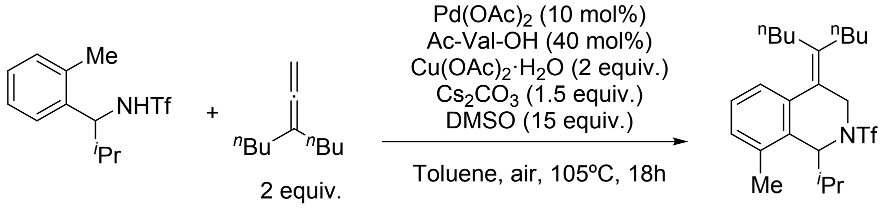

8

To a solution of $Pd(OAc)_2$ (3.7 mg, 10 mol%), Ac-Val-OH (10.6 mg, 40 mol%), $Cu(OAc)_2•H_2O$ (66.7 mg, 2 equiv.), Cs_2CO_3 (81.6 mg, 1.5 equiv.) and **4g** (50.9 mg, 0.167 mmol) in toluene (2 mL) and DMSO (0.178 µL, 15 equiv.), under air atmosphere, in a Schlenk tube was added the allene **2a** (50.9 mg, 0.333 mmol, 2 equiv.). The tube was sealed with a rubber septum and an air atmosphere was injected in the flask with a balloon and a needle. The reaction was heated at 105 °C, stirred during 18 h using a Thermowatch-controlled heating block and then cooled to room temperature. Evaporation and column chromatography on silica gel (hexanes:diethylether; 99:1) afforded **8-methyl-4-(nonan-5-ylidene)-2-((trifluoromethyl)sulfonyl)-1,2,3,4-tetrahydroisoquinoline (8)** as a yellow oil (66.2 mg, 89% yield). **^{1}H NMR** (500 MHz, $CDCl_3$) δ 7.20 – 6.98 (m, 3H), 4.55 (d, J = 10.7 Hz, 1H), 4.44 (d, J = 11.6 Hz, 1H), 4.07 (brs, 1H), 2.23 (s, 3H), 2.12 – 2.02 (m, 4H), 1.37 – 1.29 (m, 6H), 1.26 – 1.15 (m, 3H), 1.09 (d, J = 6.6 Hz, 3H), 0.89 (t, J = 7.1 Hz, 3H), 0.79 (t, J = 7.3 Hz, 3H), 0.72 (d, J = 6.8 Hz, 3H). **^{19}F NMR** (282 MHz, $CDCl_3$) δ -75.46. **^{13}C NMR** (126 MHz, $CDCl_3$) δ 140.4 (C), 137.0 (C), 134.12 (C), 134.05 (C), 129.1 (CH), 127.1 (CH), 124.0 (C), 120.1 (d, J = 324.7 Hz, C), 62.6 (CH), 46.4 (CH_2), 33.2 (CH_2), 31.5 (CH_2), 31.0 (CH_2), 30.3 (CH_2), 23.2 (CH_2), 23.0 (CH_2), 20.8 (CH), 19.4 (CH_3), 19.2 (CH_3), 14.1 (CH_3), 14.0 (CH_3). **HRMS** [APCI]: m/z calculated for $C_{23}H_{35}F_3NO_2S$ $[M+H]^+$: 446.2335, found 446.2324.

Mechanistic experiments

Synthesis of the 1,1,1-trifluoro-N-(o-tolyl)methanesulfonamide deuterated in the methyl group

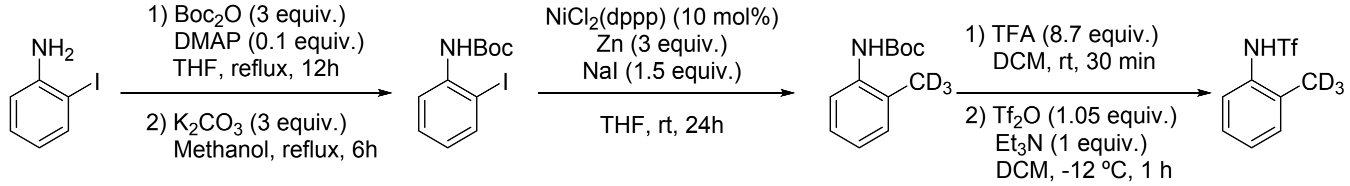

Following a previously reported procedure,[9] in a flame-dried round flask under argon atmosphere, 2-iodoaniline (1.00 g, 4.57 mmol.) was added. Then, it was dissolved in anhydrous THF (50 mL), followed by the addition of Boc_2O (2.98 g, 3 equiv.) and DMAP (56 mg, 0.1 equiv.). The solution was stirred at reflux for 12h and then cooled to room temperature. The solvent was evaporated and the crude was partitioned between 40 mL of HCl (0.5N) and 40 mL of EtOAc. The aqueous phase was extracted with EtOAc (2x40 mL) and the organic phases were washed with brine (40 mL), dried over Na_2SO_4, filtered and concentrated to afford the crude di-Boc product as a brown solid. The crude was redissolved in methanol (50 mL), followed by the addition of K_2CO_3 (1.89g, 3 equiv.). The solution was stirred at reflux for 6h. The solvent was evaporated and the crude was purified by column chromatography on silica gel (hexanes:ethyl acetate; 95:5), affording **tert-butyl (2-iodophenyl)carbamate** as a white solid (1.04 g, 71% yield). All the spectral data recorded were in agreement with those found in the corresponding literature.[9]

Following a previously reported procedure,[10] to a flame-dried Schlenk under argon atmosphere was added $NiCl_2$(dppp) (147 mg, 0.1 equiv.), zinc powder (534 mg, 3 equiv.), NaI (613 mg, 1.5 equiv.) and tert-butyl (2-iodophenyl)carbamate (870 mg, 2.73 mmol). Then, anhydrous THF (27 mL) and CDI_3 (619 µL, 3.5 equiv.) were sequentially added. The reaction was stirred at room temperature for 24h. Then, 20 mL of NH_4Cl (sat.) was added to destroy all remaining CD_3I, and the solution was stirred during 2 hours. After that, the solution was filtered through a pad of Celite©, eluted with DCM. The volatiles were evaporated, followed by a purification by column chromatography on silica gel (hexanes:diethyl ether; 95:5), affording **tert-butyl (2-(methyl-d3)phenyl)carbamate** as a white solid (451 mg, 79% yield, 100% deuterated). **^{1}H NMR** (300 MHz, $CDCl_3$) δ 7.80 (d, J = 8.1 Hz, 1H), 7.26 – 7.10 (m, 2H), 7.00 (td, J = 7.4, 1.3 Hz, 1H), 6.28 (s, 1H), 1.53 (s, 9H). **^{2}D NMR** (46 MHz, $CDCl_3$) δ 2.23 (brs). **^{13}C NMR** (75 MHz, $CDCl_3$) δ 153.2 (C), 136.5 (C), 130.4 (CH), 126.9 (CH), 123.8 (CH), 121.0 (CH), 80.5 (C), 28.5 (CH_3).

Following a general procedure for N-boc deprotection,[11] to a solution of tert-butyl (2-(methyl-d3)phenyl)carbamate (440 mg, 2.09 mmol) in DCM (7 mL) was added TFA (1.4 mL, 8.7 equiv.). The reaction was stirred at rt during 30 min. After the reaction was completed, the residue was diluted with DCM, basified with $NaHCO_3$ (sat.) until pH 8, extracted with DCM (3x10 mL), dried over Na_2SO_4, filtered and concentrated. The crude amine (228 mg) was used in the next step without further purification.

To a solution of the crude amine (220 mg, 2.00 mmol) in dichloromethane (4 mL) under argon atmosphere was added triethylamine (278 µL, 2.00 mmol) at -12ºC. After the solution was stirred 5 minutes at that temperature, trifluoromethanesulfonic anhydride (353 µL, 1.05 equiv.) was added dropwise. The reaction was stirred for 1 h at that temperature before being quenched with water. The organic layer was separated and the aqueous layer extracted with dichloromethane. The combined organic phase was washed with brine and then dried over Na_2SO_4. Evaporation and column chromatography on silica gel (hexanes:diethylether; 80:20) afforded **1,1,1-trifluoro-N-(2-(methyl-d3)phenyl)methanesulfonamide** as a white solid (370 mg, 77%). **^{1}H NMR** (300 MHz, $CDCl_3$) δ 7.42 – 7.35 (m, 1H),

7.31 – 7.19 (m, 3H), 6.82 (s, 1H). **^{2}D NMR** (46 MHz, $CDCl_3$) δ 2.36. **^{19}F NMR** (282 MHz, $CDCl_3$) δ -76.51. **^{13}C NMR** (75 MHz, $CDCl_3$) δ 133.8 (C), 132.1 (C), 131.4 (CH), 128.6 (CH), 127.4 (CH), 126.4 (CH), 119.9 (d, J = 322.3 Hz, C).

Measure of the kinetic isotopic effect (KIE) by a competition test

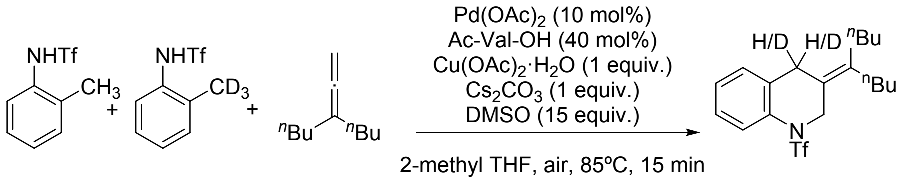

3aa + 3aa-*d*

k_H/k_D = **7.3**

To a solution of $Pd(OAc)_2$ (3.7 mg, 10 mol%), Ac-Val-OH (10.6 mg, 40 mol%), $Cu(OAc)_2•H_2O$ (33.3 mg, 1 equiv.), Cs_2CO_3 (54.4 mg, 1 equiv.), **1a** (39.9 mg, 0.167 mmol) and **1a-*d*** (40.5 mg, 0.167 mmol) in 2-methyl THF (2 mL) and DMSO (0.178 µL, 15 equiv.), under air atmosphere, in a Schlenk tube was added the allene **2a** (25.4 mg, 0.167 mmol). The tube was sealed with a rubber septum and an air atmosphere was injected in the flask with a balloon and a needle. The reaction was heated at 85 °C using a Thermowatch-controlled heating block, stirred during 15 min and then cooled to room temperature. Evaporation and column chromatography on silica gel (hexanes:diethylether; 99:1) afforded 23.5 mg of a mixture of 3aa and 3aa-*d*. The KIE value (approximately **7.3**) was obtained by integrating the H_a signal and the H^4/D signal of compound **4aa**.

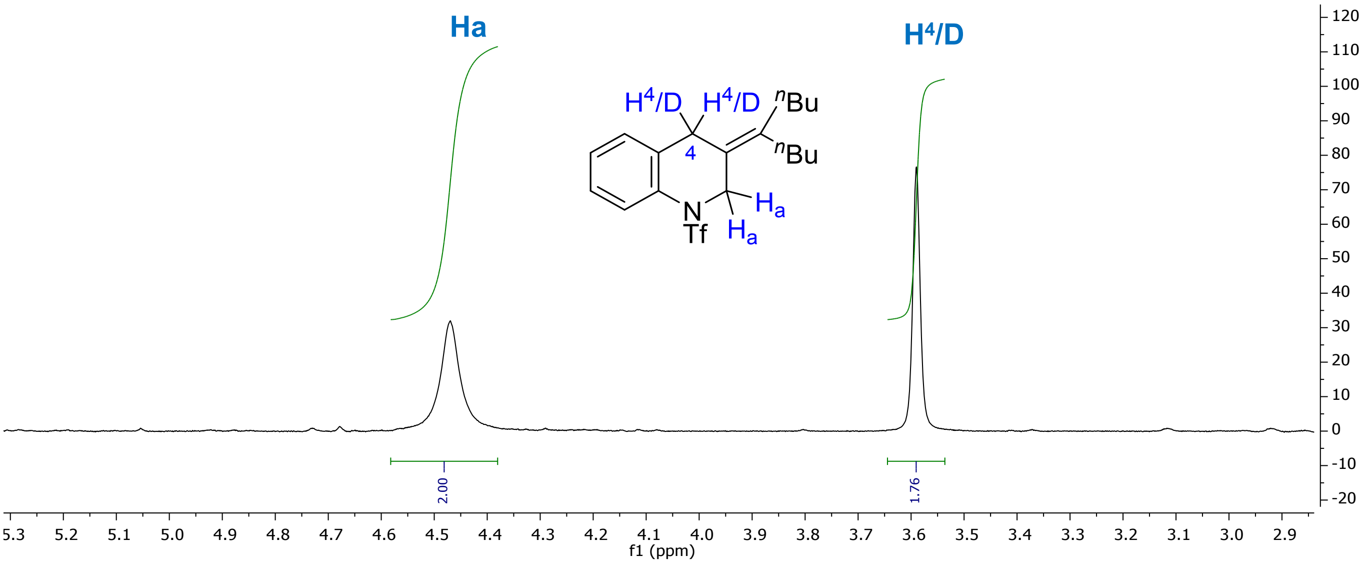

Measure of the kinetic isotopic effect (KIE) by a parallel test

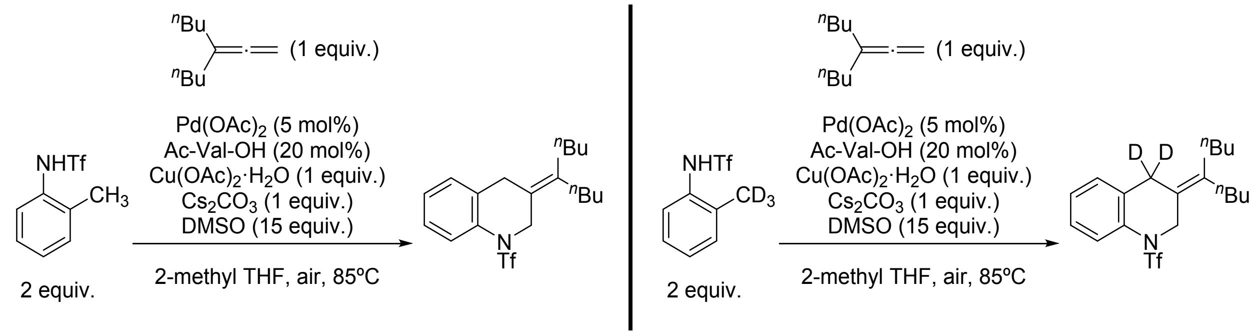

Following the general procedure for the synthesis of compounds **4**, two reactions were set using two separated Schlenk tubes under air atmosphere. $Pd(OAc)_2$ (1.9 mg, 5 mol%), Ac-Val-OH (5.3 mg, 20 mol%), $Cu(OAc)_2$•H_2O (33.3 mg, 1 equiv.), Cs_2CO_3 (54.4 mg, 1 equiv.) were added in each Schlenk. Then, in the first Schlenk, **1a** (39.9 mg, 0.167 mmol) was added, and in the second Schlenk, **1a-*d*** (40.5 mg, 0.167 mmol) was added. After that, 2-methyl THF (2 mL), DMSO (0.178 µL, 15 equiv.) and the allene **2a** (25.4 mg, 0.167 mmol) were added. The tubes were sealed with a rubber septum and stirred at 85 ºC using a Thermowatch-controlled heating block. Both test were set up with 3 minutes of delay. Aliquots of 250 µL of the reaction were taken at 5, 10, 20 and 60 min, filtered through a Florisil© pad and eluted with ethyl acetate. The volatiles were evaporated and the crude residues were analysed with ^{19}F NMR spectrometry. The parallel tests were repeated, taking from the non-deuterated experiments, aliquots of 250 µL at 10, 30, 60 and 120 min, and from the deuterated experiment, aliquots of 250 µL at 20, 40, 60 and 90 min.

To calculate KIE, the linear regression method was used. The quotient between the integral of the product signal and the integral of the remaining starting material signal was plotted as a function of time, so that the slope can be calculated with a least squares approximation. Duplicate points were calculated with the mean between the two points. KIE was calculated as the quotient between the slope of the non-deuterated experiment and the deuterated experiment, resulting in an approximate value of **2.7**.

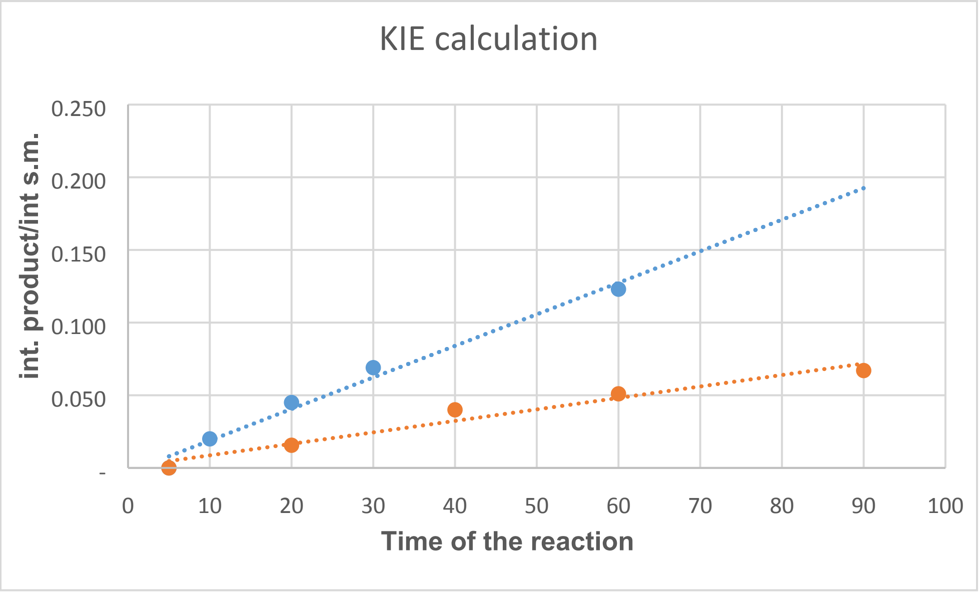

Non-deuterated

Time (min)	Ratio
5	0.000
10	0.020
20	0.045
30	0.069
60	0.123

Deuterated

Time (min)	Ratio
5	0.000
20	0.016
40	0.040
60	0.051
90	0.067

Procedure for the kinetic resolution of 4f

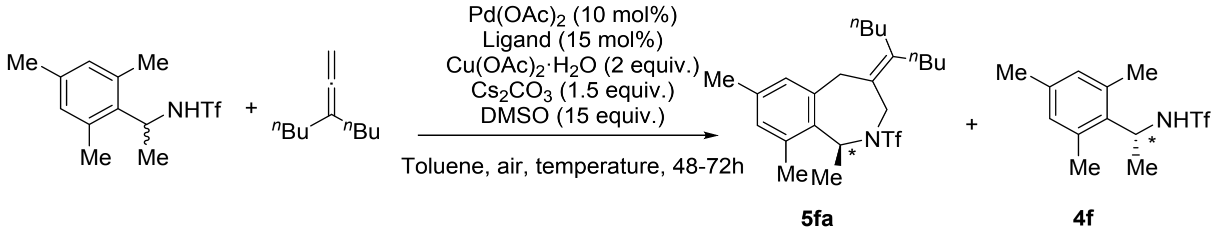

Absolute configurations not determined, stereocenters are orientative

To a solution of $Pd(OAc)_2$ (2.2 mg, 10 mol%), Ligand (15 mol%), $Cu(OAc)_2$•H_2O (39.9 mg, 2 equiv.), Cs_2CO_3 (48.9 mg, 1.5 equiv.) and **4e** (30.9 mg, 0.1 mmol) in toluene (1.5 mL) and DMSO (0.107 µL, 15 equiv.), under air atmosphere, in a Schlenk tube was added the allene **2a** (15.2 mg, 0.1 mmol, 1 equiv. or 30.4 mg, 0.2 mmol, 2 equiv.). The tube was sealed with a rubber septum and an air atmosphere was injected in the flask with a balloon and a needle. The reaction was heated at the corresponding temperature using a Thermowatch-controlled heating block, stirred during 48 - 72 h and then cooled to room temperature. Evaporation and column chromatography on silica gel (hexanes:diethylether; 99:1 – 70:30) afforded **1,7,9-trimethyl-4-(nonan-5-ylidene)-2-((trifluoromethyl)sulfonyl)-2,3,4,5-tetrahydro-1H-benzo[c]azepine (5fa)** as a yellow oil: **^{1}H NMR** (300 MHz, $CDCl_3$) δ 6.84 (s, 2H), 5.53 – 5.38 (m, 1H), 4.37 (d, *J* = 14.7 Hz, 1H), 4.24 (d, *J* = 14.8 Hz, 1H), 3.57 (s, 2H), 2.27 (d, *J* = 9.1 Hz, 6H), 2.21 – 1.90 (m, 4H), 1.45 (d, *J* = 7.3 Hz, 3H), 1.41 – 1.17 (m, 8H), 0.89 (dt, *J* = 21.3, 6.9 Hz, 6H). **^{19}F NMR** (282 MHz, $CDCl_3$) δ -76.04. **^{13}C NMR** (75 MHz, $CDCl_3$) δ 139.5 (C), 137.8 (C), 136.6 (C), 135.3 (C), 134.2 (C), 130.0 (CH), 129.5 (CH), 126.5 (C), 120.3 (d, *J* = 323.8 Hz, C), 55.6 (CH), 48.1 (CH_2), 37.1 (CH_2), 33.1 (CH_2), 32.5 (CH_2), 31.2 (CH_2), 30.6 (CH_2), 23.1 (CH_2), 20.8 (CH_3), 19.5 (CH_3), 19.4 (CH_3), 14.1 (CH_3), 14.0 (CH_3). **HRMS** [APCI]: *m/z* calculated for $C_{23}H_{35}F_3NO_2S$ $[M+H]^+$: 446.2335, found 446.2350; and remaining **4f** as a white solid.

Boc-Phe-NHOMe as ligand at 70 ºC using 1 equiv. of allene during 48h.

5fa: (15.7 mg, 38% yield).

Remaining 4f: (17.7 mg, 57% recovered).

Boc-Leu-NHOMe as ligand at 70 ºC using 1 equiv. of allene during 72h.

5fa: (17.2 mg, 42% yield).

Remaining 4f: (12.7 mg, 41% recovered).

Boc-Leu-NHOMe as ligand at 60 ºC using 2 equiv. of allene during 72h.

5fa: (8.4 mg, 20% yield).

Remaining 4f: (24.3 mg, 79% recovered).

Selectivity (*s*) was calculated with the following equation: $(s) = \ln[(1 - C)(1 - ee_{SM})]/\ln[(1 - C)(1 + ee_{SM})]$, where $C = ee_{SM}/(ee_{SM} + ee_{PR})$

*Enantioselectivity of **5fa** was determined by chiral SFC analysis on Phenomenex i-Cellulose-5 at 40 ºC (CO_2 : MeOH = 99:01, 0.5 mL/min). Enantioselectivity of **4a** was determined by chiral SFC analysis on Phenomenex Cellulose-1 at 40 ºC (CO_2 : MeOH = 98:02, 0.5 mL/min).*

Racemic sample

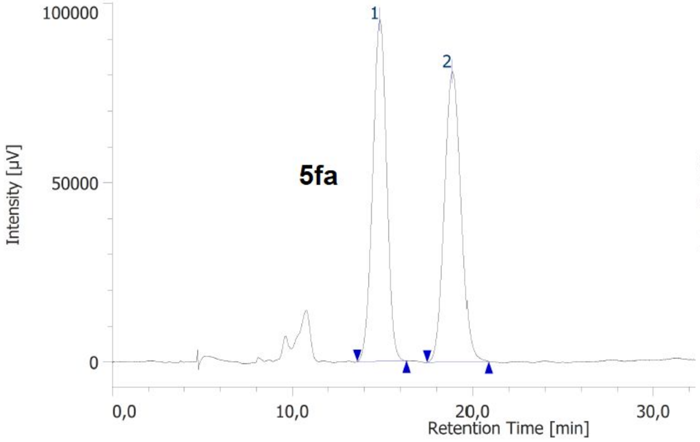

Channel Name 220,0nm

#	Peak Name	CH	tR [min]	Area [µV·sec]	Height [µV]	Area%	Height%
1	Unknown	5	14,863	5111224	95253	49,806	54,040
2	Unknown	5	18,887	5151065	81010	50,194	45,960

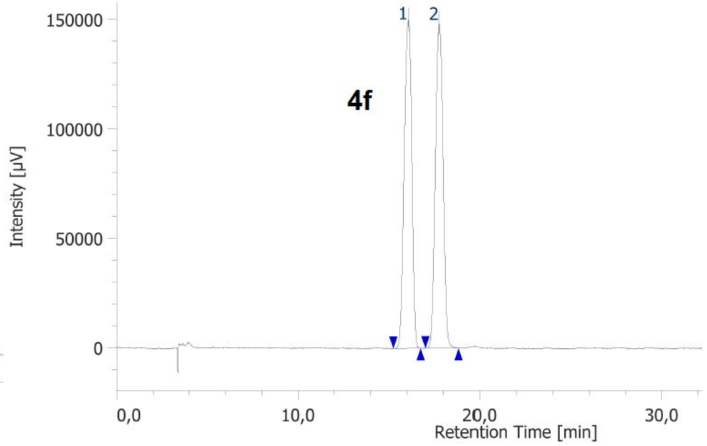

Channel Name 222,0nm

#	Peak Name	CH	tR [min]	Area [µV·sec]	Height [µV]	Area%	Height%
1	Unknown	6	16,077	4394782	150093	49,840	50,318
2	Unknown	6	17,783	4422936	148197	50,160	49,682

Asymmetric sample with Boc-Phe-NHOMe as ligand at 70 ºC using 1 equiv. of allene during 48h: (**5fa:** 86 : 14 e.r. ; **4f**: 74 : 26 e.r., *s* = 10)

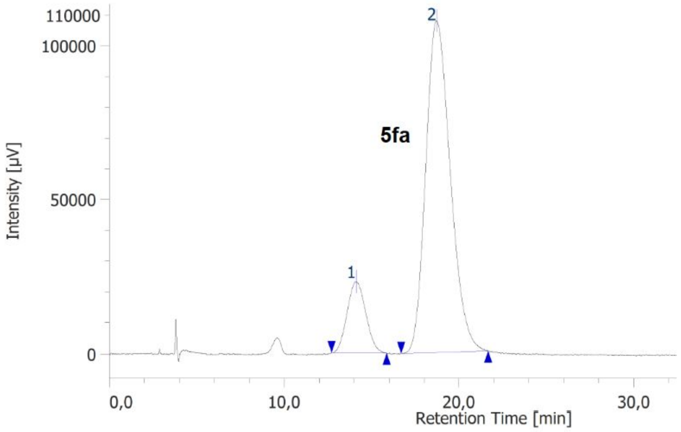

Channel Name 220,0nm

#	Peak Name	CH	tR [min]	Area [µV·sec]	Height [µV]	Area%	Height%
1	Unknown	5	14,130	1696557	22909	13,908	17,558
2	Unknown	5	18,727	10501544	107570	86,092	82,442

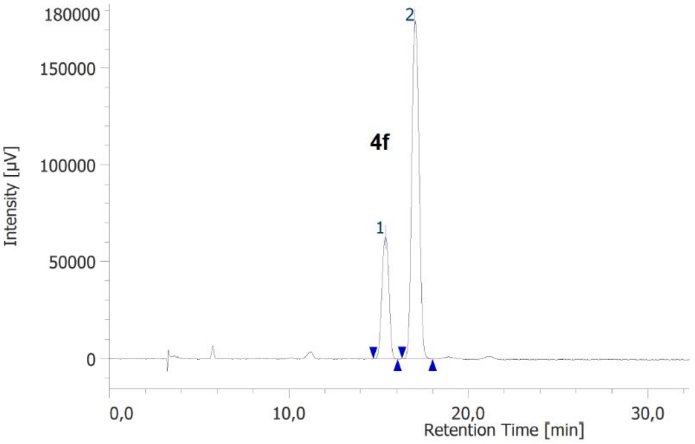

Channel Name 222,0nm

#	Peak Name	CH	tR [min]	Area [µV·sec]	Height [µV]	Area%	Height%
1	Unknown	6	15,373	1706298	62492	25,677	26,399
2	Unknown	6	17,037	4939069	174228	74,323	73,601

Asymmetric sample with Boc-Leu-NHOMe as ligand at 70 ºC using 1 equiv. of allene during 72h: (**5fa**: 88 : 12 e.r. $[\alpha]_D^{19.8}$ = -19.3 (*c* 1.0, CH_2Cl_2); **4f:** 78 : 22 e.r. $[\alpha]_D^{19.8}$ = -10.3 (*c* 1.0, CH_2Cl_2), *s* = 13).

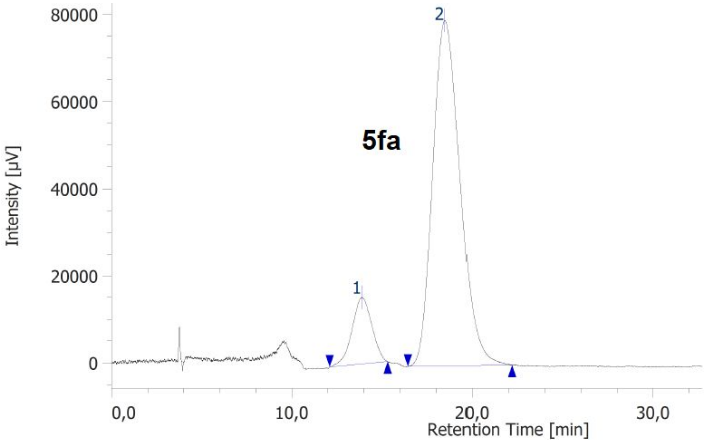

Channel Name 220,0nm

#	Peak Name	CH	tR [min]	Area [µV·sec]	Height [µV]	Area%	Height%
1	Unknown	5	13,890	1165617	15296	12,267	16,173
2	Unknown	5	18,480	8336394	79284	87,733	83,827

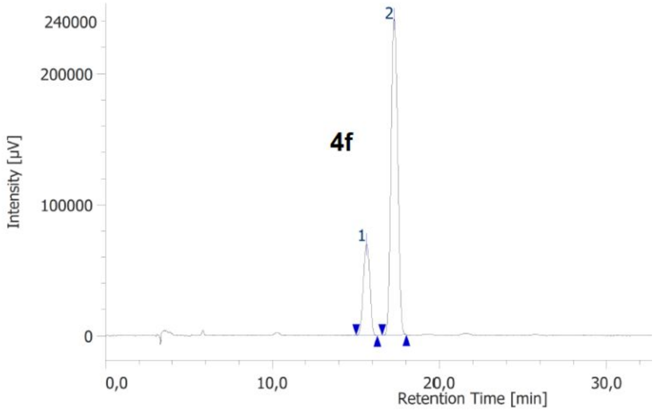

Channel Name 222,0nm

#	Peak Name	CH	tR [min]	Area [µV·sec]	Height [µV]	Area%	Height%
1	Unknown	6	15,657	1905173	69542	21,900	22,398
2	Unknown	6	17,310	6794155	240942	78,100	77,602

Asymmetric sample with Boc-Leu-NHOMe as ligand at 60 ºC using 2 equiv. of allene during 72h: (**5fa**: 90 : 10 e.r.; **4f:** 59 : 41, *s* = 11)

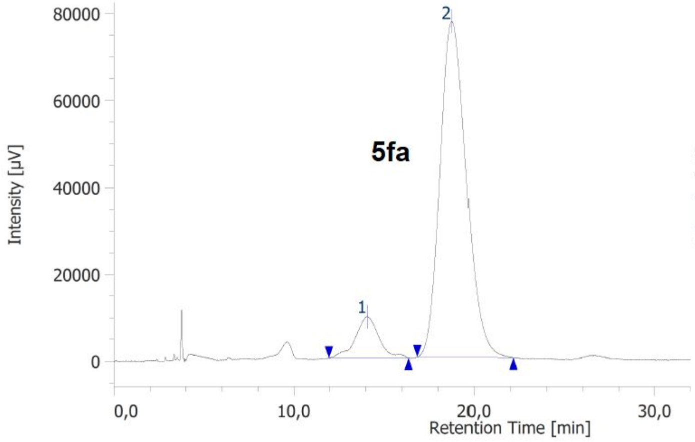

Channel Name 220,0nm

#	Peak Name	CH	tR [min]	Area [µV·sec]	Height [µV]	Area%	Height%
1	Unknown	5	14,077	867122	9522	10,022	10,973
2	Unknown	5	18,763	7785395	77254	89,978	89,027

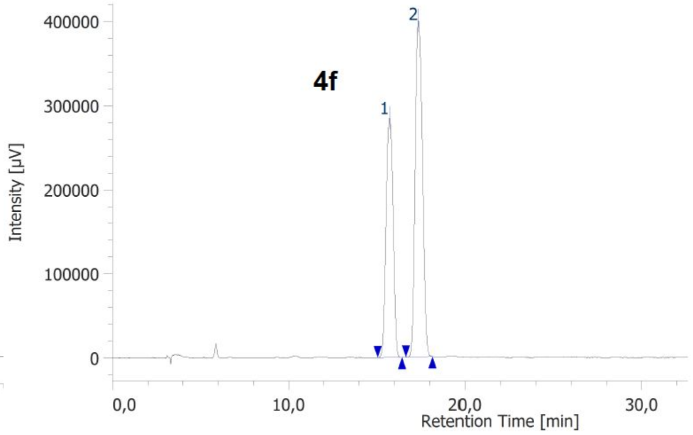

Channel Name 222,0nm

#	Peak Name	CH	tR [min]	Area [µV·sec]	Height [µV]	Area%	Height%
1	Unknown	6	15,730	7886958	284579	40,754	41,586
2	Unknown	6	17,370	11465740	399738	59,246	58,414

Procedure for the kinetic resolution of 4e

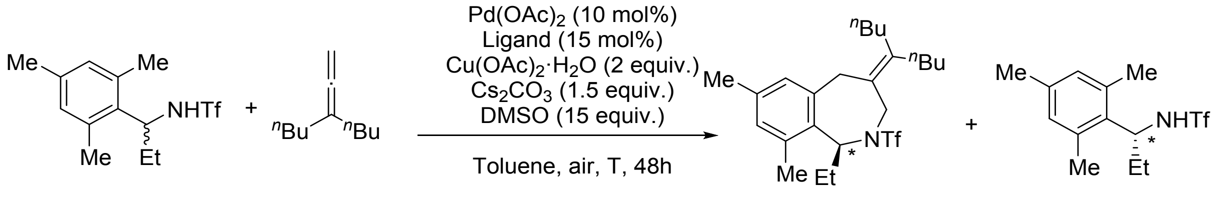

5ea **4e**

*Absolute configurations not determined, stereocenters are orientative

To a solution of $Pd(OAc)_2$ (2.2 mg, 10 mol%), Ligand (15 mol%), $Cu(OAc)_2$•H_2O (39.9 mg, 2 equiv.), Cs_2CO_3 (48.9 mg, 1.5 equiv.) and **4e** (30.9 mg, 0.1 mmol) in toluene (1.5 mL) and DMSO (0.107 µL, 15 equiv.), under air atmosphere, in a Schlenk tube was added the allene **2a** (15.2 mg, 0.1 mmol, 1 equiv.). The tube was sealed with a rubber septum and an air atmosphere was injected in the flask with a balloon and a needle. The reaction was heated at the corresponding temperature using a Thermowatch-controlled heating block, stirred during 48 h and then cooled to room temperature. Evaporation and column chromatography on silica gel (hexanes:diethylether; 99:1 – 70:30) afforded **1-ethyl-7,9-dimethyl-4-(nonan-5-ylidene)-2-((trifluoromethyl) sulfonyl)-2,3,4,5-tetrahydro-1H-benzo[c]azepine (5ea)** as a yellow oil and remaining **4e** as a white solid.

Boc-Leu-NHOMe as ligand at 80 ºC.

5ea: (22.1 mg, 48% yield).

Remaining 4e: (13.3 mg, 43% recovered).

Boc-Phe-NHOMe as ligand at 70 ºC.

5ea: (14.9 mg, 33% yield).

Remaining 4e: (17.8 mg, 58% recovered).

*Enantioselectivity of **5ea** was determined by chiral HPLC analysis on Chiralpak IF-3 at 40 ºC (Hexane, 0.5 mL/min). Enantioselectivity of **4e** was determined by chiral HPLC analysis on Chiralpak IA at 40 ºC (Hexane : iPrOH = 99:1, 0.5 mL/min).*

Racemic sample

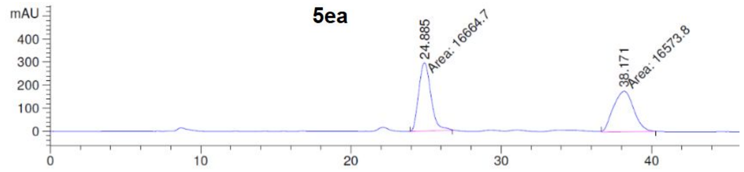

Signal 1: DAD1 A, Sig=220,8 Ref=360,100

Peak #	RetTime [min]	Type	Width [min]	Area [mAU*s]	Height [mAU]	Area %
1	24.885	MM	0.9390	1.66647e4	295.79361	50.1368
2	38.171	MM	1.5681	1.65738e4	176.16078	49.8632
Totals :				3.32385e4	471.95439	

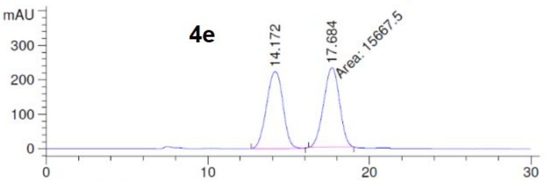

Signal 1: DAD1 A, Sig=220,8 Ref=600,100

Peak #	RetTime [min]	Type	Width [min]	Area [mAU*s]	Height [mAU]	Area %
1	14.172	BB	1.1000	1.57048e4	223.43663	50.0594
2	17.684	MM	1.1316	1.56675e4	230.75403	49.9406
Totals :				3.13722e4	454.19066	

Asymmetric sample with Boc-Leu-NHOMe as ligand at 80 ºC: (**5ea:** 78 : 22 e.r.; **4e:** 82 : 18 e.r., *s* = 7)

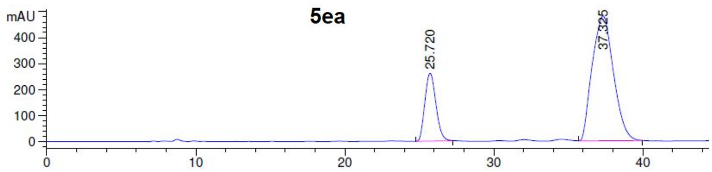

Signal 1: DAD1 A, Sig=220,8 Ref=360,100

Peak #	RetTime [min]	Type	Width [min]	Area [mAU*s]	Height [mAU]	Area %
1	25.720	PB	0.8121	1.34255e4	261.01547	21.9972
2	37.325	VB	1.3953	4.76070e4	480.75317	78.0028
Totals :				6.10325e4	741.76865	

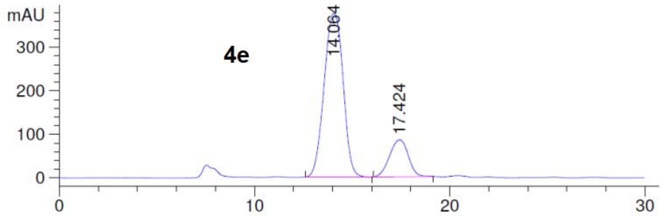

Signal 1: DAD1 A, Sig=220,8 Ref=600,100

Peak #	RetTime [min]	Type	Width [min]	Area [mAU*s]	Height [mAU]	Area %
1	14.064	BP	1.0807	2.53633e4	376.12076	81.7360
2	17.424	BB	1.0019	5667.44531	84.99376	18.2640
Totals :				3.10307e4	461.11452	

Asymmetric sample with Boc-Phe-NHOMe as ligand at 70 ºC: (**5ea:** 83 : 17 e.r.; **4e:** 69 : 31 e.r., *s* = 7)

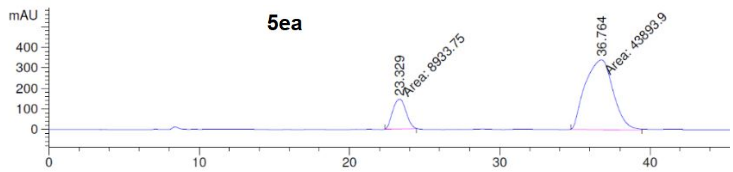

Signal 1: DAD1 A, Sig=220,8 Ref=360,100

Peak #	RetTime [min]	Type	Width [min]	Area [mAU*s]	Height [mAU]	Area %
1	23.329	MM	1.0239	8933.75195	145.41899	16.9111
2	36.764	MM	2.1518	4.38939e4	339.97278	83.0889
Totals :				5.28276e4	485.39177	

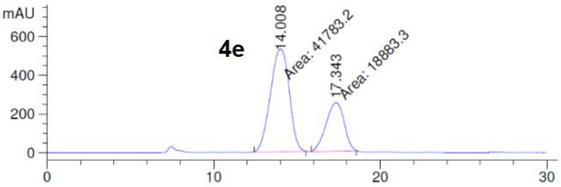

Signal 1: DAD1 A, Sig=220,8 Ref=600,100

Peak #	RetTime [min]	Type	Width [min]	Area [mAU*s]	Height [mAU]	Area %
1	14.008	MM	1.3121	4.17832e4	530.74652	68.8735
2	17.343	MM	1.2565	1.88833e4	250.48181	31.1265
Totals :				6.06665e4	781.22833	

Procedure for the hydrogenation of compound 5ch

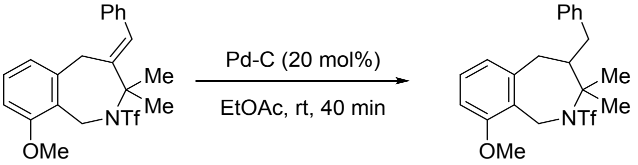

5ch **6**

To a suspension of Pd-C (20.0 mg, 20 mol%, 10% purity) in ethyl acetate (2 mL) was added **5ch** (40.0 mg, 0.094 mmol). Then, the solution was saturated with H_2 and stirred under H_2 pressure (balloon) during 40 min. Then, the solution was filtered through a Florisil© pad and eluted with ethyl acetate. The solvent was removed under reduced pressure. Flash chromatography on silica gel (hexanes:diethyl ether 99:1 – 95:5) afforded **4-benzyl-9-methoxy-3,3-dimethyl-2-((trifluoromethyl)sulfonyl)-2,3,4,5-tetrahydro-1H-benzo[c]azepine** (**6)** as a white solid (29.2 mg, 73% yield). ^{1}**H NMR** (300 MHz, $CDCl_3$) δ 7.35 – 7.09 (m, 5H), 7.03 (t, *J* = 7.6 Hz, 1H), 6.68 (d, *J* = 8.2 Hz, 1H), 6.50 (d, *J* = 7.6 Hz, 1H), 5.38 (d, *J* = 18.0 Hz, 1H), 4.44 (d, *J* = 18.0 Hz, 1H), 3.80 (s, 3H), 3.09 (dd, *J* = 16.8, 7.6 Hz, 1H), 2.87 – 2.73 (m, 2H), 2.49 – 2.31 (m, 2H), 1.64 (s, 6H). ^{19}**F NMR** (282 MHz, $CDCl_3$) δ -76.77. ^{13}**C NMR** (75 MHz, $CDCl_3$) δ 156.2 (C), 139.97 (C), 139.95 (C), 129.1 (CH), 128.7 (CH), 127.8 (CH), 126.5 (CH), 124.7 (C), 122.6 (CH), 119.9 (d, *J* = 324.2 Hz, C), 108.5 (CH), 67.9 (C), 55.9 (CH_3), 49.8 (CH), 43.5 (CH_2), 37.7 (CH_2), 34.0 (CH_2), 26.3 (CH_3), 21.8 (CH_3). **HRMS** [APCI]: *m/z* calculated for $C_{21}H_{23}F_3NO_3S$ $[M-H]^+$: 426.1345, found 426.1346. The structure of this compound was confirmed by X-Ray diffraction analysis (CCDC: 2025994).

Procedure for the removal of trifyl group of compound 6

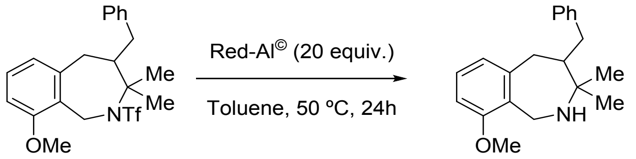

6 **7**

To a stirred solution of **6** (40.0 mg, 0.094 mmol) in toluene (1 mL) was added sodium bis(2- methoxyethoxy)-aluminum dihydride (0.28 mL, 10 equiv., 65% wt in toluene) dropwise at rt. Then, the reaction was heated at 50 ºC overnight using a Thermowatch-controlled heating block. After that, another portion of bis(2- methoxyethoxy)-aluminum dihydride (0.28 mL, 10 equiv., 65% wt in toluene) was added and the reaction was monitored by TLC. After complete conversion of the starting material, the reaction was carefully quenched at 0 ºC with 5% aqueous ammonium chloride (2 mL). The mixture was extracted with DCM (3x5 mL). The organic phases were washed with brine and dried over Na_2SO_4. Evaporation and column chromatography on silica gel (DCM:MeOH:Et_3N; 98:1:1) afforded the product **7** as a white solid (21.1 mg, 76% yield). ^{1}**H NMR** (300 MHz, $CDCl_3$) δ 7.36 – 7.11 (m, 5H), 6.98 (t, *J* = 7.9 Hz, 1H), 6.68 (d, *J* = 7.9 Hz, 1H), 6.43 (d, *J* = 7.5 Hz, 1H), 4.20 (d, *J* = 16.8 Hz, 1H), 4.04 (d, *J* = 16.7 Hz, 1H), 3.79 (s, 3H), 3.06 – 2.79 (m, 2H), 2.65 (d, *J* = 15.5 Hz, 1H), 2.26 (dd, *J* = 13.8, 11.4 Hz, 1H), 2.09 – 1.99 (m, 1H), 1.40 (s, 3H), 1.24 (s, 3H). ^{13}**C NMR** (75 MHz, $CDCl_3$) δ 156.4 (C), 142.1 (C), 141.3 (C), 129.5 (C), 129.2 (CH), 128.4 (CH), 126.8 (CH), 125.9 (CH), 122.7 (CH), 108.2 (CH), 56.3 (C), 55.6 (CH_3), 48.8 (CH), 38.5 (CH_2), 37.8 (CH_2), 33.9 (CH_2), 27.6 (CH_3), 23.8 (CH_3). **HRMS** [APCI]: *m/z* calculated for $C_{20}H_{26}NO$ $[M+H]^+$: 296.2009, found 296.2008.

Proposed mechanism for the reaction

As catalytic cycle for these annulations we propose an initial ligand exchange between the palladium acetate complex and the amino acid ligand followed by coordination to the amine to form a species of type I, which undergoes a C—H activation to form the five membered palladacycle II (a six-membered palladacycle for the benzylamides). After this turnover-limiting step, coordination of the allene is followed by regioselective migratory insertion to give a π-allylic palladacycle IV, which then evolves by reductive elimination to form the quinoline products (Scheme S1). We cannot rule out the possibility of the final step going through an outsphere mechanism, but in that case we might have observed alkenyl side products arising from β-hydride elimination processes, which were not detected. Furthermore, when external nucleophiles were added to the reaction medium, the formation products resulting from the addition to intermediate **IV** were not observed.

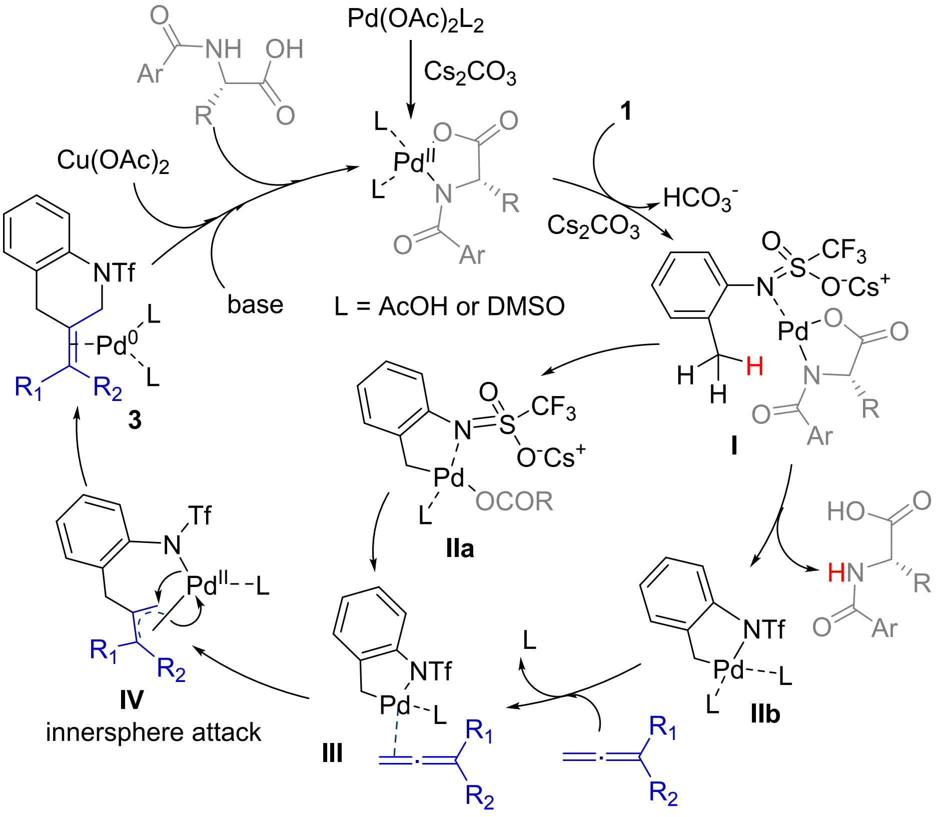

Scheme S1. Pausible mechanistic proposal.

Derivatization of the 5ch

Of course, the cycloadducts are susceptible of synthetic manipulation. For instance, the exocyclic double bond in substrate 5cg can be easily hydrogenated to give a fully saturated tetrahydrobenzazepine skeleton. Moreover, the triflyl protecting group at the nitrogen can be readily removed by treatment with Red-Al (Scheme S2).

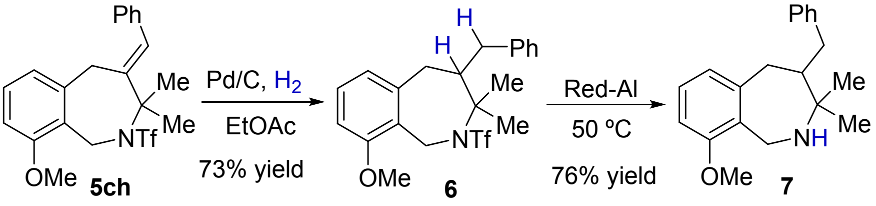

Scheme S2. Derivatization of benzazepine products.

REFERENCES

[ ] Allene **2a**: T. Kippo, T. Fukuyama, I. Ryu, *Org. Lett.* **2011**, *13*, 3864–3867. Allene **2c**: Z. Zhao, L. Racicot, G. K. Murphy, *Angew. Chemie - Int. Ed.* **2017**, *56*, 11620–11623. Allene **2d**: J. Y. Pfeiffer, S. I. Gorelsky, A. M. Beauchemin, *Org. Lett.* **2009**, *11*, 1895–1898. Allene **2e**: M. L. Hossain, F. Ye, Y. Zhang, J. Wang, *J. Org. Chem.* **2013**, *78*, 1236–1241. Allene **2g**: Gevorgyan, A.; Obst, M. F.; Guttormsen, Y.; Maseras, F.; Hopmann, K. H.; Bayer, A. *Chem. Sci.* **2019**, *10*, 10072–10078. Allene **2h** and **2j**: C.-H. Ting, Y.-L. Hsu, R.-S. Liu, *Chem. Commun.* **2012**, *48*, 6577-6579. Allene **2i**: Ahmar, M.; Barieux, J.-J.; Cazes, B.; Gore, J. *Tetrahedron* **1987**, *43*, 513–526.

[2] For 3-methyl-[1,1'-biphenyl]-4-amine and 3,5-dimethyl-[1,1'-biphenyl]-4-amine: J. Liu, R. Ma, F. Bi, F. Zhang, C. Hu, H. Venter, S. J. Semple, S. Ma, *Bioorganic and Medicinal Chemistry Letters*. 2018, 1825–1831 and for 3-methylnaphthalen-2-amine: G. Q. Li, H. Gao, C. Keene, M. Devonas, D. H. Ess, L. Kürti, *J. Am. Chem. Soc.* **2013**, *135*, 7414–7417.

[3] For 3-methyl-[1,1'-biphenyl]-4-amine: Z. Li, C. Gelbaum, W. L. Heaner, J. Fisk, A. Jaganathan, B. Holden, P. Pollet, C. L. Liotta, *Org. Process Res. Dev.* **2016**, *20*, 1489–1499, for 3,5-dimethyl-[1,1'-biphenyl]-4-amine P. J. Rayner, P. Norcott, K. M. Appleby, W. Iali, R. O. John, S. J. Hart, A. C. Whitwood, S. B. Duckett, *Nat. Commun.* **2018**, *9*, 1–11 and for 3-methylnaphthalen-2-amine: G. Q. Li, H. Gao, C. Keene, M. Devonas, D. H. Ess, L. Kürti, *J. Am. Chem. Soc.* **2013**, *135*, 7414–7417,

[4] S. Levinger, S, R. Nair, A. Hassner, *Beilstein J. Org. Chem.* **2008**, *4*, 1–5.

[5] Zhang, Y.; Lu, Z.; Desai, A.; Wulff, W. D. *Org. Lett.* **2008**, *10*, 5429–5432.

[6] J. Wang, F. Li, W. Pei, M. Yang, Y. Wu, D. Ma, F. Zhang, J. Wang, *Tetrahedron Letters* **2018**, 59, 1902–1905.

[7] X. Wang, T. S. Mei, J.-Q. Yu, *J. Am. Chem. Soc.* **2009**, *131*, 7520–7521.

[8] M. Nakatani, K. Takahashi, S. Watanabe, A. Shintokii, T. Hase, *Bulletin of the Chemical Society of Japan* **1984**, 1510–1514.

[9] S. Jana, A. Verma, R. Kadu, S. Kumar, *Chem. Sci.* **2017**, *8*, 6633–6644.

[10] L. Hu, X. Liu, X. Liao, *Angew. Chemie - Int. Ed.* **2016**, *55*, 9743–9747.

[11] Y. So, J. H. Song, D. Il. Jung, *J. Org. Chem.* **2008**, *73*, 5658–5661.

<u>Additional references that could be useful for reading the manuscript:</u>

[12] For alternative, formal (3+3) annulations to access tetrahydroquinolines that require multistep processes, see: (a) Shen, S.; Hadley, M.; Ustinova, K.; Pavlicek, J.; Knox, T.; Noonepalle, S.; Tavares, M. T.; Zimprich, C. A.; Zhang, G.; Robers, M. B.; Bařinka, C.; Kozikowski, A. P.; Villagra, A., Discovery of a New Isoxazole-3-hydroxamate-Based Histone Deacetylase 6 Inhibitor SS-208 with Antitumor Activity in Syngeneic Melanoma Mouse Models. *J. Med. Chem.* **2019,** *62*, 8557-8577. (b) Song, J.; Jones, L. M. Chavarria, G. E.; Charlton-Sevcik, A. K.; Jantz, A.; Johansen, A.; Bayeh, L.; Soeung, V.; Snyder, L. K.; Lade, S. D.; Chaplin, D. J.; Trawick, M. L.; Pinney, K. G., Small-molecule inhibitors of cathepsin L incorporating functionalized ring-fused molecular frameworks. *Bioorg. Med. Chem. Let.* **2013,** *23*, 2801-2807. (c) Minakawa, M.; Watanabe, K.; Toyoda, S.; Uozumi, Y., Iridium-Catalyzed Direct Cyclization of Aromatic Amines with Diols. *Synlett* **2018,** *29*, 2385-2389. (e) Pennington, F.; Martin, L.; Reid, R.; Lapp, T., Notes- Synthesis of 1,2,3,4-Tetrahydroquinolin-3-ols. *J. Org. Chem.* **1959,** *24*, 2030-2031.

[13] For an additional reviews in C-H activation: (a) Chen, Z.; Wang, B.; Zhang, J.; Yu, W.; Liu, Z.; Zhang, Y. Transition metal-catalyzed C-H bond functionalization by the use of diverse directing groups. *Org. Chem. Front.* **2015**, *2*, 1107-1295. (b) Abrams, D. J.; Provencher, P. A.; Sorensen, E. J. Recent applications of C–H functionalization in complex natural product synthesis. *Chem. Soc. Rev.*, **2018**, *47*, 8925-8967.

[14] For a review on the synthesis of indoles through C—H functionalization, see: (c) Guo, T.; Huang, F.; Yu, L.; Yu, Z. Indole synthesis through transition metal-catalyzed C–H activation. *Tetrahedron Lett.*, **2015**, *56*, 296-302.

CRYSTALOGRAPHIC INFORMATION

Compound 3fa

The single crystal employed for the X-ray diffraction analysis was grown via natural evaporation of a solution of **3fa** in chloroform at room temperature (CCDC: 2026002)

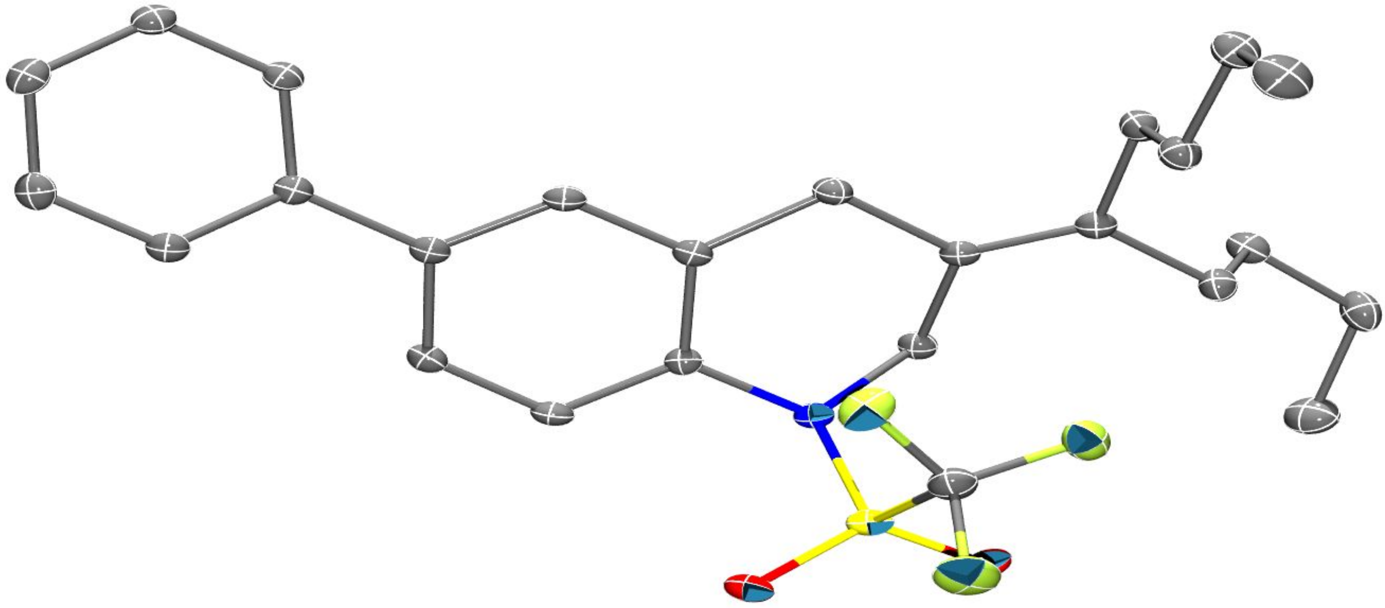

Figure S10: Ellipsoid plot of compound **3fa** at 30% probability

Table S2

**Empirical formula**	C25 H30 F3 N O2 S
**Formula weight**	465.56
**Temperature**	100.0(1) K
**Wavelength**	0.71073 Å
**Crystal system**	monoclinic
**Space group**	P 21/c
**Unit cell dimensions**	a = 19.002 Å α = 90º
	b = 9.573 Å β = 102.67º
	c = 13.598 Å γ = 90º
**Volume**	2413.3 Å^3
**Z**	4
**Density (calculated)**	1.281 Mg/m^3
**Absorption coefficient**	0.179 mm^{-1}
**F(000)**	984
**Crystal size**	0.02 x 0.06 x 0.16 mm^3
**Theta range for data collection**	2.195 to 26.14º
**Index ranges**	-23<=h<=23, -11<=k<=11, -16<=l<=16
**Reflections collected**	35051
**Independent reflections**	4927 [R(int) 0.0738]
**Completeness to theta =26.14º**	100%
**Absorption correction**	Multi-scan
**Max. and min. transmission**	0.9585 and 0.7736
**Refinement method**	Full-matrix least-squares on F^2
**Data/restraints/parameters**	4927/ 0/ 289
**Goodness-of-fit on F^2**	1.004
**Final R indices [I>2sigma(I)]**	R1 = 0.0440, wR2 = 0.0928
**R indices (all data)**	R1 = 0.0710, wR2 = 0.1063
**Largest diff. peak and hole**	0.2300 and -0.5200 e. Å^{-3}

Compound 6

The single crystal employed for the X-ray diffraction analysis was grown via natural evaporation of a solution of **6** in chloroform at room temperature (CCDC: 2025994)

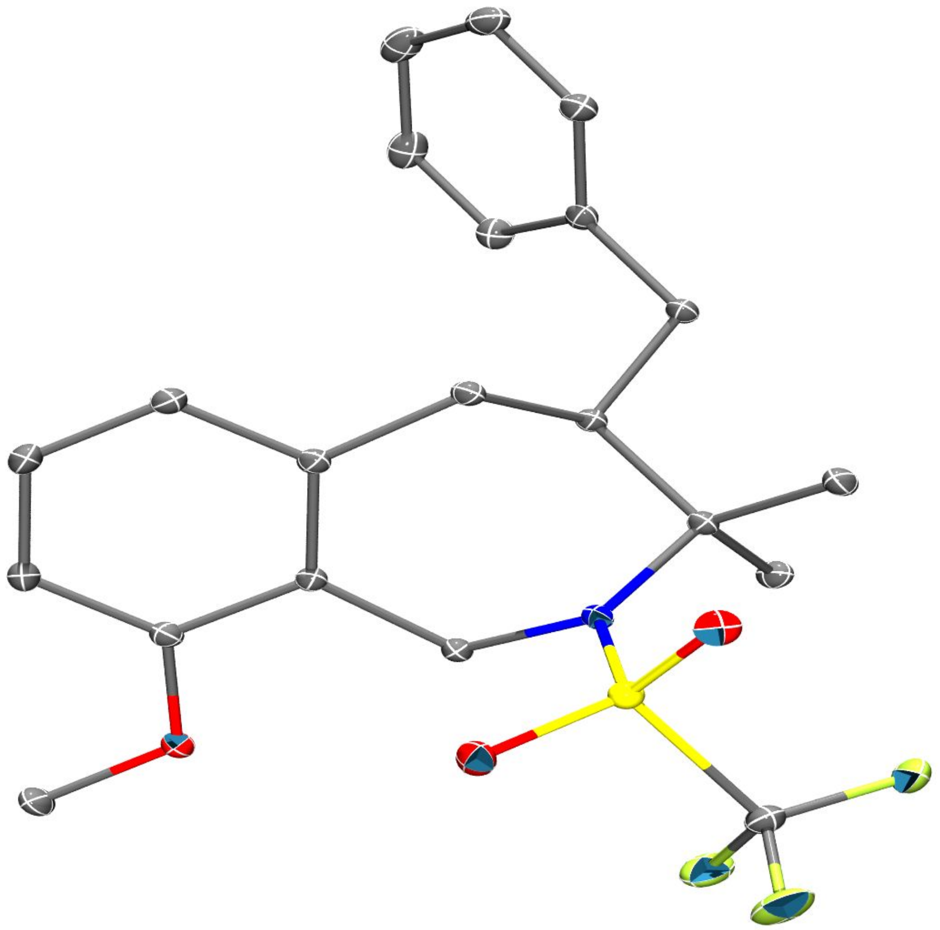

Figure S11: Ellipsoid plot of compound **6** at 30% probability

Table S3

**Empirical formula**	C21 H24 F3 N O3 S	
**Formula weight**	427.47	
**Temperature**	100.0(1) K	
**Wavelength**	0.71073 Å	
**Crystal system**	triclinic	
**Space group**	P -1	
**Unit cell dimensions**	a = 7.4504 Å	α = 105.45º
	b = 9.9847 Å	β = 94.32º
	c = 14.4860 Å	γ = 100.33º
**Volume**	1013.17 $Å^3$	
**Z**	2	
**Density (calculated)**	1.401 Mg/m^3	
**Absorption coefficient**	0.209 mm^{-1}	
**F(000)**	448	
**Crystal size**	0.32 x 0.20 x 0.06 mm^3	
**Theta range for data collection**	2.24 to 30.51º	
**Index ranges**	-10<=h<=10, -14<=k<=14, -18<=l<=20	
**Reflections collected**	30054	
**Independent reflections**	6184 [R(int) 0.0264]	
**Completeness to theta =26.14º**	99.9%	
**Absorption correction**	Multi-scan	
**Max. and min. transmission**	0.9705 and 0.9345	
**Refinement method**	Full-matrix least-squares on F^2	
**Data/restraints/parameters**	6184/ 0/ 262	
**Goodness-of-fit on F^2**	1.045	
**Final R indices [I>2sigma(I)]**	R1 = 0.0340, wR2 = 0.0923	
**R indices (all data)**	R1 = 0.0371, wR2 = 0.0948	
**Largest diff. peak and hole**	0.4700 and -0.3800 e.$Å^{-3}$	

NMR SPECTRA

^{1}H NMR (300 MHz, $CDCl_3$)

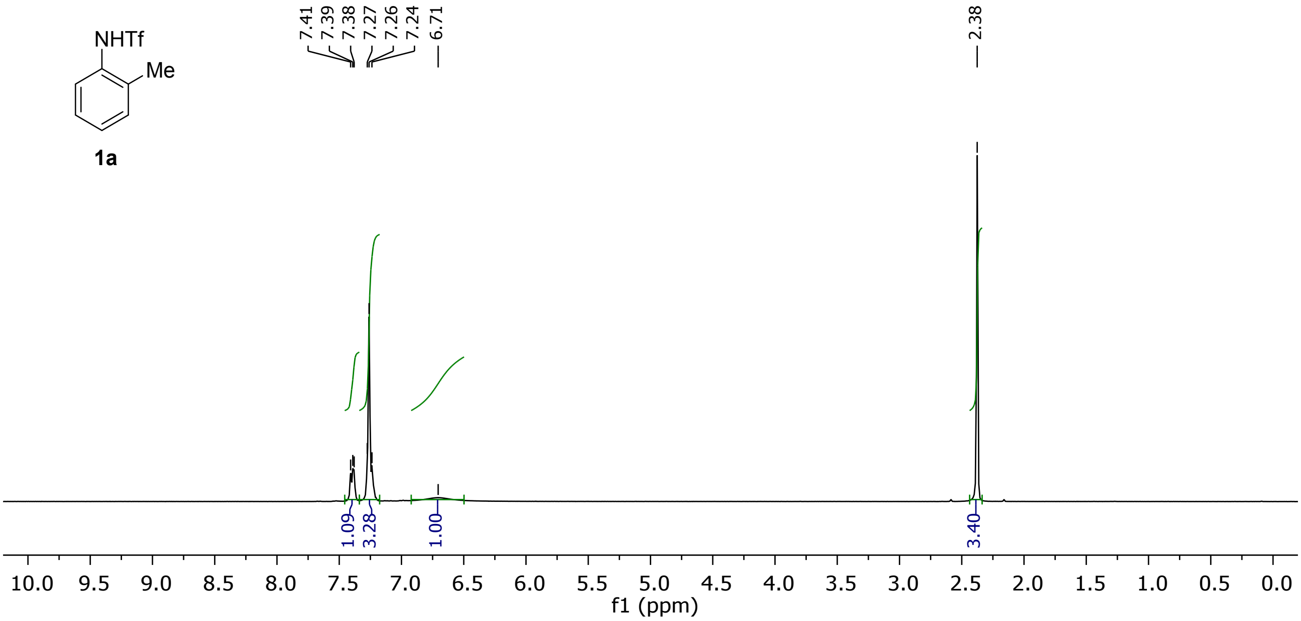

1a

DEPT-135

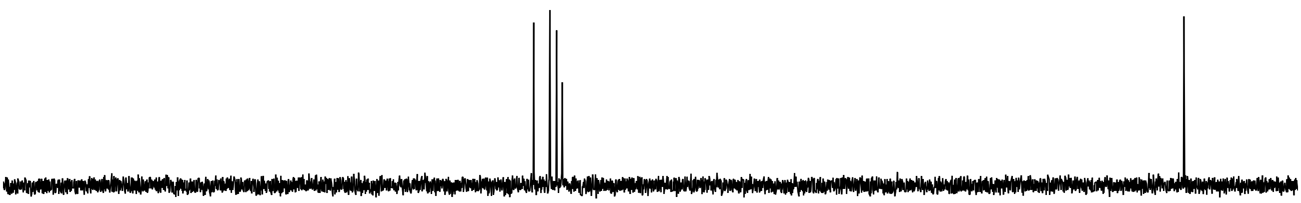

^{13}C NMR (75 MHz, $CDCl_3$)

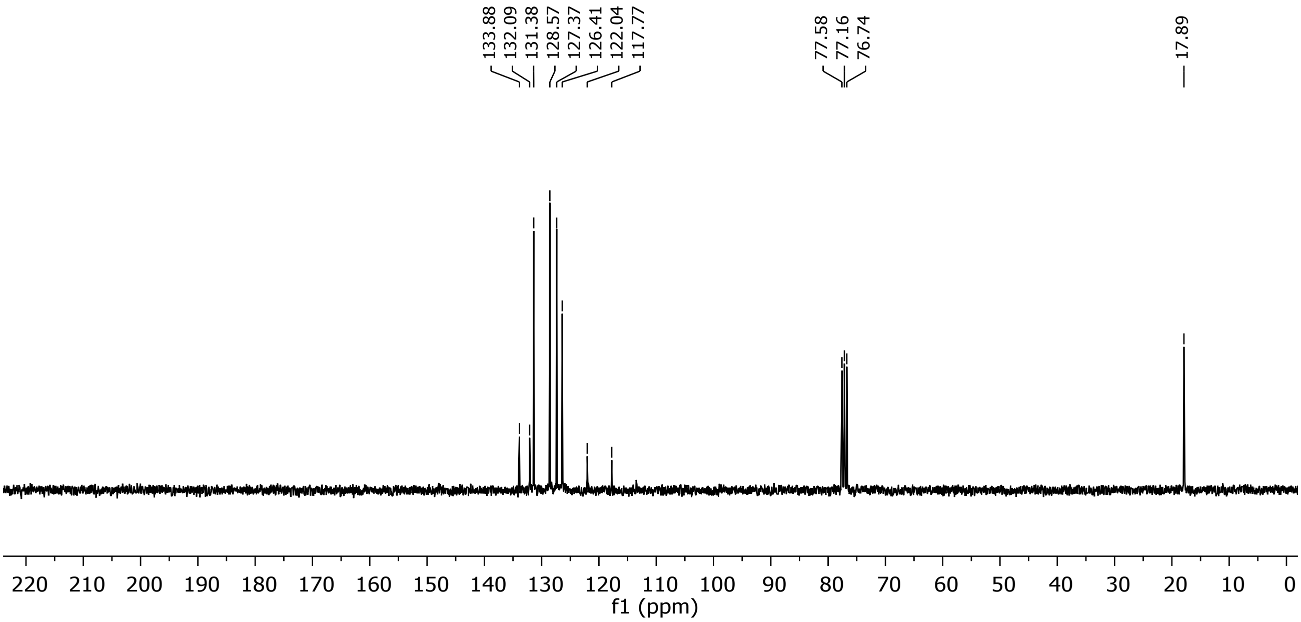

^{1}H NMR (300 MHz, $CDCl_3$)

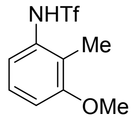

1b

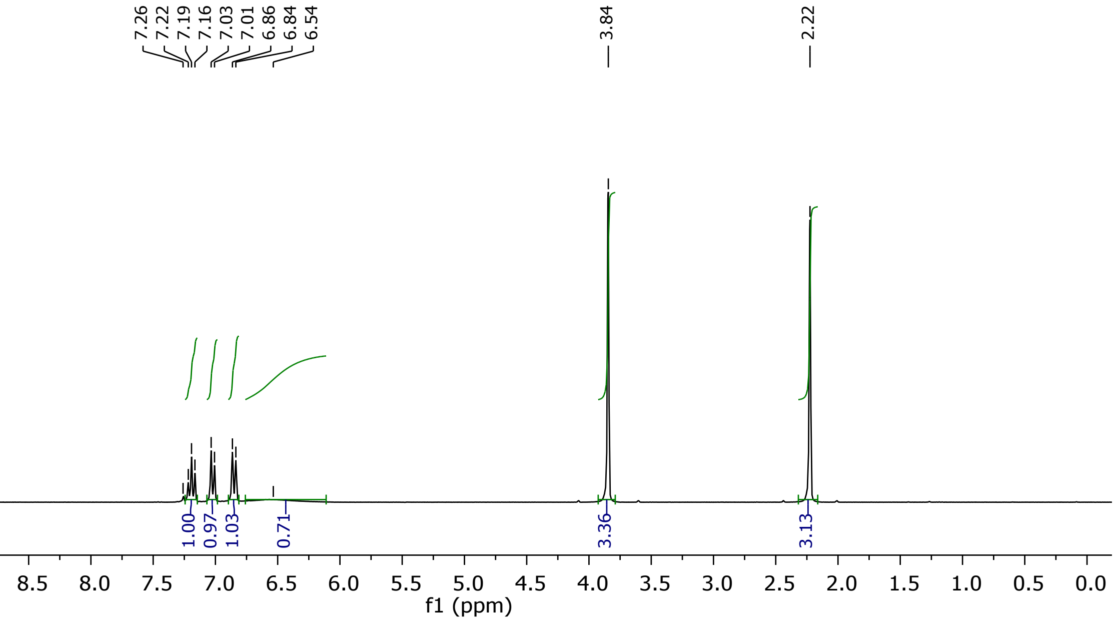

DEPT-135

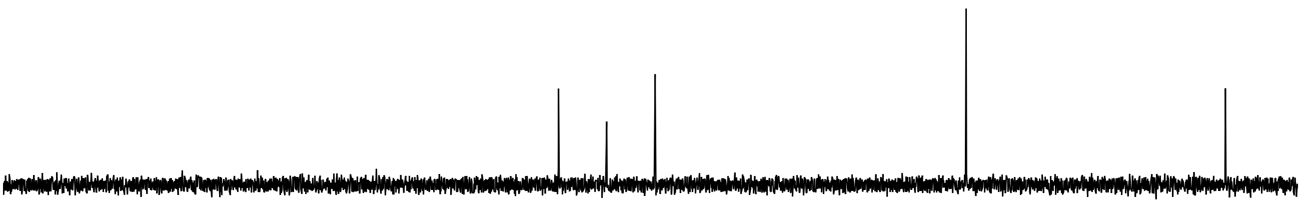

^{13}C NMR (75 MHz, $CDCl_3$)

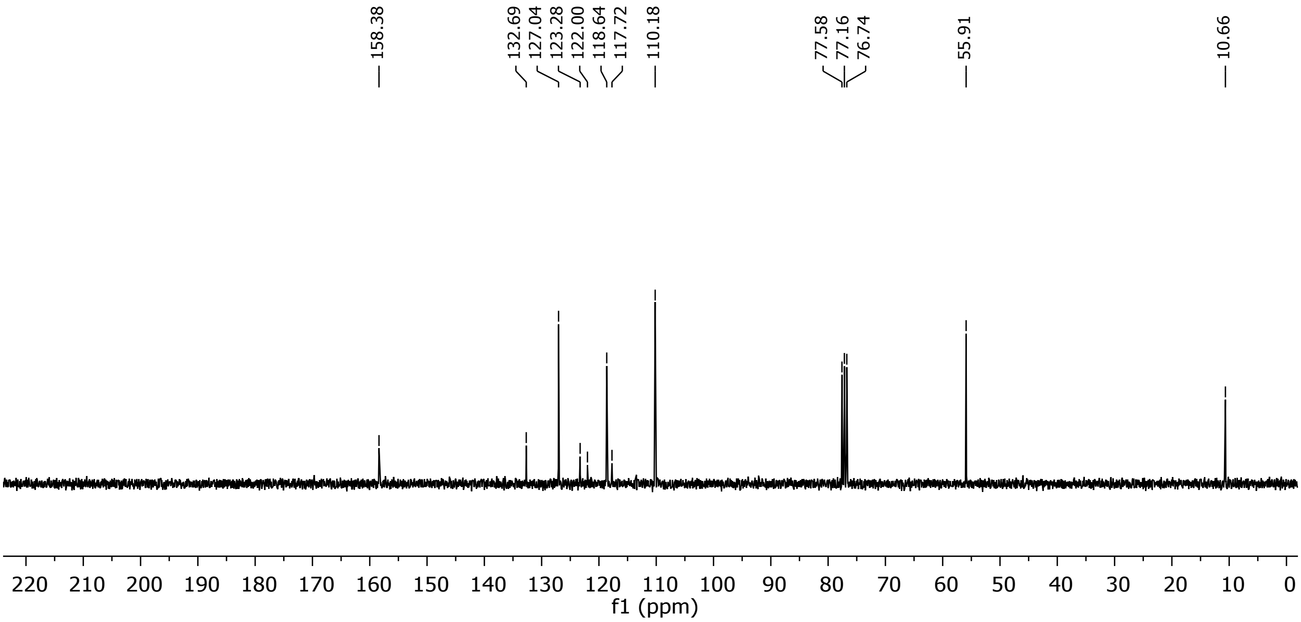

^{1}H NMR (300 MHz, $CDCl_3$)

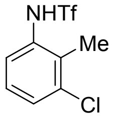

1c

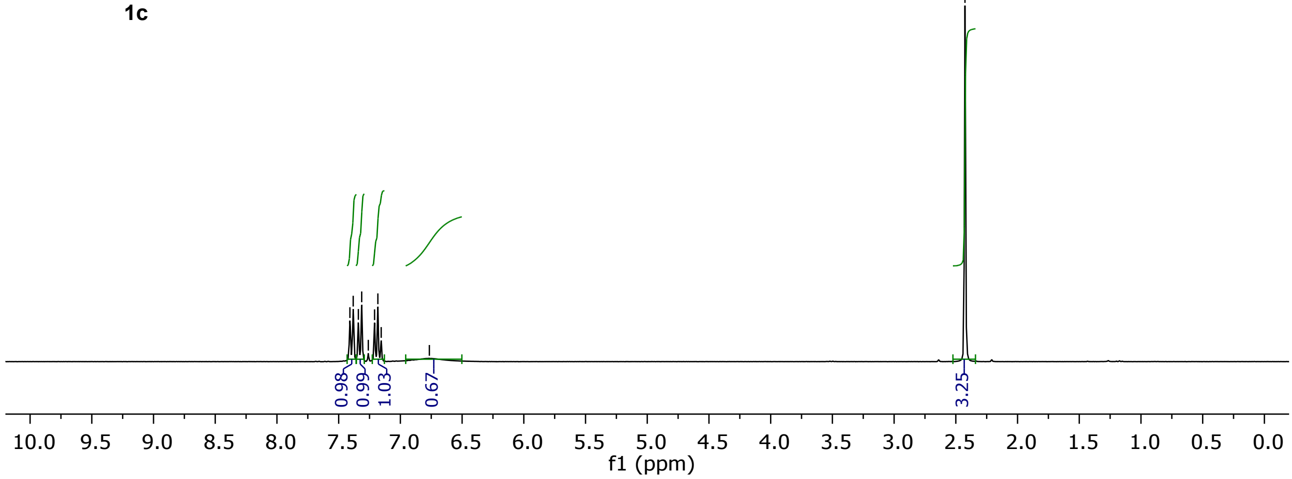

DEPT-135

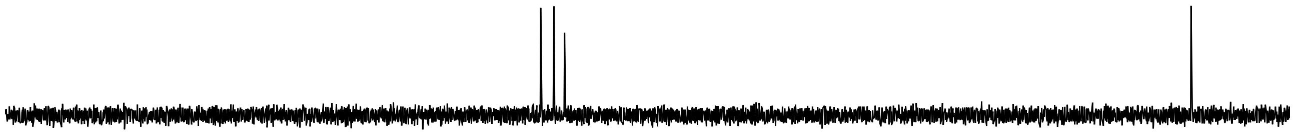

^{13}C NMR (75 MHz, $CDCl_3$)

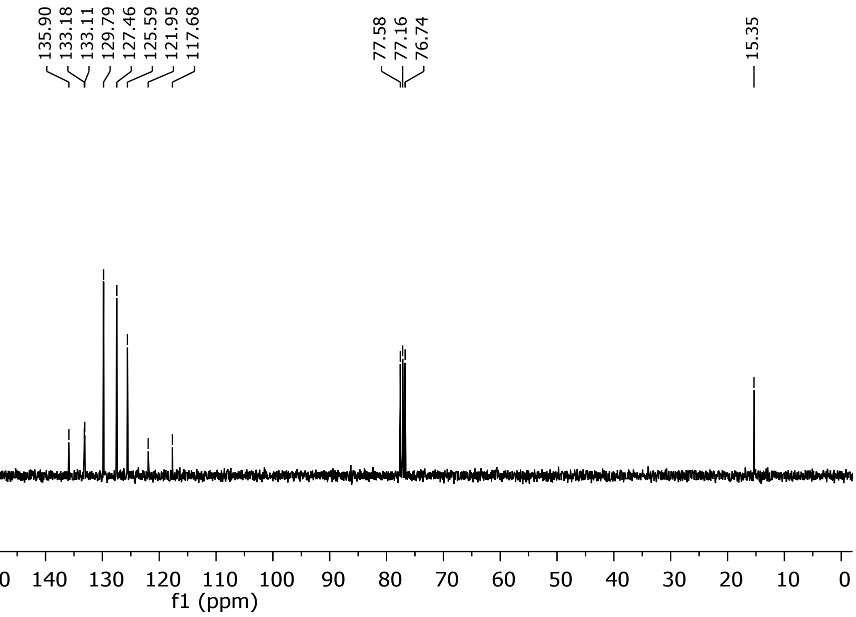

^{1}H NMR (300 MHz, $CDCl_3$)

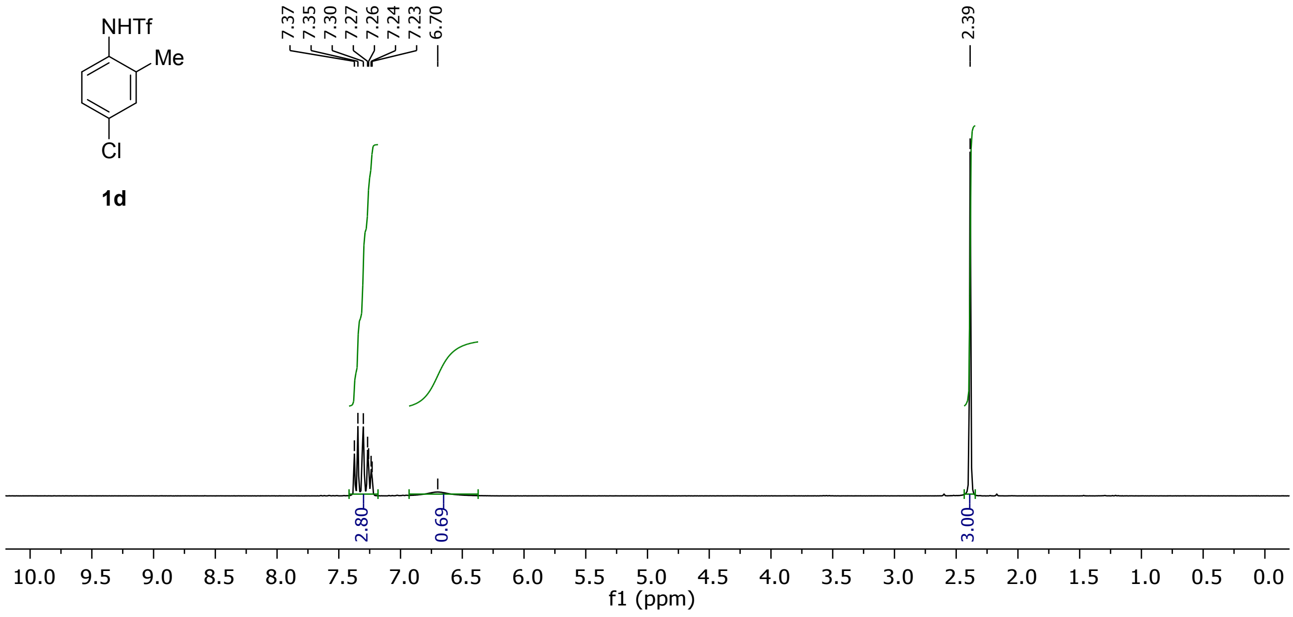

DEPT-135

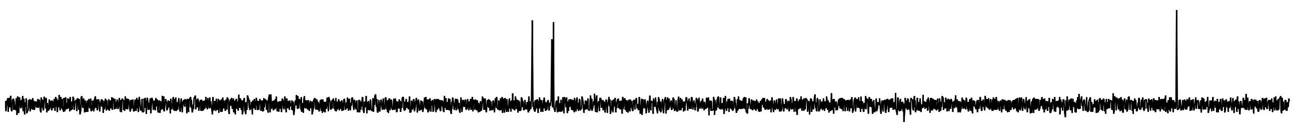

^{13}C NMR (75 MHz, $CDCl_3$)

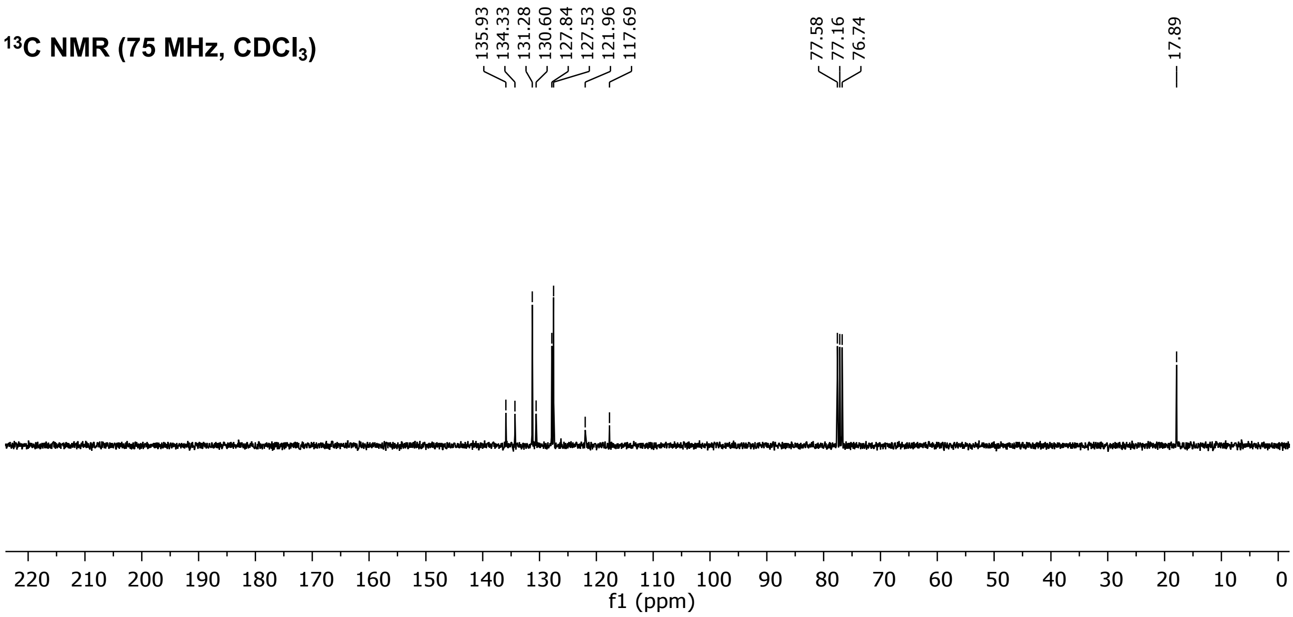

^{1}H NMR (300 MHz, $CDCl_3$)

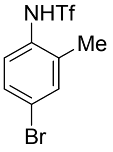

1e

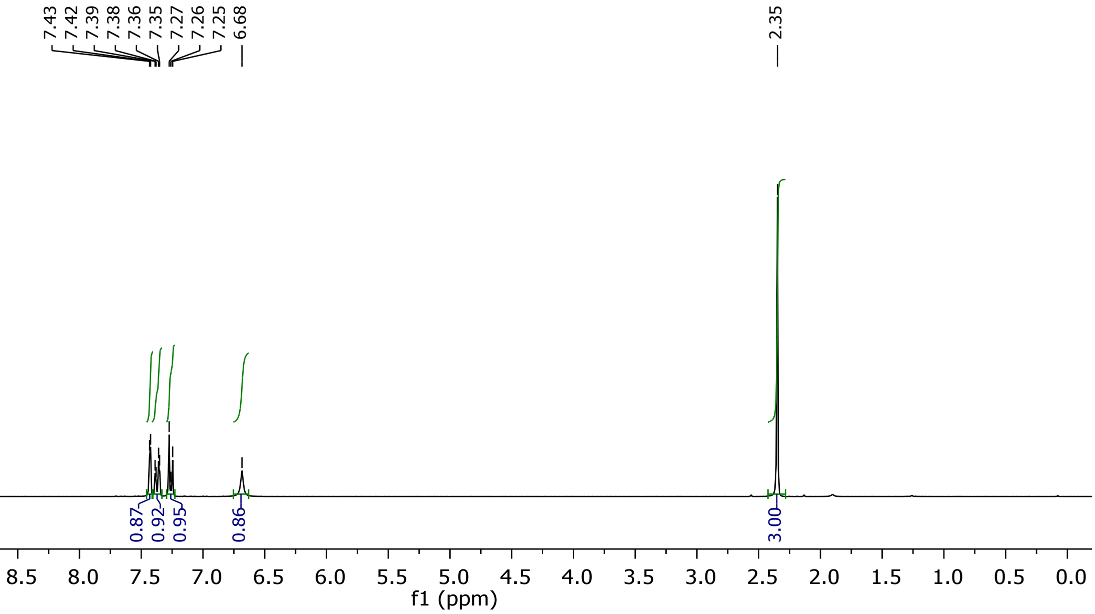

DEPT-135

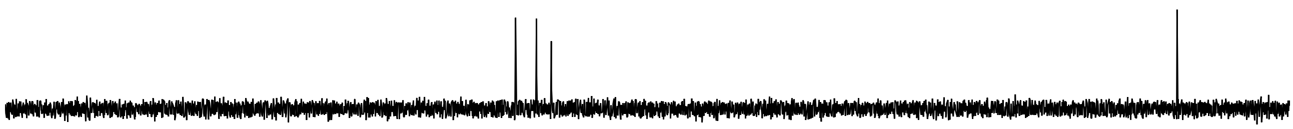

^{13}C NMR (75 MHz, $CDCl_3$)

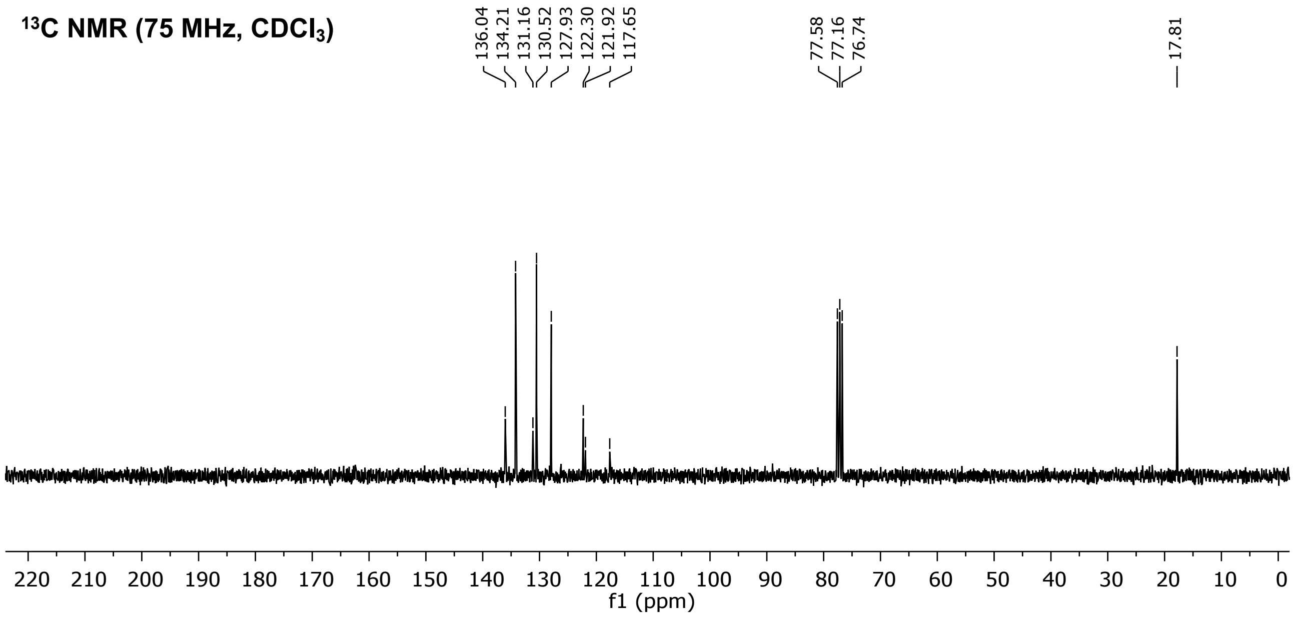

^{1}H NMR (300 MHz, CDCl$_3$)

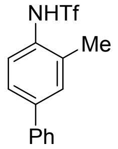

1f

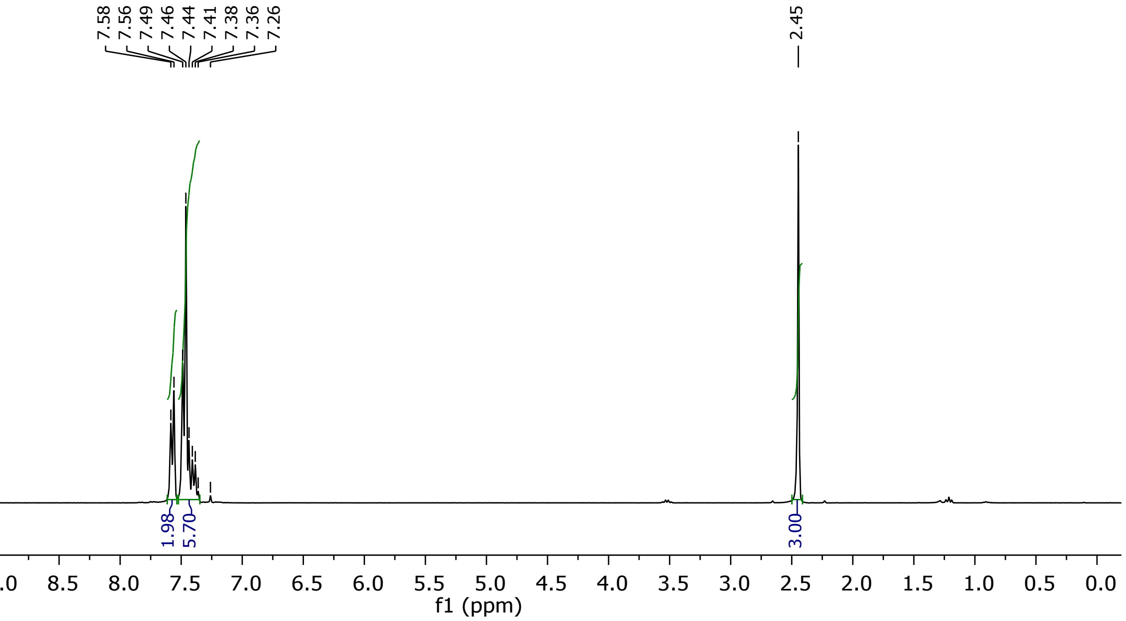

DEPT-135

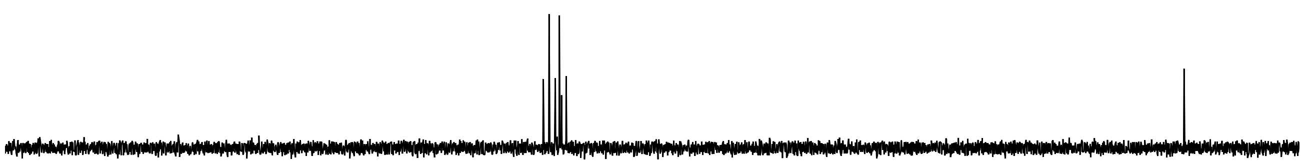

^{13}C NMR (75 MHz, CDCl$_3$)

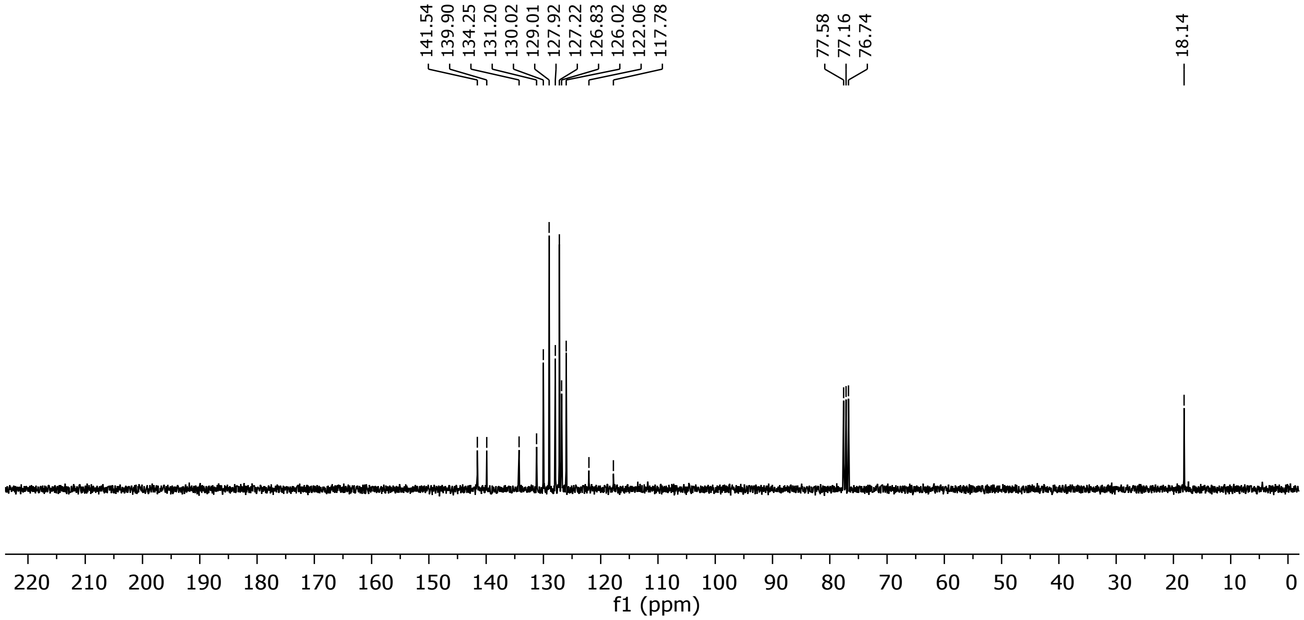

^{1}H NMR (300 MHz, CDCl$_3$)

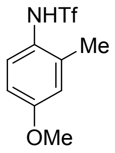

1g

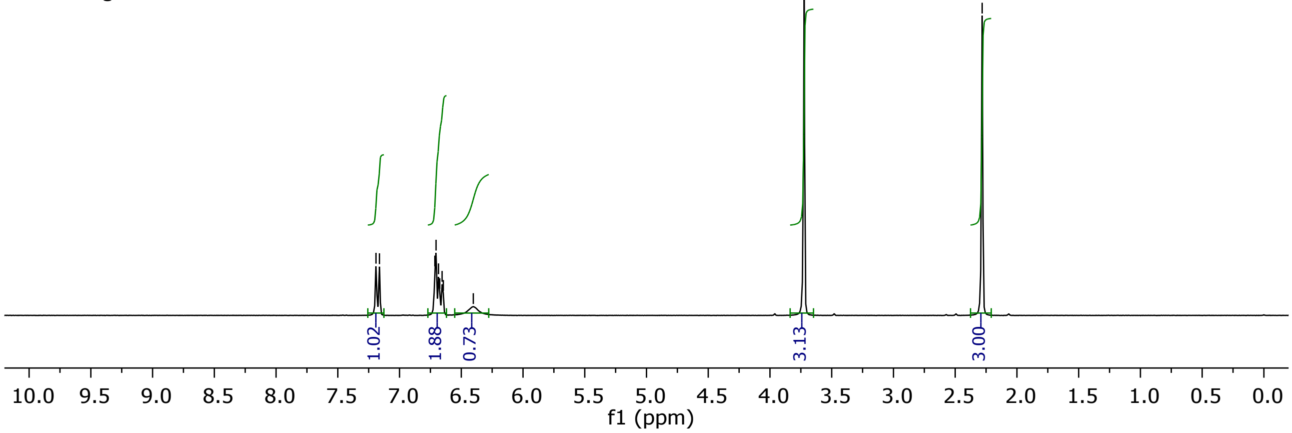

DEPT-135

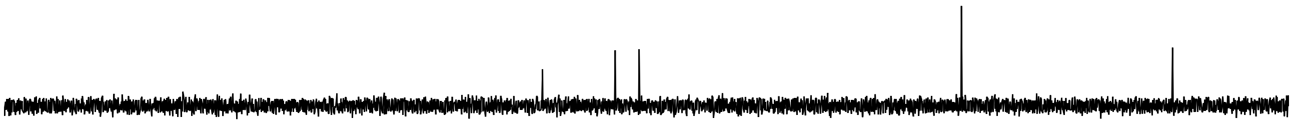

^{13}C NMR (75 MHz, CDCl$_3$)

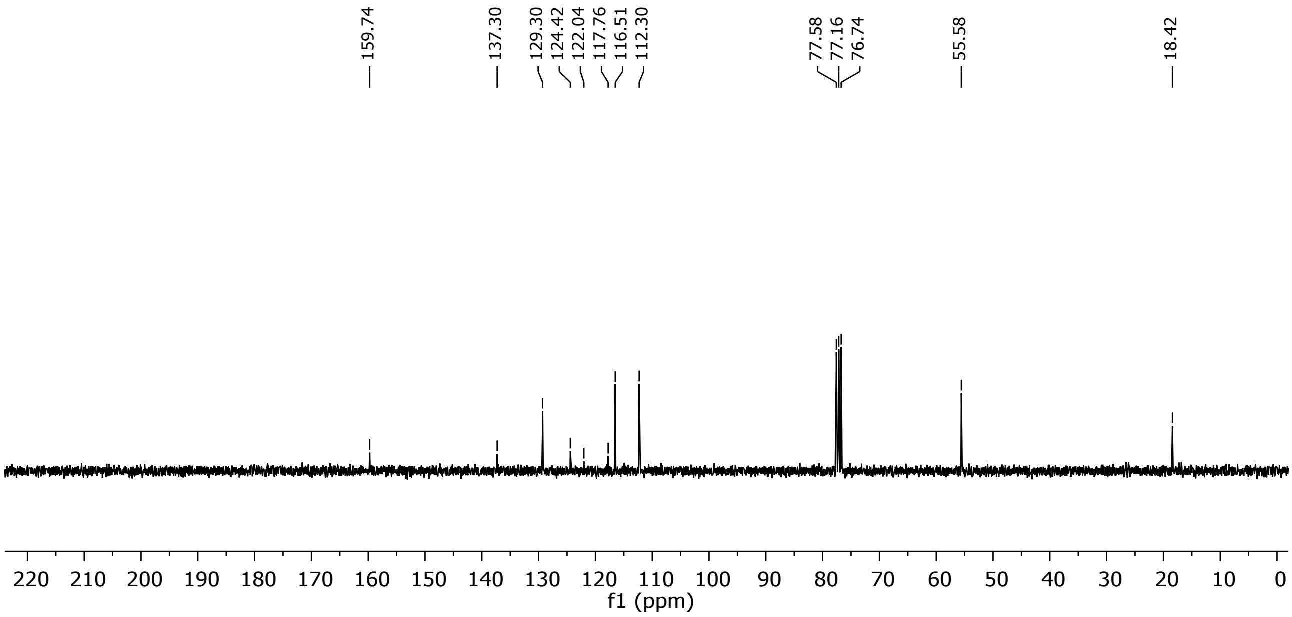

^{1}H NMR (300 MHz, CDCl$_3$)

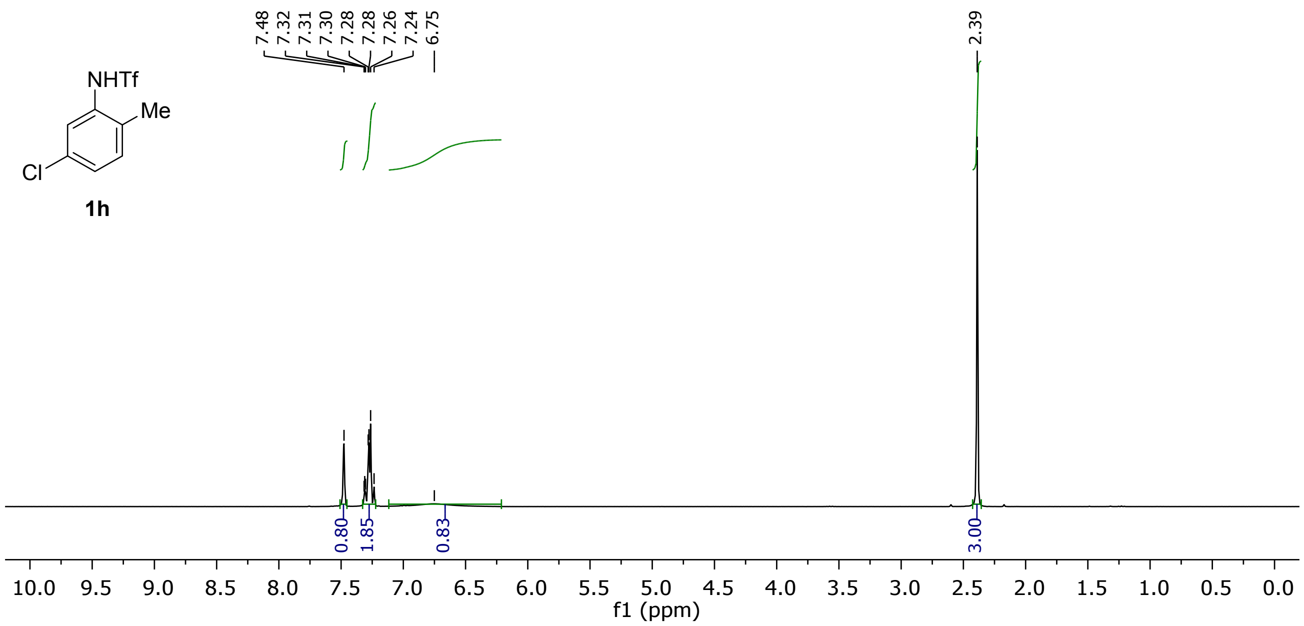

DEPT-135

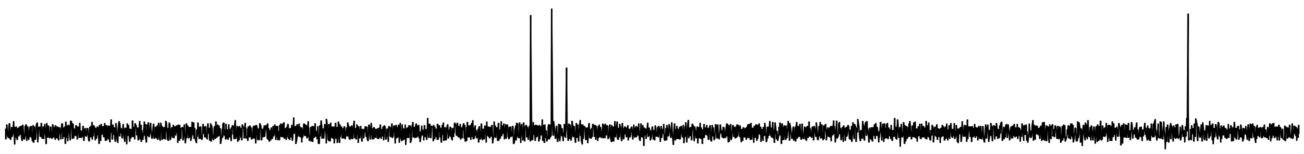

^{13}C NMR (75 MHz, CDCl$_3$)

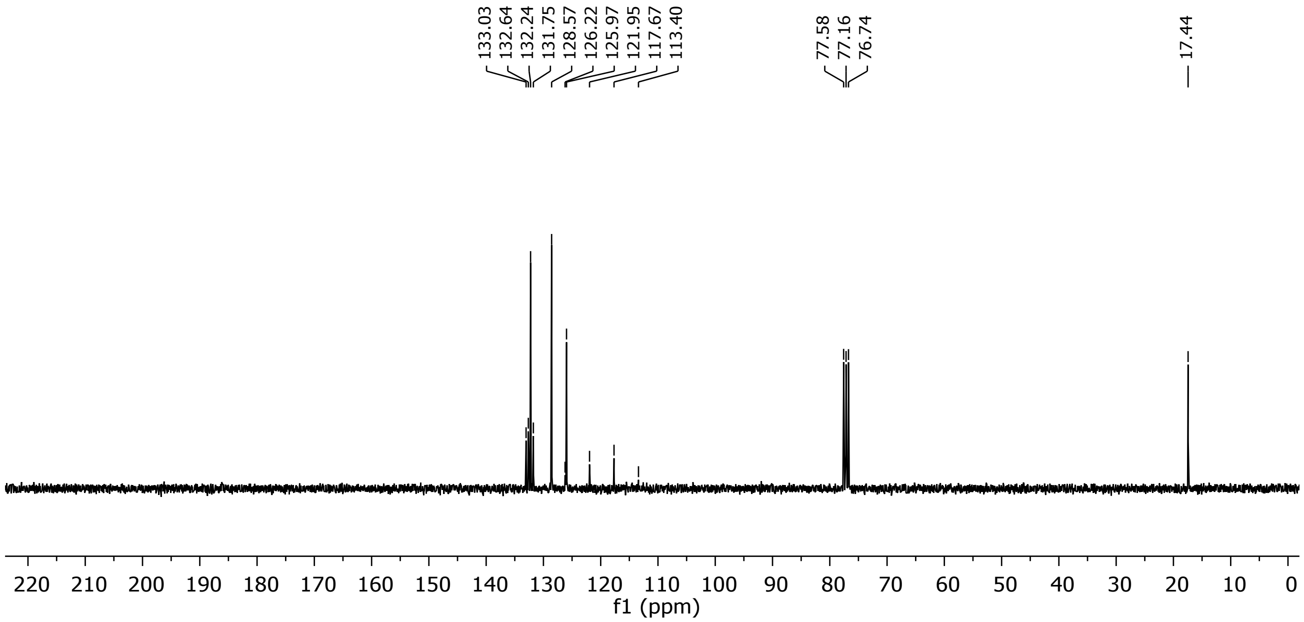

^1H NMR (300 MHz, $CDCl_3$)

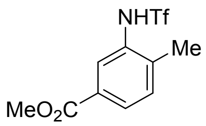

1i

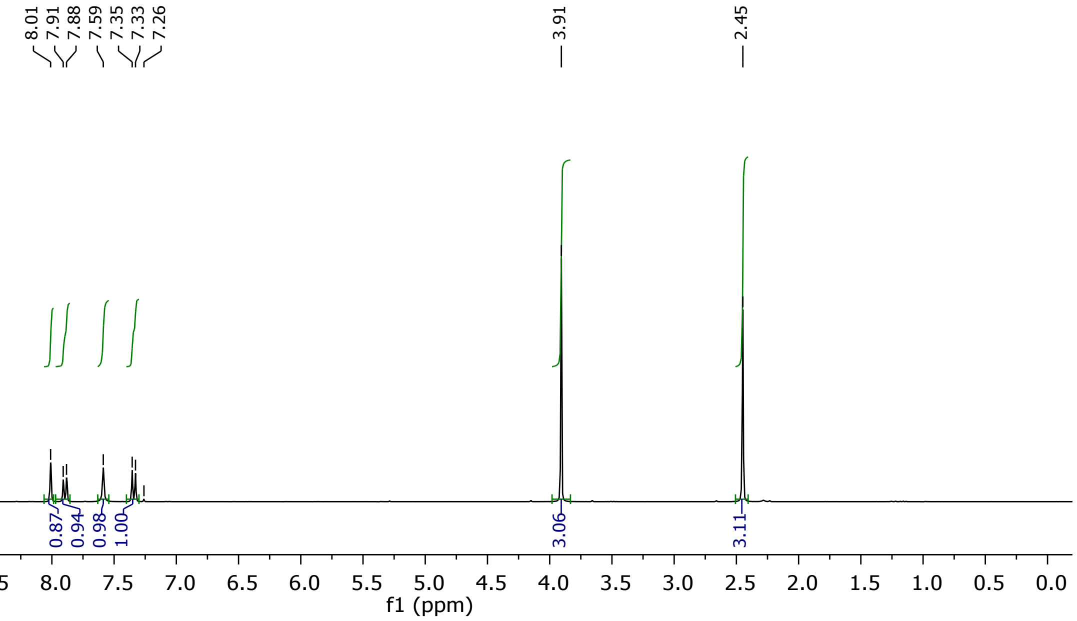

DEPT-135

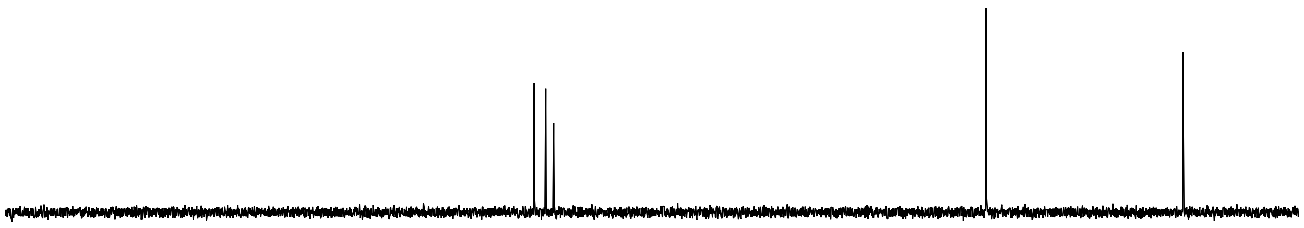

^{13}C NMR (75 MHz, $CDCl_3$)

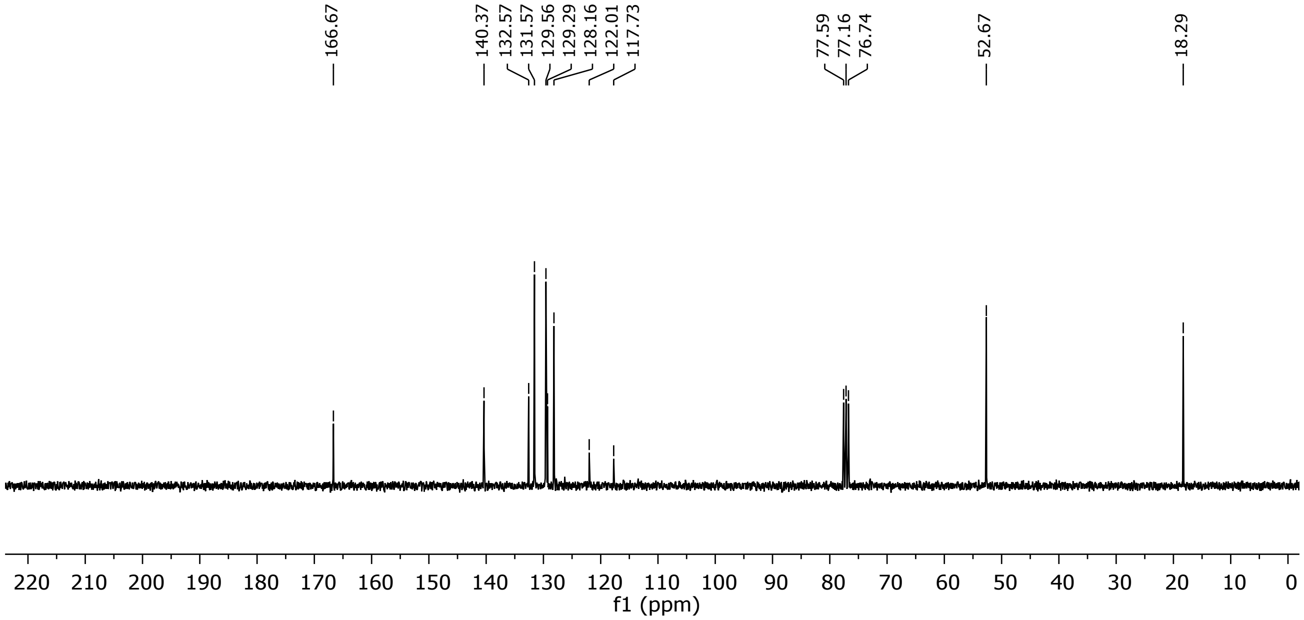

^{1}H NMR (300 MHz, $CDCl_3$)

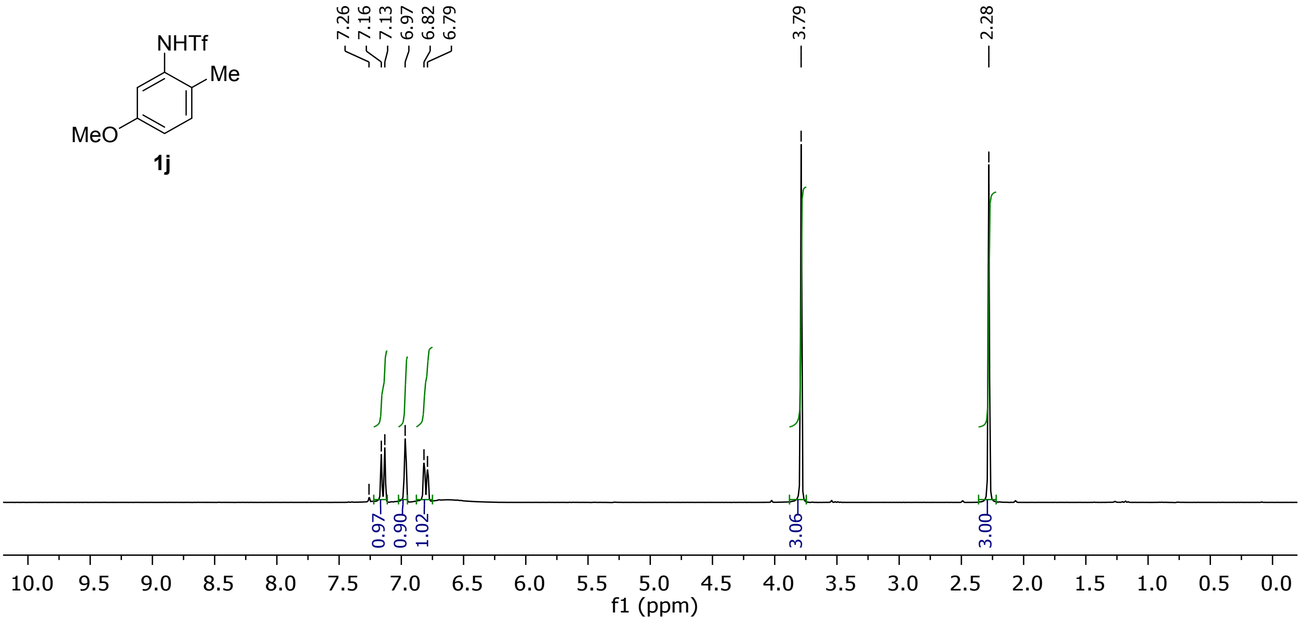

DEPT-135

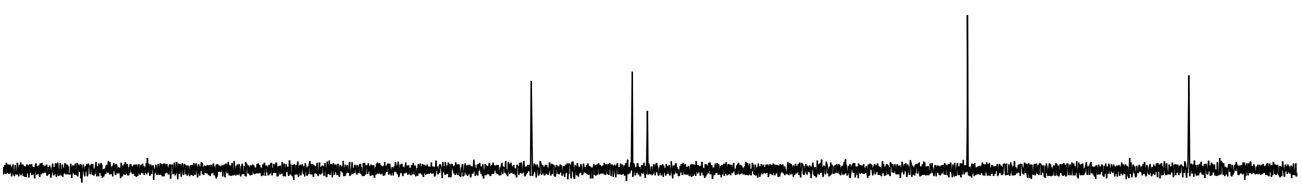

^{13}C NMR (75 MHz, $CDCl_3$)

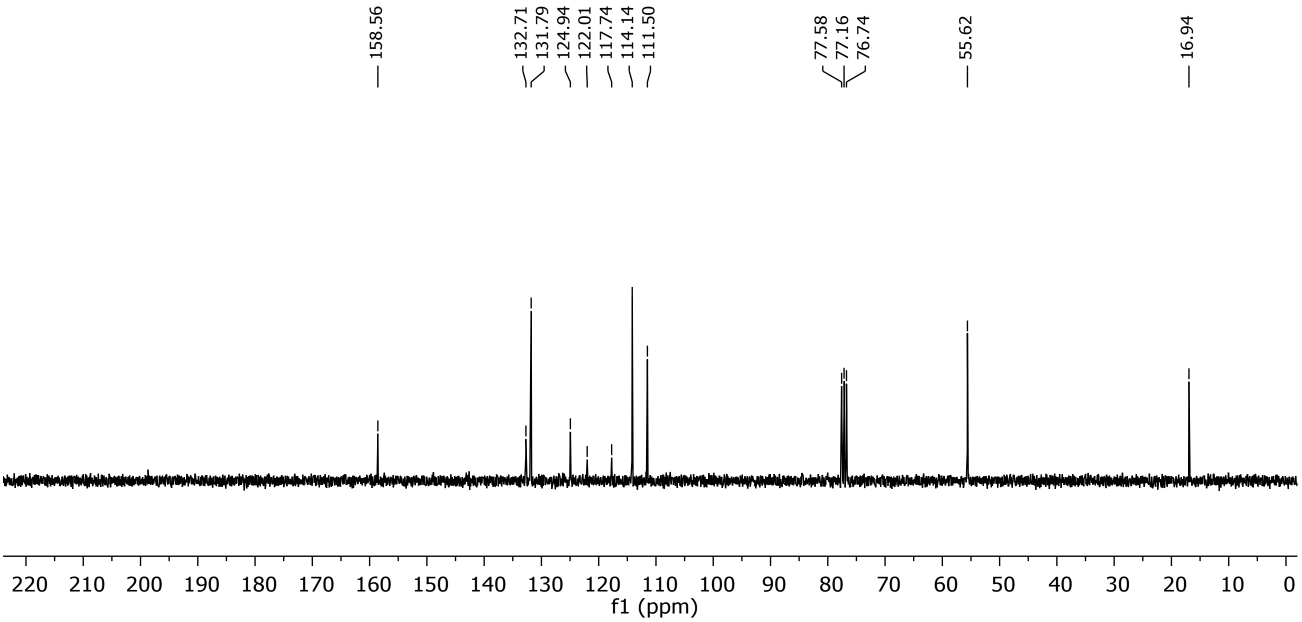

^{1}H NMR (300 MHz, $CDCl_3$)

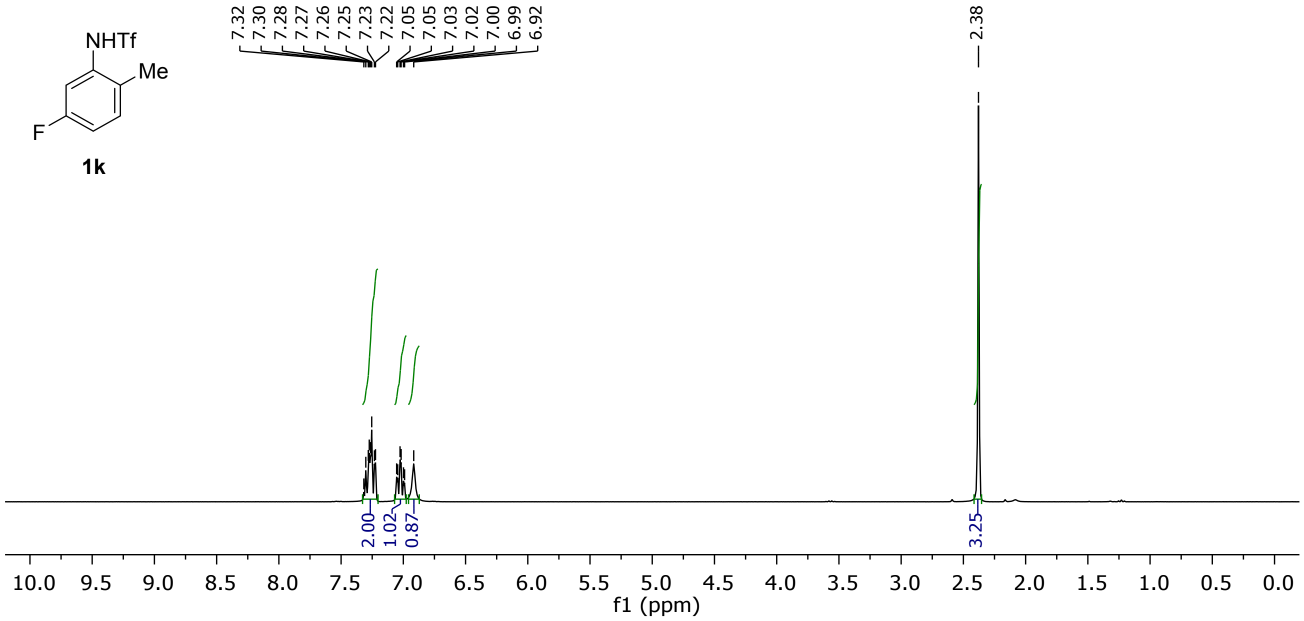

DEPT-135

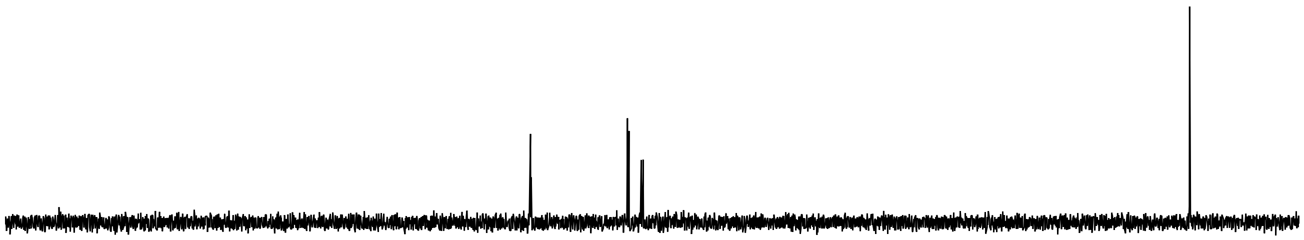

^{13}C NMR (75 MHz, $CDCl_3$)

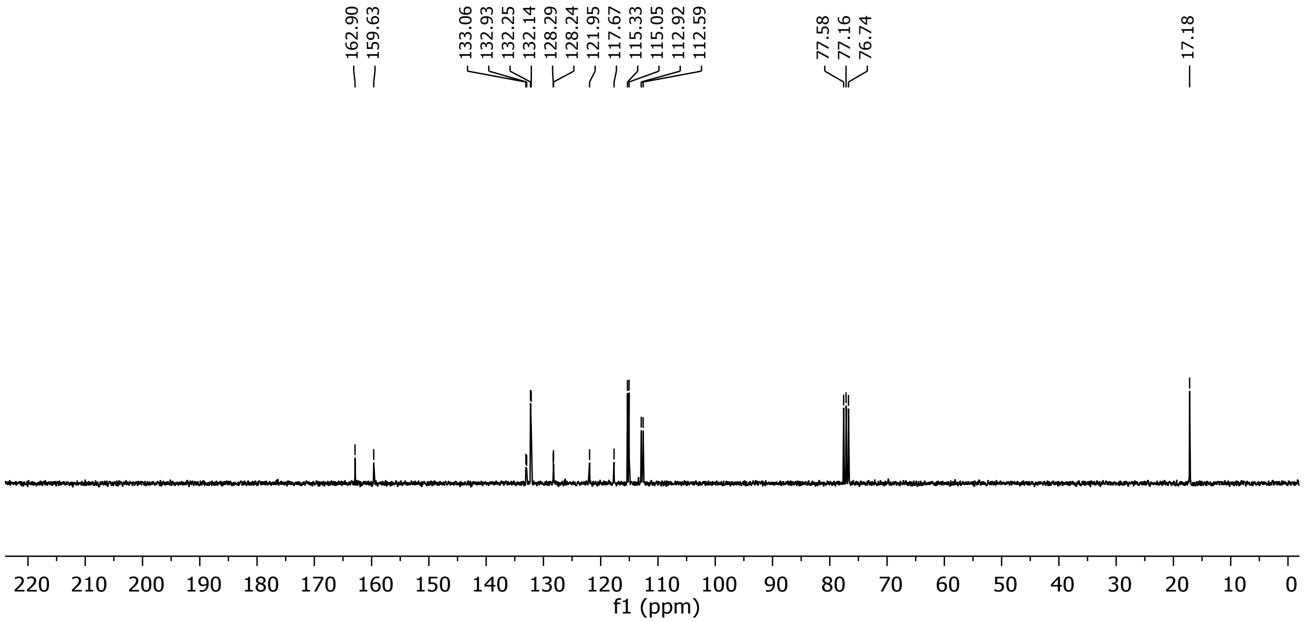

^{1}H NMR (300 MHz, $CDCl_3$)

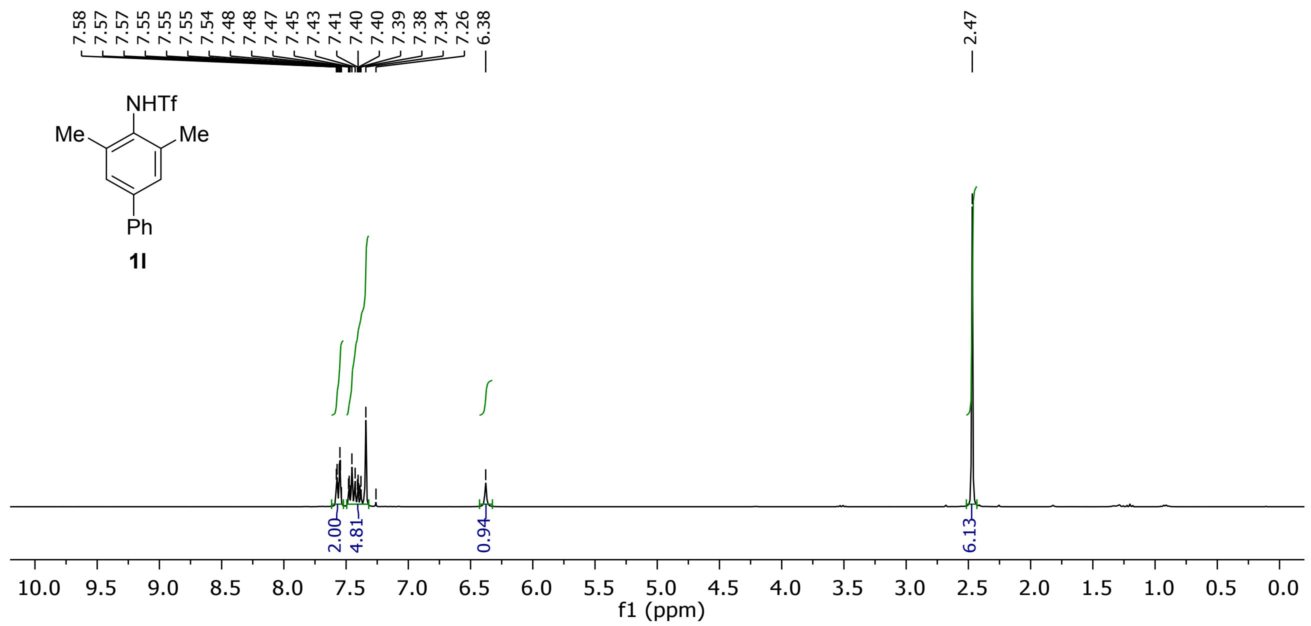

DEPT-135

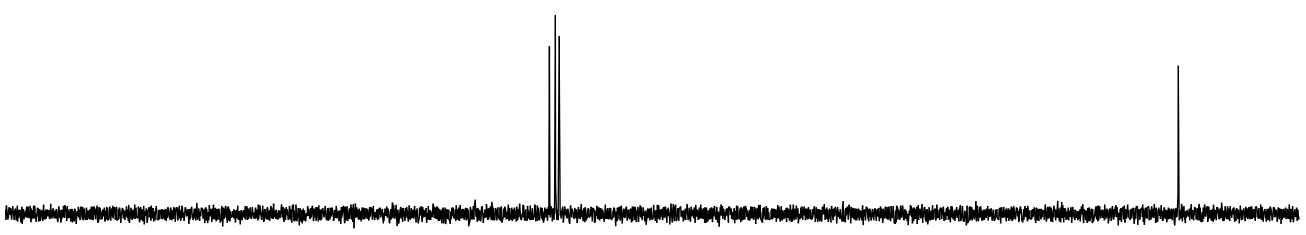

^{13}C NMR (75 MHz, $CDCl_3$)

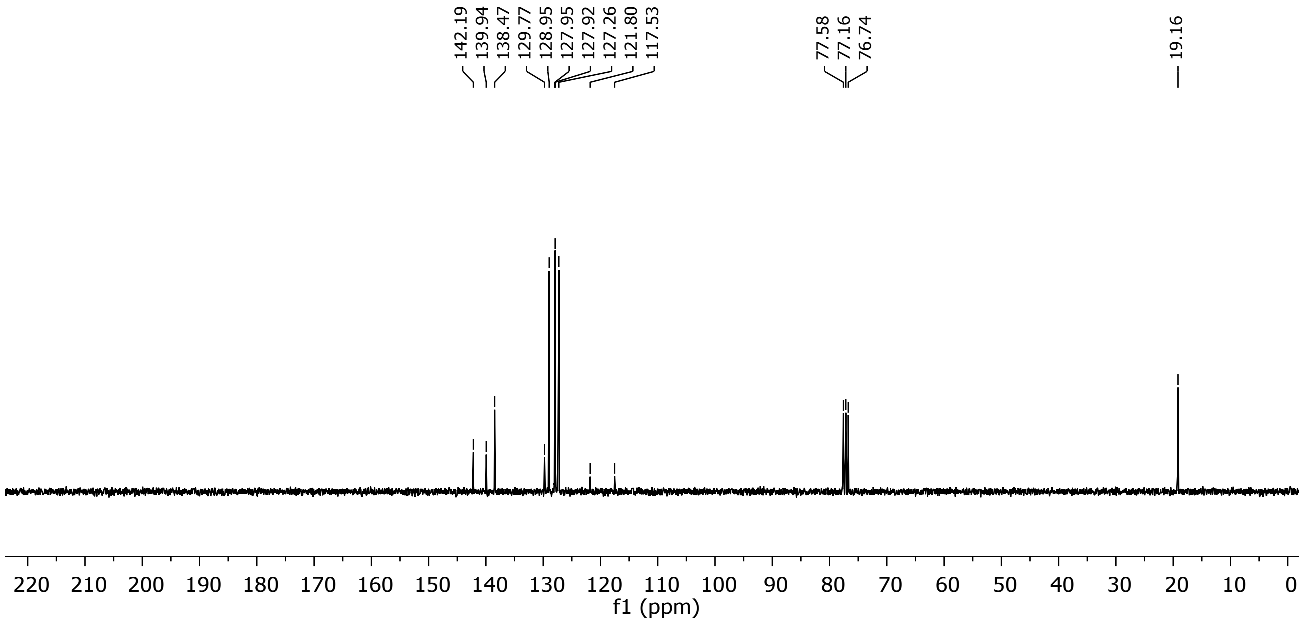

^{1}H NMR (300 MHz, $CDCl_3$)

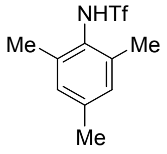

1m

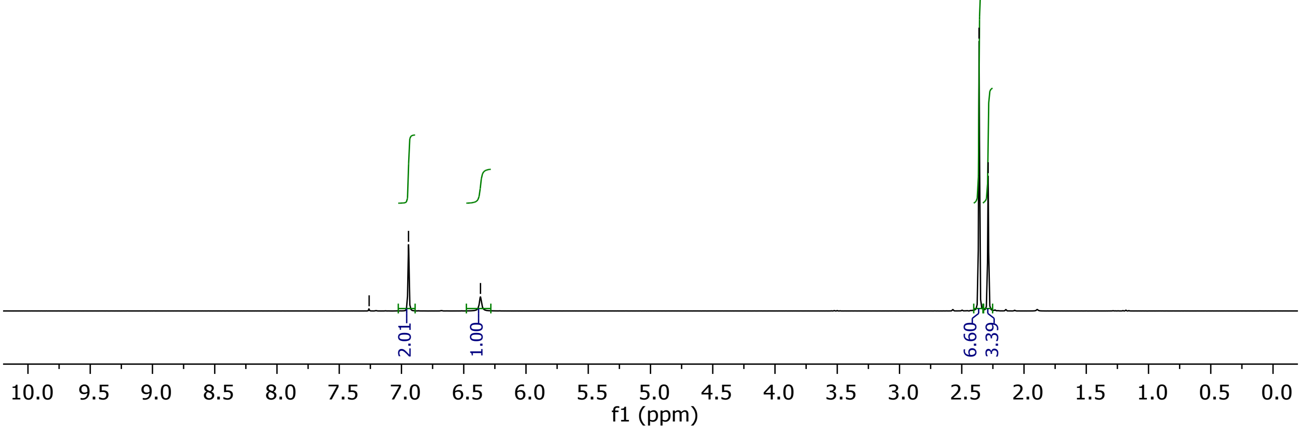

DEPT-135

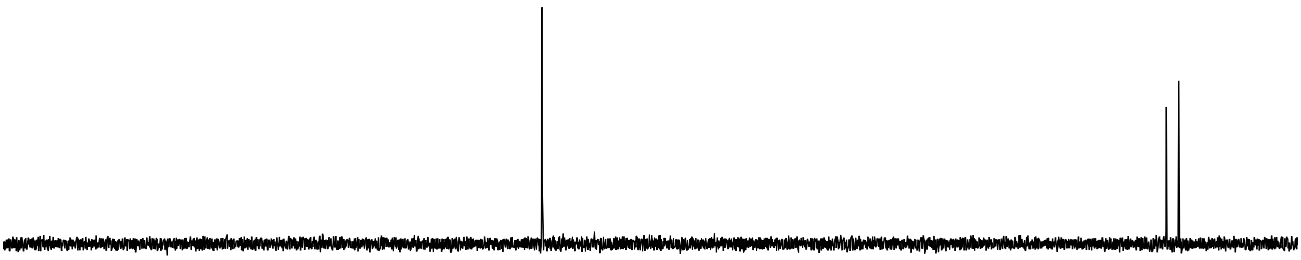

^{13}C NMR (75 MHz, $CDCl_3$)

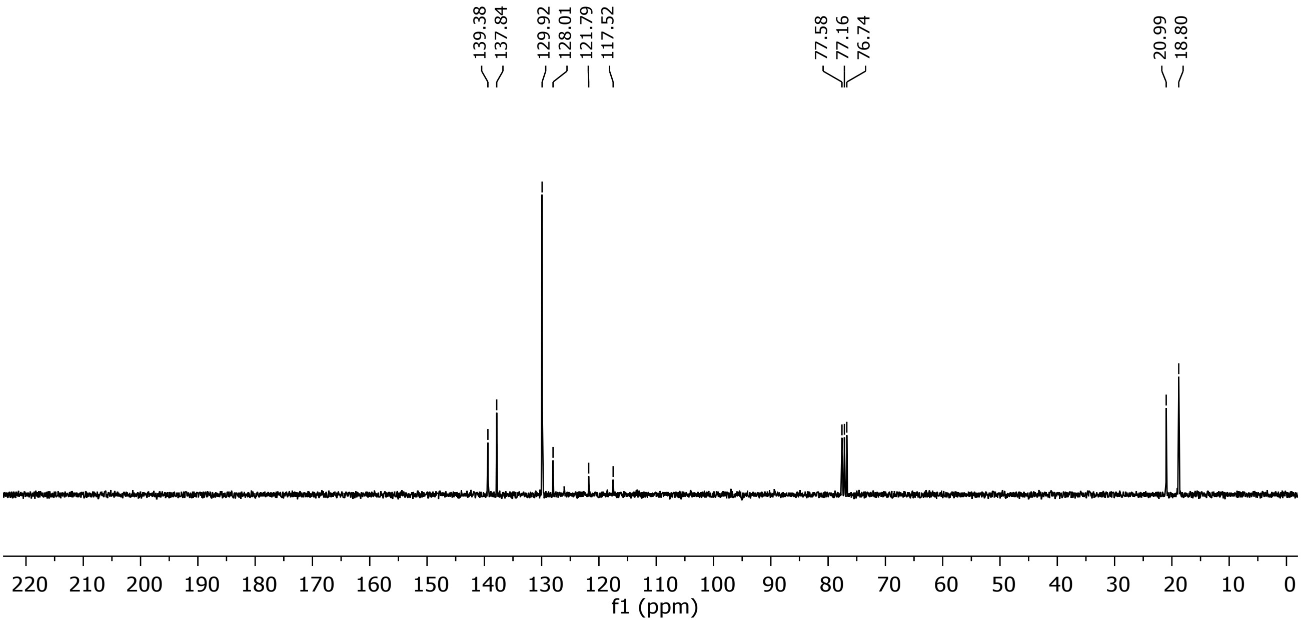

^{1}H NMR (300 MHz, $CDCl_3$)

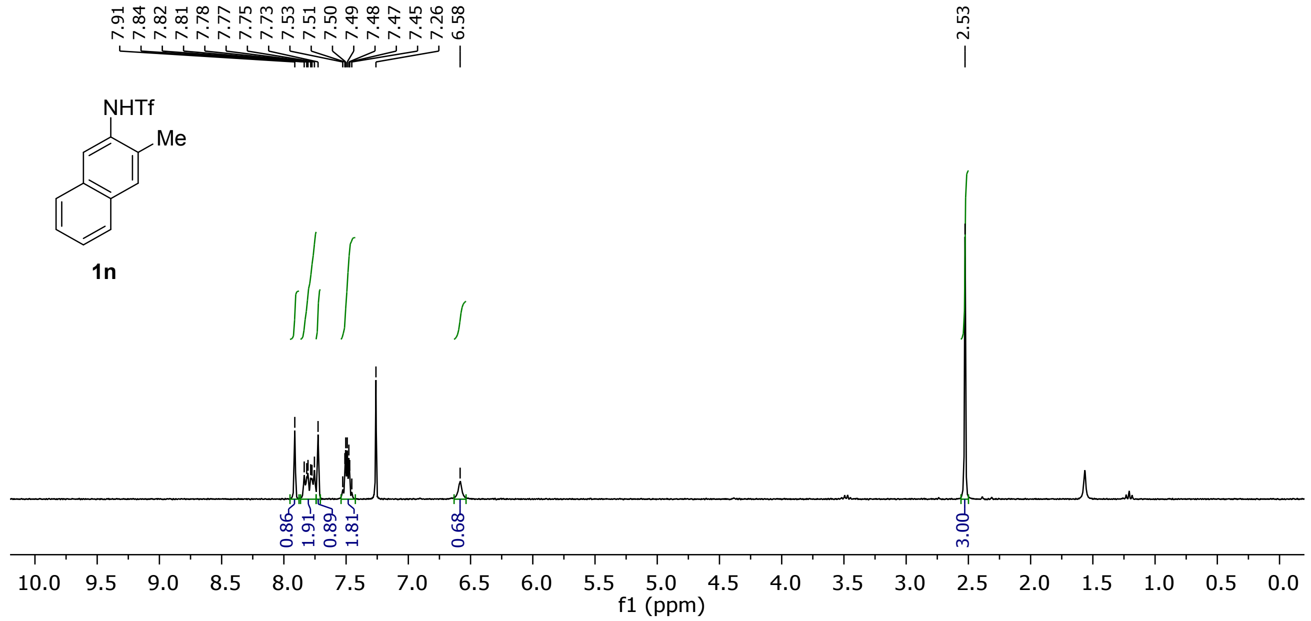

DEPT-135

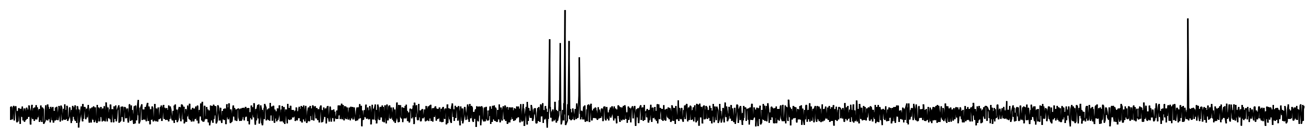

^{13}C NMR (75 MHz, $CDCl_3$)

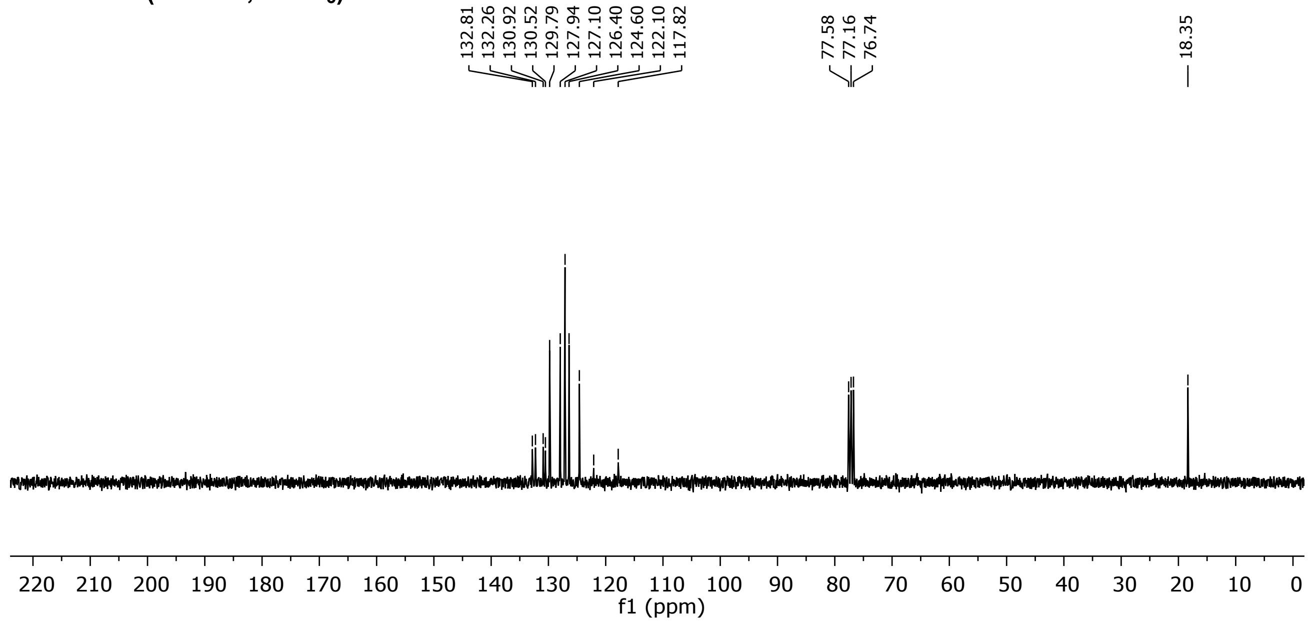

^{1}H NMR (300 MHz, $CDCl_3$)

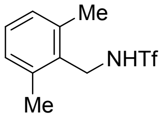

4a

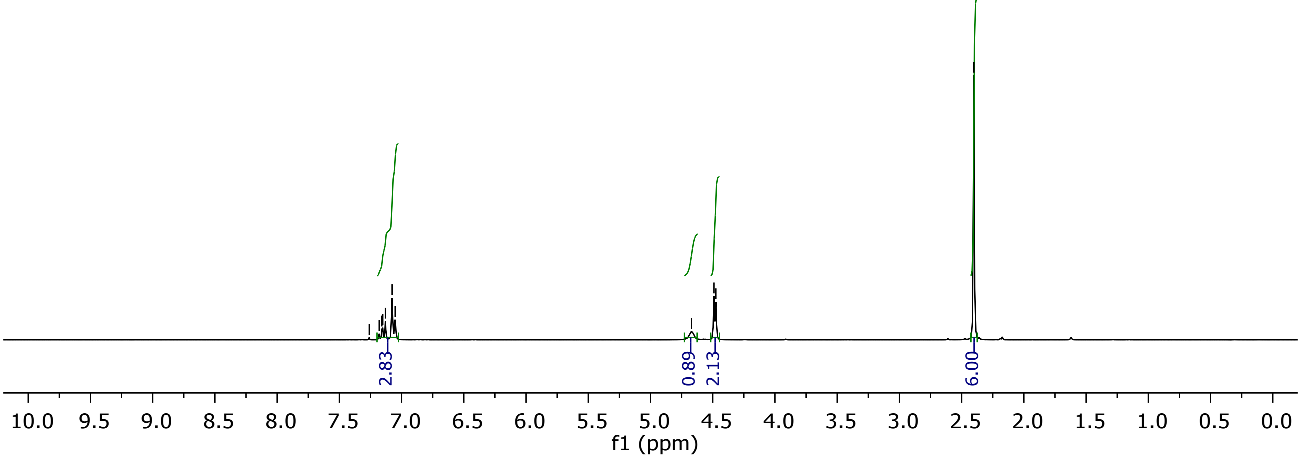

DEPT-135

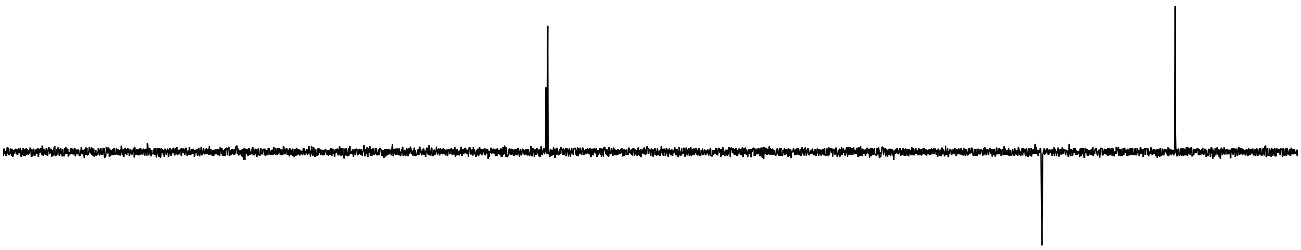

^{13}C NMR (75 MHz, $CDCl_3$)

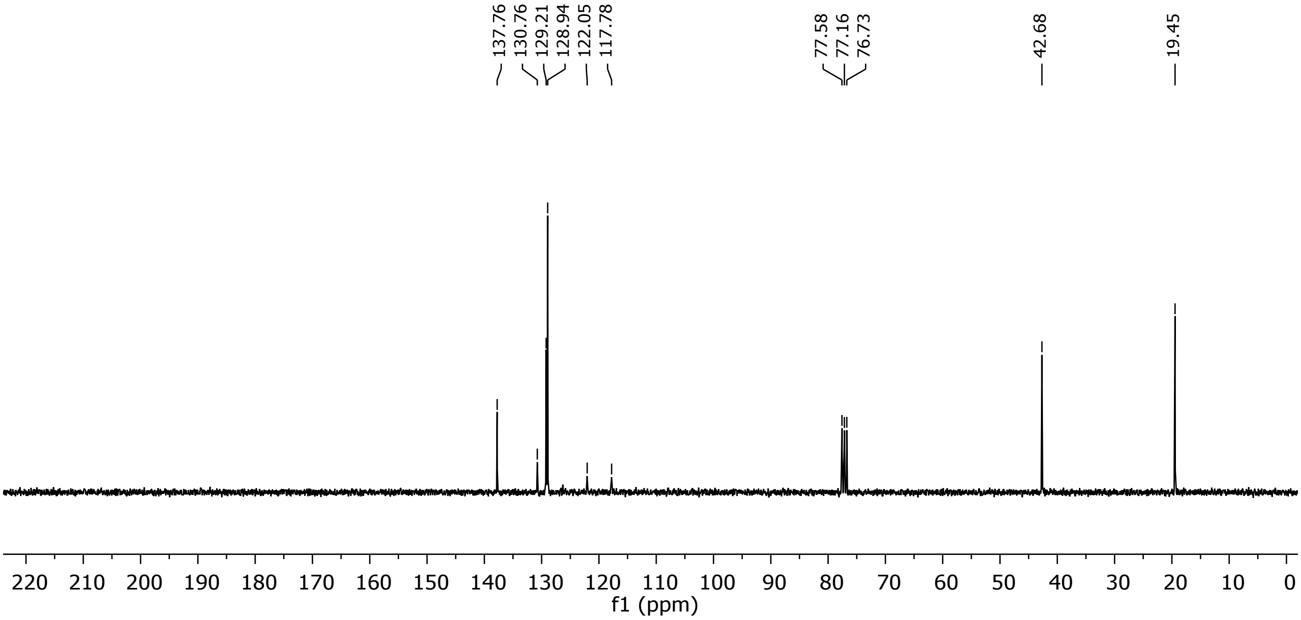

^{1}H NMR (300 MHz, $CDCl_3$)

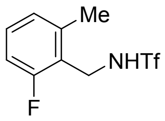

4b

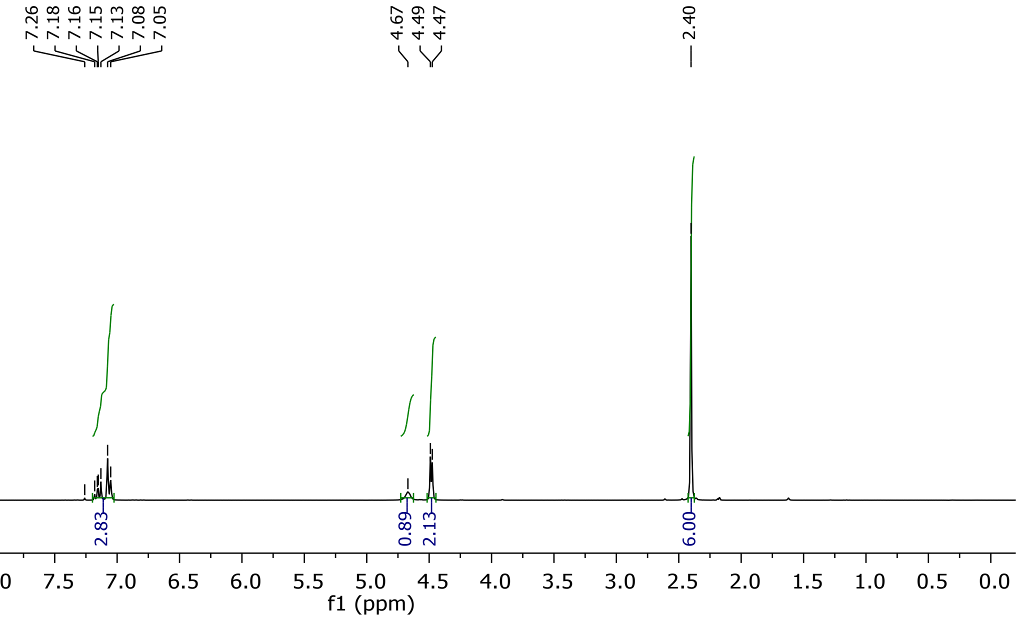

DEPT-135

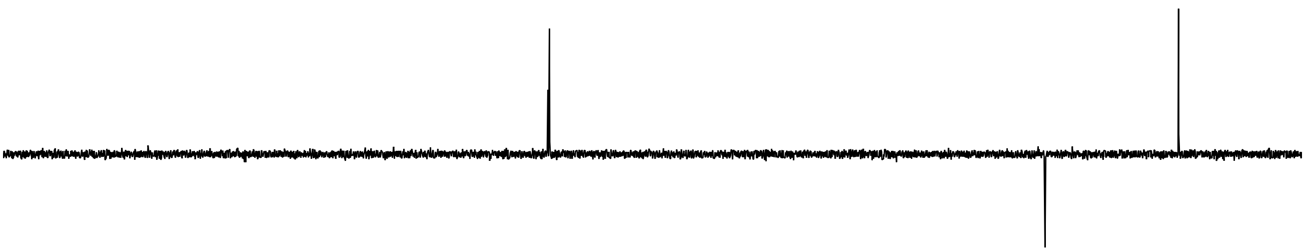

^{13}C NMR (75 MHz, $CDCl_3$)

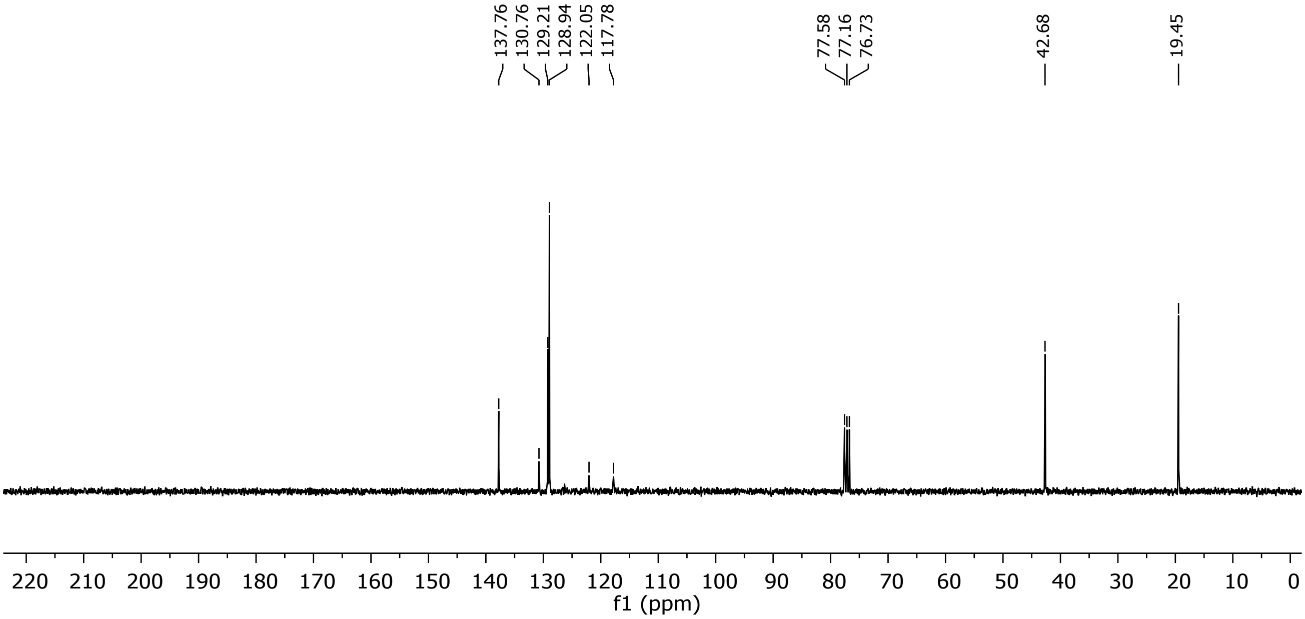

^{1}H NMR (300 MHz, $CDCl_3$)

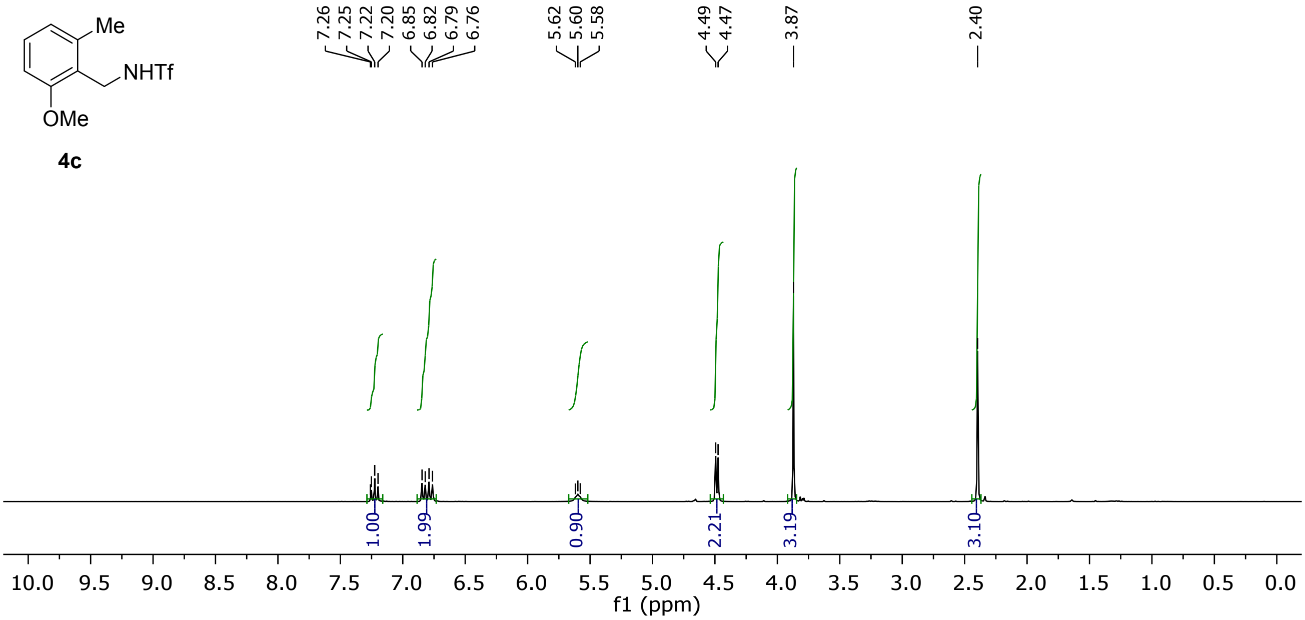

DEPT-135

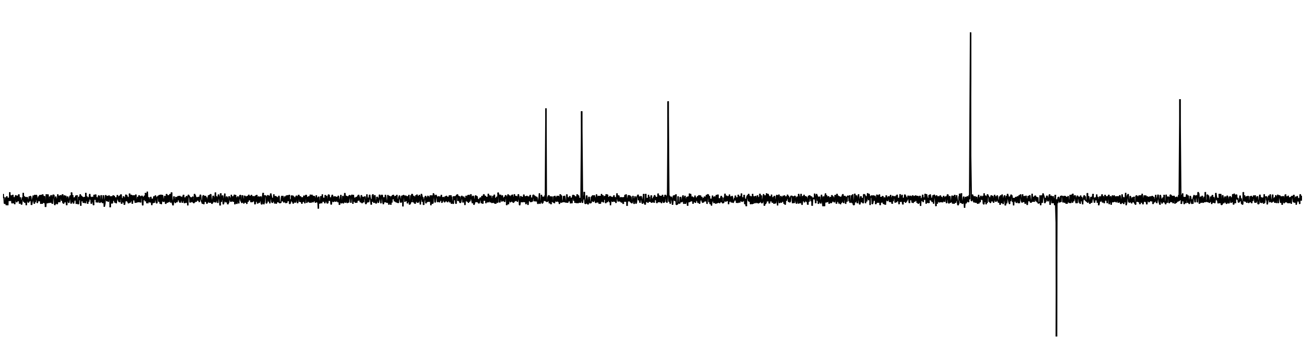

^{13}C NMR (75 MHz, $CDCl_3$)

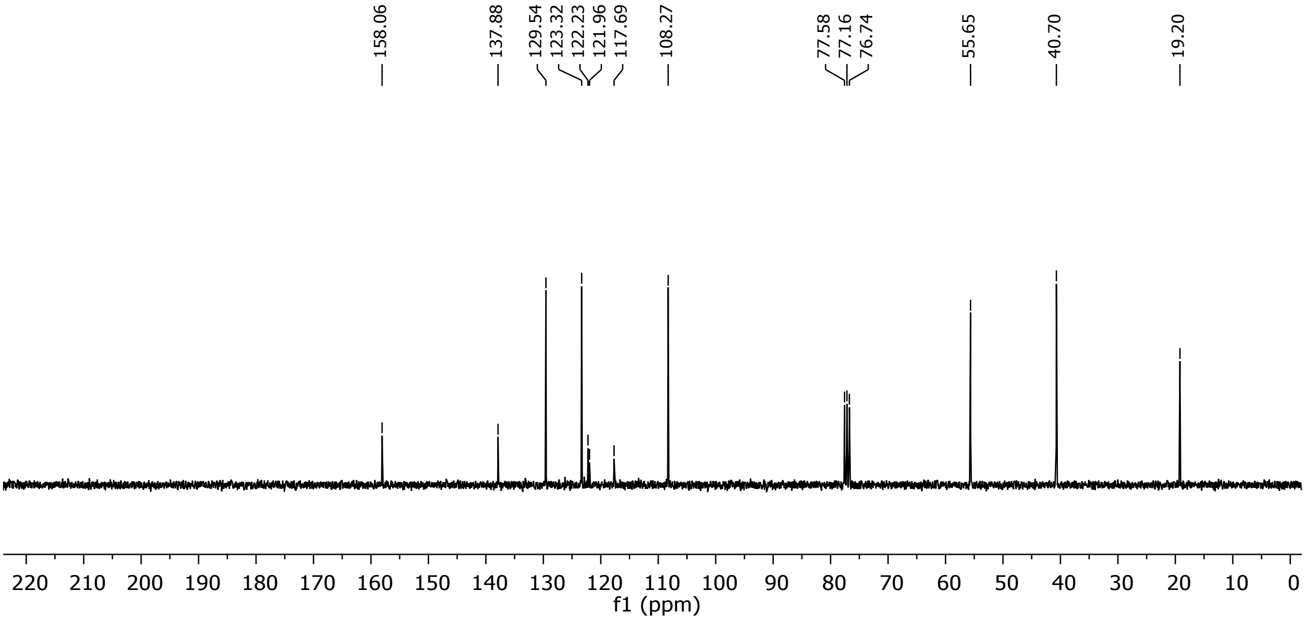

^{1}H NMR (300 MHz, $CDCl_3$)

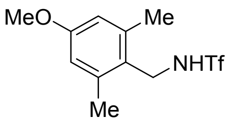

4d

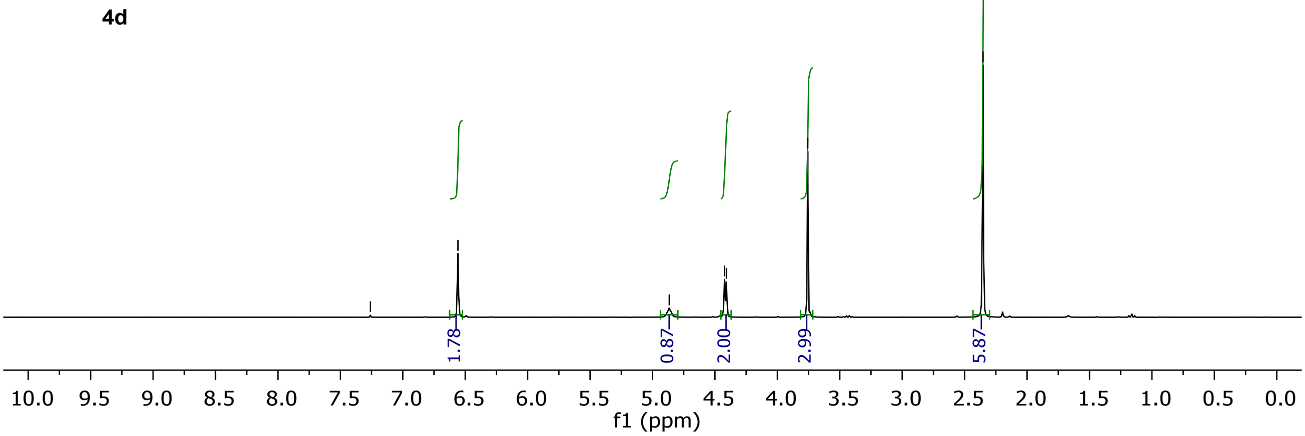

DEPT-135

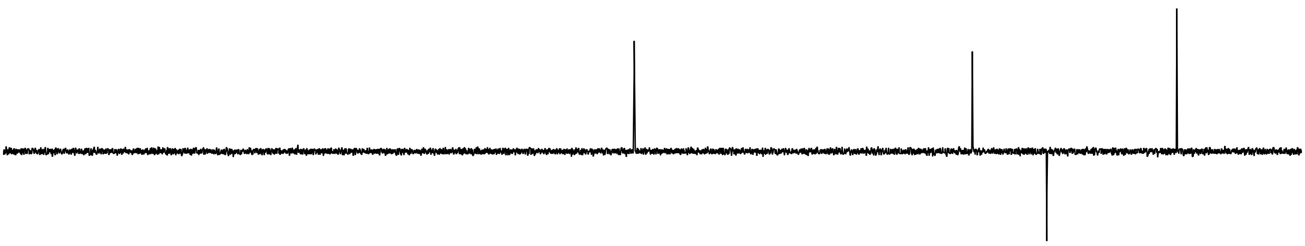

^{13}C NMR (75 MHz, $CDCl_3$)

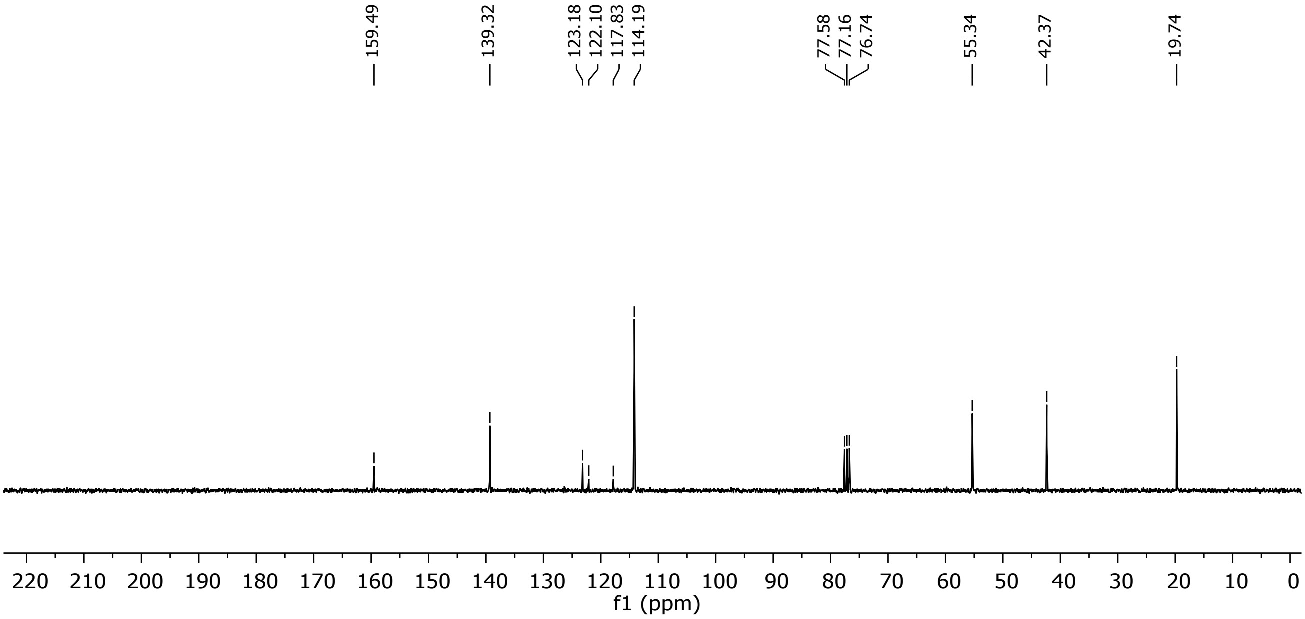

^{1}H NMR (300 MHz, $CDCl_3$)

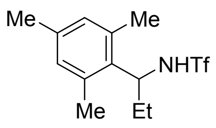

4e

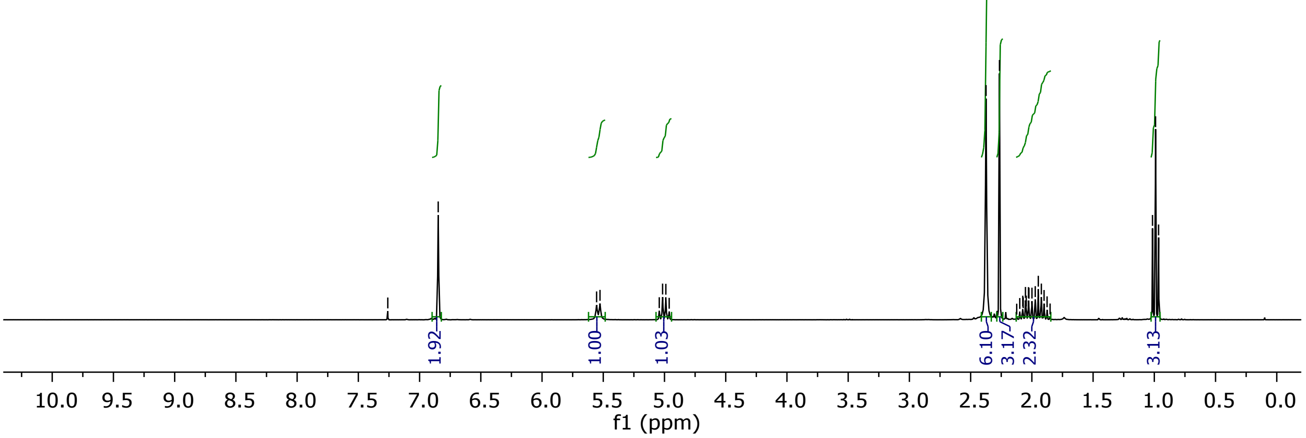

DEPT-135

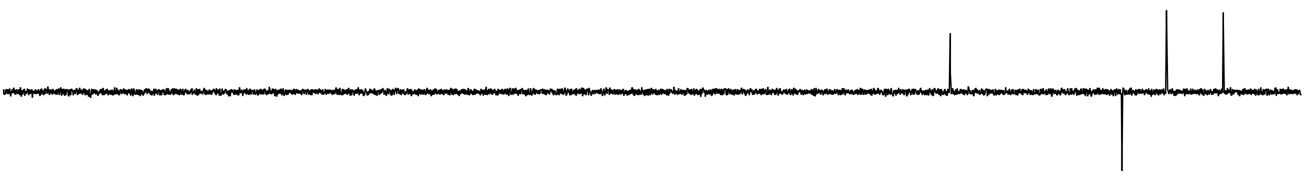

^{13}C NMR (75 MHz, $CDCl_3$)

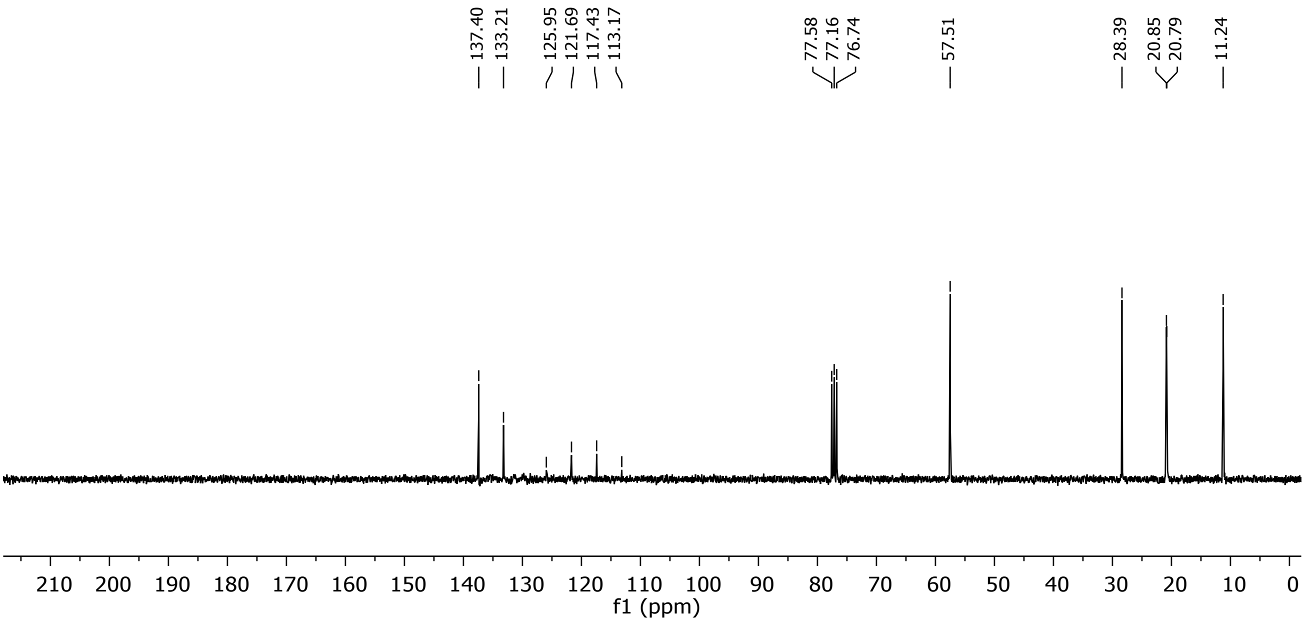

^{1}H NMR (300 MHz, CDCl$_3$)

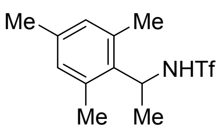

4f

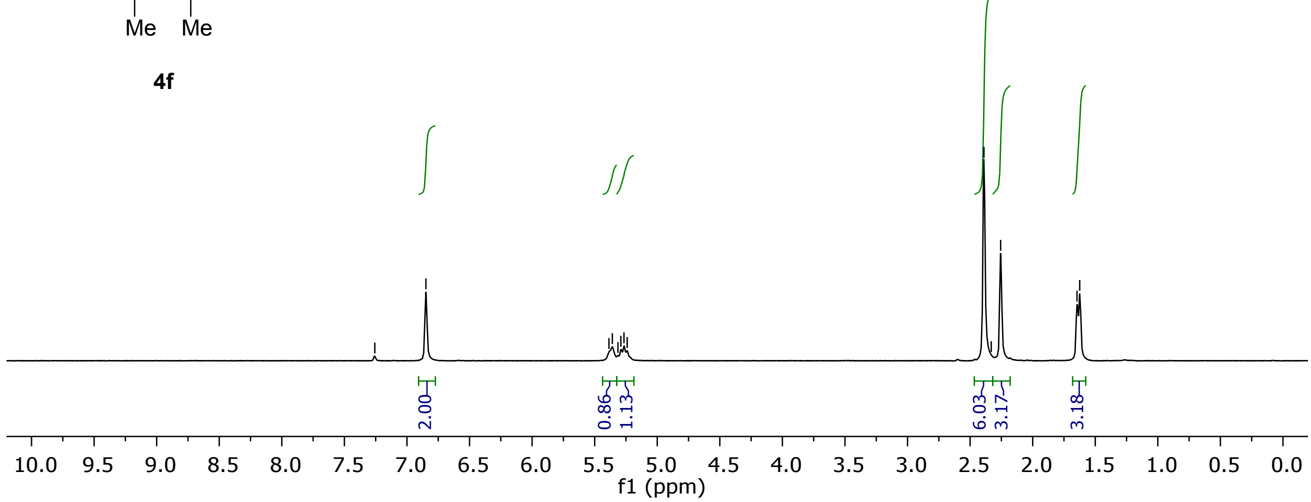

DEPT-135

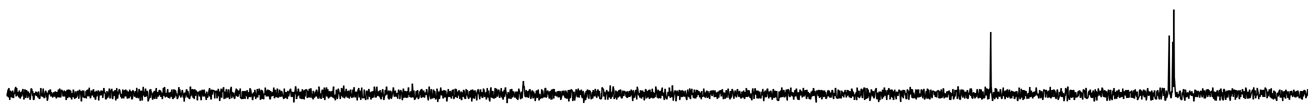

^{13}C NMR (75 MHz, CDCl$_3$)

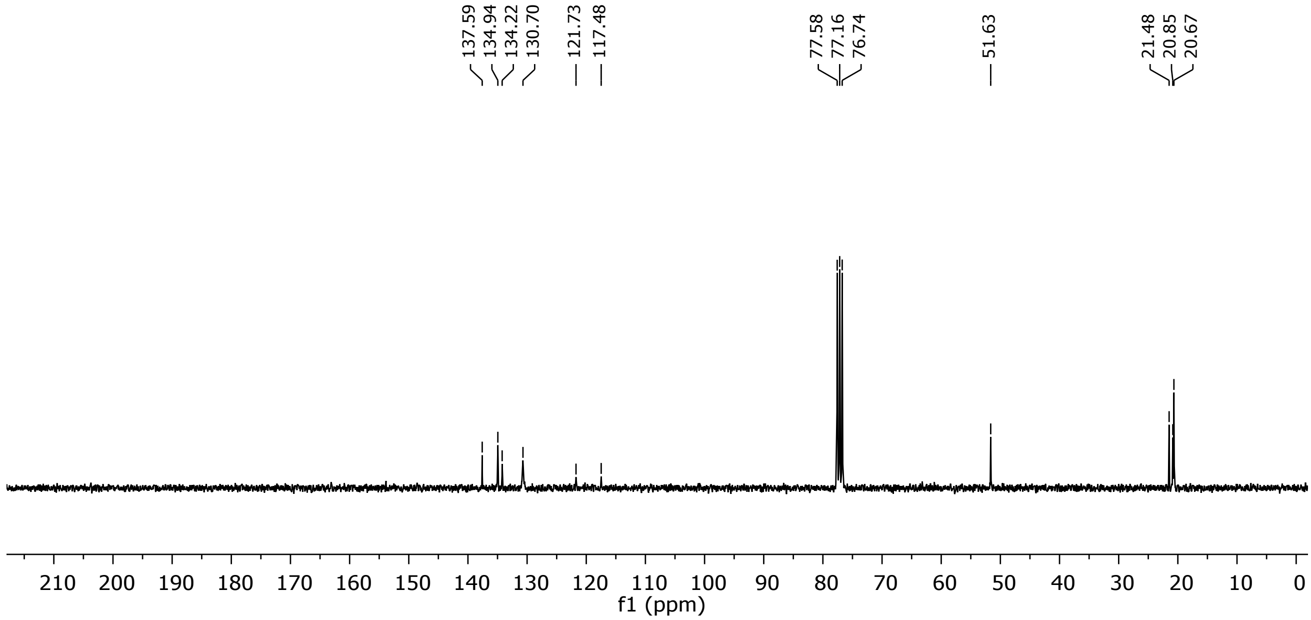

^{1}H NMR (300 MHz, CDCl$_3$)

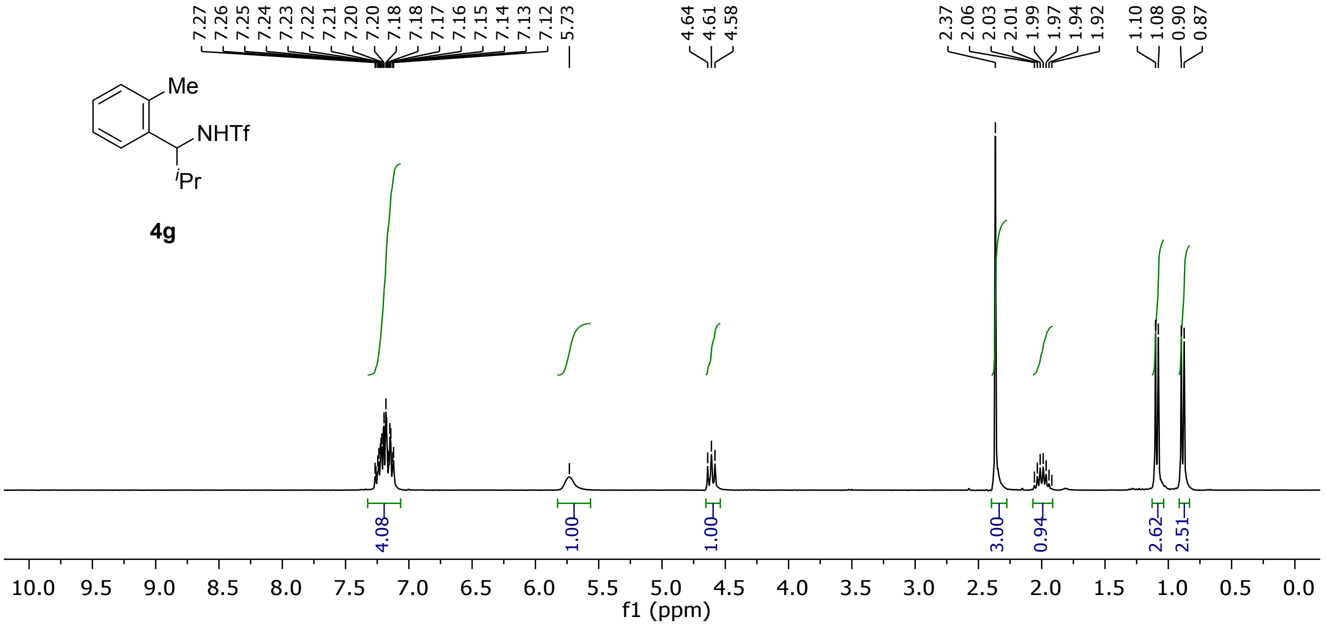

4g

DEPT-135

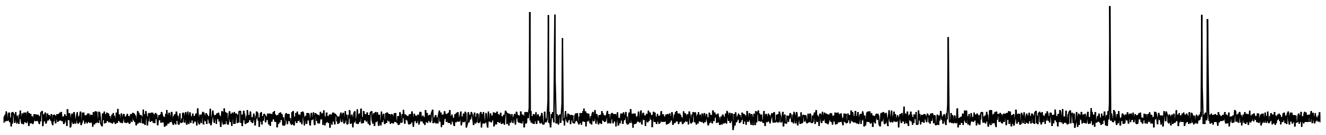

^{13}C NMR (75 MHz, CDCl$_3$)

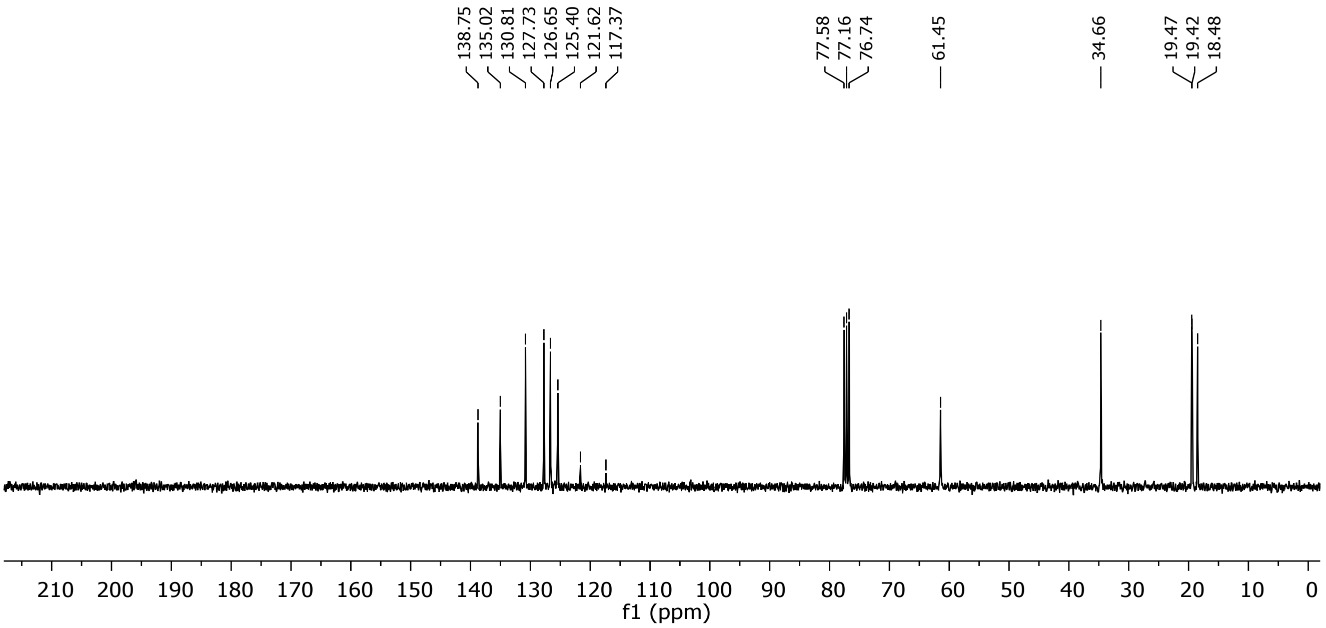

^{1}H NMR (300 MHz, CDCl$_3$)

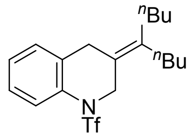

3aa

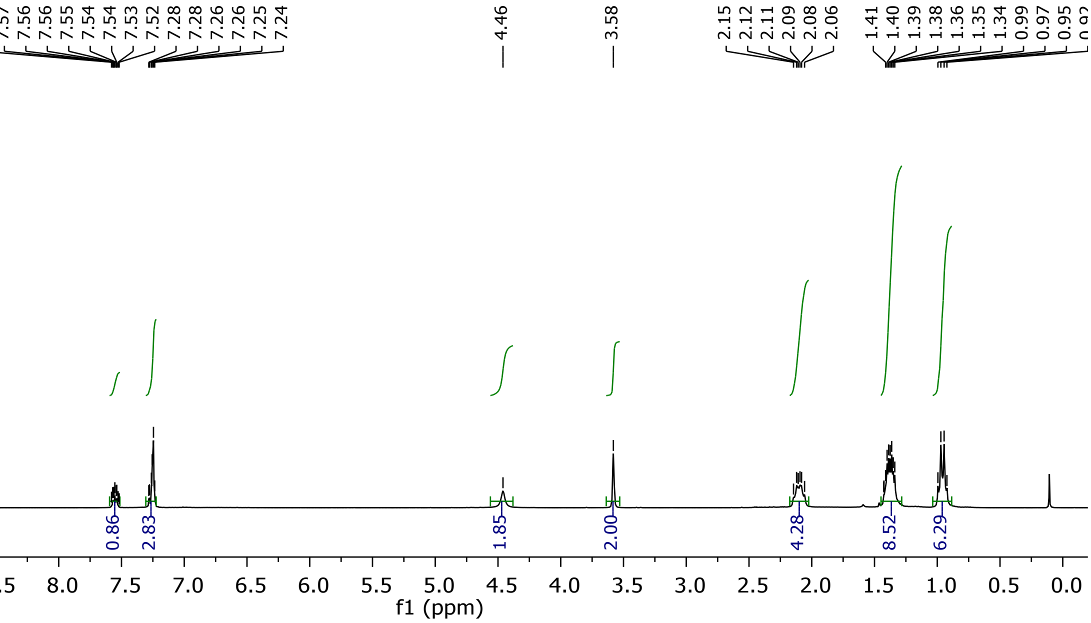

DEPT-135

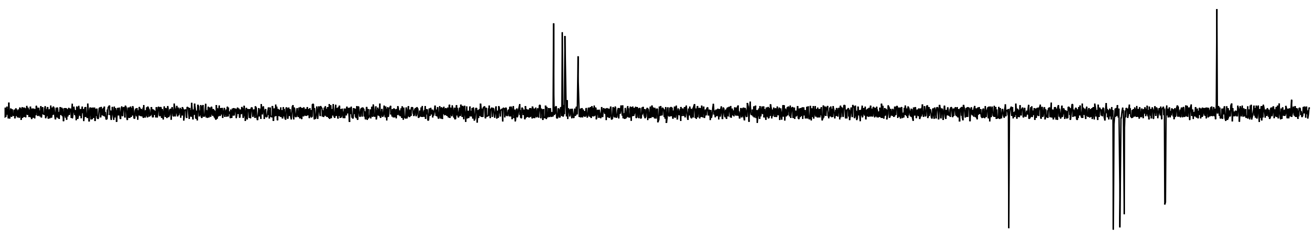

^{13}C NMR (75 MHz, CDCl$_3$)

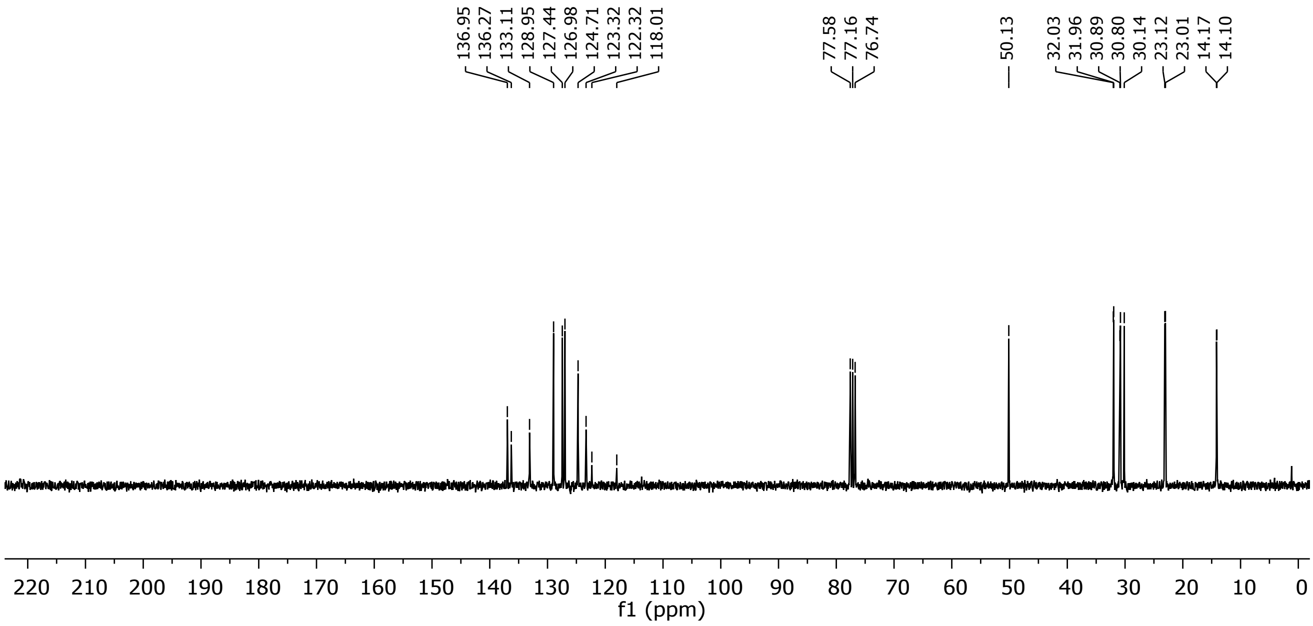

¹H NMR (300 MHz, $CDCl_3$)

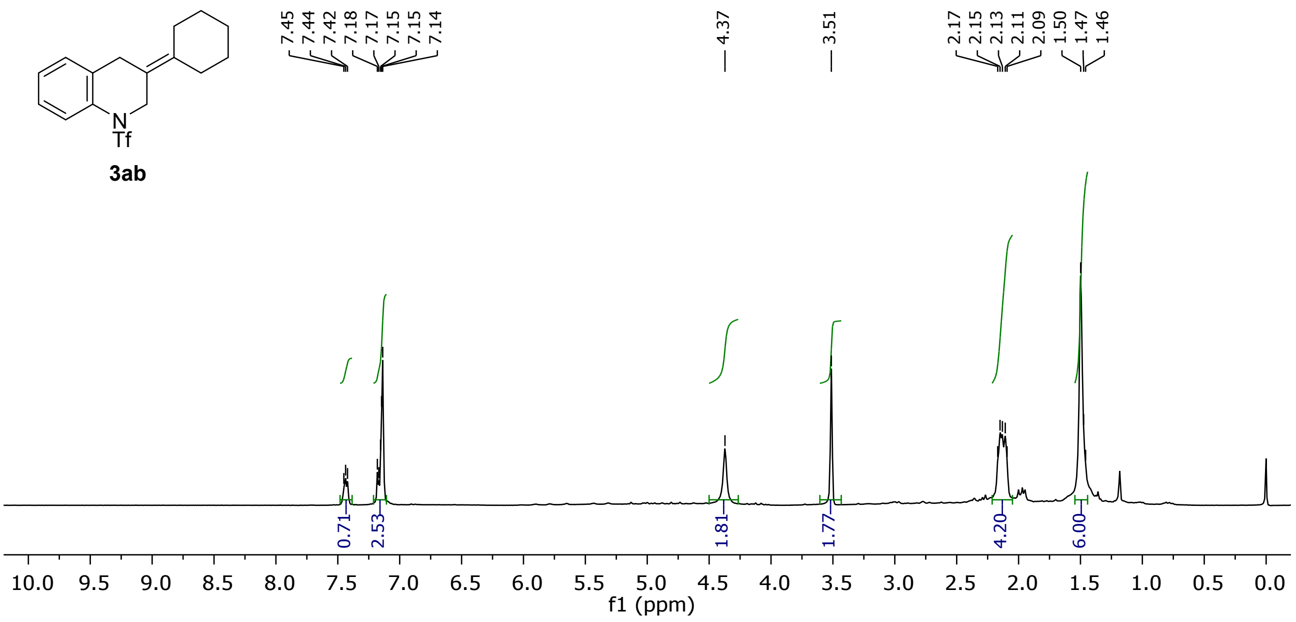

DEPT-135

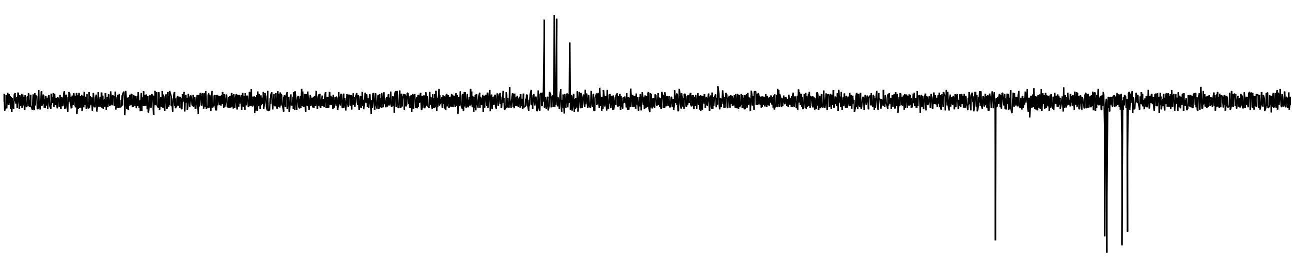

¹³C NMR (75 MHz, $CDCl_3$)

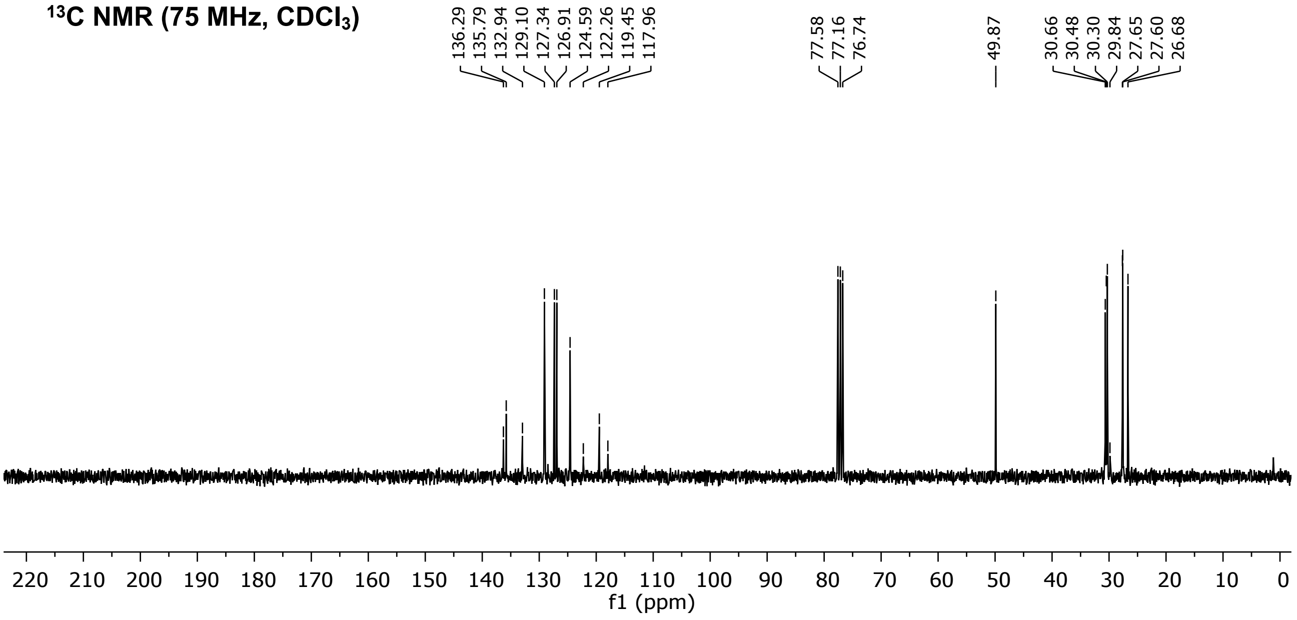

^{1}H NMR (500 MHz, CDCl$_3$)

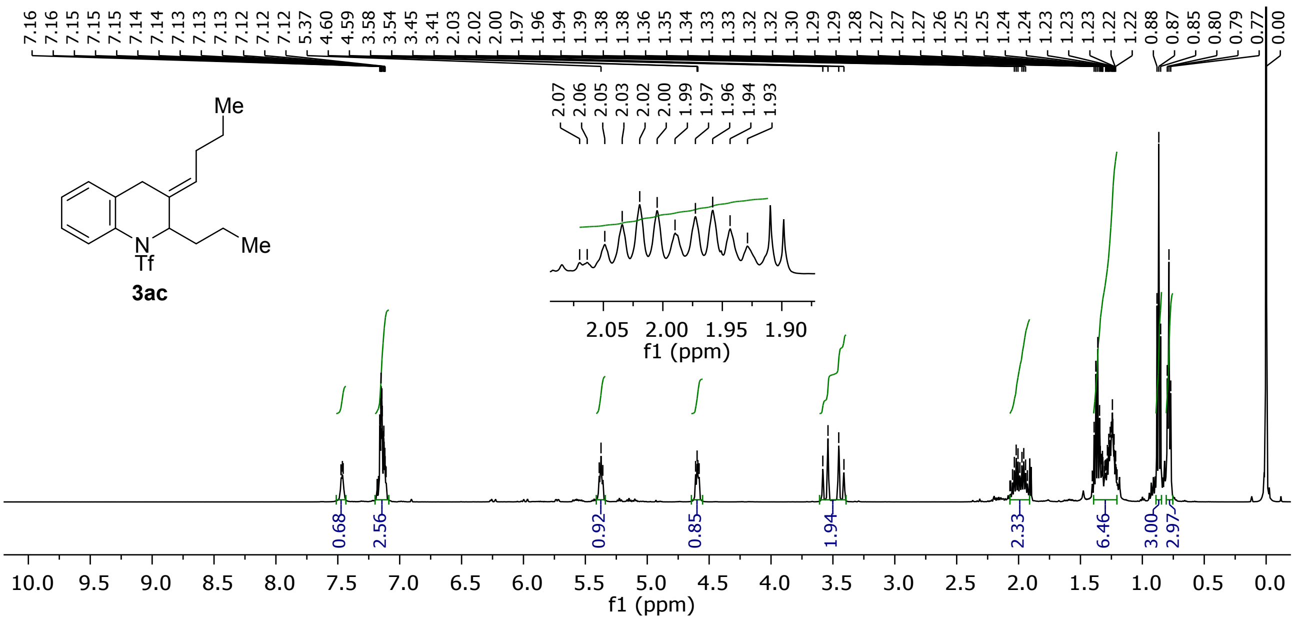

DEPT-135

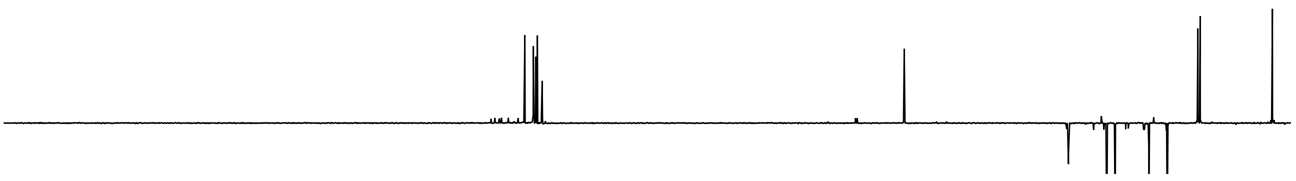

^{13}C NMR (126 MHz, CDCl$_3$)

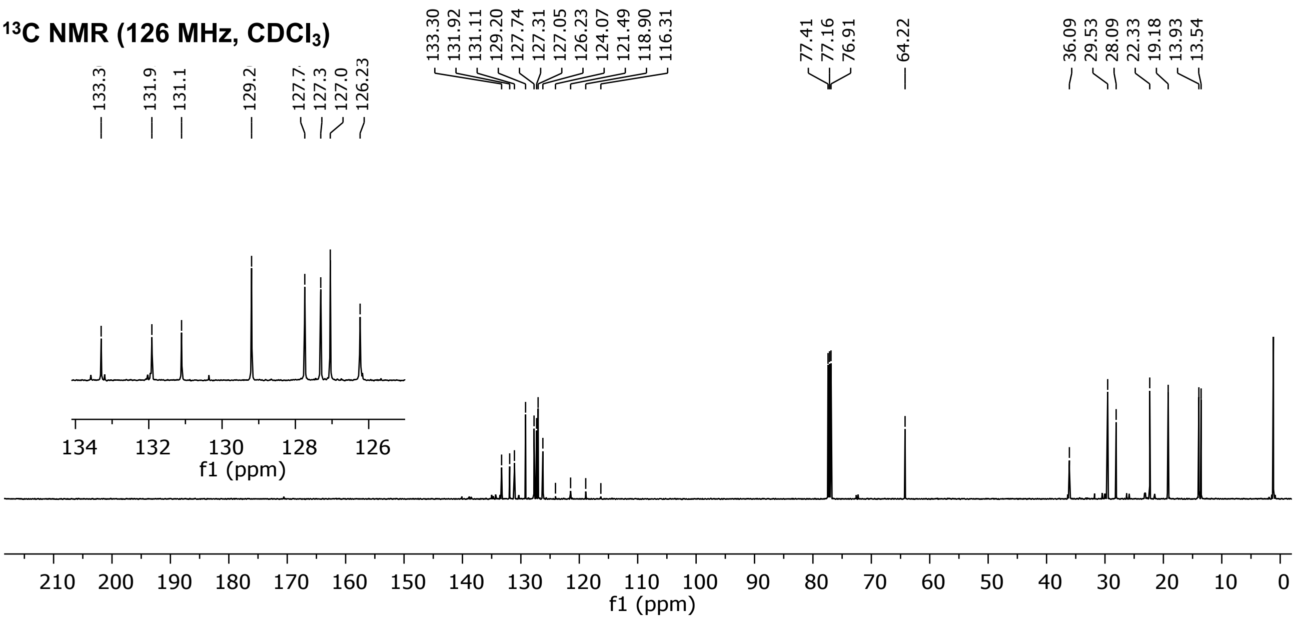

^{1}H NMR (500 MHz, $CDCl_3$)

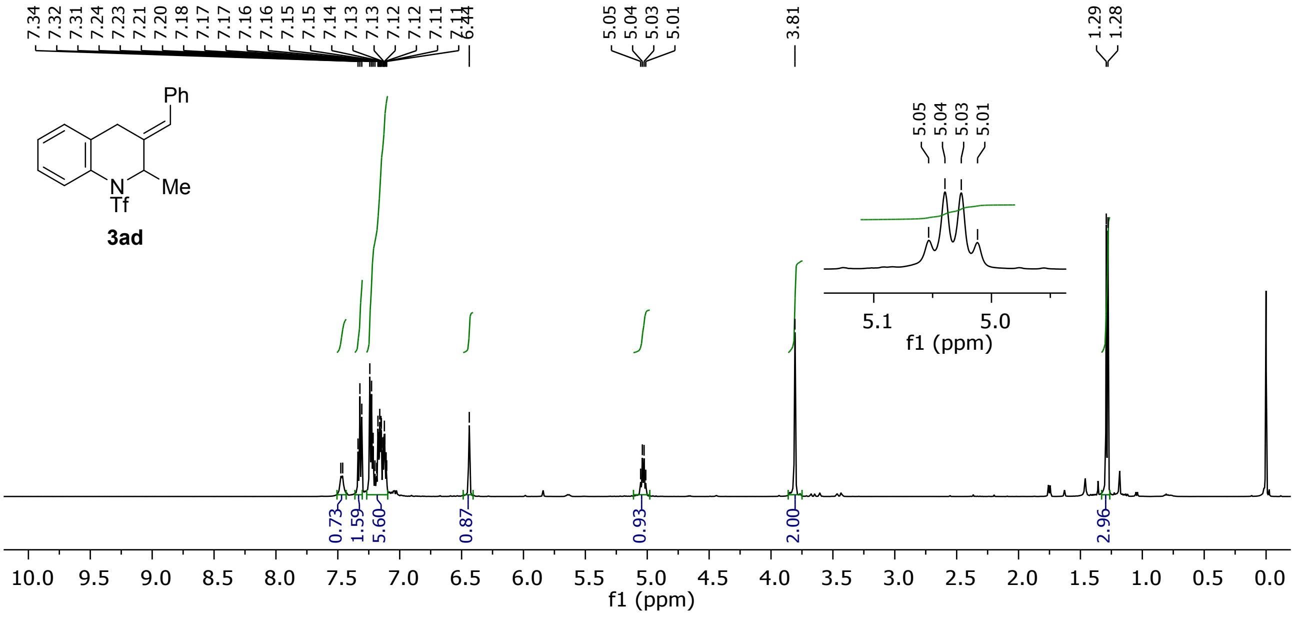

DEPT-135

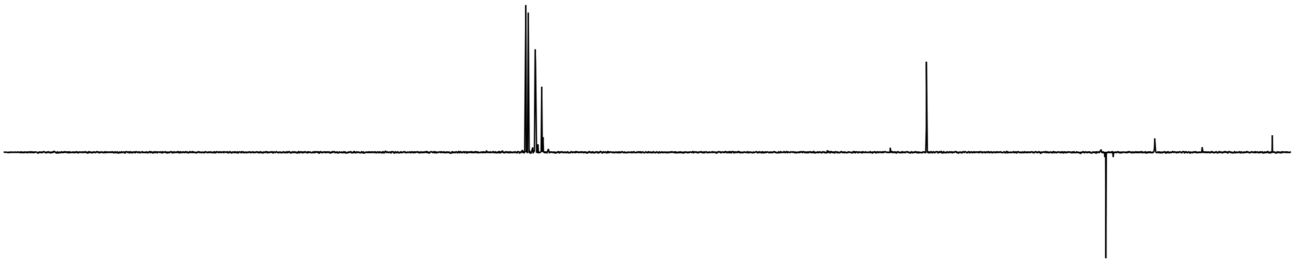

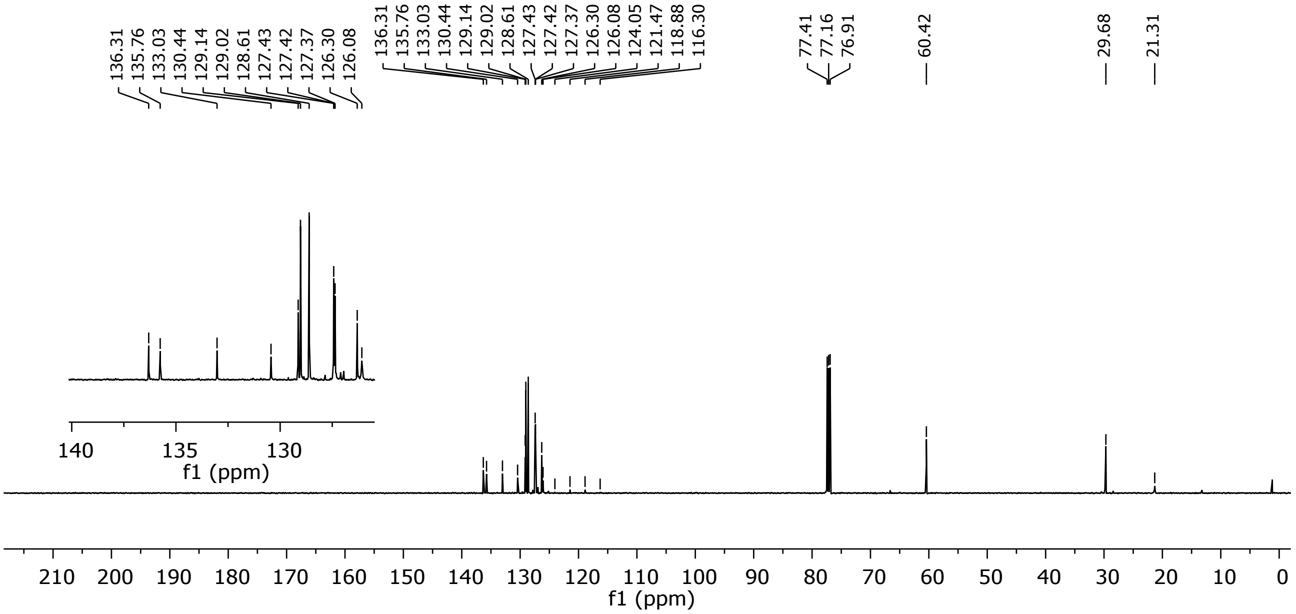

^{1}H NMR (500 MHz, $CDCl_3$)

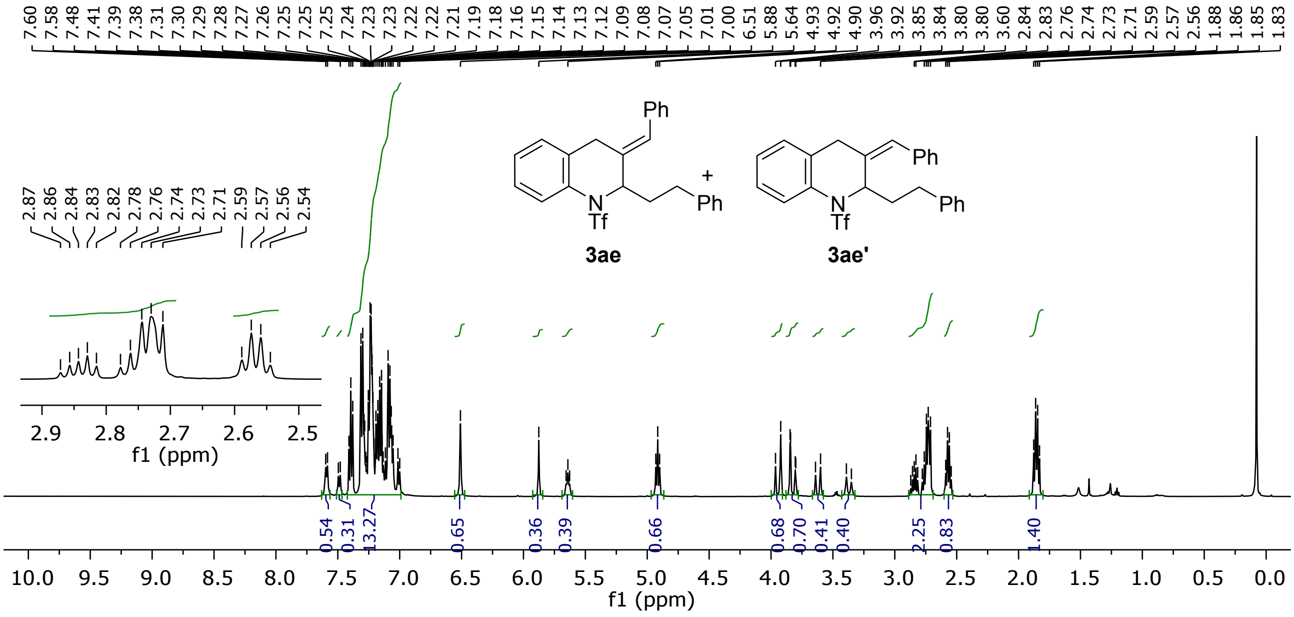

DEPT-135

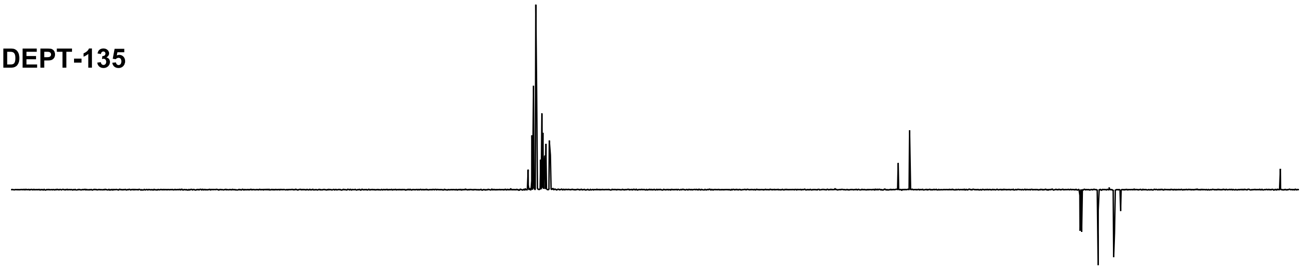

^{13}C NMR (126 MHz, $CDCl_3$)

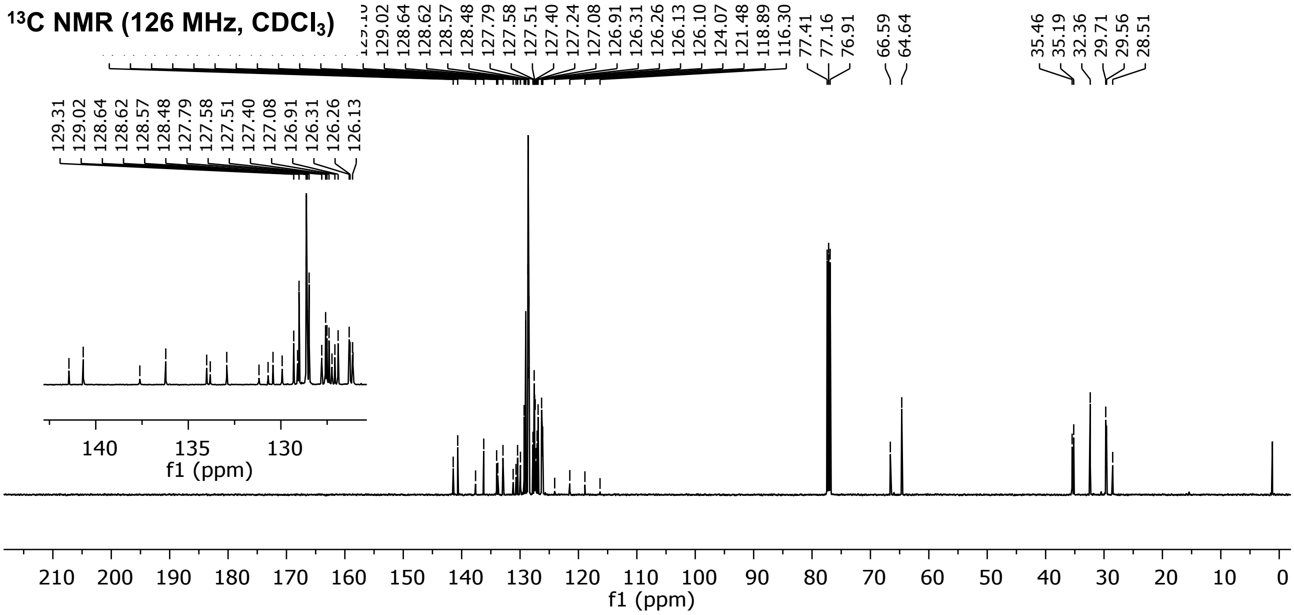

^{1}H NMR (500 MHz, CDCl$_3$)

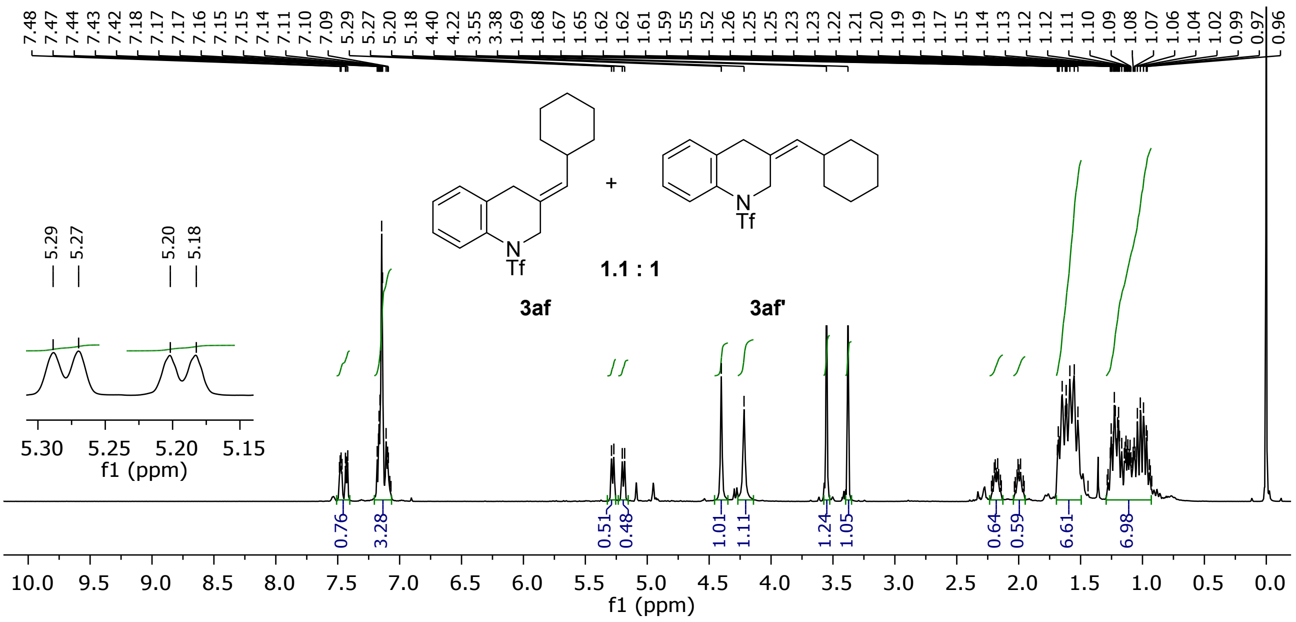

DEPT-135

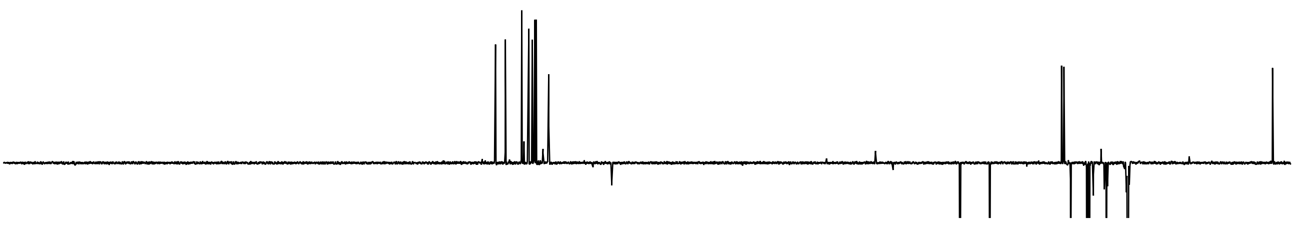

^{13}C NMR (126 MHz, CDCl$_3$)

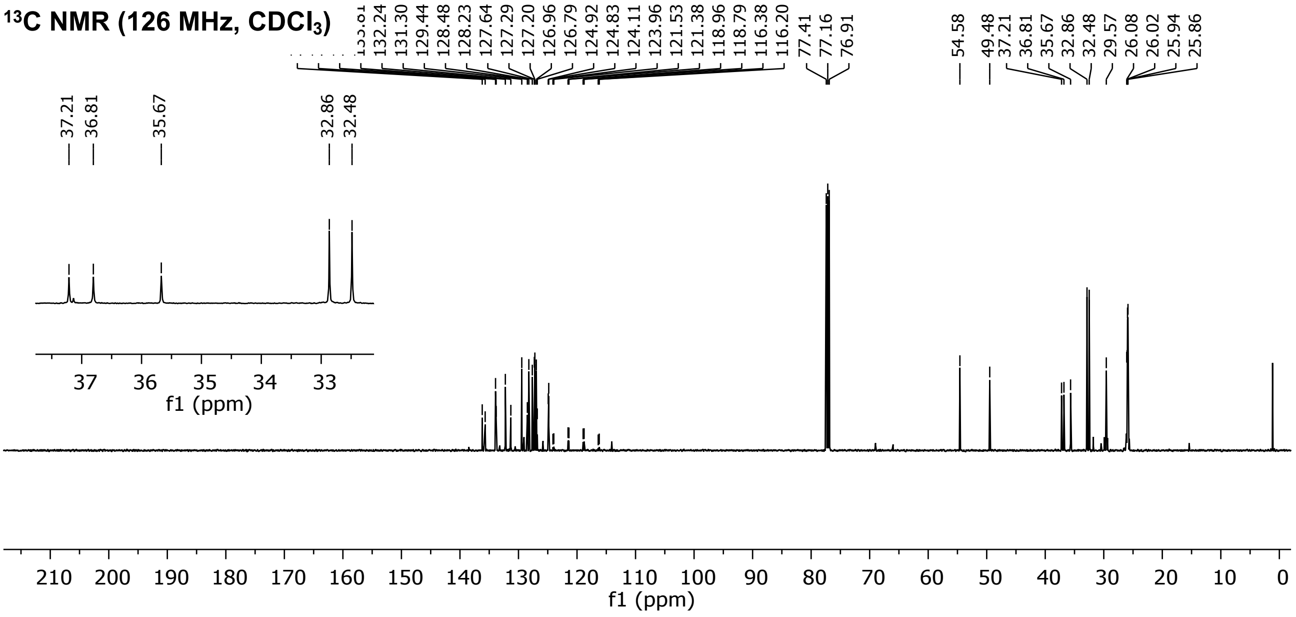

^{1}H NMR (500 MHz, CDCl$_3$)

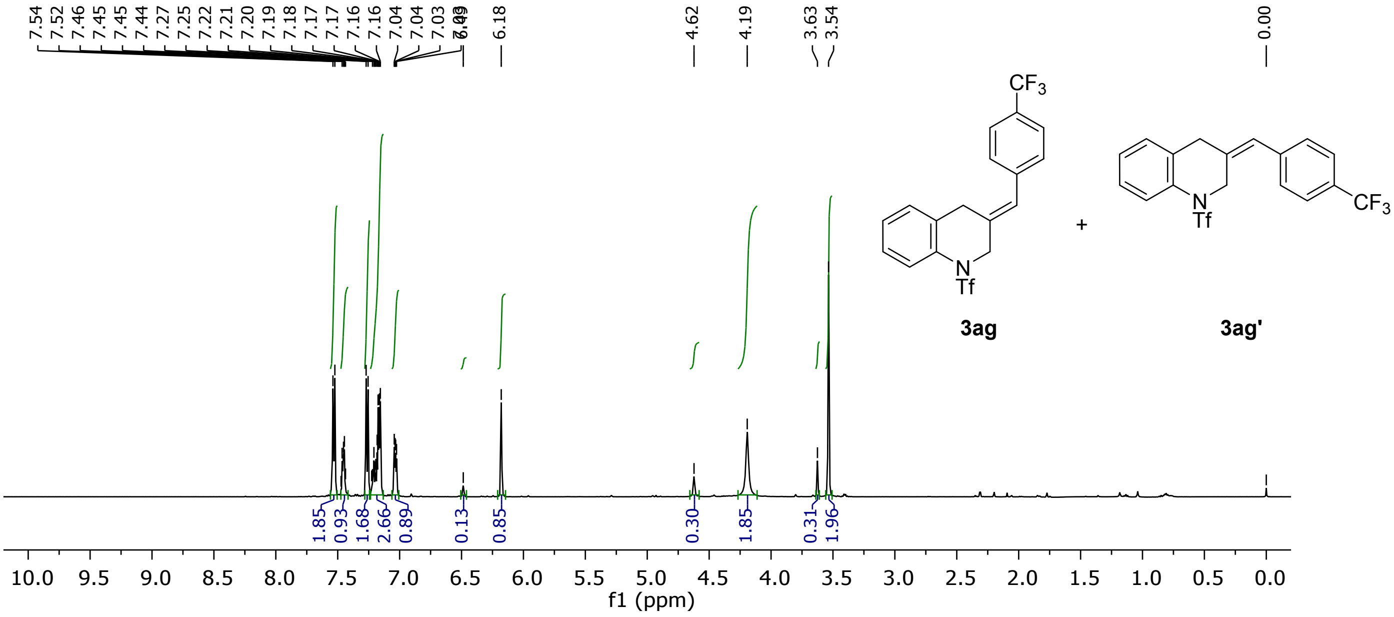

DEPT-135

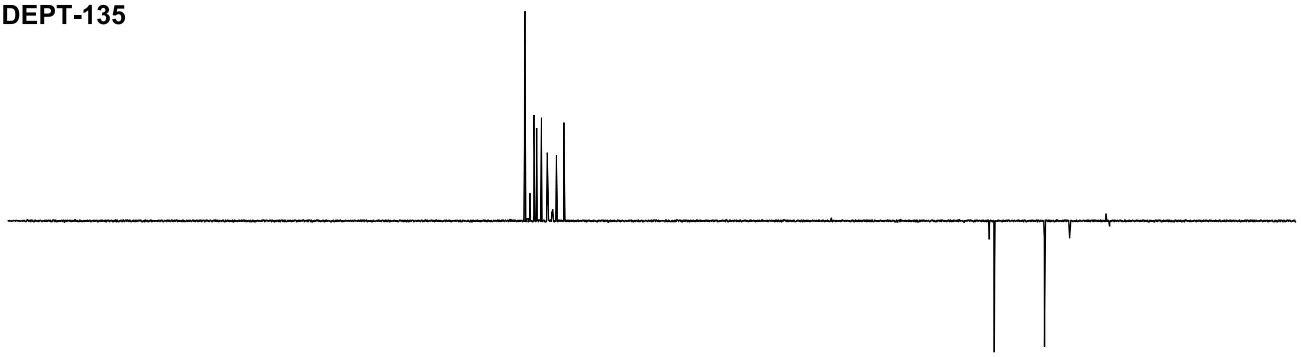

^{13}C NMR (126 MHz, CDCl$_3$)

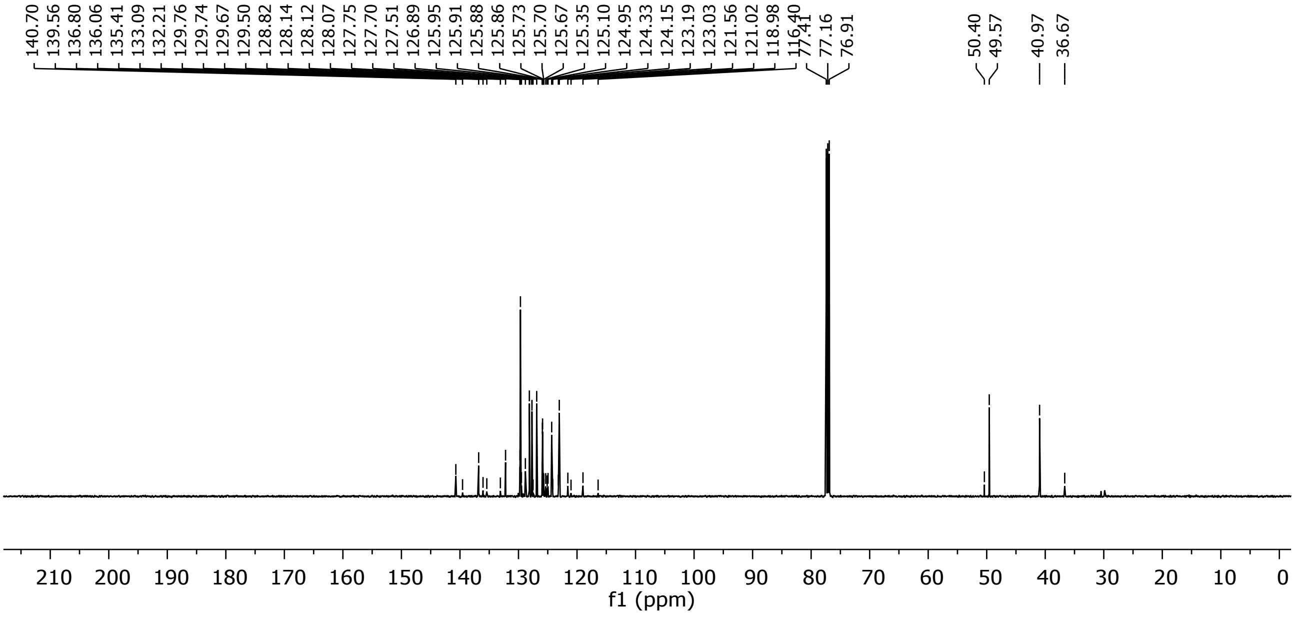

^{1}H NMR (300 MHz, CDCl$_3$)

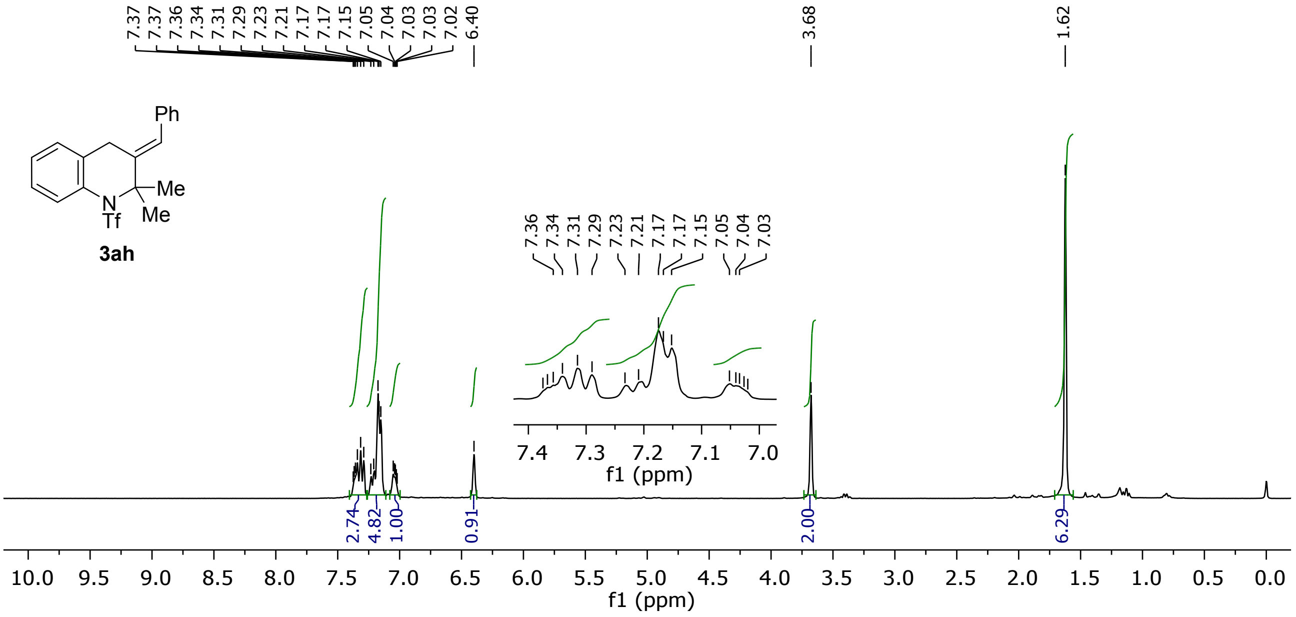

DEPT-135

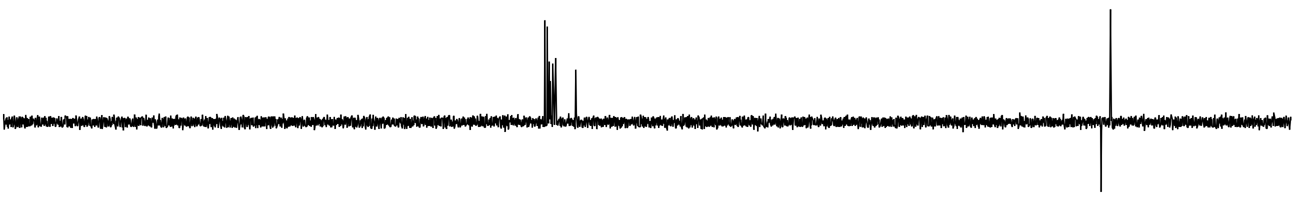

^{13}C NMR (75 MHz, CDCl$_3$)

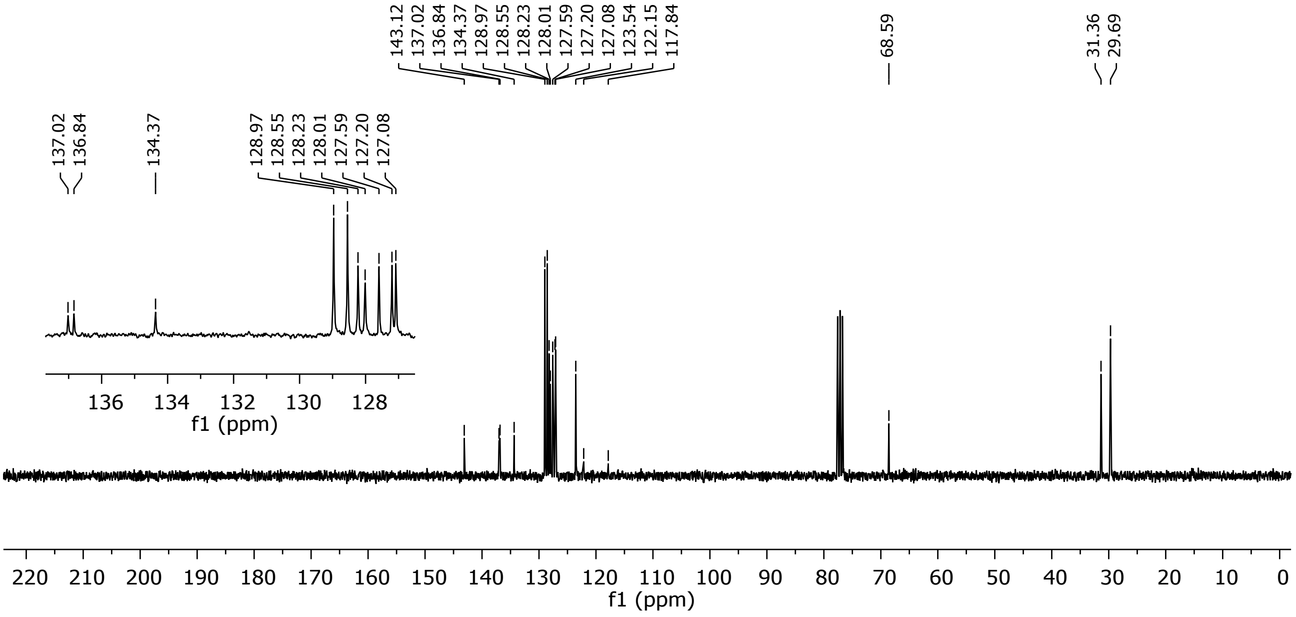

^{1}H NMR (500 MHz, CDCl$_3$)

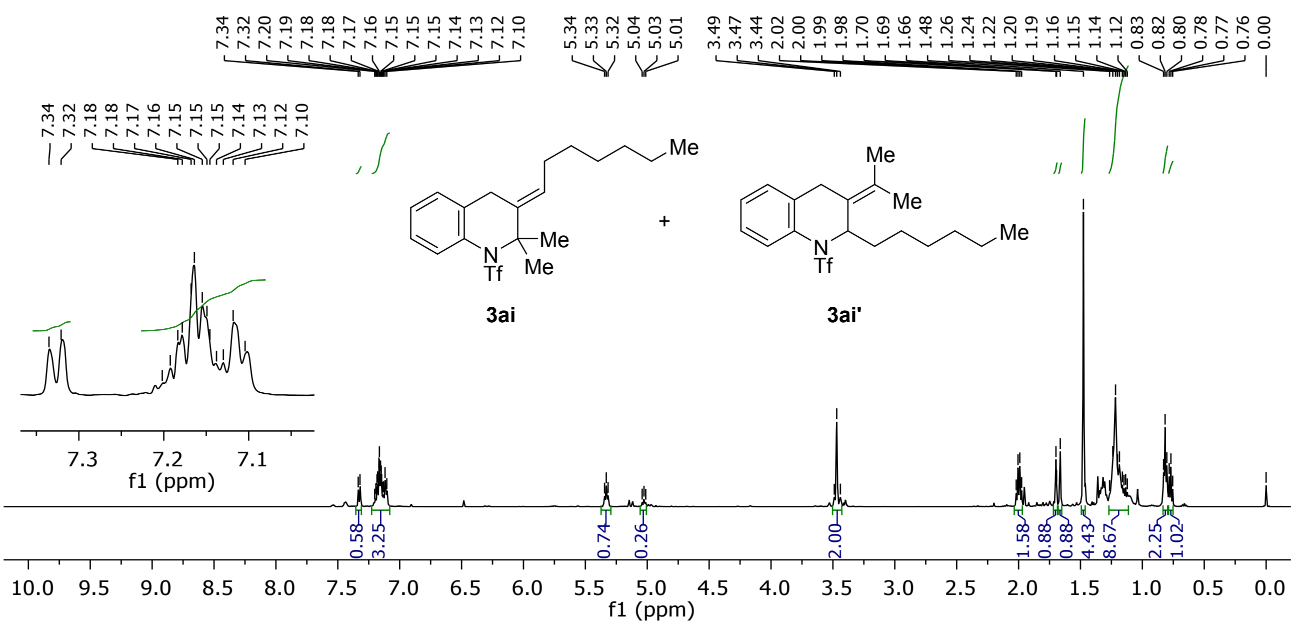

DEPT-135

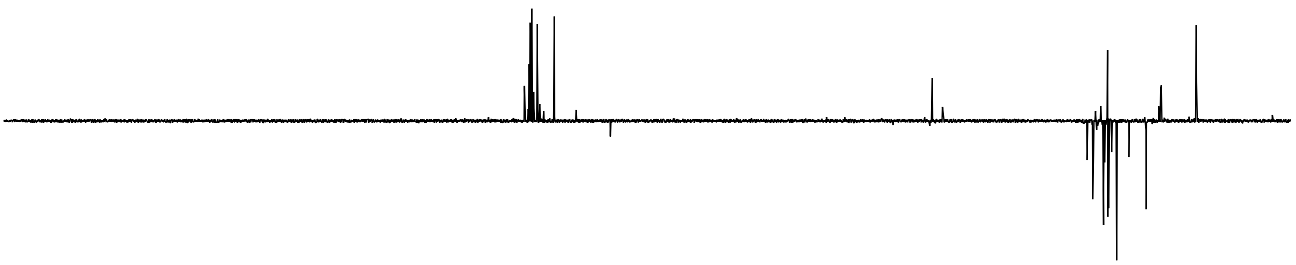

^{13}C NMR (126 MHz, CDCl$_3$)

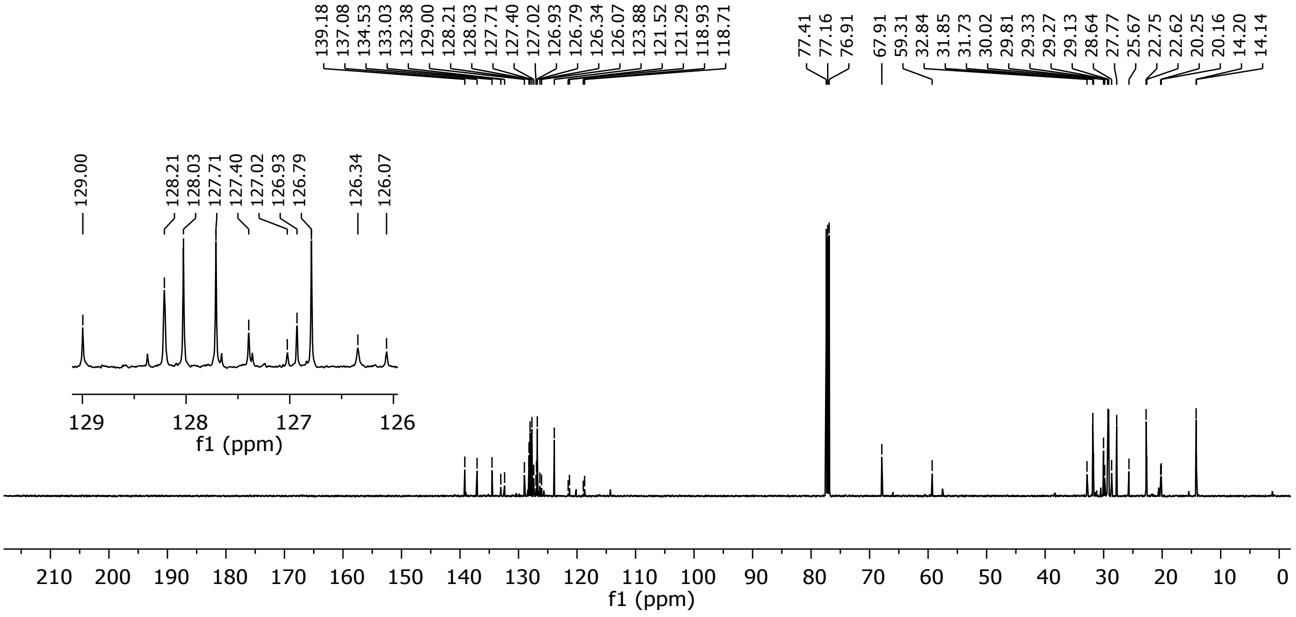

HSQC spectrum (CDCl$_3$)

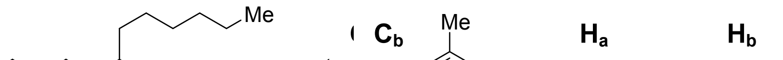

^{1}H NMR (300 MHz, $CDCl_3$)

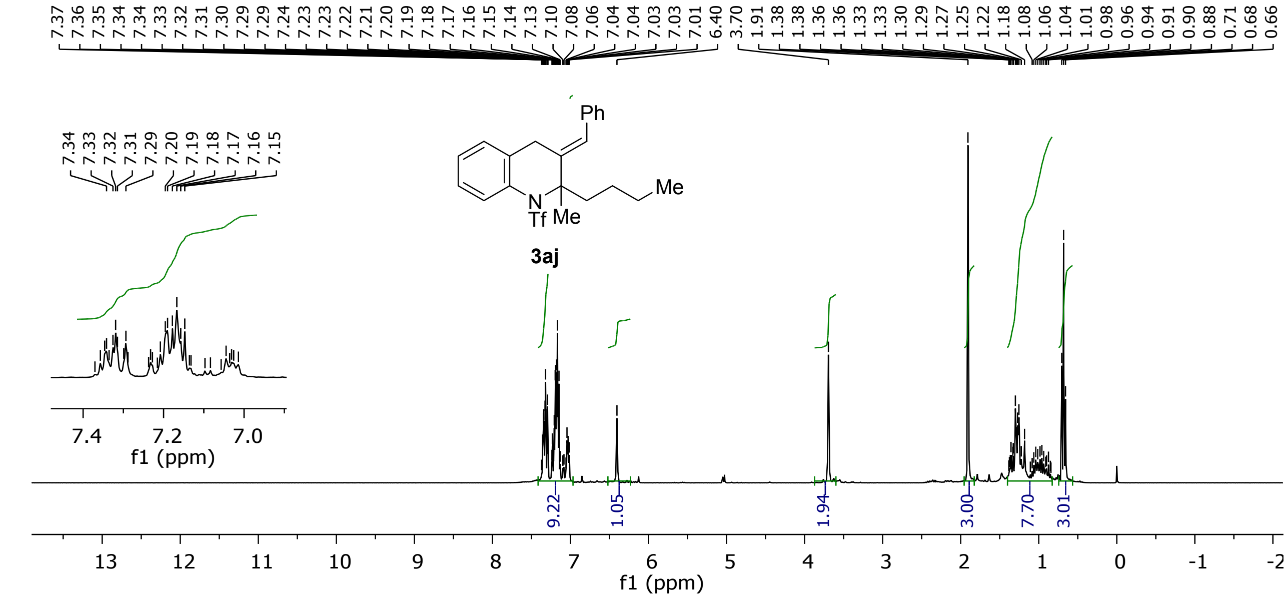

DEPT-135

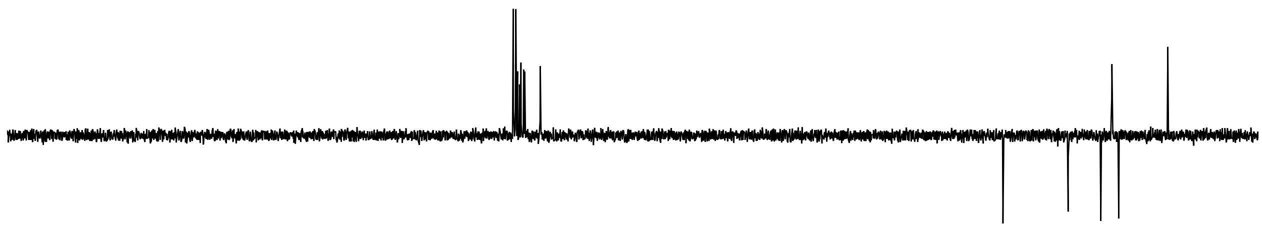

^{13}C NMR (75 MHz, $CDCl_3$)

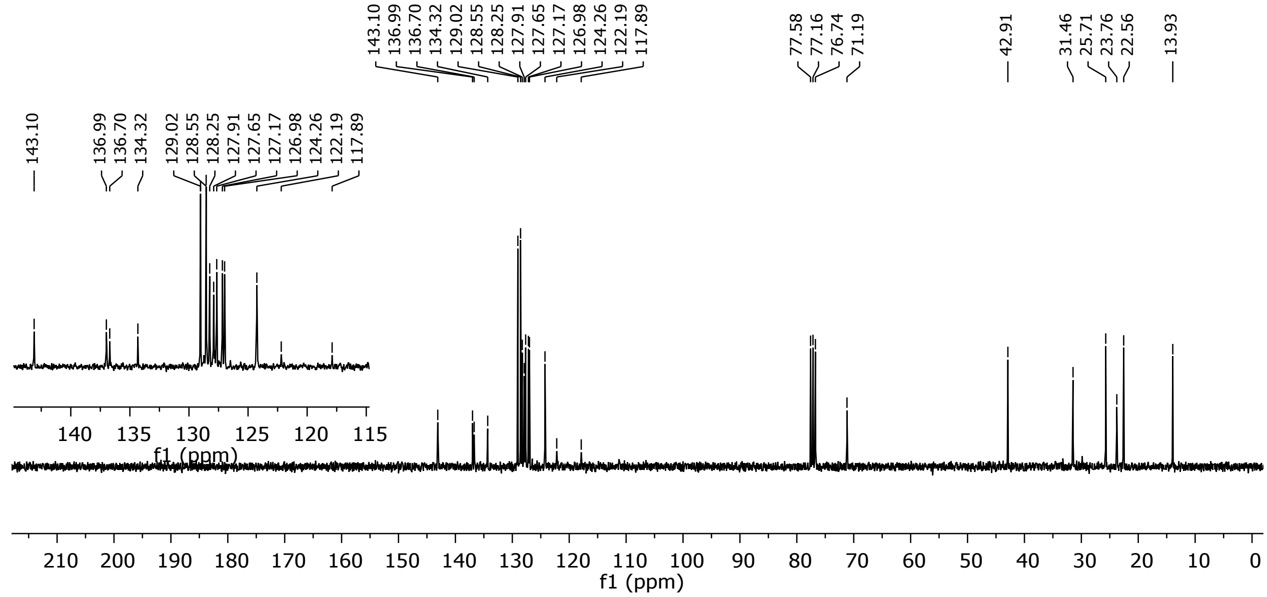

^{1}H NMR (300 MHz, CDCl$_3$)

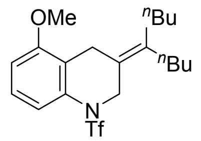

3ba

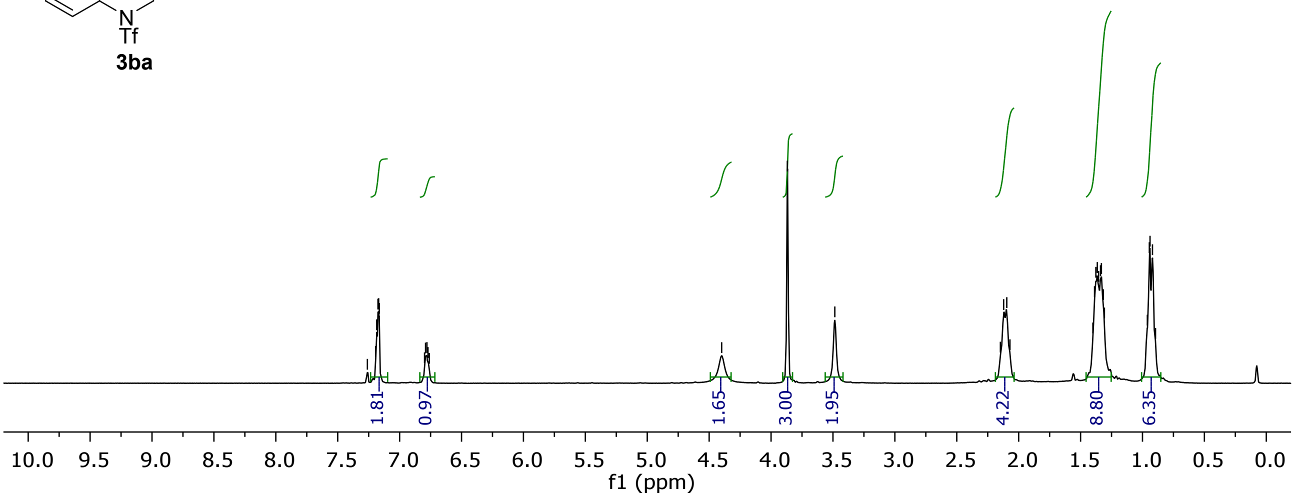

DEPT-135

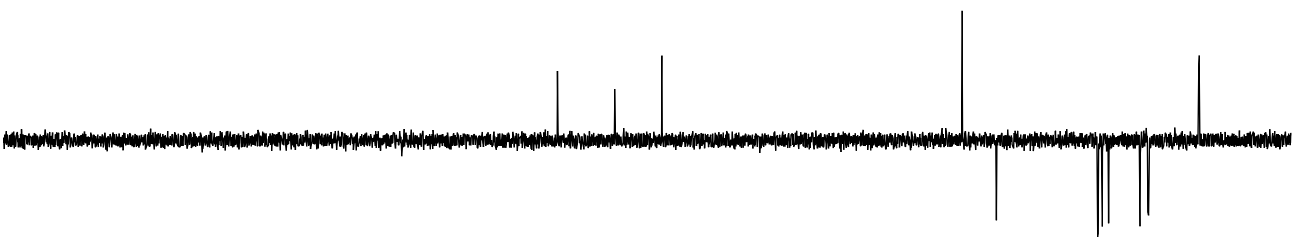

^{13}C NMR (75 MHz, CDCl$_3$)

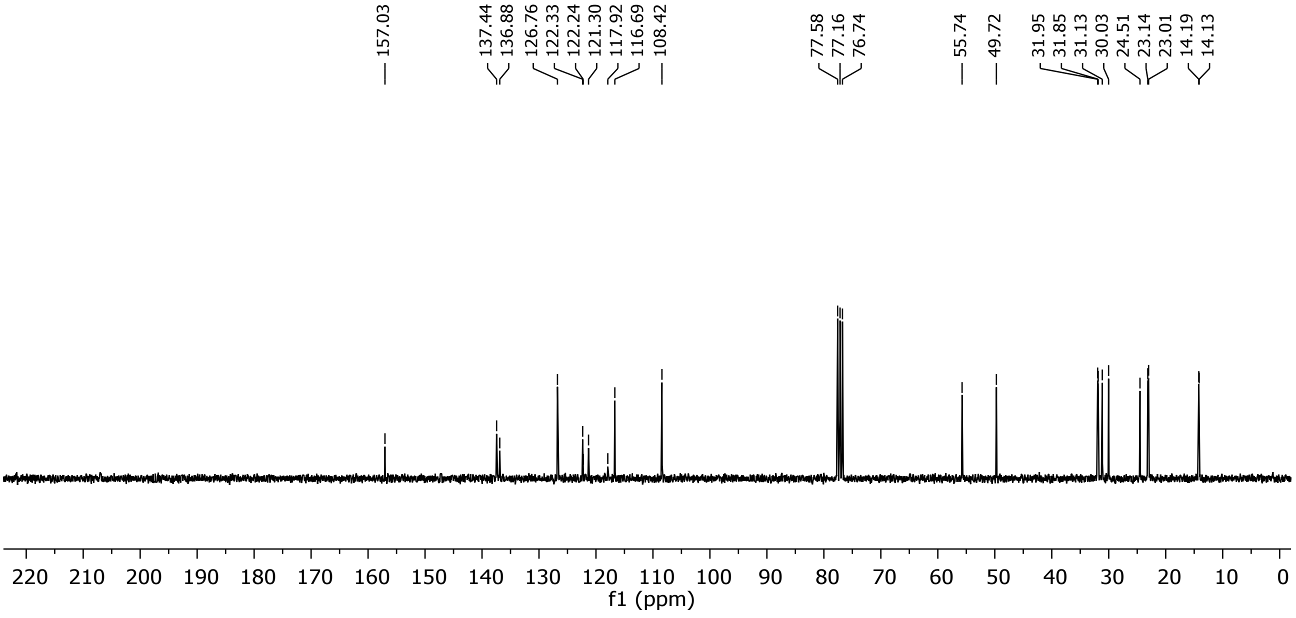

^{1}H NMR (500 MHz, CDCl$_3$)

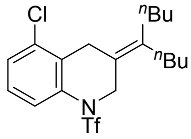

3ca

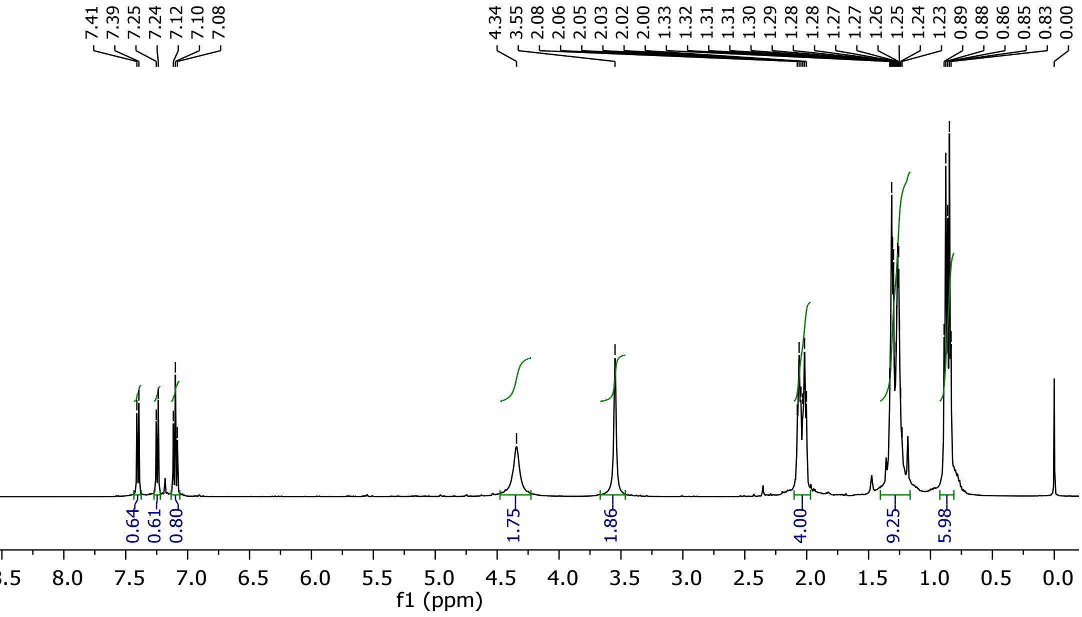

DEPT-135

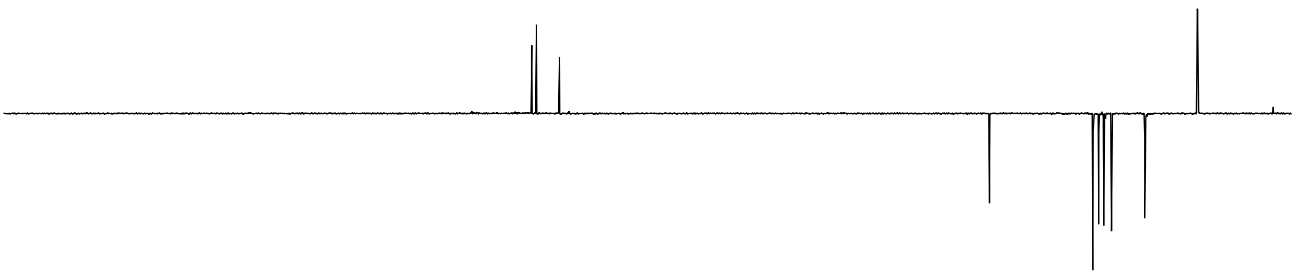

^{13}C NMR (126 MHz, CDCl$_3$)

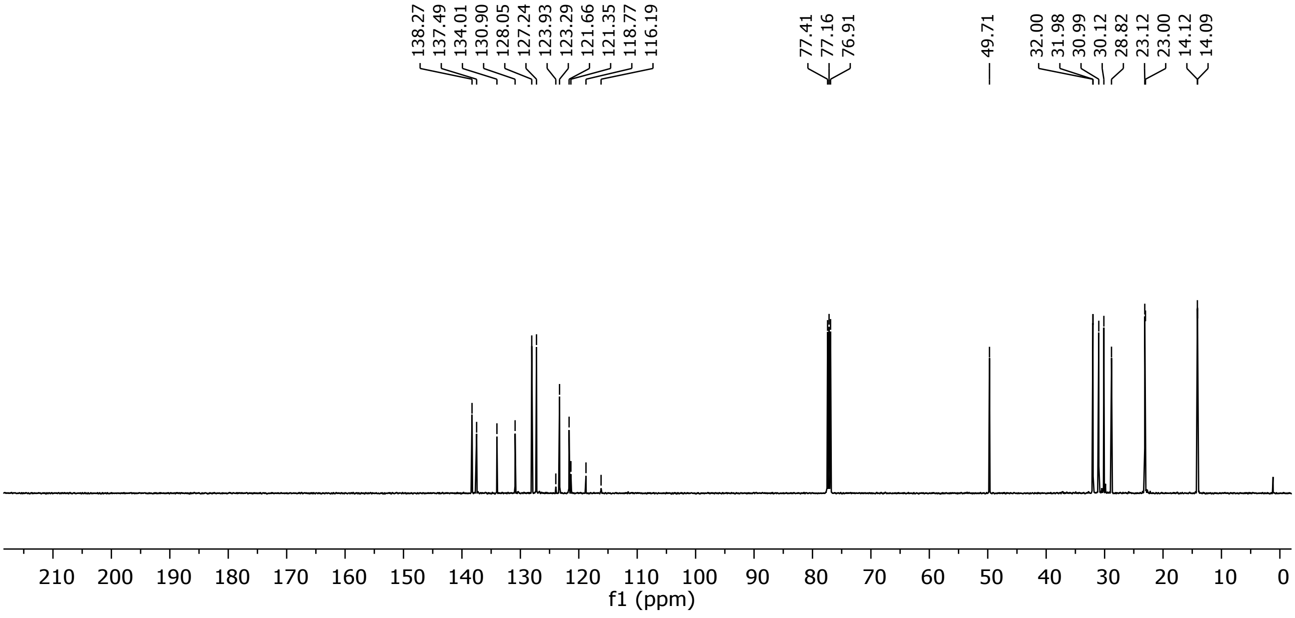

^{1}H NMR (300 MHz, $CDCl_3$)

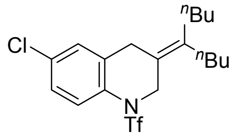

3da

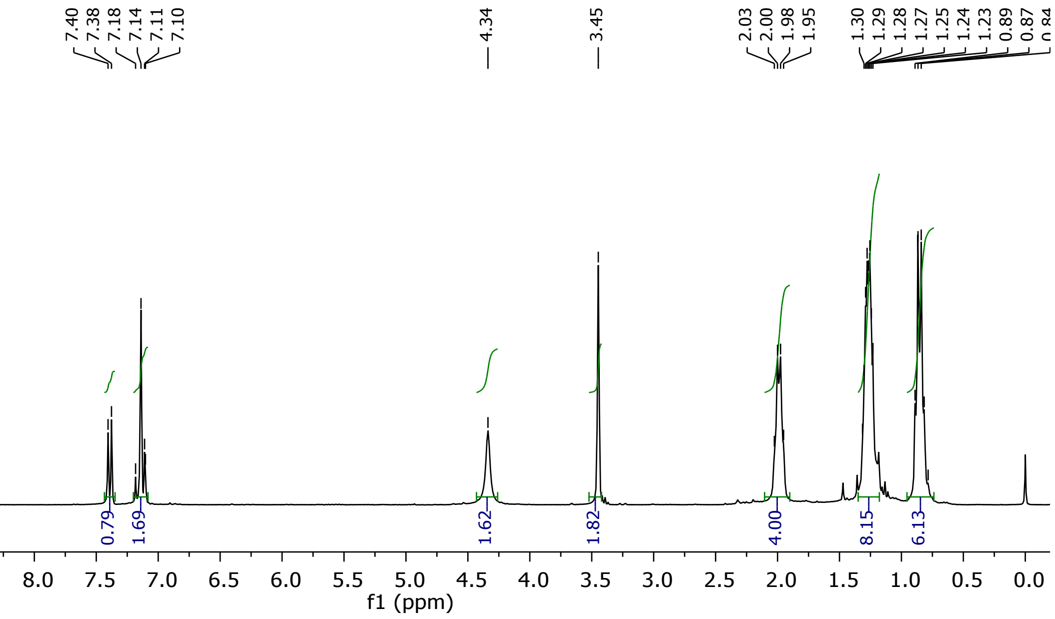

DEPT-135

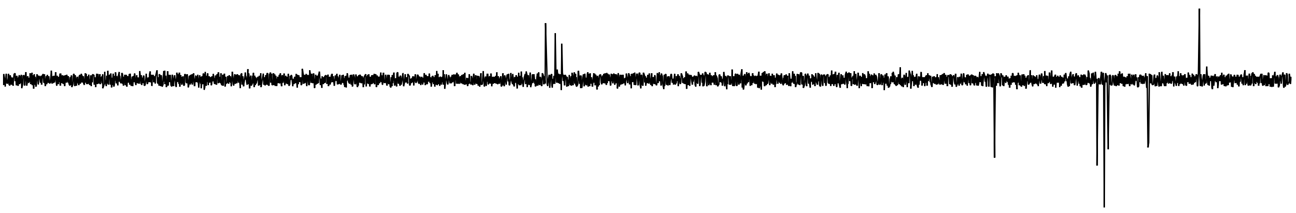

^{13}C NMR (75 MHz, $CDCl_3$)

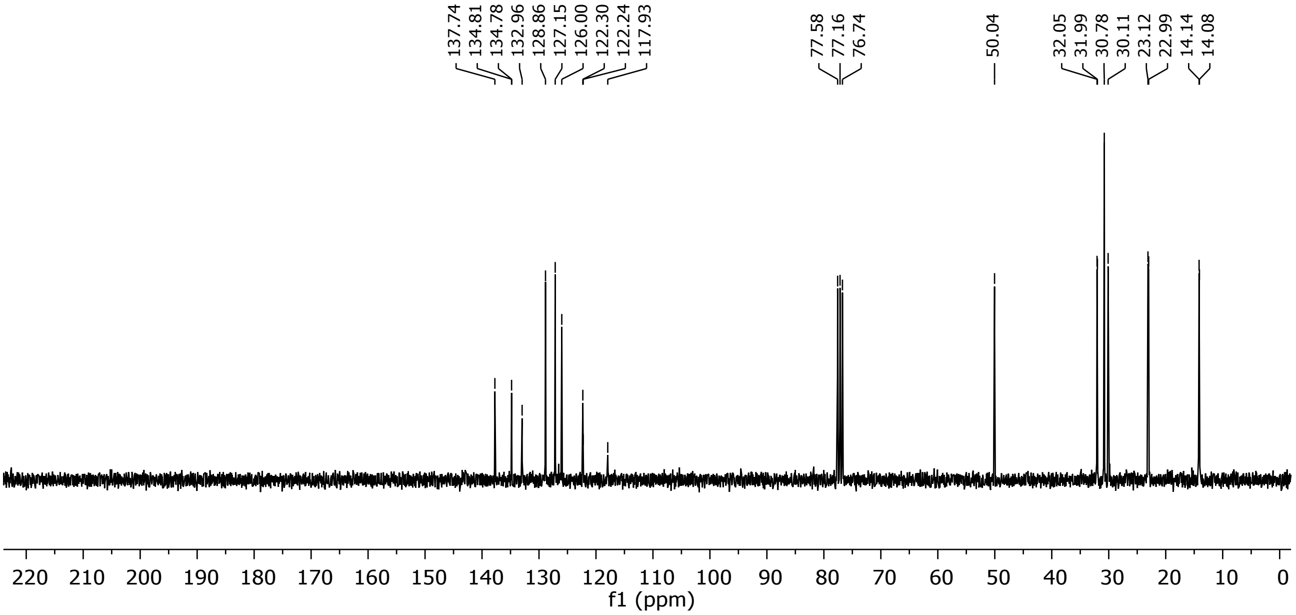

^{1}H NMR (300 MHz, $CDCl_3$)

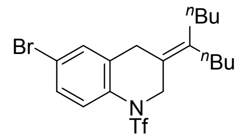

3ea

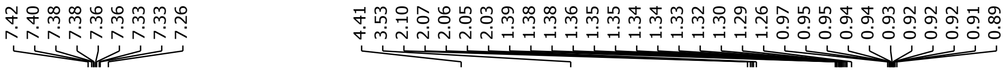

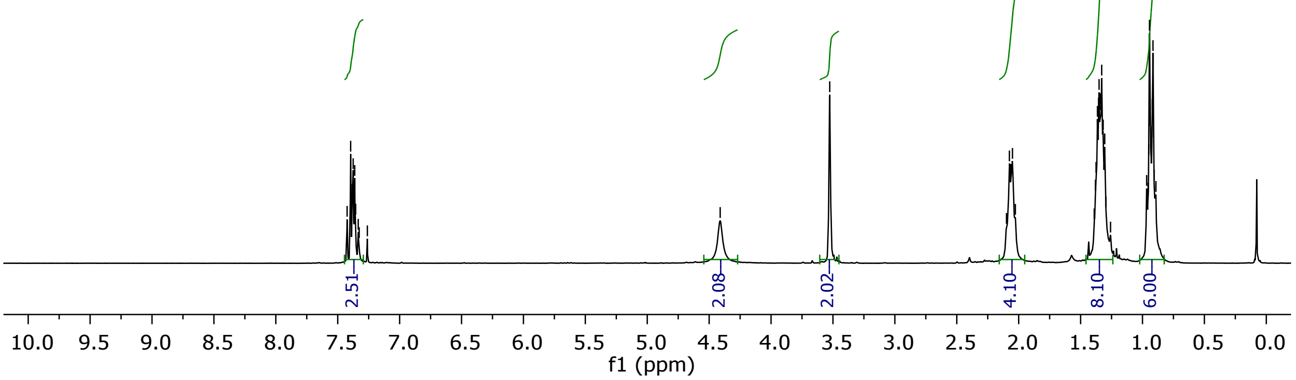

DEPT-135

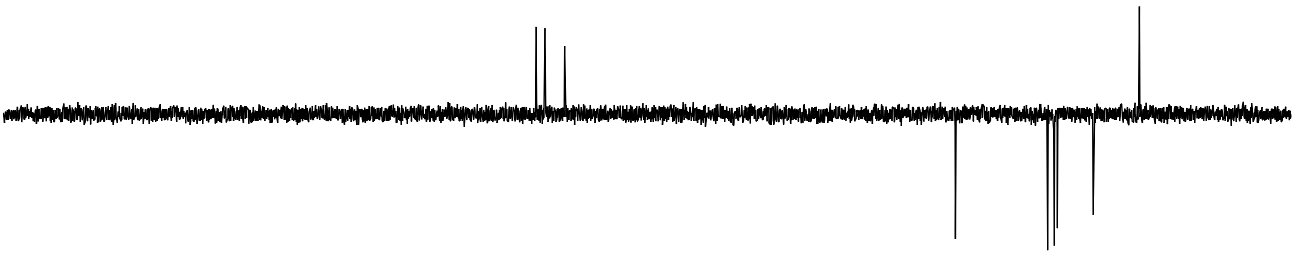

^{13}C NMR (75 MHz, $CDCl_3$)

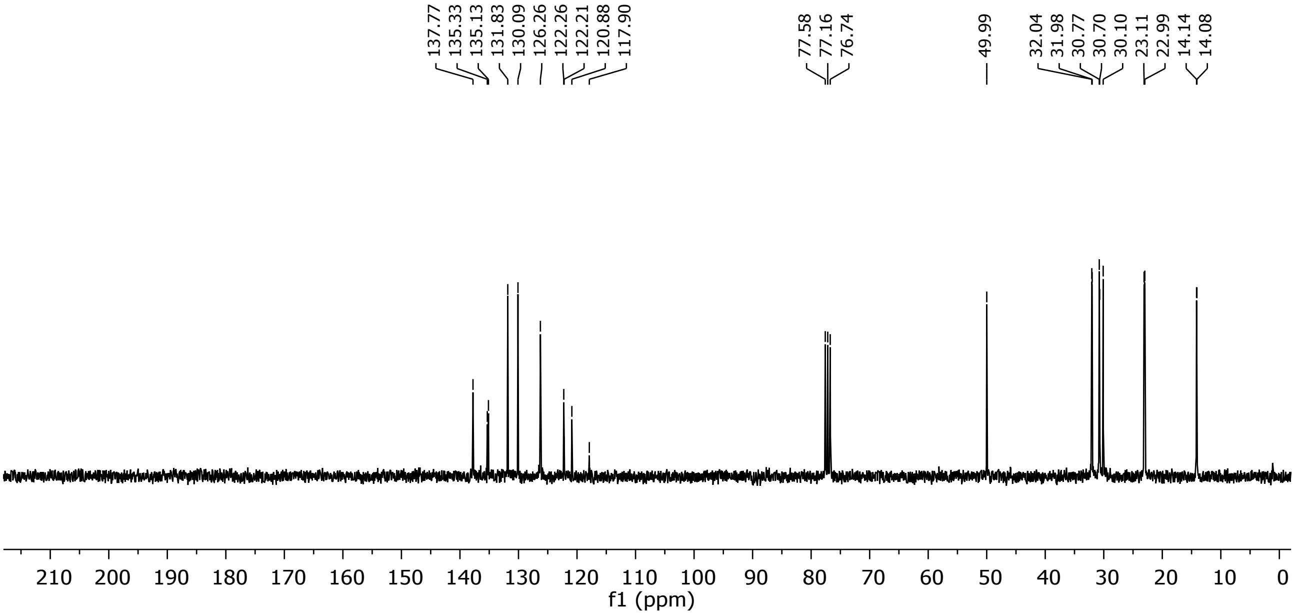

^{1}H NMR (300 MHz, $CDCl_3$)

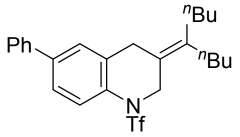

3fa

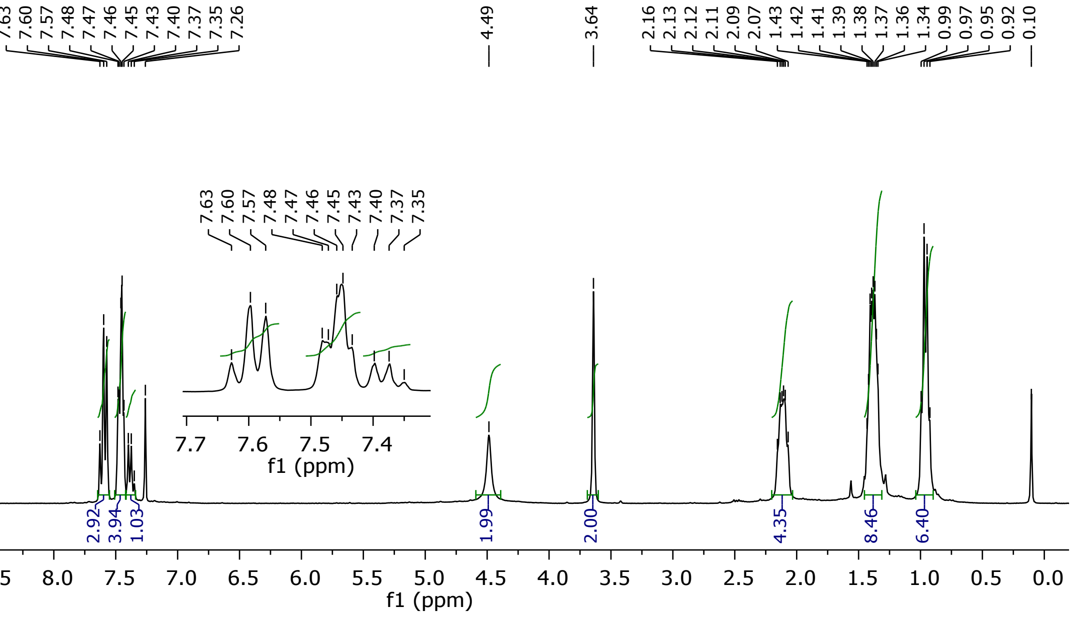

DEPT-135

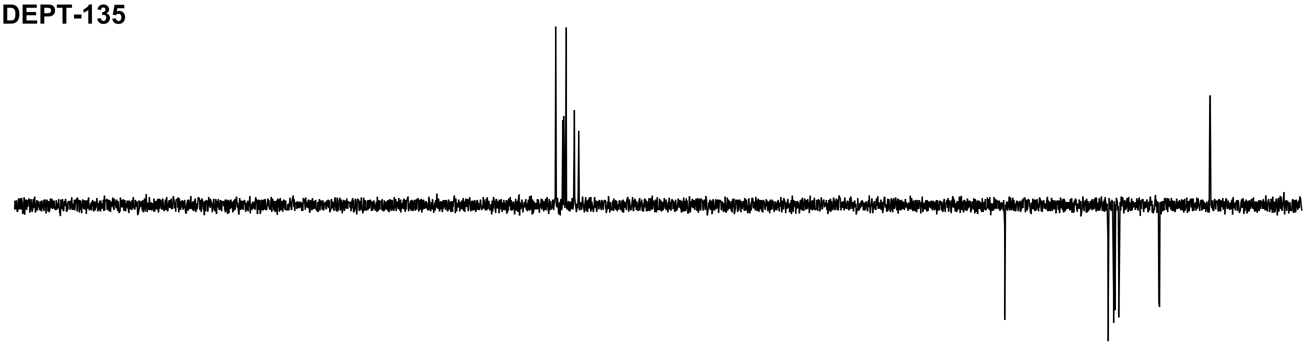

^{13}C NMR (75 MHz, $CDCl_3$)

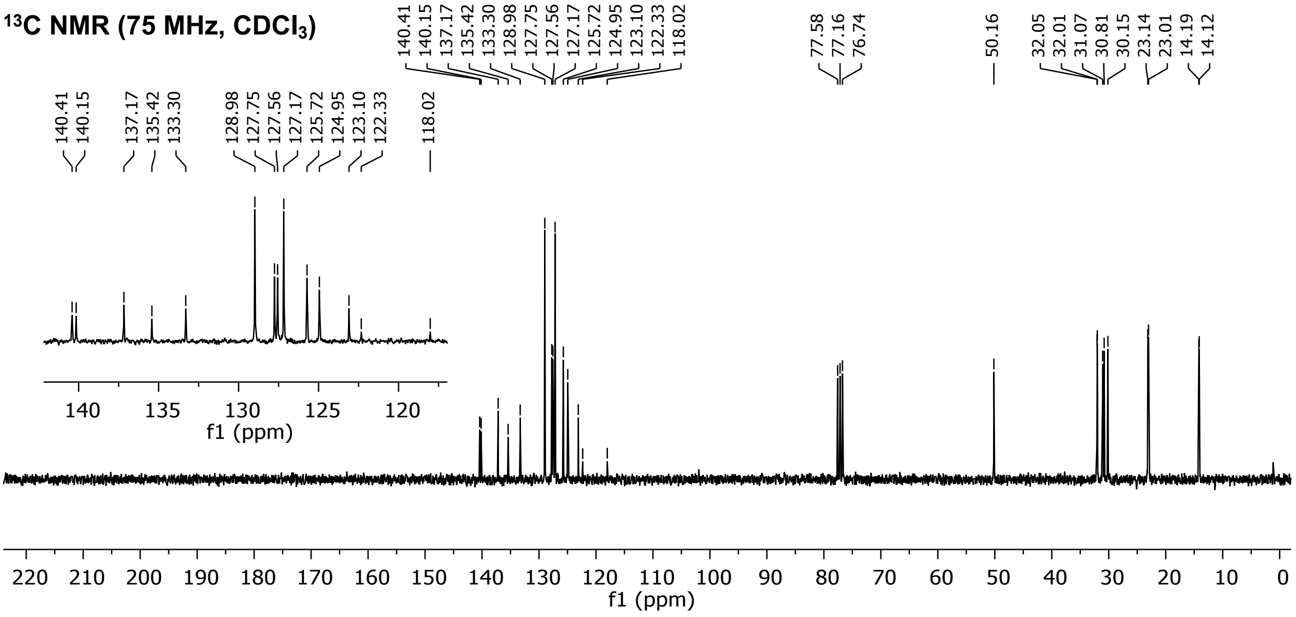

^{1}H NMR (300 MHz, $CDCl_3$)

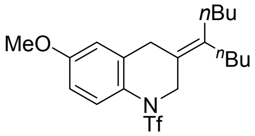

3ga

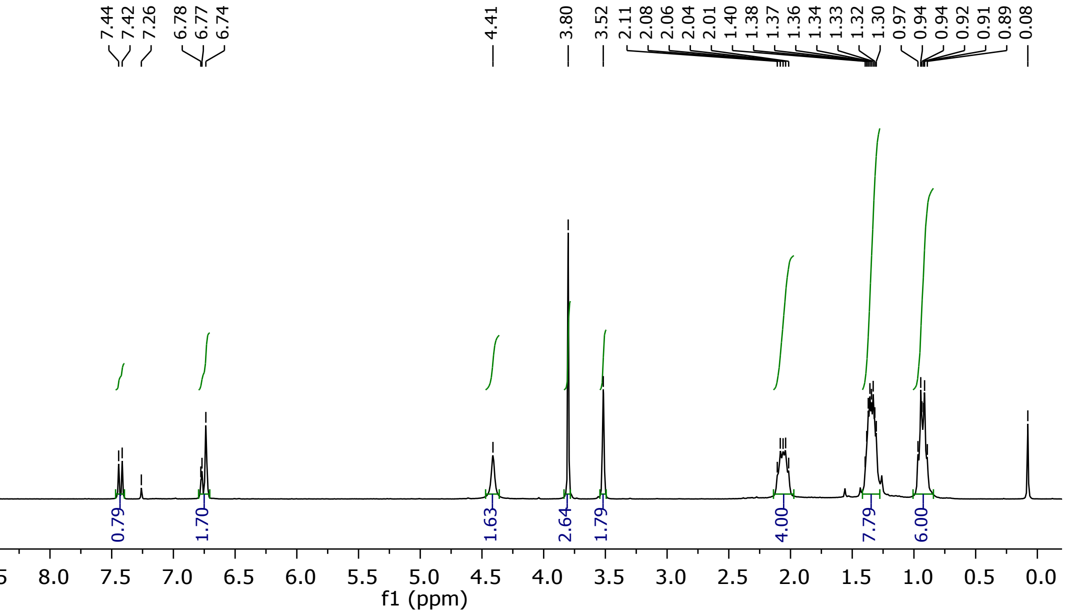

DEPT-135

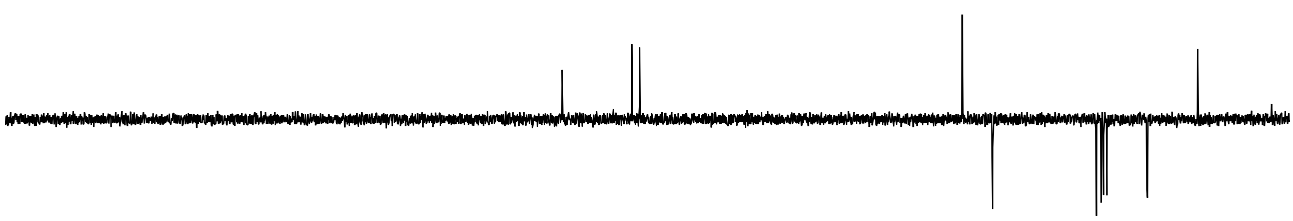

^{13}C NMR (75 MHz, $CDCl_3$)

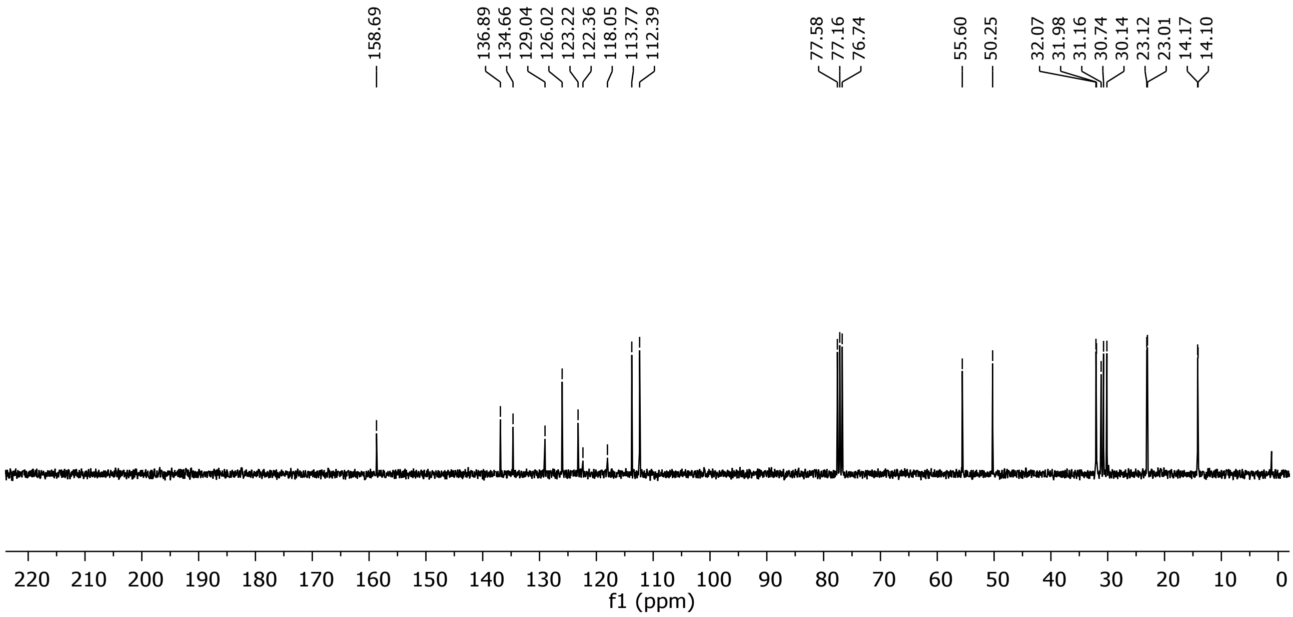

^{1}H NMR (300 MHz, CDCl$_3$)

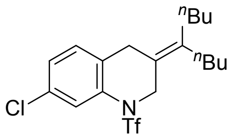

3ha

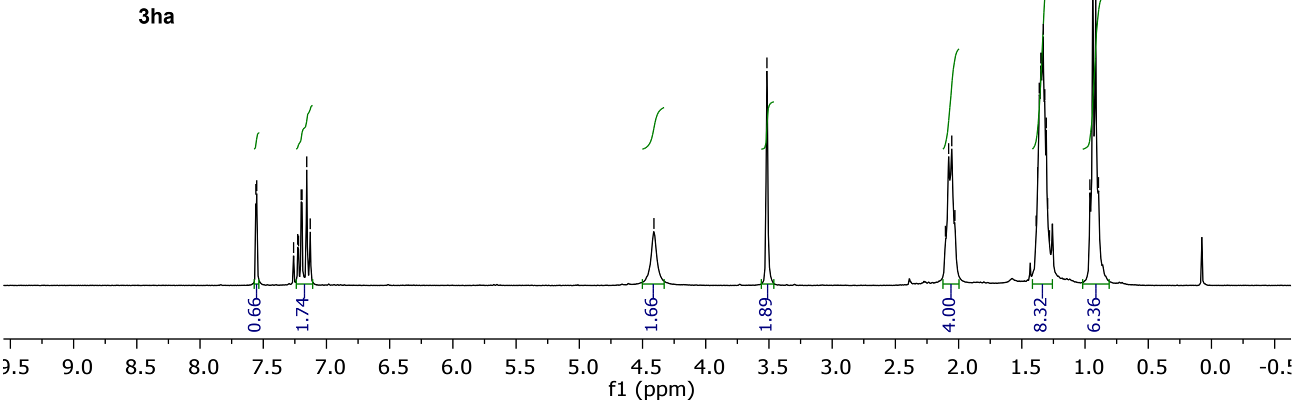

DEPT-135

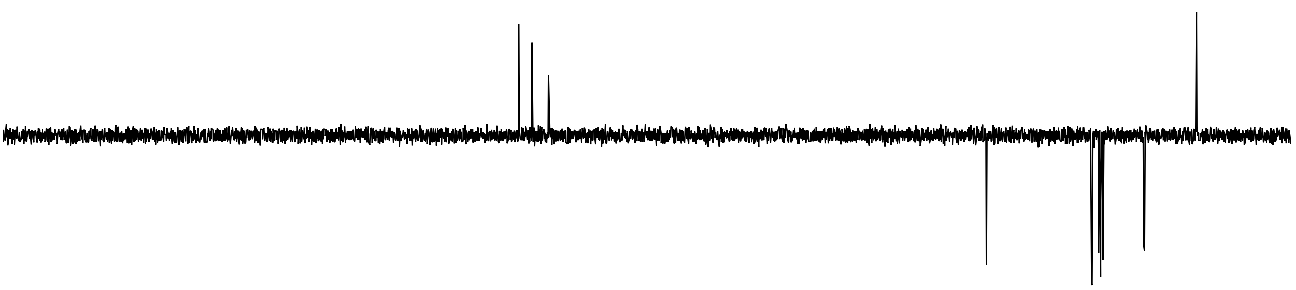

^{13}C NMR (75 MHz, CDCl$_3$)

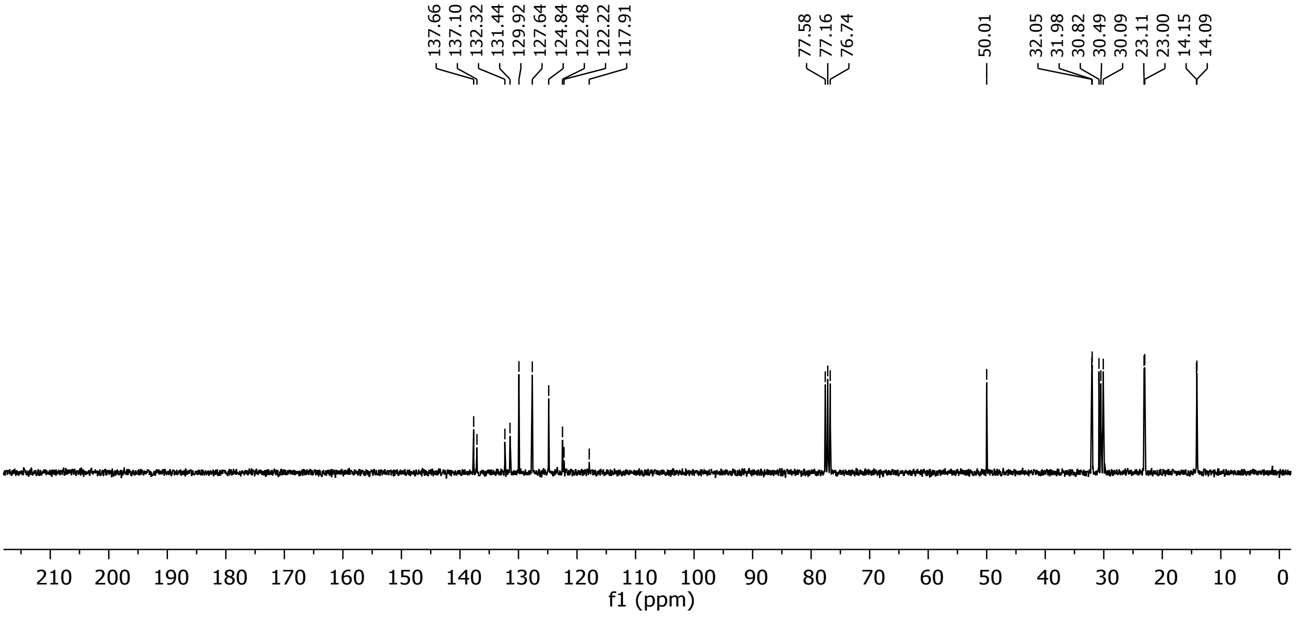

^{1}H NMR (500 MHz, $CDCl_3$)

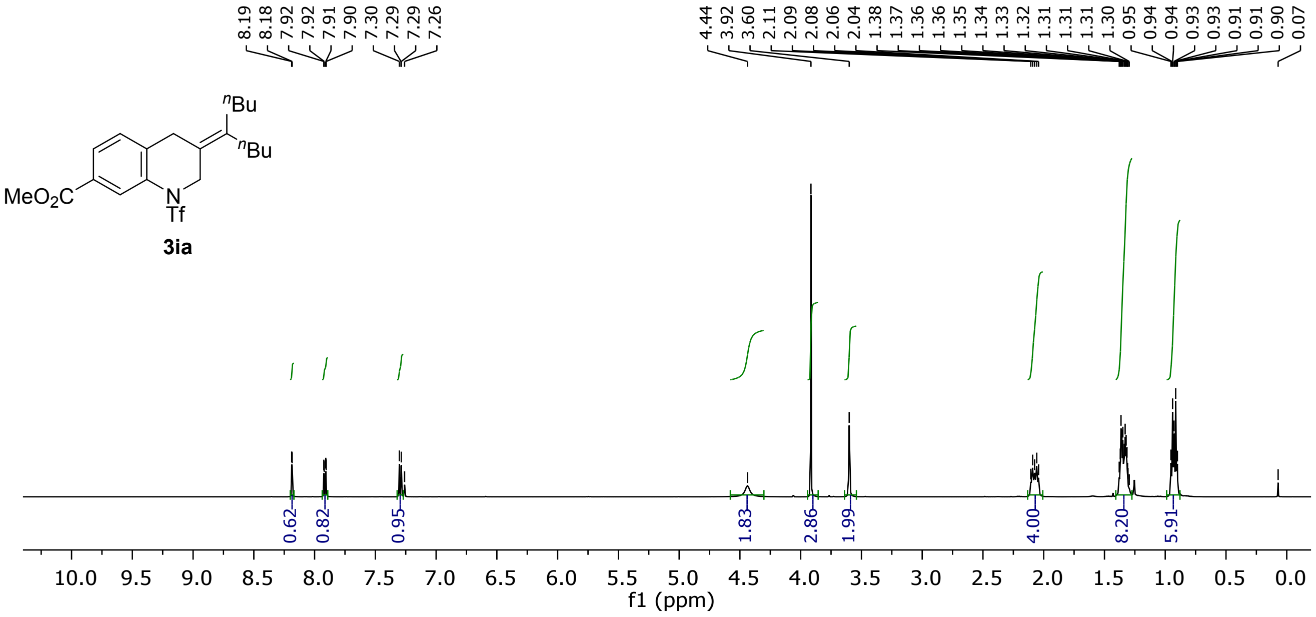

DEPT-135

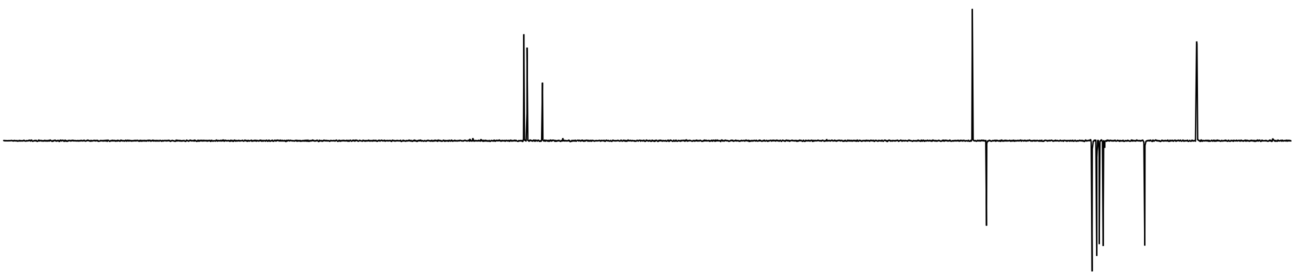

^{13}C NMR (126 MHz, $CDCl_3$)

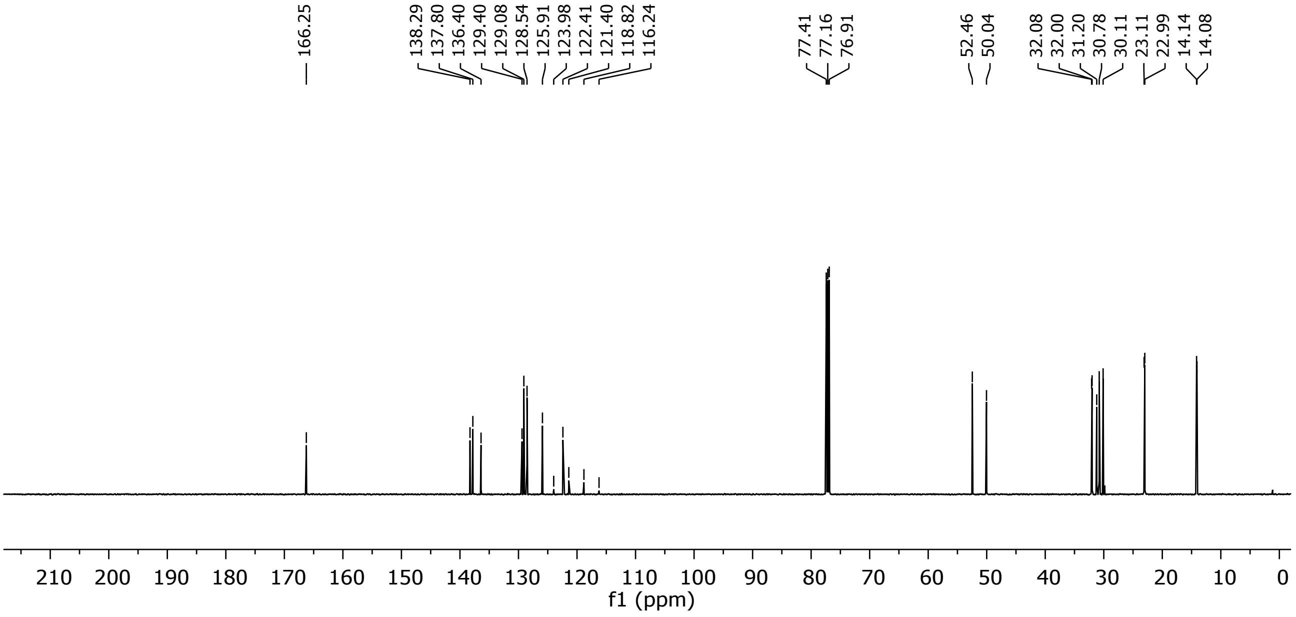

¹H NMR (500 MHz, CDCl$_3$)

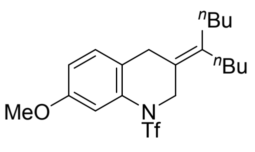

3ja

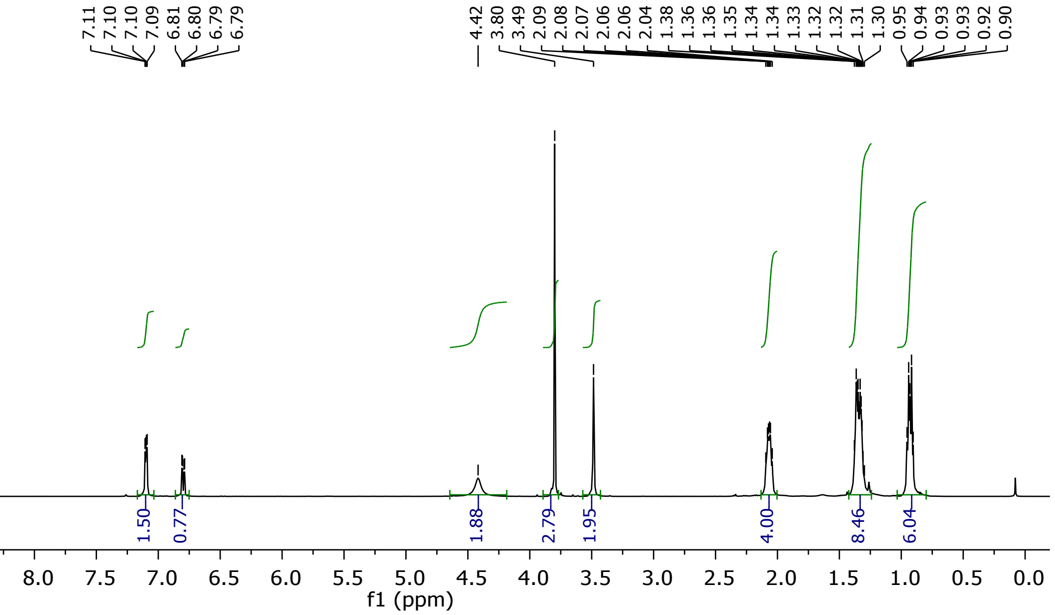

DEPT-135

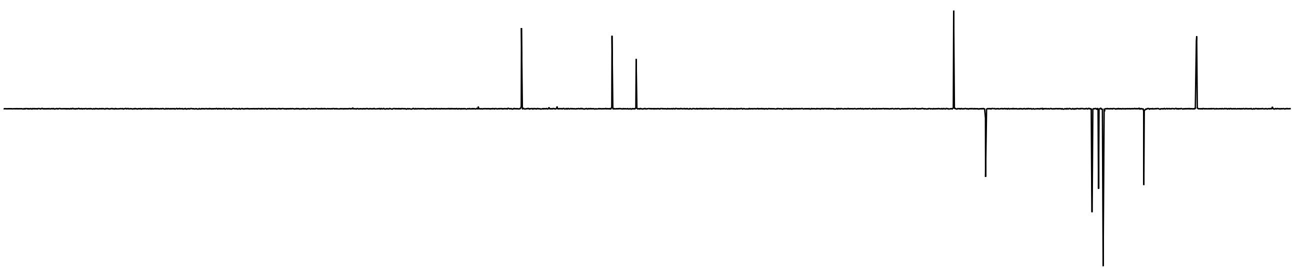

¹³C NMR (126 MHz, CDCl$_3$)

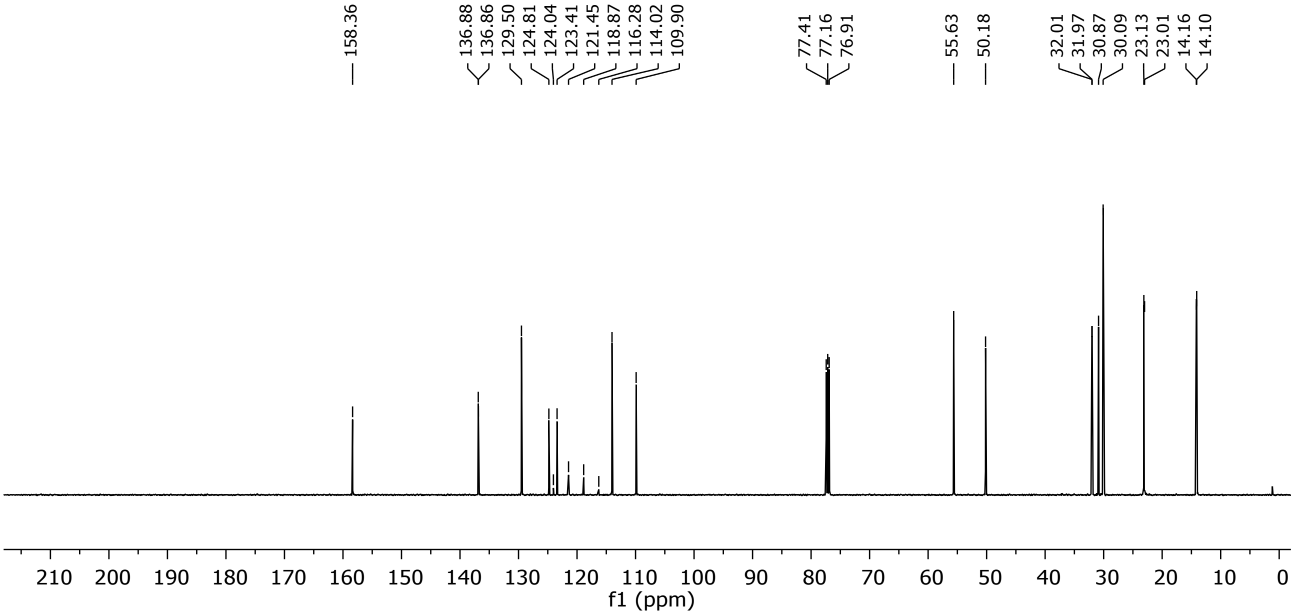

^{1}H NMR (300 MHz, CDCl$_3$)

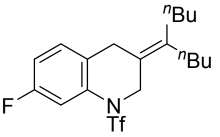

3ka

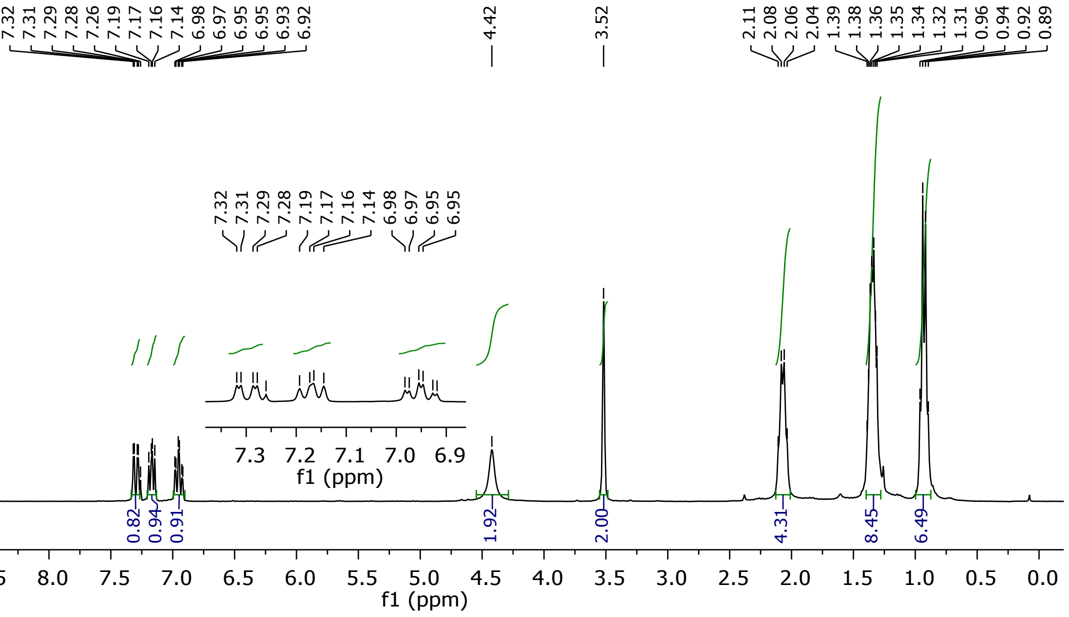

DEPT-135

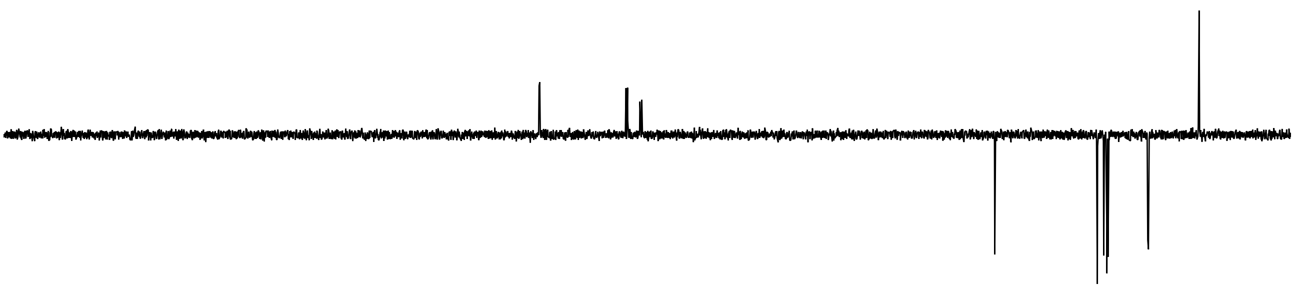

^{13}C NMR (75 MHz, CDCl$_3$)

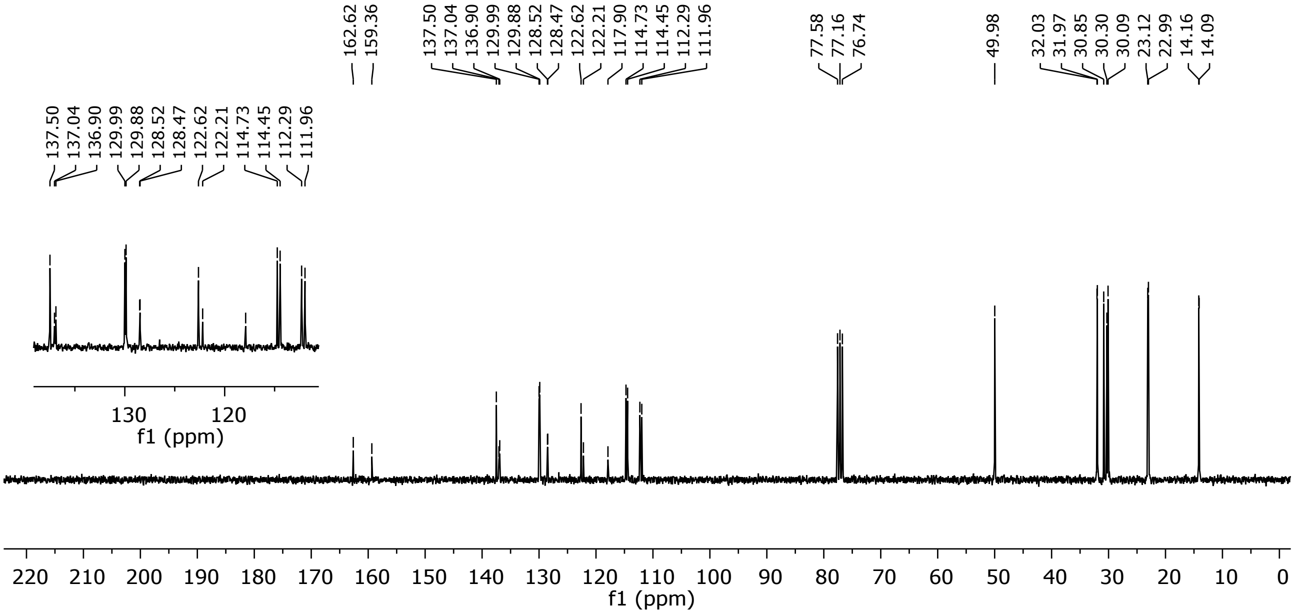

^{1}H NMR (500 MHz, CDCl$_3$)

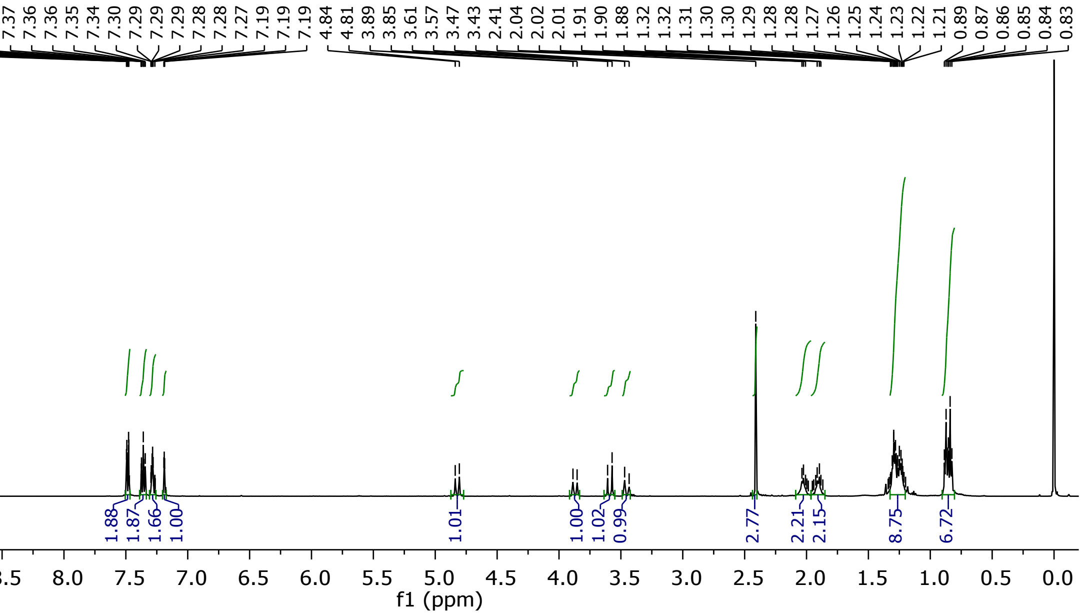

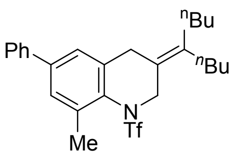

3la

DEPT-135

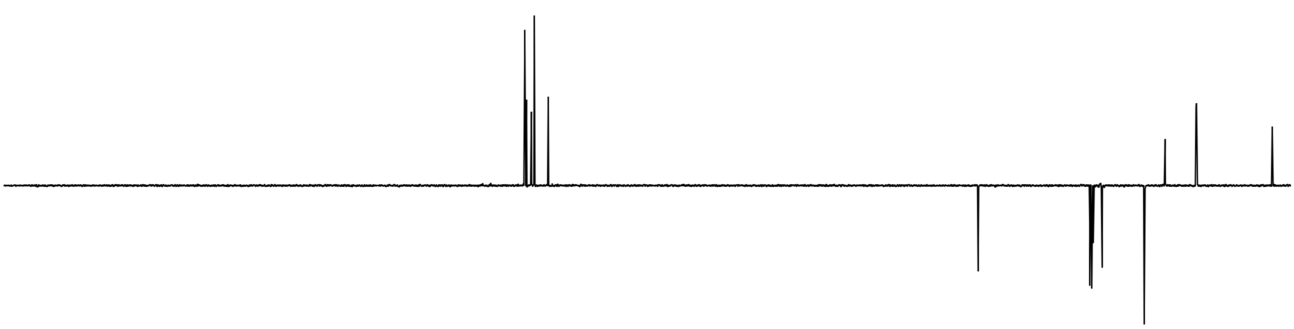

^{13}C NMR (126 MHz, CDCl$_3$)

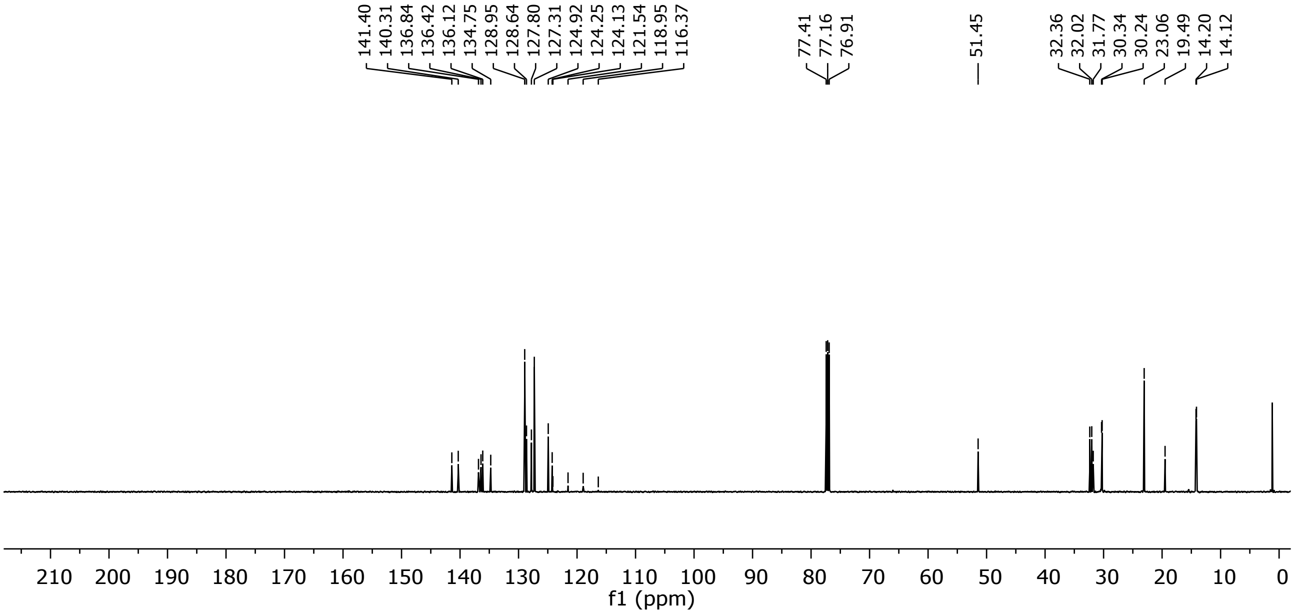

^{1}H NMR (500 MHz, CDCl$_3$)

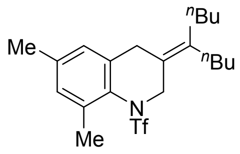

3ma

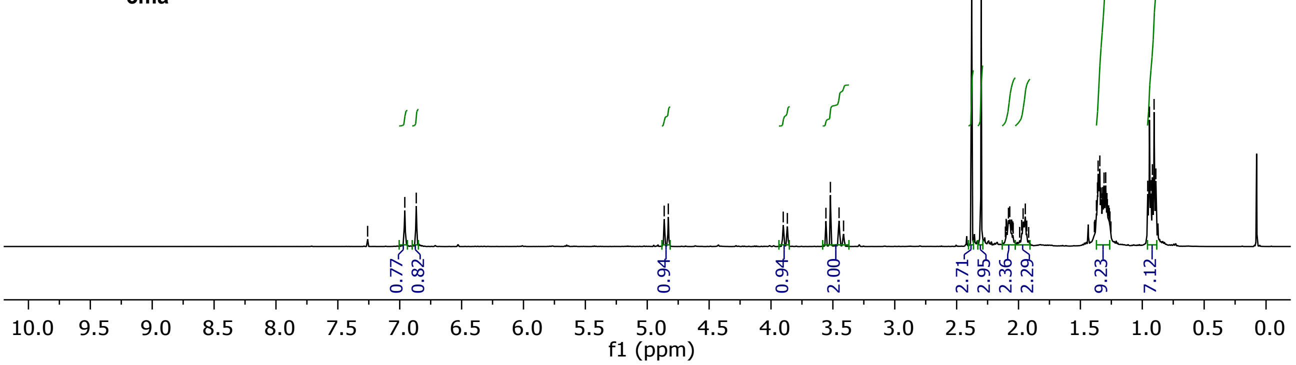

DEPT-135

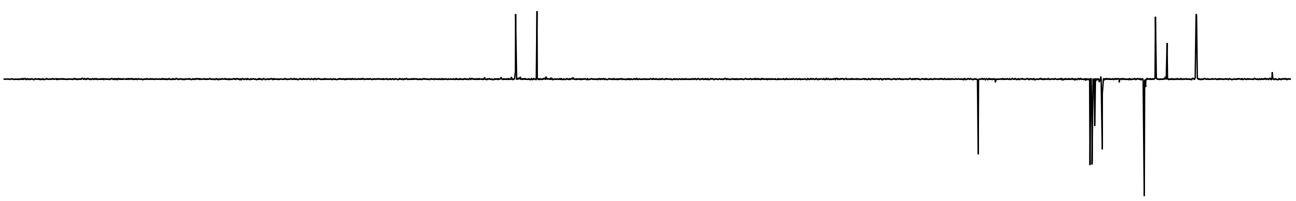

^{13}C NMR (126 MHz, CDCl$_3$)

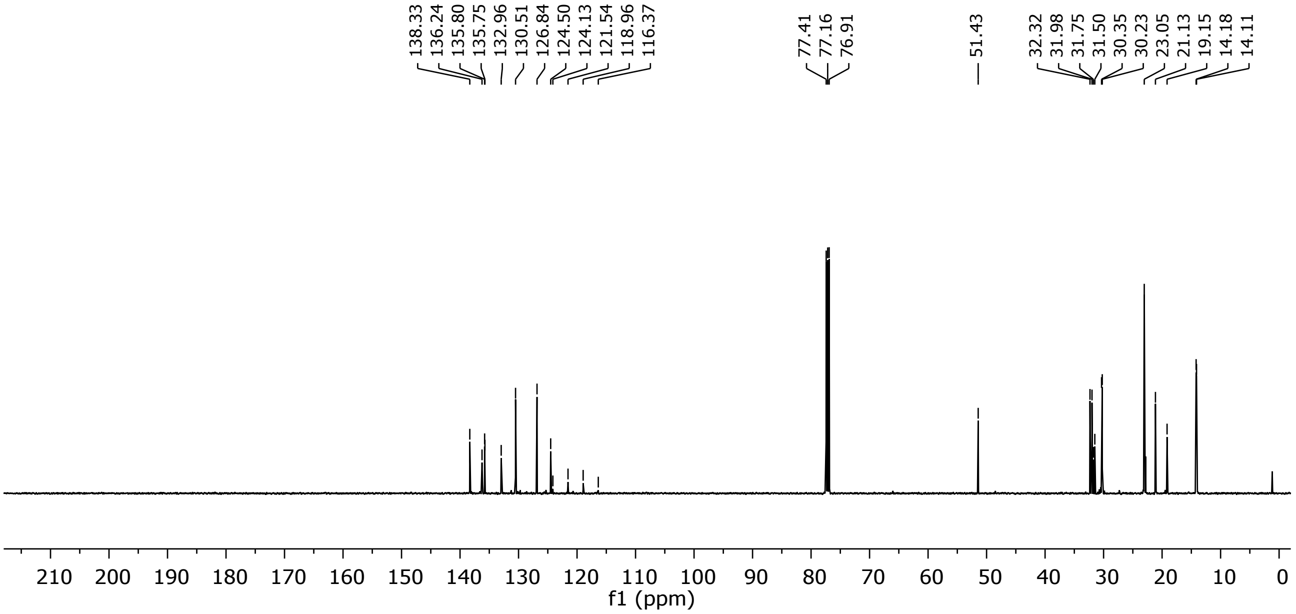

^{1}H NMR (300 MHz, CDCl$_3$)

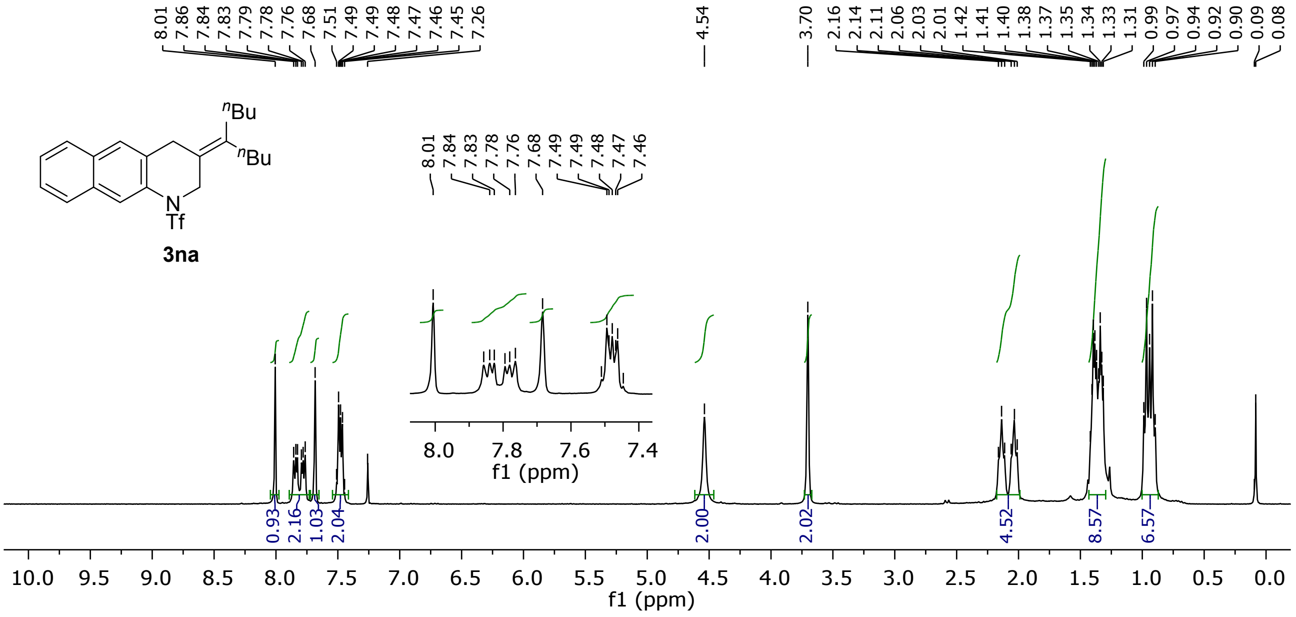

DEPT-135

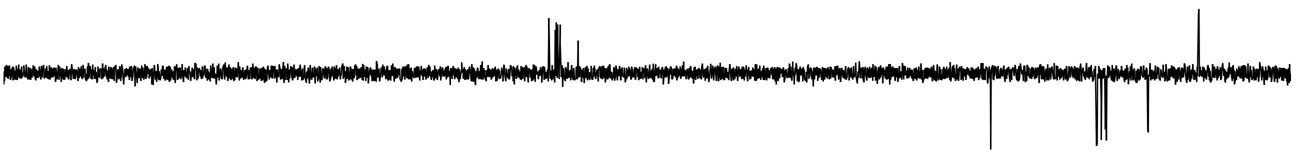

^{13}C NMR (75 MHz, CDCl$_3$)

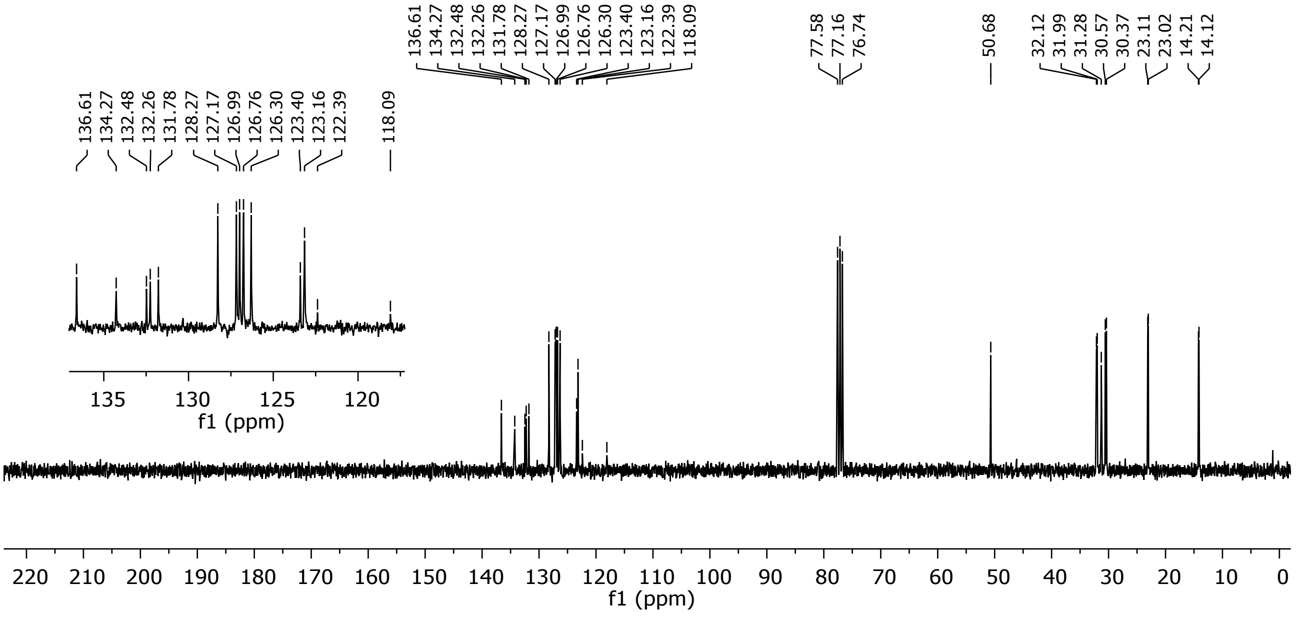

^{1}H NMR (500 MHz, $CDCl_3$)

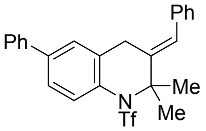

3fh

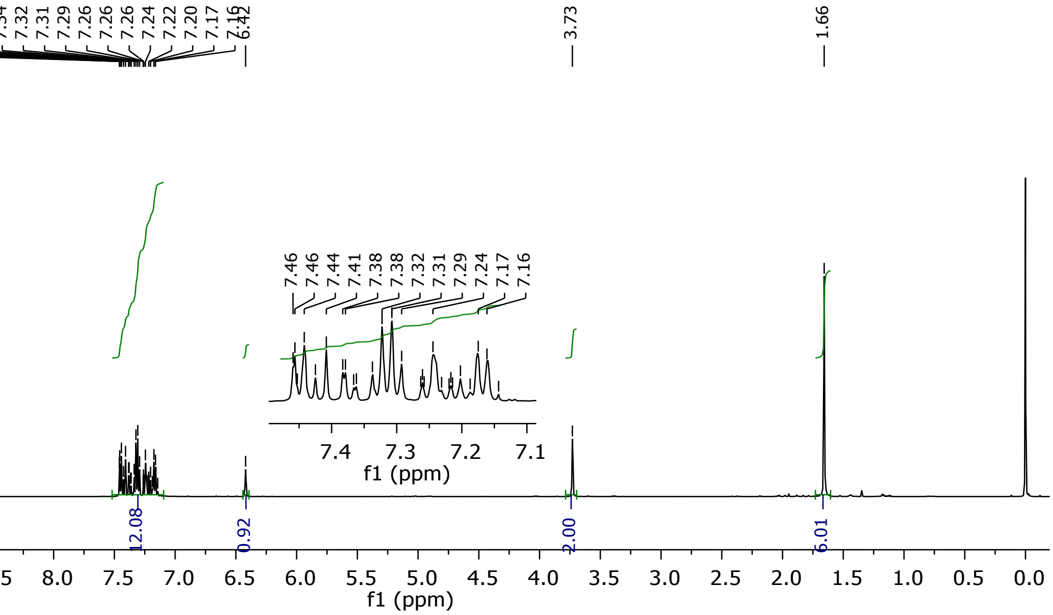

DEPT-135

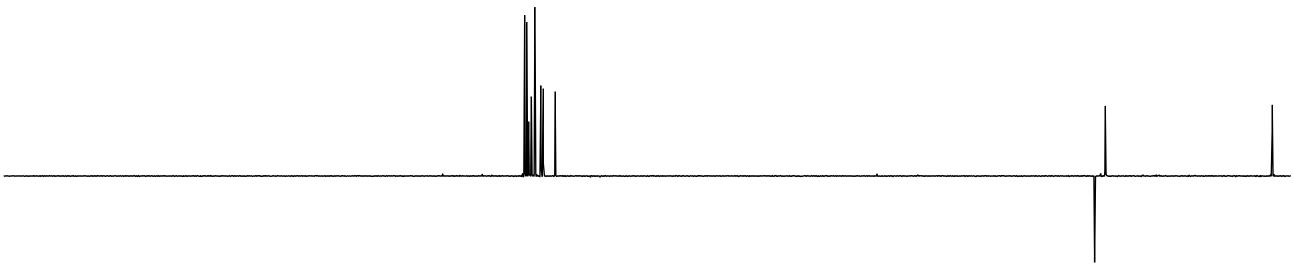

^{13}C NMR (126 MHz, $CDCl_3$)

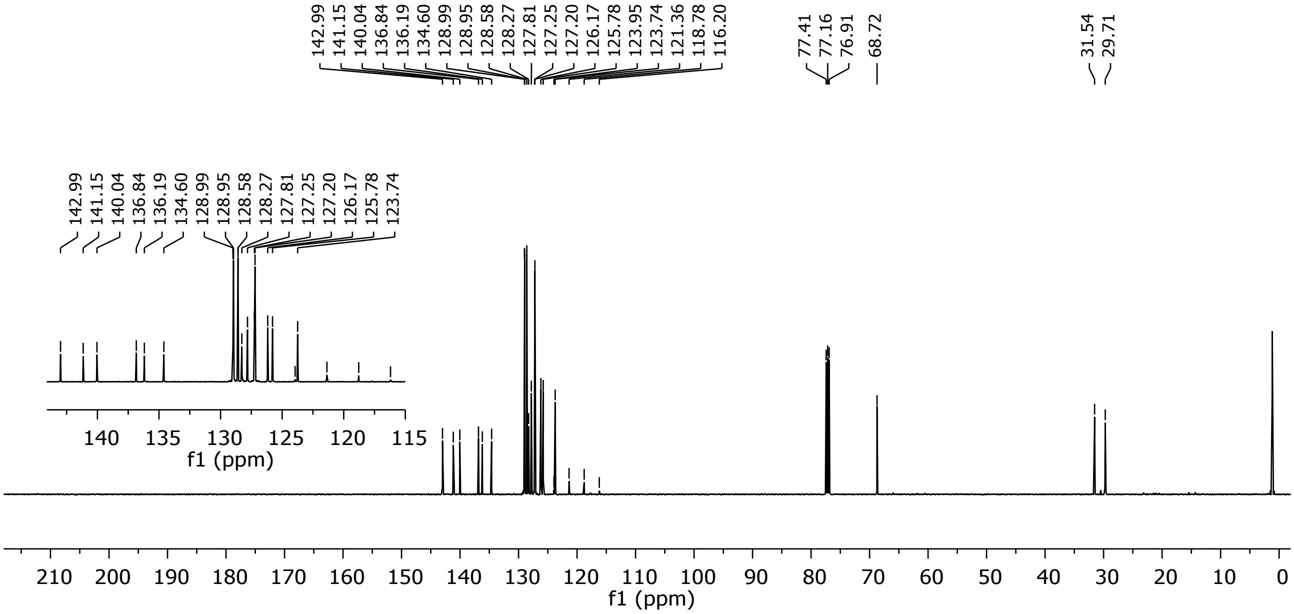

^{1}H NMR (300 MHz, $CDCl_3$)

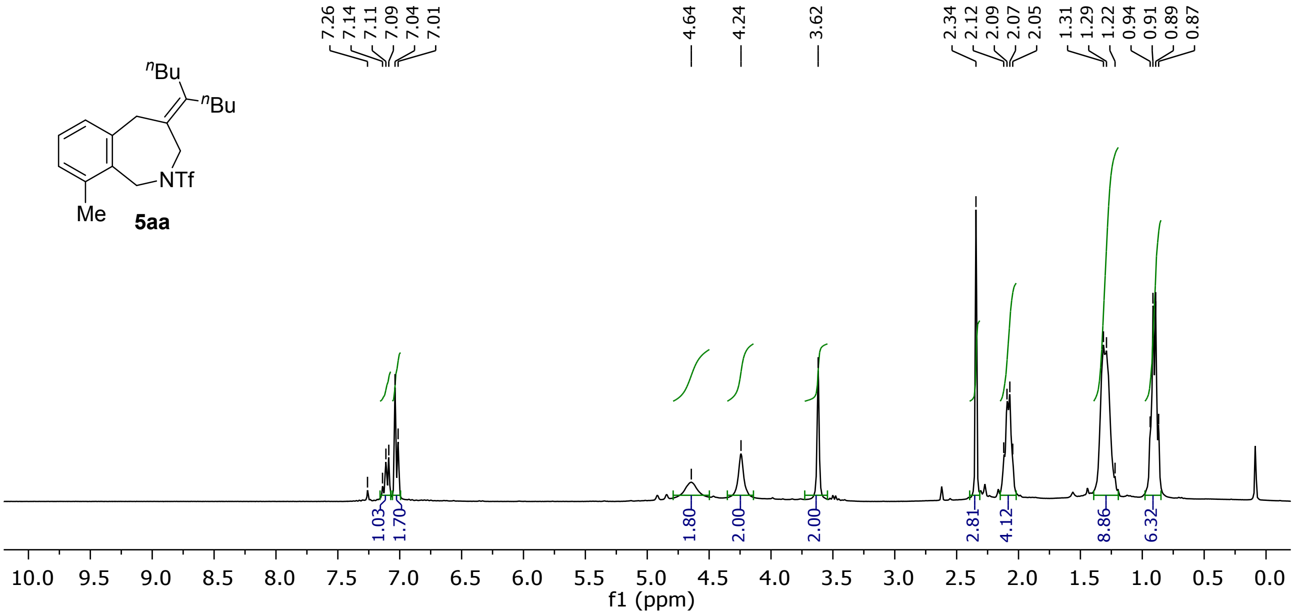

DEPT-135

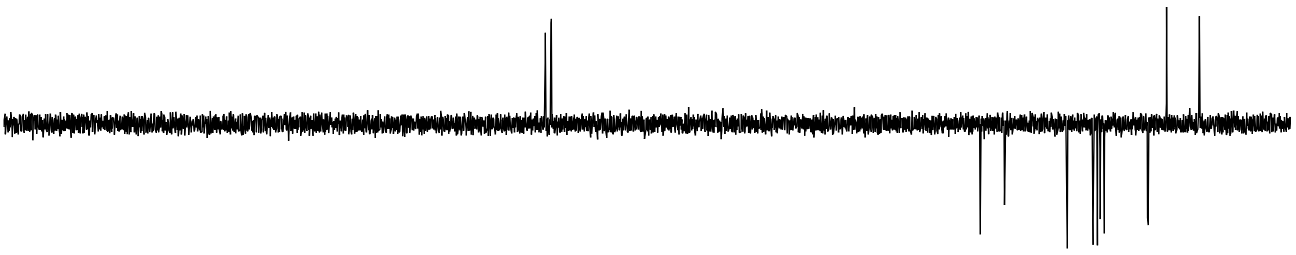

^{13}C NMR (75 MHz, $CDCl_3$)

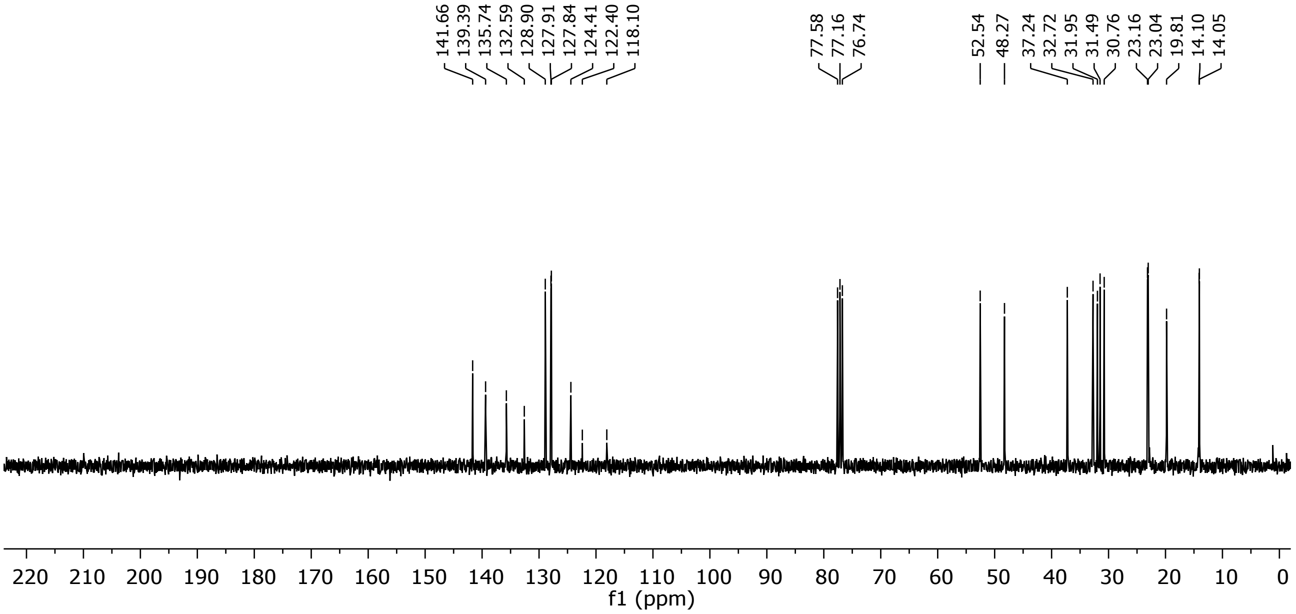

^{1}H NMR (300 MHz, $CDCl_3$)

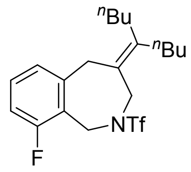

5ba

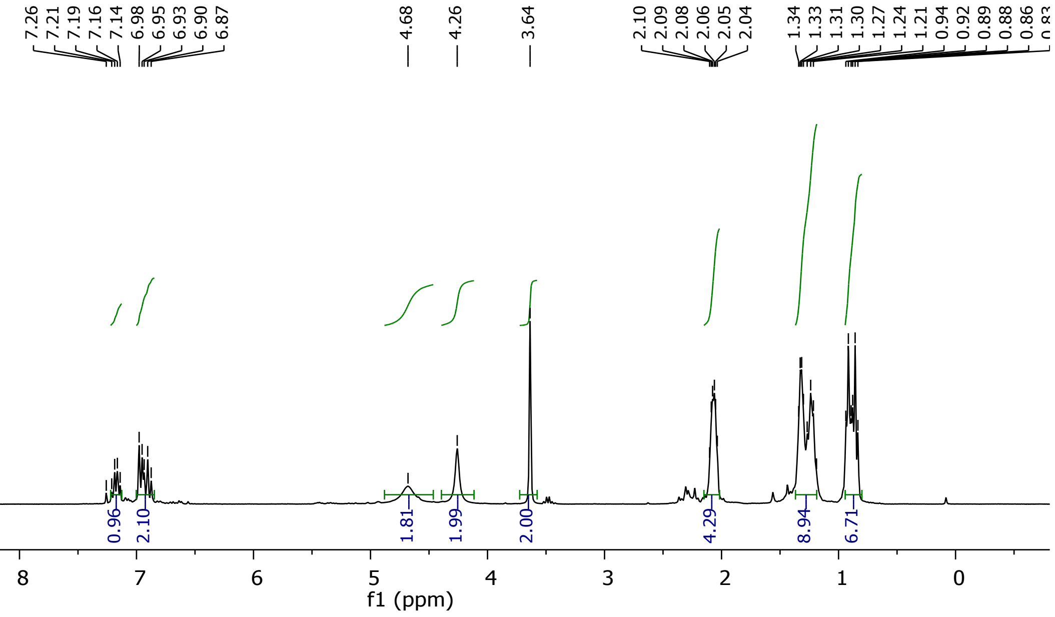

DEPT-135

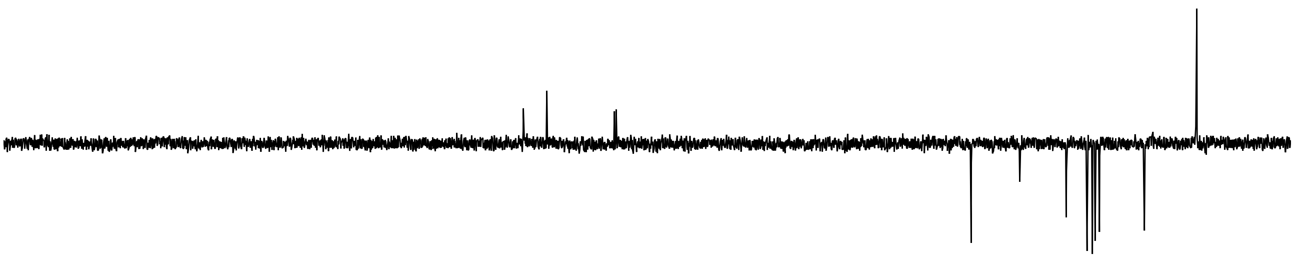

^{13}C NMR (75 MHz, $CDCl_3$)

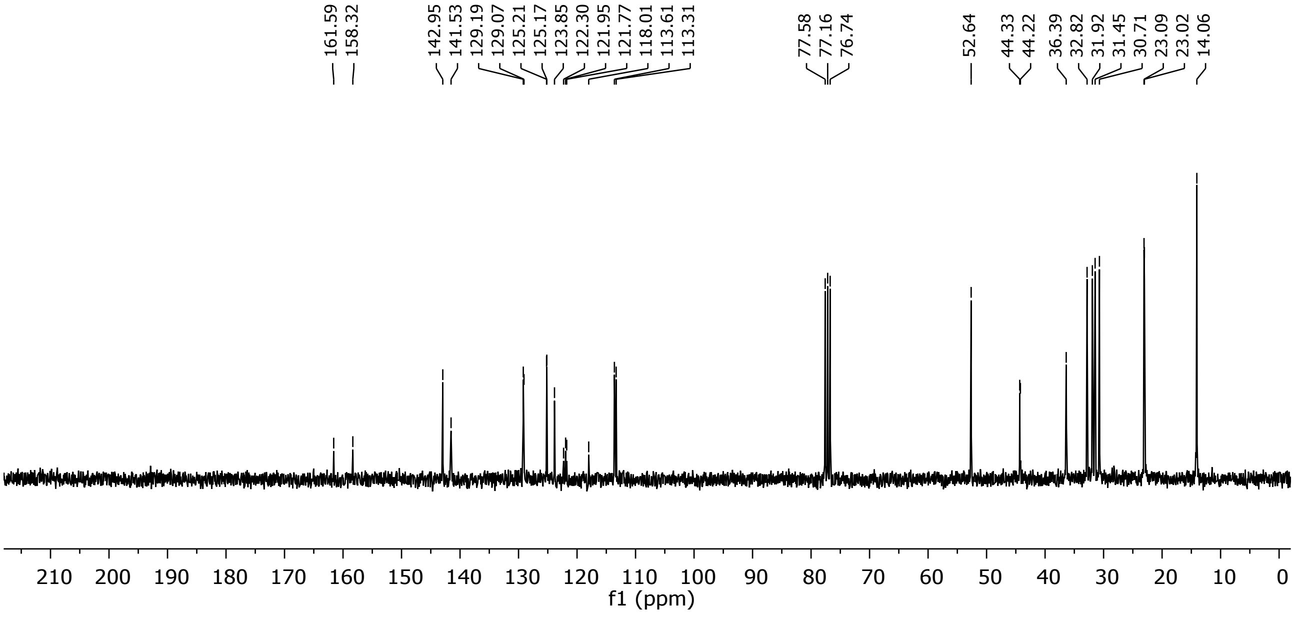

^{1}H NMR (500 MHz, CDCl$_3$)

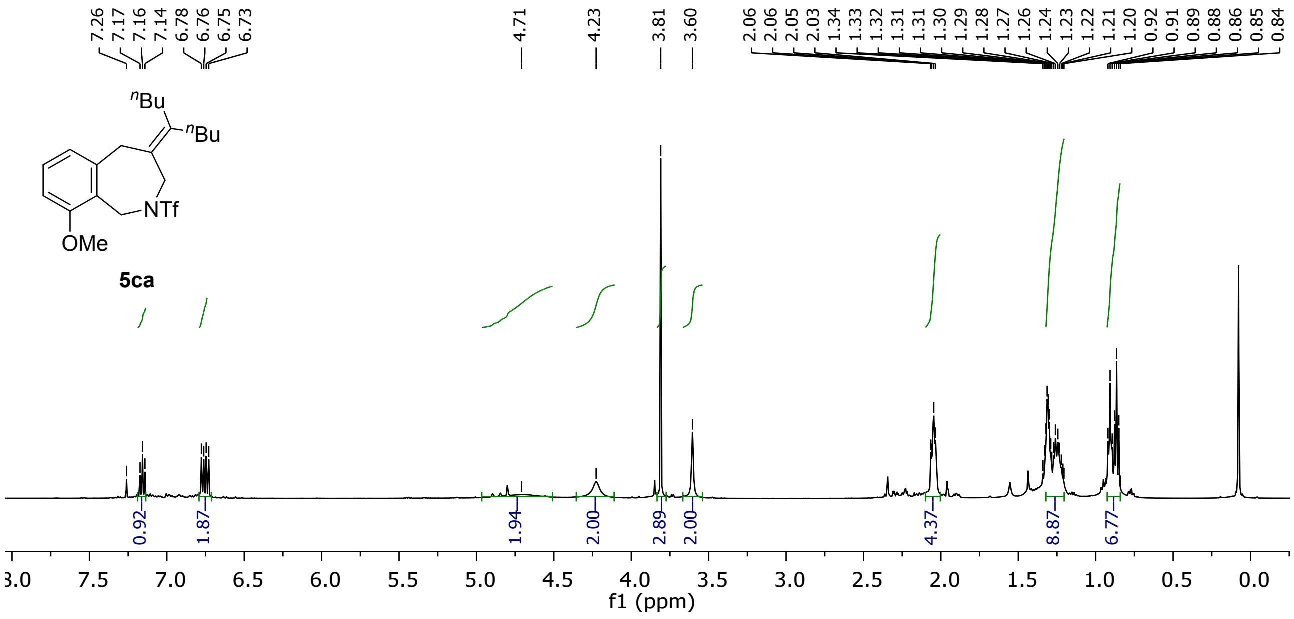

DEPT-135

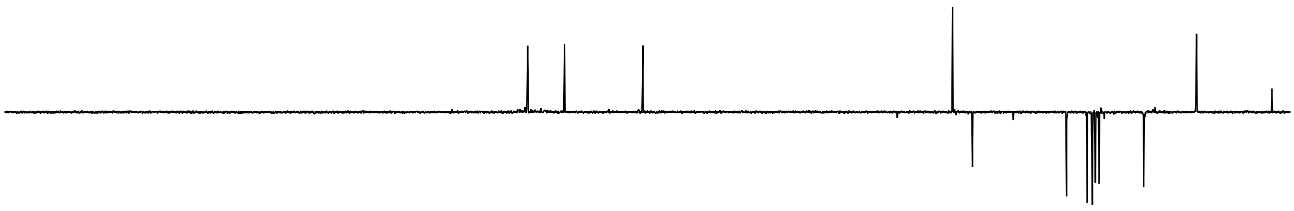

^{13}C NMR (126 MHz, CDCl$_3$)

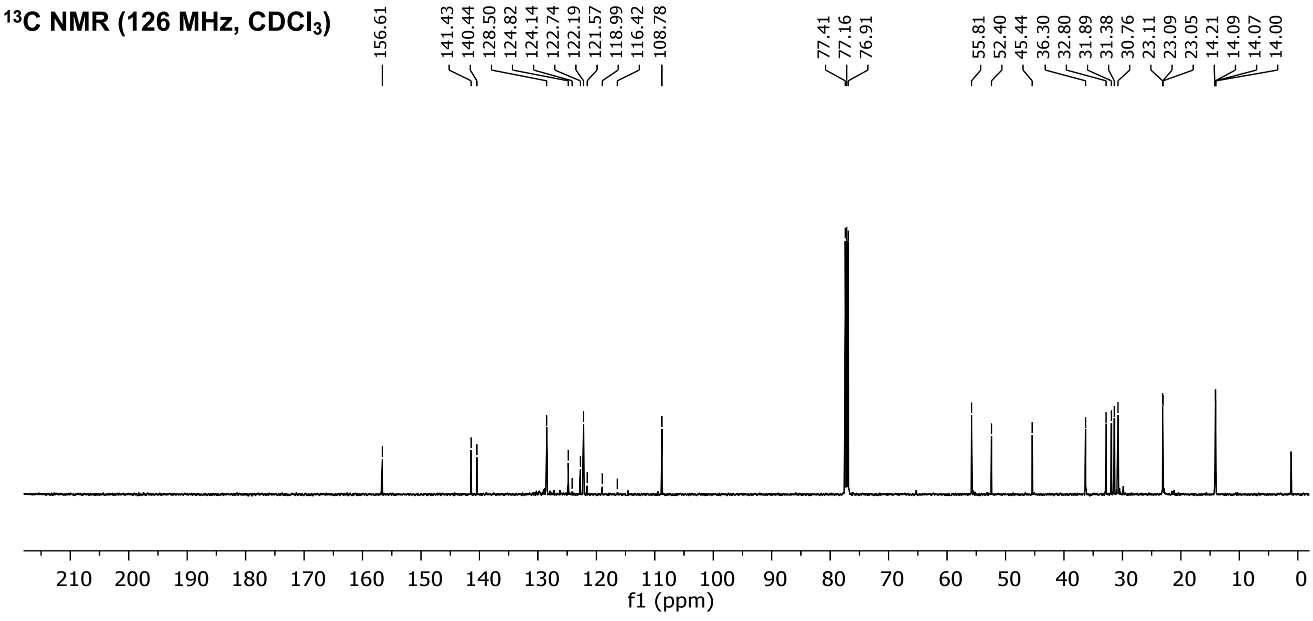

^{1}H NMR (300 MHz, CDCl$_3$)

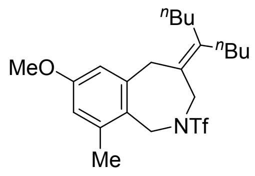

5da

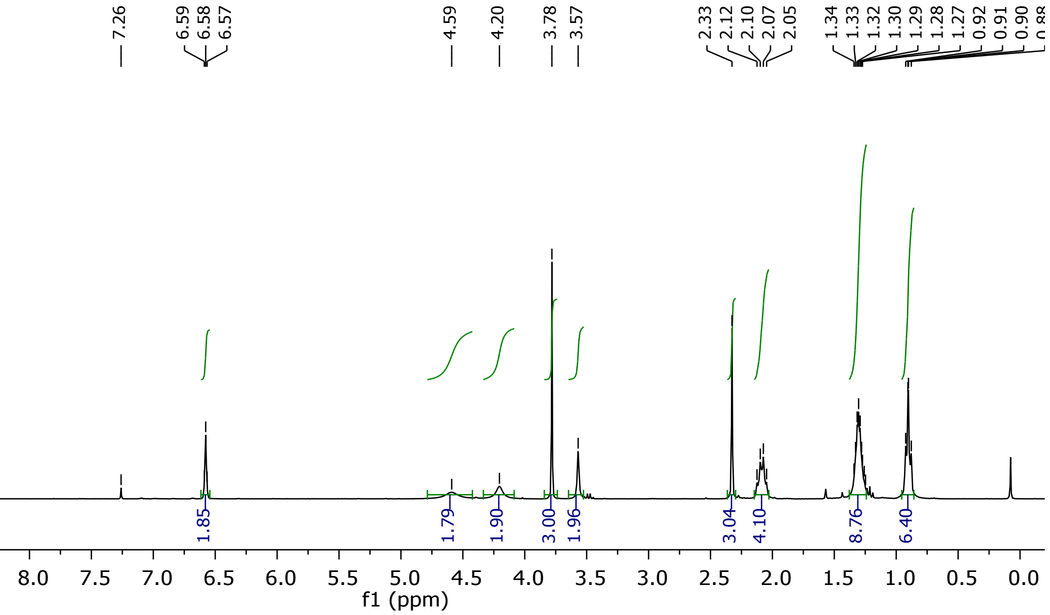

DEPT-135

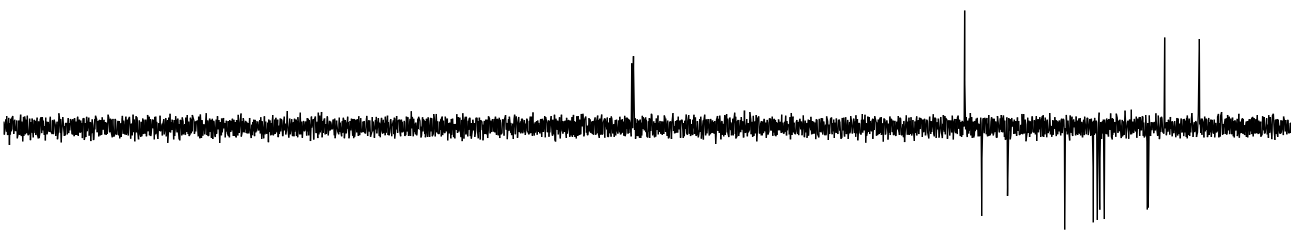

^{13}C NMR (75 MHz, CDCl$_3$)

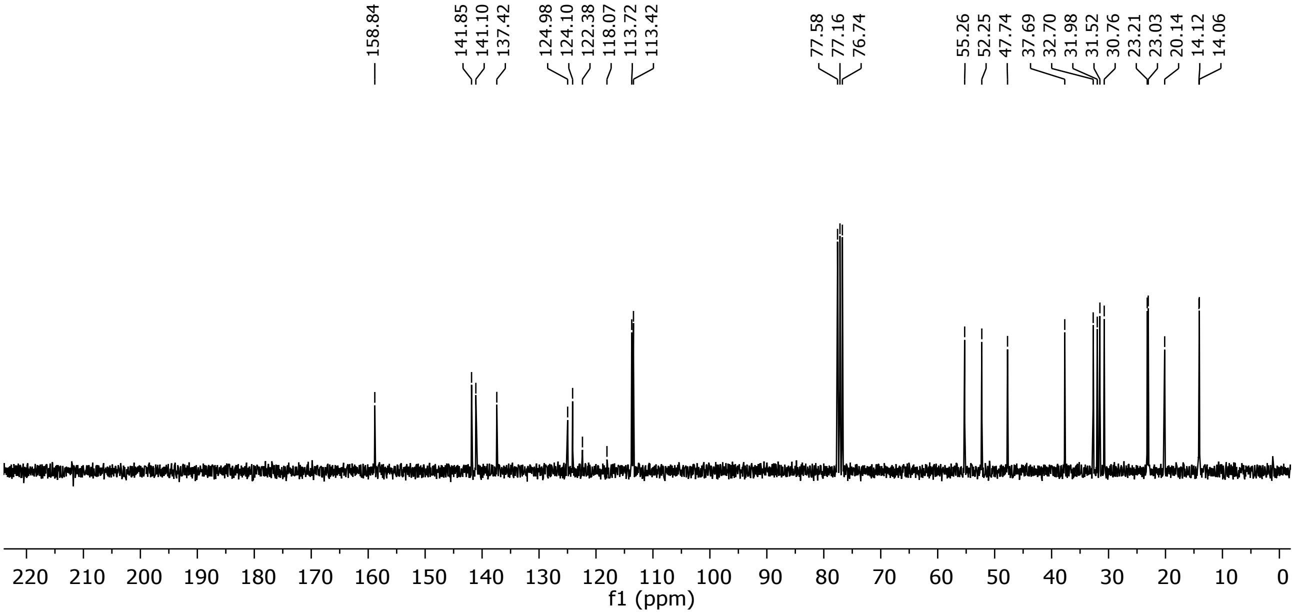

^{1}H NMR (500 MHz, $CDCl_3$)

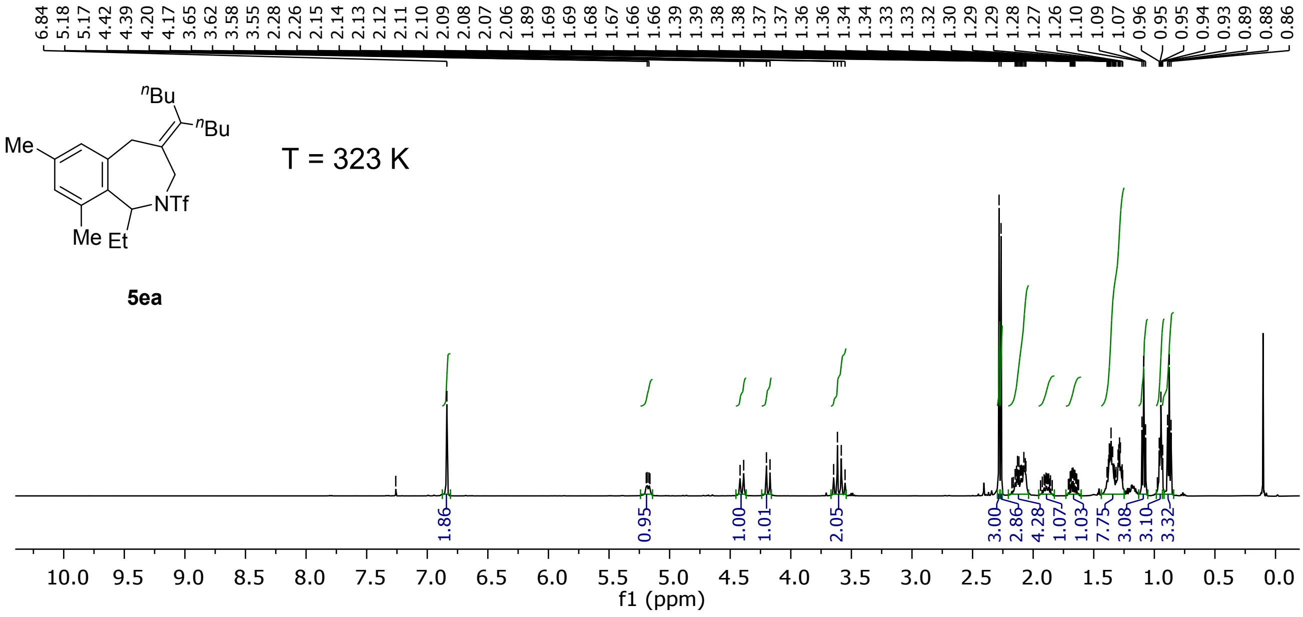

DEPT-135

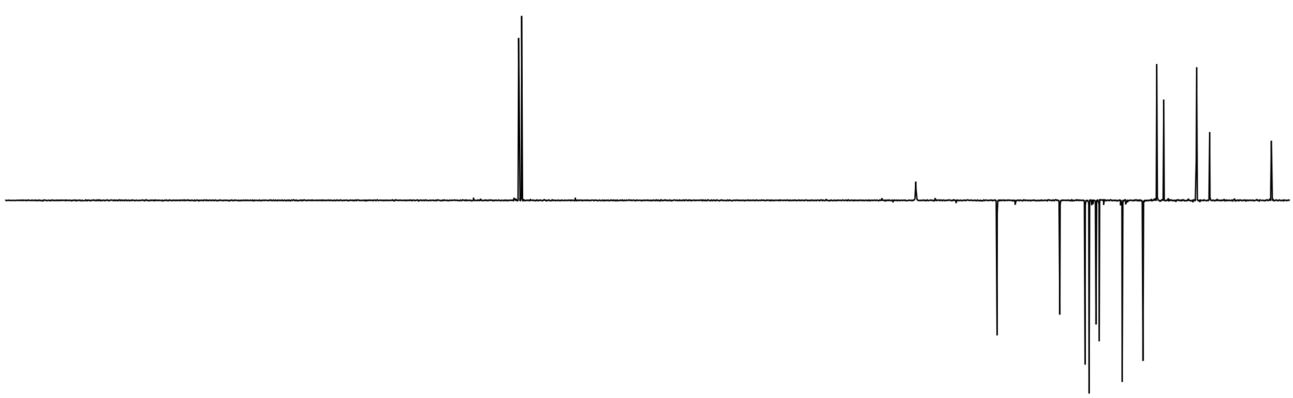

^{13}C NMR (126 MHz, $CDCl_3$)

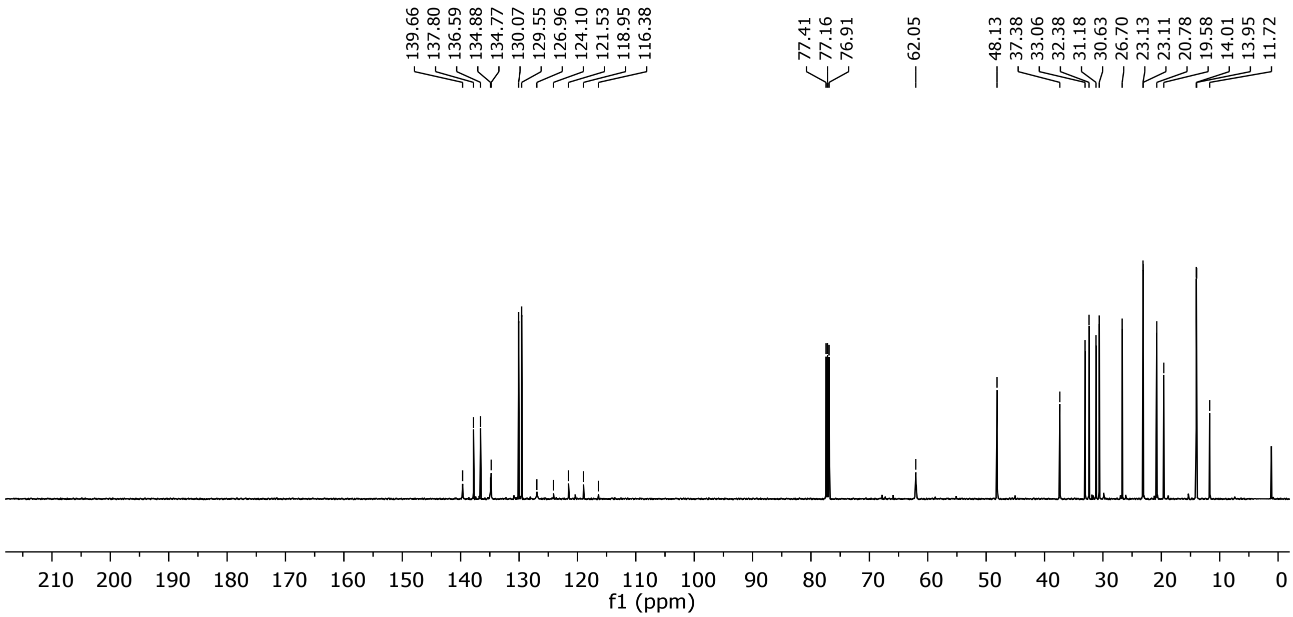

^{1}H NMR (300 MHz, $CDCl_3$)

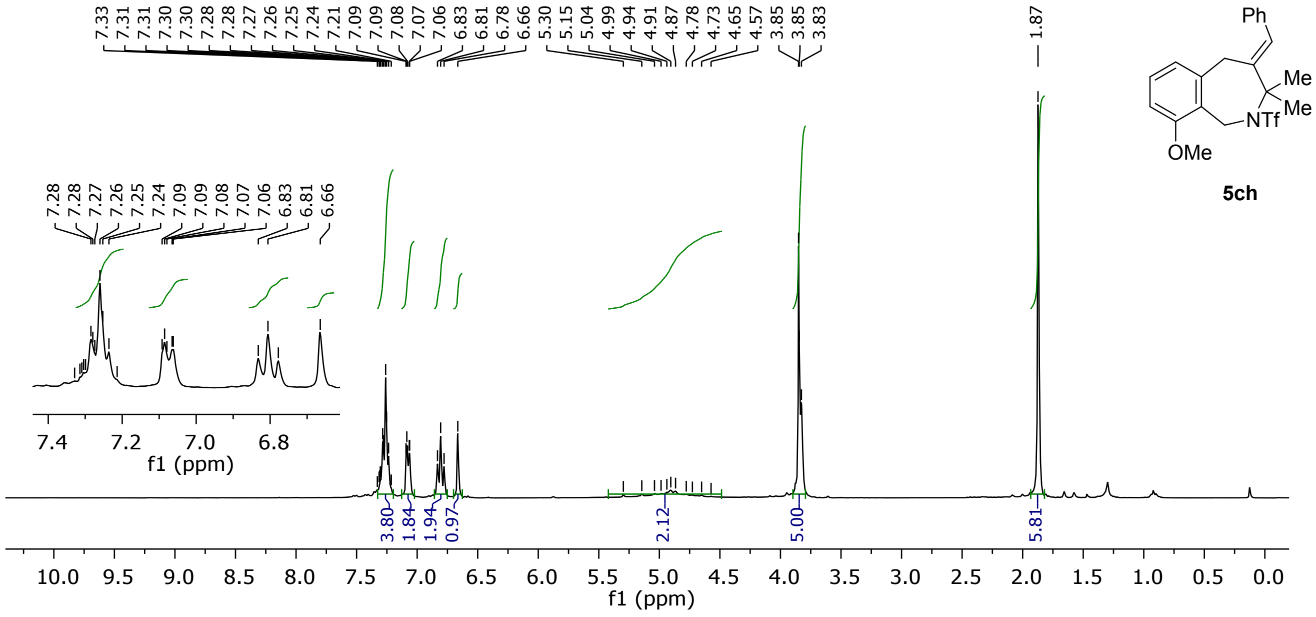

5ch

DEPT-135

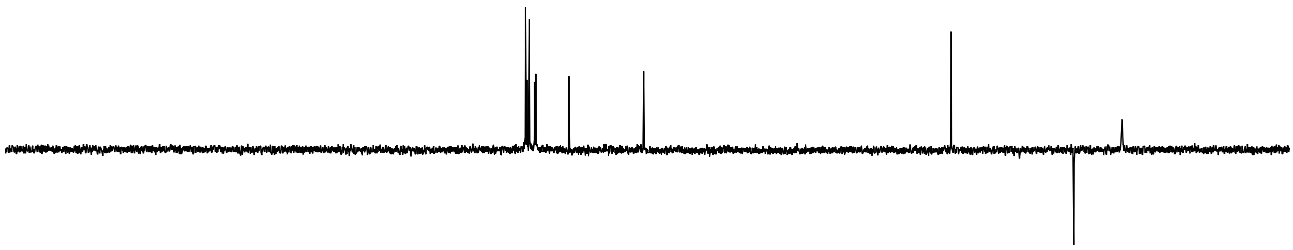

^{13}C NMR (75 MHz, $CDCl_3$)

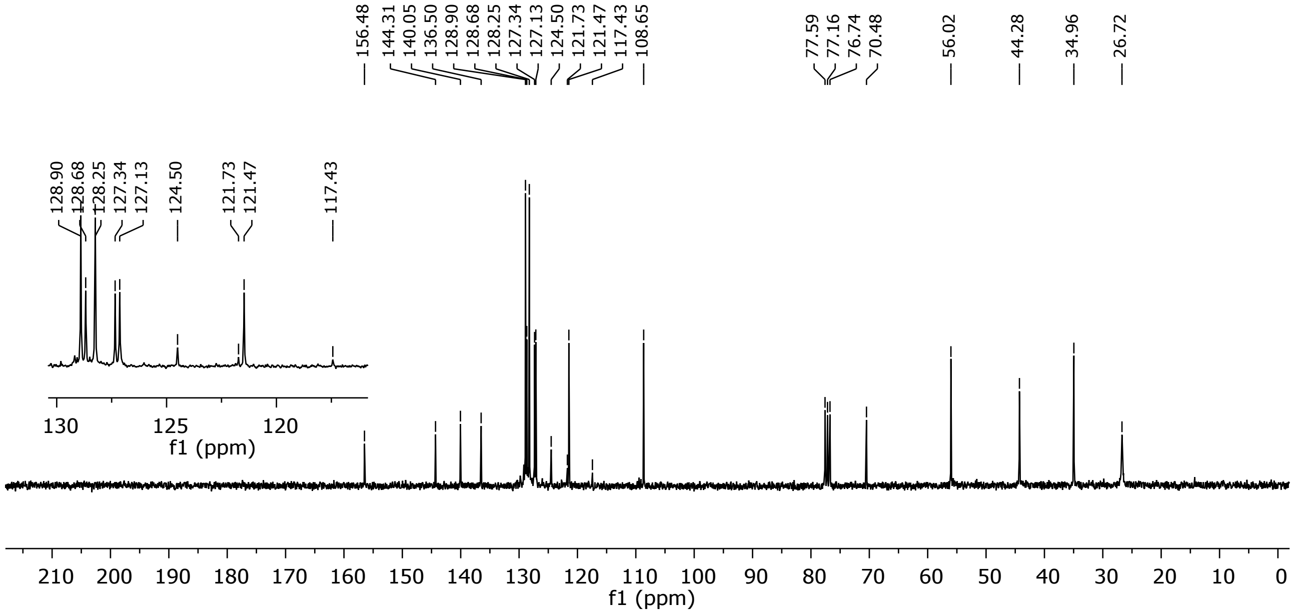

^{1}H NMR (500 MHz, $CDCl_3$)

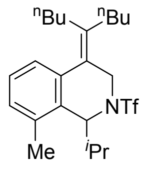

8

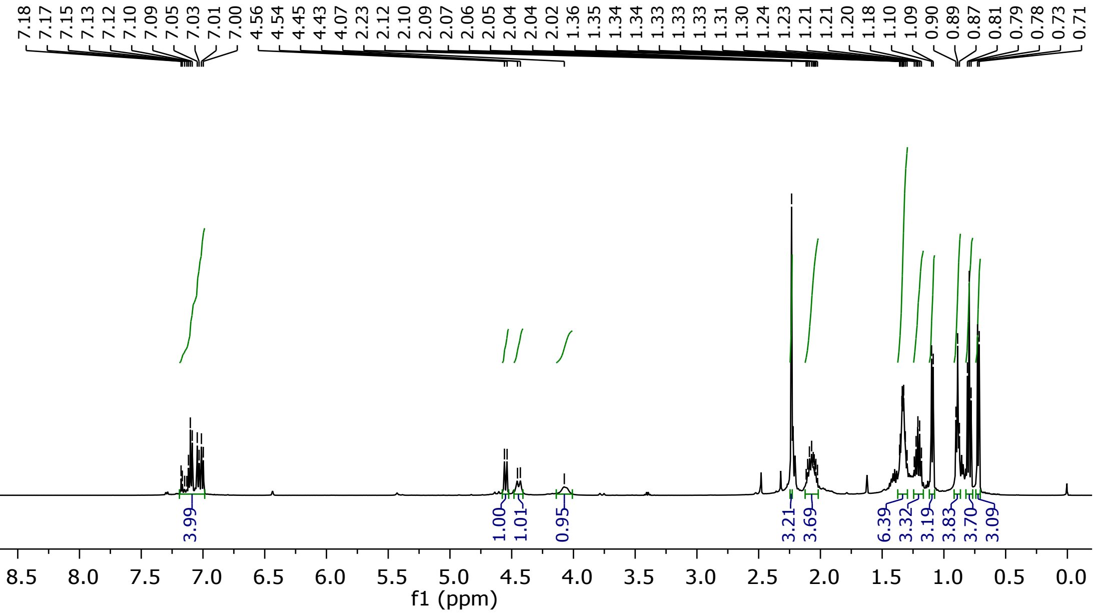

DEPT-135

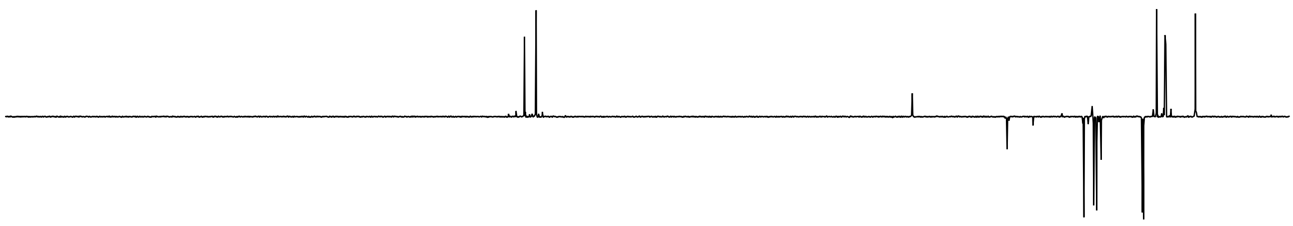

^{13}C NMR (126 MHz, $CDCl_3$)

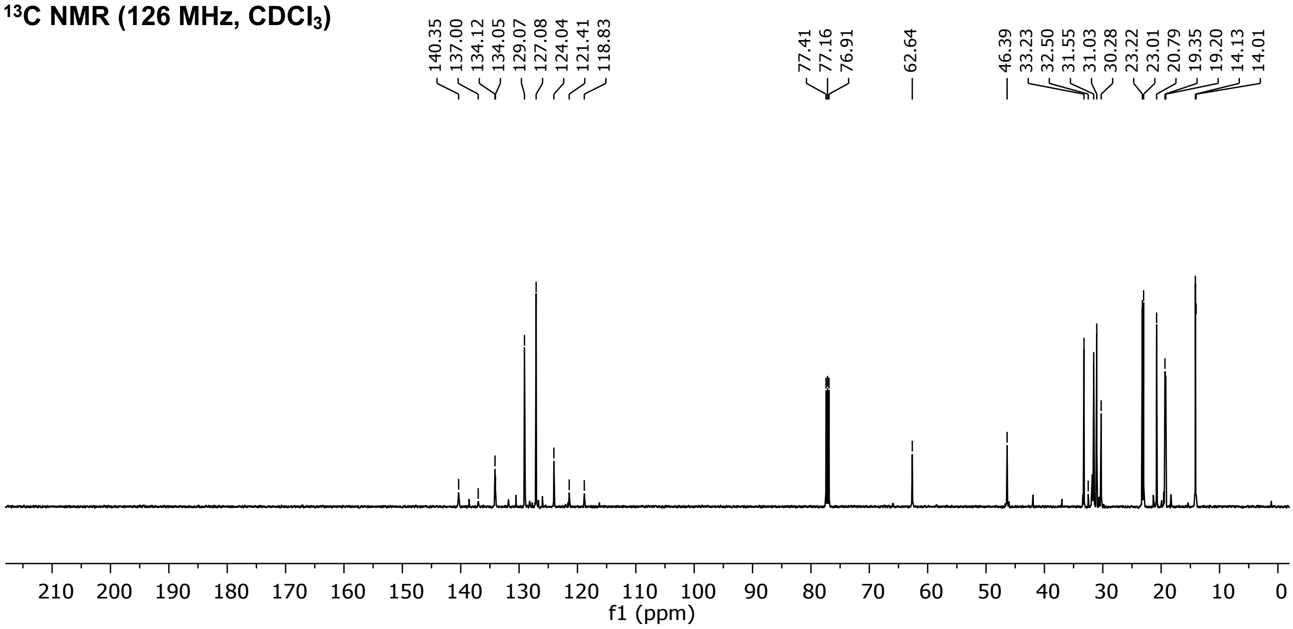

^{1}H NMR (300 MHz, $CDCl_3$)

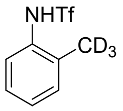

1a-*d*

[2]D NMR (46 MHz, $CDCl_3$)

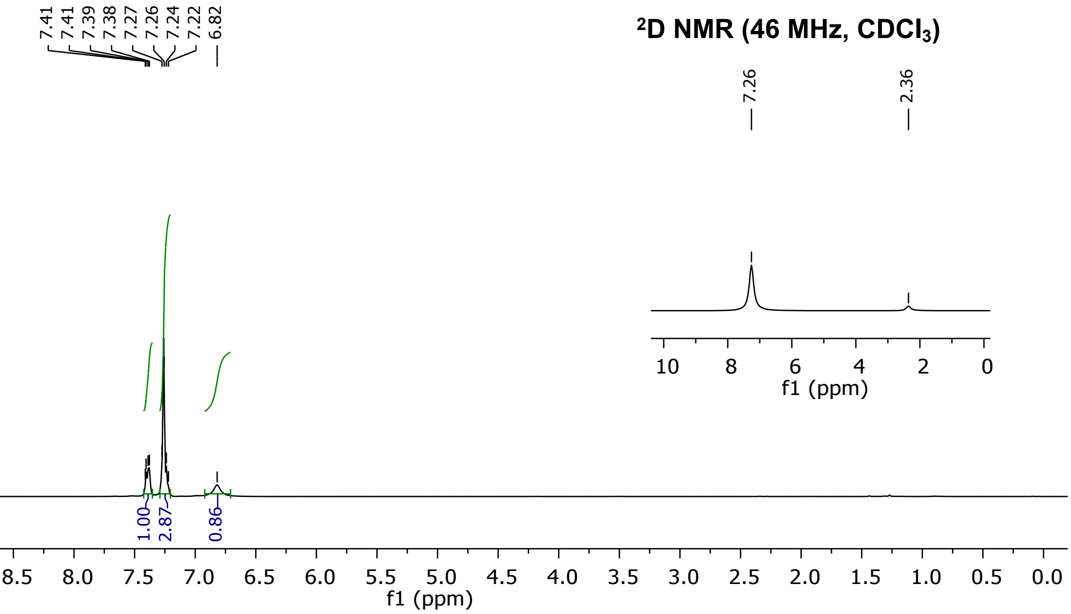

DEPT-135

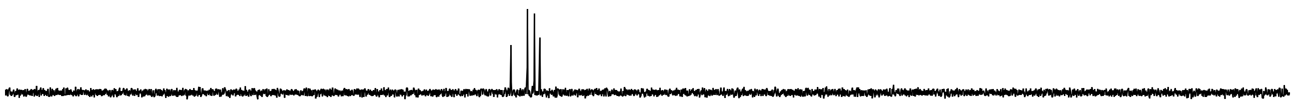

^{13}C NMR (75 MHz, $CDCl_3$)

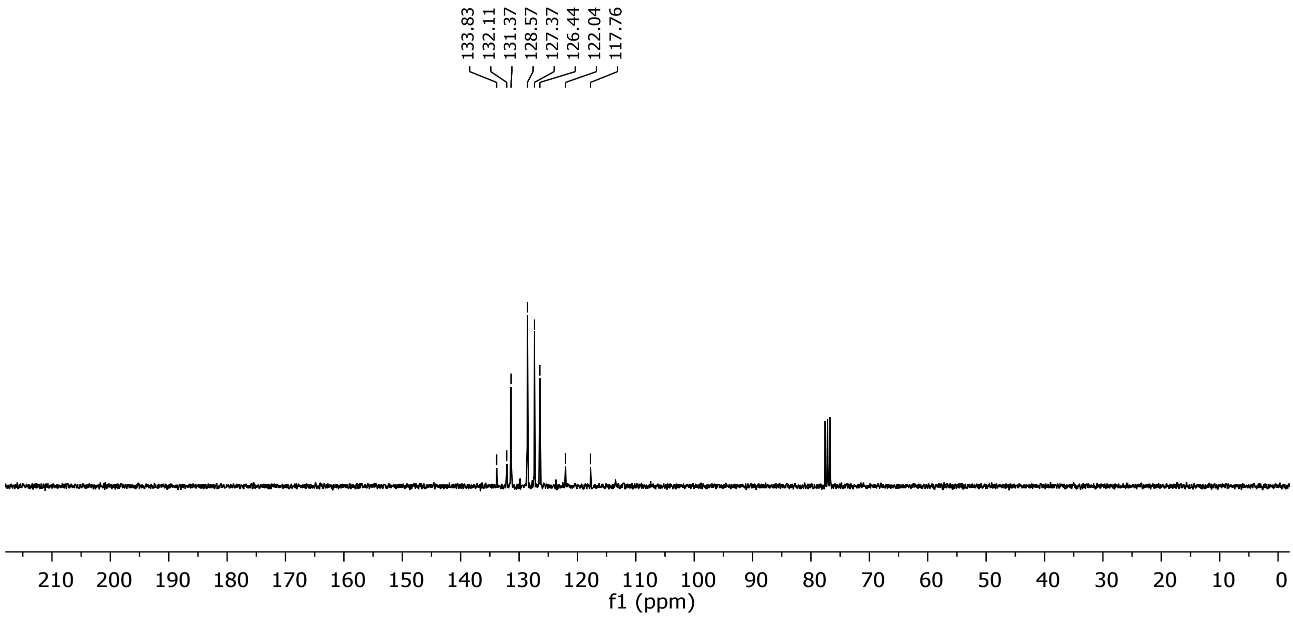

^{1}H NMR (300 MHz, $CDCl_3$)

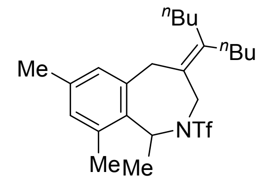

5fa

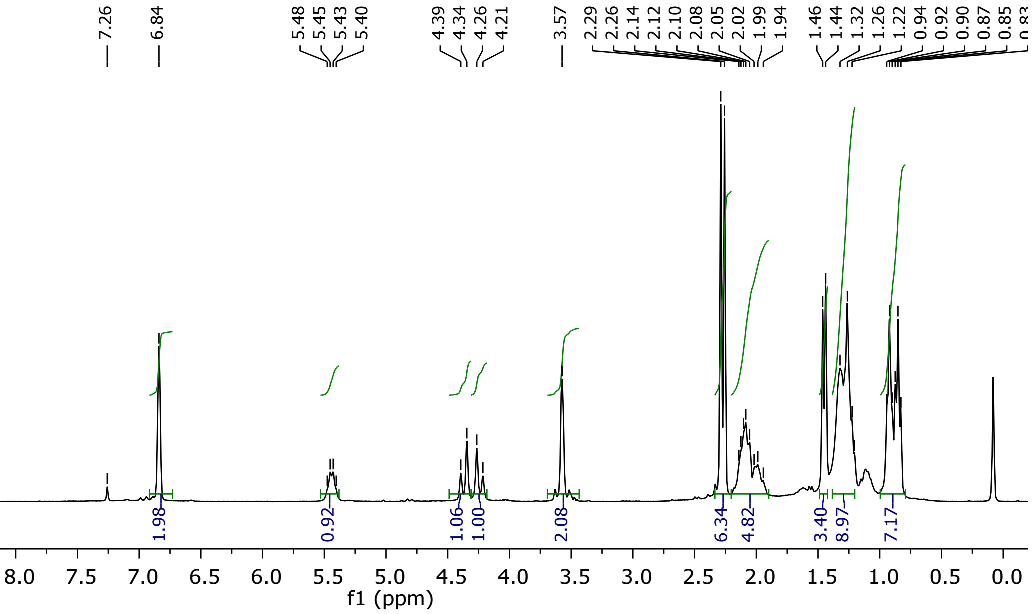

DEPT-135

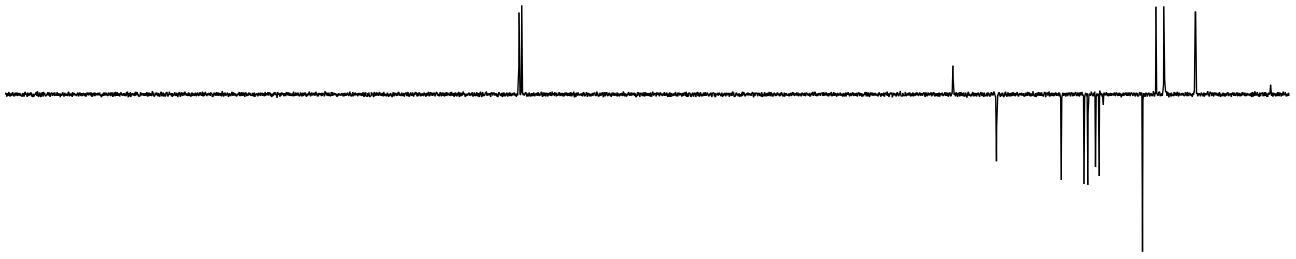

^{13}C NMR (75 MHz, $CDCl_3$)

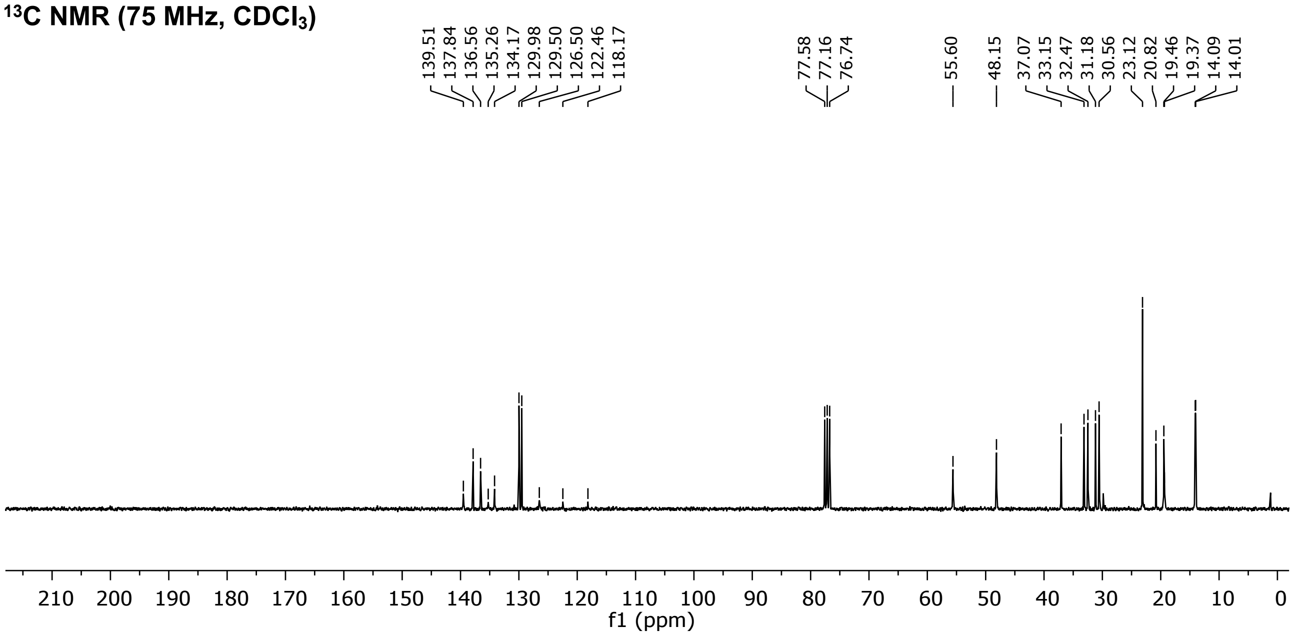

^{1}H NMR (300 MHz, $CDCl_3$)

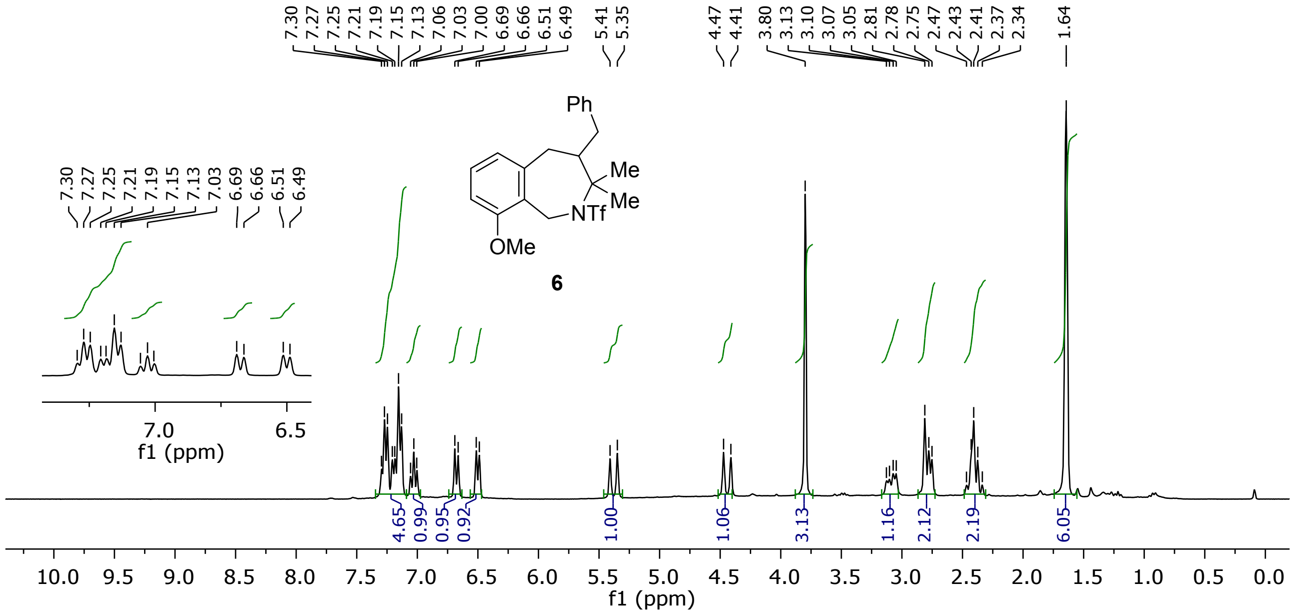

DEPT-135

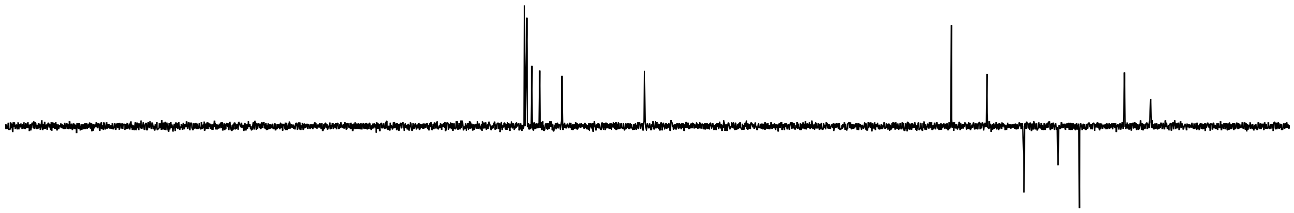

^{13}C NMR (75 MHz, $CDCl_3$)

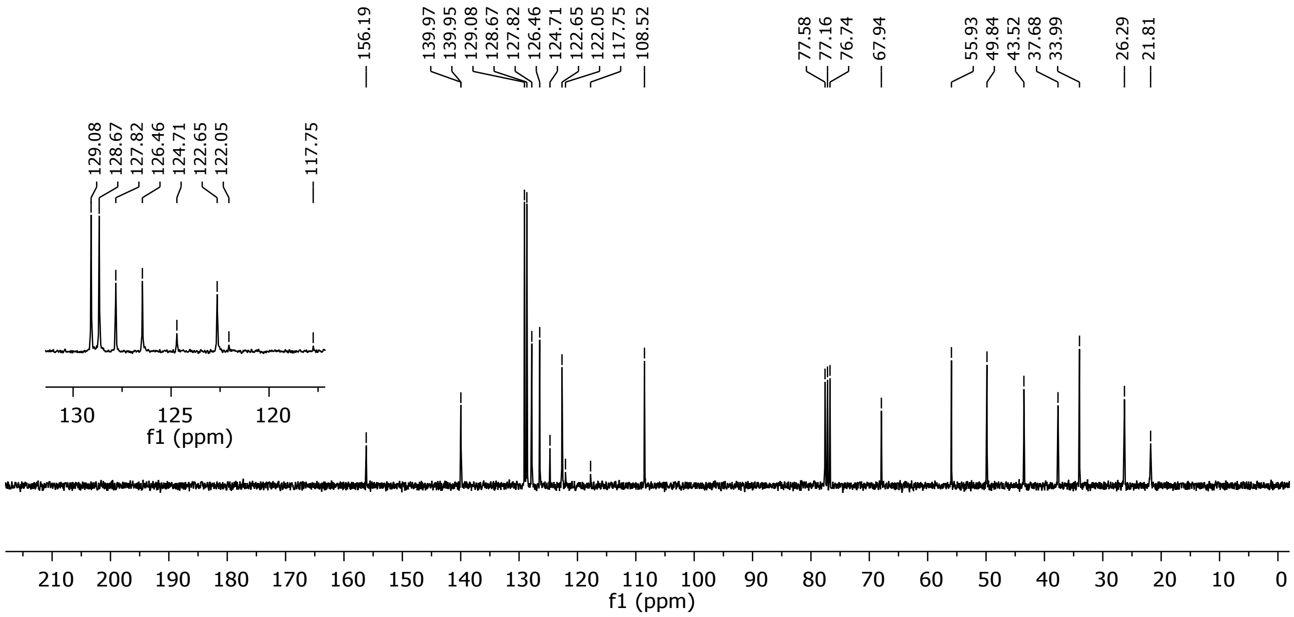

^{1}H NMR (300 MHz, $CDCl_3$)

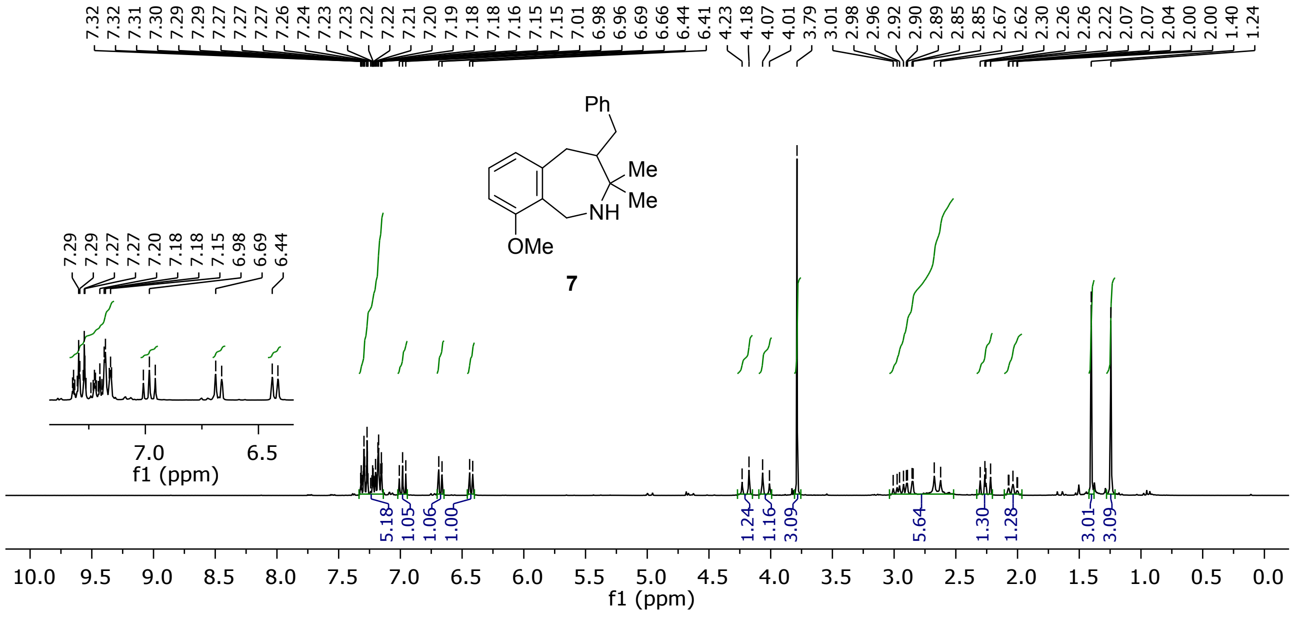

DEPT-135

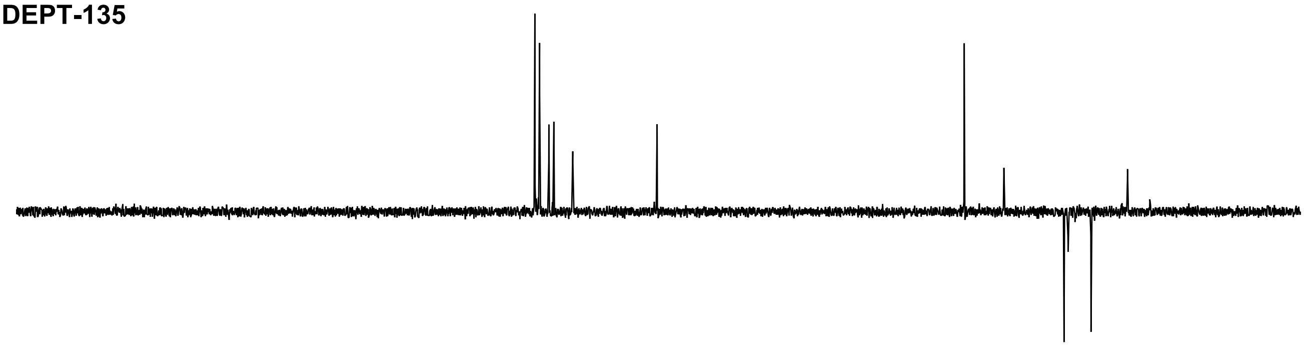

^{13}C NMR (75 MHz, $CDCl_3$)

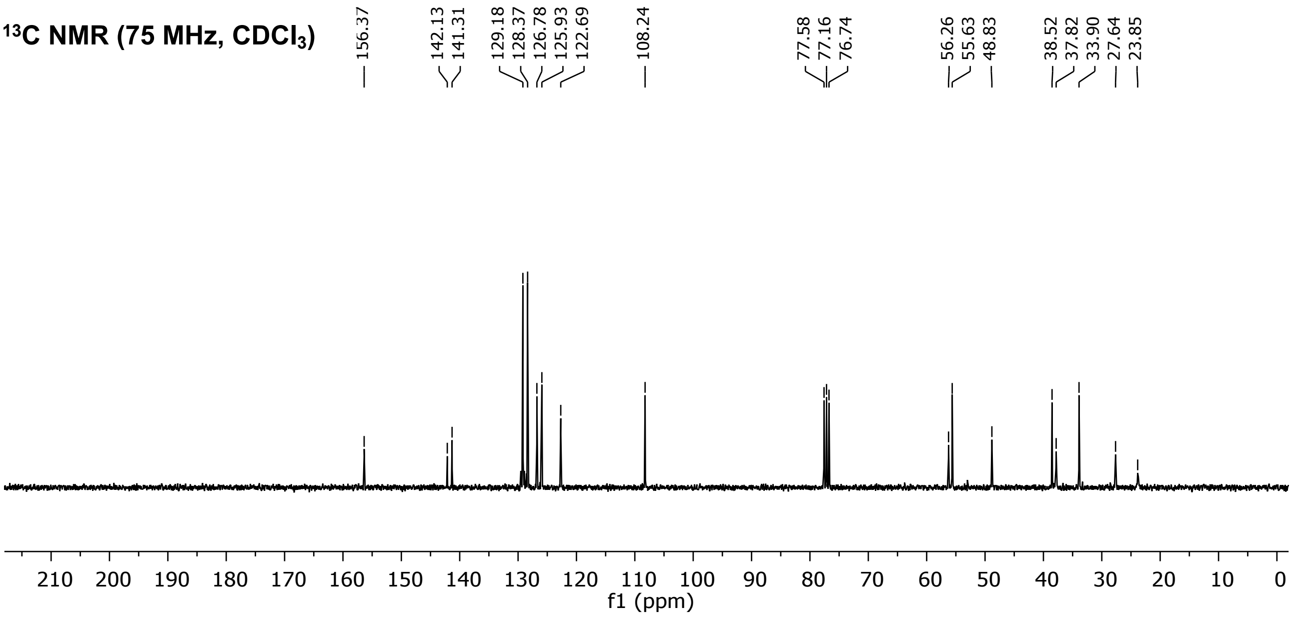

Supplement: Supplementary file 1 — ol1c01594_si_001.pdf [file ol1c01594_si_001.pdf]
